# Previously undocumented diversity and abundance of cryptic species: a phylogenetic analysis of Indo-Pacific Arminidae Rafinesque, 1814 (Mollusca: Nudibranchia) with descriptions of 20 new species of *Dermatobranchus*

**DOI:** 10.1111/j.1096-3642.2010.00649.x

**Published:** 2011-02

**Authors:** Terrence M Gosliner, Shireen J Fahey

**Affiliations:** 1 California Academy of Sciences55 Music Concourse Drive, San Francisco, CA 94118, USA; 2 University of the Sunshine CoastMaroochydore DC QLD 4558, Australia

**Keywords:** Arminina, biodiversity, biogeography, Indo-Pacific, systematics, trophic diversification

## Abstract

The phylogenetic relationships amongst the Arminidae were analysed based upon morphological characters of 58 presently described species or nudibranchs, including 35 previously described Arminidae and 20 new species of *Dermatobranchus*. From the literature review and anatomical examinations, 43 characters were considered for 78 taxa. These characters were polarized using *Berthella canariensis* as the outgroup taxon and the type species of several other genera identified from recent publications. The resulting phylogeny supports the monophyly of Arminidae, *Dermatobranchus*, Doridina, and Proctonotidae. The paraphyly of the Arminina is further demonstrated in this study. Two previously described, but poorly known, species of Indo-Pacific *Armina* are redescribed, *Armina magna* Baba, 1955 and *Armina paucifoliata* Baba, 1955. The anatomy and taxonomic status of nine previously described species of *Dermatobranchus* were examined in this study. The anatomy of *Dermatobranchus pustulosus* (van Hasselt, 1824) has been overlooked since Bergh (1888) illustrated the radula of van Hasselt's specimen. It is redescribed and its range is extended to several new localities in the western Pacific. *Dermatobranchus pulcherrimus* Miller & Willan, 1986 is considered here as a new synonym of *Dermatobranchus rubidus* (Gould, 1852). The following 20 species of *Dermatobranchus* are new and are described in the present paper: ***Dermatobranchus albineus* sp. nov.**, ***Dermatobranchus arminus* sp. nov.**, ***Dermatobranchus caesitius* sp. nov.**, ***Dermatobranchus caeruleomaculatus* sp. nov.**, ***Dermatobranchus cymatilis* sp. nov.**, ***Dermatobranchus dendonephthyphagus* sp. nov.**, ***Dermatobranchus diagonalis* sp. nov.**, ***Dermatobranchus earlei* sp. nov.**, ***Dermatobranchus fasciatus* sp. nov.**, ***Dermatobranchus funiculus* sp. nov.**, ***Dermatobranchus kalyptos* sp. nov.**, ***Dermatobranchus kokonas* sp. nov.**, ***Dermatobranchus leoni* sp. nov.**, ***Dermatobranchus microphallus* sp. nov.**, ***Dermatobranchus oculus* sp. nov.**, ***Dermatobranchus phyllodes* sp. nov.**, ***Dermatobranchus piperoides* sp. nov.**, ***Dermatobranchus rodmani* sp. nov.**, ***Dermatobranchus semilunus* sp. nov.**, and ***Dermatobranchus tuberculatus* sp. nov.** Eighteen of these new taxa are found in the Indo-Pacific tropics and two are found in temperate South Africa, *D. albineus* and *D. arminus*. Unique combinations of morphological characters distinguish these as new species of *Dermatobranchus*. Several species that are externally similar have radically divergent internal morphology, are members of different clades of *Dermatobranchus*, and represent cryptic species. Especially important is the radular morphology, which shows remarkable diversity of form, probably related directly to the diversification of feeding of members of this clade on various octocorals.

## INTRODUCTION

Recent surveys of several localities throughout the Indo-Pacific tropics and from temperate southern Africa have produced an abundance of specimens of *Dermatobranchus* species. Few of these specimens can be identified as previously described taxa and the majority of specimens represent undescribed species. Specimens from these collections representing 20 undescribed species are here described, doubling the known diversity of the taxon. Many of these taxa have similar external anatomy, but radically divergent internal anatomy and are therefore considered to represent cryptic species. Detailed comparison of these species provides critical new information for discerning these taxa.

Six genera have traditionally been recognized within the Arminidae: *Armina* Rafinesque, 1814, *Dermatobranchus* Hasselt, 1824, *Histiomena* Mörch, 1859, *Linguella* Blainville, 1823, *Pleurophyllidiella* Eliot, 1903, and *Pleurophyllidiopsis* Tchang-Si, 1934 (Kolb & Wägele, 1998). However, some recent publications (see for example Willan, 1997) suggest that only three genera should be included in the Arminidae (*Armina*, *Dermatobranchus*, and *Heterodoris*).

Early authors presented summaries of the characteristics that distinguish the genera of Arminidae (Odhner, 1934; Pruvot-Fol, 1937; Odhner, 1939; MacFarland, 1966; Marcus & Marcus, 1966). However, some of the characters that these authors cited are not unique to the group, but are also found in other nudibranch taxa. These characters included for example the shape of the rhinophores and the presence of hyponotal lamellae. Kolb & Wägele (1998) followed these early authors with a table of characters, along with a discussion of those characters and a phylogenetic analysis of the family with a detailed discussion of the history of the classification. Kolb (1998) completed a thorough examination of the morphology and histology of four species of *Armina* and made comparisons to other species of *Armina*. Her results showed little variation amongst species with regard to external characters such as number of notal ridges, caruncle shape, and number of rhinophoral lamellae. Other characters that had historically been considered to distinguish amongst species, such as rows of jaw rods and ampulla morphology were also shown to provide little phylogenetic information. Kolb & Wägele (1998) concluded that only two morphological characters unite the Arminidae, the presence of marginal sacs and rhinophores with longitudinal lamellae.

Several recent publications have contributed considerably to the knowledge of the Arminina (Gosliner, 1981; Miller & Willan, 1986; Gosliner & Behrens, 1996; Jensen, 1997; Kolb, 1998; Ardila & Valdés, 2004). This species-rich group of nudibranchs has a worldwide distribution, with species described from all oceans (see Kolb & Wägele, 1998 for a discussion of the biogeographical distribution and map).

The present study builds on the previously published studies of the Arminidae by further examination of several morphological characters. We expand the morphological character list to test whether any new characters or combination of characters can further elucidate the phylogeny of the group. Field work over the last two decades has produced a rich diversity of new taxa from many localities in temperate South Africa and tropical localities in South Africa, Madagascar, Reunion Island, Oman, Malaysia, Philippines, Papua New Guinea, Indonesia, and Okinawa. Twenty new Indo-Pacific and South African *Dermatobranchus* species are described and compared to known species. A phylogenetic analysis is presented that incorporates the newly described species along with Arminidae from other geographical localities. Outgroup taxa were chosen from other closely related and more basally situated nudibranch groups in order to gain a better perspective of the evolution within and amongst the Arminidae.

## MATERIAL AND METHODS

### Morphological analysis

Type material and additional nontype material were obtained from the California Academy of Sciences (CASIZ) and the South African Museum (SAM A). Specimens were drawn from microscopical examination using a camera lucida attached to a dissecting microscope. Following dissection that began with a dorsal or ventral incision, the internal anatomy was examined and drawn either by compound or scanning electron microscope (SEM). External features were examined directly when specimens were available, by photographs, or by literature review (see Table 1). In cases involving new species, where more than two specimens were available for study, at least two individuals were dissected for full anatomical study to determine intraspecific variation. In instances where only two individuals were available for study, one was fully dissected and the second was examined for external anatomy, thereby retaining an intact holotype. In cases where only a single individual was available, the specimen was fully dissected and the parts preserved as a dissected holotype. In a few instances, such as in *Dermatobranchus rodmani*, *Dermatobranchus semilunus*, and *Dermatobranchus tuberculatus*, the holotype was also dissected, with the radula removed. This was carried out to ensure that these individuals were conspecific with other material in instances where cryptic species were difficult to identify from external morphology alone. In the Material examined section, all specimens indicated as dissected were dissected during the present study. Special attention was given to the reproductive anatomy, as some of these features were infrequently or cursorily described in the literature. In order to see the ampulla and bursa copulatrix it is necessary to dissect the nidamental glands as these structures are situated inside the folds of the female gland mass.

**Table 1:** Sources of morphological data for described species studied

Species	Distribution	Literature	Additional material examined
Outgroup			
*Berthella canariensis*Cervera *et al.*, 2000	Canary Islands	Cervera *et al.*, 2000	
Doridina			
*Actinocyclus verrucosus* Ehrenberg, 1831	Red Sea, Australia, Hawaii	Valdés, 2002	
*Bathydoris hodgsoni*Eliot, 1907	Weddell Sea	Wägele, 1989	
*Calycidoris guentheri*Abraham, 1876	Alaskan Arctic	Abraham, 1876; Fahey & Valdés, 2005	
*Diaphorodoris luteocincta*Sars, 1870	Mediterranean, north and east Atlantic	Portmann & Sandmeier, 1960; Schmekel & Portmann, 1982; Fahey & Valdés, 2005	
Dendronotina Tritoniidae			
*Marionia echinomuriceae*Jensen, 1994	Hong Kong	Jensen, 1994	
*Tritonia bollandi*Smith & Gosliner, 2003	Okinawa, Indonesia	Smith & Gosliner, 2003	
*Tritonia hombergi*Cuvier, 1803	North-east Europe	Odhner, 1922; Pruvot-Fol, 1937, 1954; Thompson, 1961; Thompson & Brown, 1984; Gosliner & Ghiselin, 1987	
Arminina Dironidae			
*Dirona picta*MacFarland in Cockerell & Eliot, 1905	West coast of North America	Cockerell & Eliot, 1905; Marcus, 1961; MacFarland, 1912, 1966	CASIZ 144725
Arminina Heroidae			
*Hero formosa*Lovén, 1841	Scotland	Bergh, 1888; Thompson & Brown, 1984	
Arminina Heterdorididae			
*Heterodoris antipodes*Willan, 1981	New Zealand	Willan, 1981	
*Heterodoris robusta* Verrill & Emerton, 1882	North Atlantic	Verrill & Emerton in Verrill, 1882; Odhner, 1926; Bouchet, 1977; Willan, 1981	
Arminina Madrellidae			
*Madrella ferruginosa* (Angas, 1864)	Eastern Mediterranean Okinawa, South-east Australia, Madagascar	Alder & Hancock, 1864; Angas, 1864; Thompson *et al.*, 1990	CASIZ 115754 CASIZ 115806
Arminina Pinufiidae			
*Pinufius rebus* Marcus & Marcus, 1960	Western Pacific, Maldives	Marcus & Marcus, 1960a; Rudman, 1981a	
Arminina Proctonotidae			
*Bonisa nakaza*Gosliner, 1981	West and east South Africa	Gosliner, 1981	
*Caldukia affinis* (Burn, 1958)	South-east Australia	Burn, 1958; Burn & Miller, 1969	CASIZ 071914
*Caldukia albolineata* Miller, 1969	New Zealand	Miller & Willan, 1986; Miller, 1970	
*Caldukia rubiginosa* Miller, 1969	New Zealand	Miller, 1970	
*Galeojanolus ionnae*Miller, 1971	New Zealand	Miller, 1971	
*Janolus australis*Bergh, 1884	Arafura Sea	Bergh, 1884; Gosliner, 1981	
*Janolus capensis*Bergh, 1907	West and east South Africa	Gosliner, 1981	
*Janolus hyalinus* (Alder & Hancock, 1854)	Australia, New Zealand, Europe, Mediterranean	Alder & Hancock, 1854, 1855; Bergh, 1888; Eliot, 1906; Schmekel, 1970; Gosliner, 1981; Schmekel & Portmann, 1982; Thompson & Brown, 1984; Miller & Willan, 1986	
*Janolus longidentatus*Gosliner, 1981	East coast of South Africa	Gosliner, 1981	
Arminina Arminidae			
*Armina aoteana*Miller & Willan, 1986	New Zealand	Miller & Willan, 1986	
*Armina* cf. *babai* (Tchang-Si, 1934)	Hong Kong	Baba, 1949; Jensen, 1997	
*Armina bayeri*Marcus & Marcus, 1966	West Africa	Marcus & Marcus, 1966	
*Armina californica* (Cooper, 1863)	Gulf of Panama, Mexico, western USA	Cooper, 1863; Marcus, 1961	CASIZ 171955
*Armina columbiana* O'Donoghue, 1924	Oregon, British Columbia, to Panama	O'Donoghue, 1921; Marcus, 1961	
*Armina comta* (Bergh, 1880)	Hong Kong	Jensen, 1997	
*Armina cordellensis*Gosliner & Behrens, 1996	Cordell Bank, California	Gosliner & Behrens, 1996	CASIZ 105717
*Armina elongata*Ardila & Valdés, 2004	Columbia	Ardila & Valdés, 2004	
*Armina gilchristi* (Bergh, 1907)	South Africa	Bergh, 1907; Marcus & Marcus, 1966; Gosliner, 1987	CASIZ 087273 CASIZ 087388
*Armina joia*Marcus & Marcus, 1966	West Africa	Marcus & Marcus, 1966	
*Armina juliana*Ardila & Diaz, 2002	Colombia	Ardila & Diaz, 2002; Ardila & Valdés, 2004	
*Armina loveni* (Bergh, 1860)	Norway, Britain, Denmark	Pruvot-Fol, 1954; Marcus & Marcus, 1966; Kolb, 1998; Ardila & Diaz, 2002	CASIZ 074516
*Armina maculata* Rafinesque, 1814	Mediterranean, Portugal, Angola	Pruvot-Fol, 1937, 1954; Marcus & Marcus, 1966; Schmekel & Portmann, 1982; Kolb, 1998	
*Armina magna*Baba, 1955	Japan, Philippines	Baba, 1955	CASIZ 173357 CASIZ 174127
*Armina mülleri* (Ihering, 1886)	Brazil	Marcus & Marcus, 1966; Marcus & Marcus, 1960b, 1967; Ardila & Diaz, 2002; Ardila & Valdés, 2004	
*Armina neapolitana* (Delle Chiaje, 1824)	Mediterranean	Pruvot-Fol, 1937, 1954; Marcus & Marcus, 1966; Kolb, 1998	CASIZ 068478
*Armina papillata*Baba, 1933	Hong Kong	Baba, 1933; Baba, 1955; Jensen, 1997	
*Armina paucifoliata*Baba, 1955	Japan Philippines		CASIZ 171415 CASIZ 174128
*Armina punctulata* Lin, 1990	Hong Kong	Jensen, 1997	
*Armina semperi* (Bergh, 1861)	Sagami Bay, Singapore, Philippines	Baba, 1955	
*Armina tigrina* Rafinesque, 1814	Mediterranean, Portugal, Senegal, Sargasso, Gulf of Mexico	Pruvot-Fol, 1937, 1954; Marcus & Marcus, 1966; Thompson *et al*., 1990; Kolb, 1998; Ardila & Diaz, 2002	CASIZ 87181
*Armina variolosa* (Bergh, 1904)	Hong Kong	Bergh, 1904; Baba, 1949; Jensen, 1997	
*Armina xandra*Marcus & Marcus, 1966	West Africa	Marcus & Marcus, 1966	
*Dermatobranchus albus* (Eliot, 1904)	Okinawa	Eliot, 1904; Edmunds & Thompson, 1972	CASIZ 087896 ASIZ 105263
*Dermatobranchus fortunatus* (Bergh, 1888)	Eastern Australia, Okinawa, Kerama Island	Bergh, 1888; Marshall & Willan, 1999	CASIZ 065771
*Dermatobranchus gonatophora* (Hasselt, 1824)	Indonesia, Okinawa, South Africa	Bergh, 1887, 1905; Gosliner, Behrens & Williams, 1996	CASIZ 105299
*Dermatobranchus marginlatus*Lin, 1981	China	Lin, 1981	
*Dermatobranchus multistriatus*Lin, 1981	China	Lin, 1981	
*Dermatobranchus ornatus* (Bergh, 1874)	Sagami Bay, Okinawa, Thailand	Baba, 1949; 1976;	CASIZ 159387 CASIZ 144033 CASIZ 144008 CASIZ 156334
*Dermatobranchus otome*Baba, 1992	Sagami Bay	Baba, 1992	CASIZ 082031 CASIZ 082033 CASIZ 082065
*Dermatobranchus primus*Baba, 1976	Sagami Bay	Baba, 1976	
*Dermatobranchus pulcherrimus*Miller & Willan, 1986	New Zealand	Miller & Willan, 1986	CASIZ 105696
*Dermatobranchus pustulosus*Hasselt, 1824	Indonesia, Philippines	Hasselt, 1824	CASIZ 069306 CASIZ 157212 CASIZ 083865 CASIZ 085963 CASIZ 096293 CASIZ 083798 CASIZ 085904
*Dermatobranchus rubidus* (Gould, 1852)	Hawaii	Gould, 1852; Kay, 1979	CASIZ 116909
*Dermatobranchus semistriatus*Baba, 1949	Sagami Bay,	Baba, 1949, 1976	CASIZ 104706
*Dermatobranchus striatus* (Hasselt, 1824)	Sagami Bay, Indonesia	Baba, 1949, 1976, 1992; Bergh, 1905	CASIZ 170101 CASIZ 086314
*Dermatobranchus tongshanensis*Lin, 1981	China	Lin, 1981	
*Histiomena convolvula* (Lance, 1962)	Gulf of California to Panama	Mörch, 1860; Lance, 1962	CASIZ 074274 CASIZ 020300

### Phylogenetic analysis

Fifty-five species of Arminidae were considered for the present analyses. All of these species were included in the final analysis. Six additional species of *Dermatobranchus* were excluded from the analysis owing to lack of sufficient morphological information. Forty-three morphological characters were considered for the present study and all characters were included in the final analysis. Table 1 contains a list of sources of material for previously described species included in the phylogenetic analysis. The character matrix is shown in Table 2. Phylogenetic analyses were performed using the program PAUP v. 4.0 (Swofford, 2002) using the heuristic algorithm (tree bisection-reconnection branch swapping option), set at maximum parsimony. One hundred replicates were run with starting trees obtained using stepwise addition. Characters were unordered and were polarized using the following outgroup species: *Actinocylus verrucosus* Ehrenberg, 1831, *Bathydoris hodgsoni* Eliot, 1907, *Berthella canariensis* Cervera *et al.*, 2000, based on Valdés' (2002) and Fahey & Valdés' (2005) analyses of the Onchidorididae, *Bonisa nakaza* Gosliner, 1981, *Caldukia affinis* (Burn, 1958), *Caldukia albolineata* Miller, 1970, *Caldukia rubiginosa* Miller, 1970, *Calycidoris guentheri* Abraham, 1876 based on Millen & Martynov's (2005), Valdés' (2002), and Fahey & Valdés' (2005) analyses of the Onchidorididae, *Diaphorodoris luteocincta* (Sars, 1870) based on the analysis of the Goniodorididae by Gosliner (2004), Valdés' (2002) and Fahey & Valdés' (2005) analyses of the Onchidoridae, *Dirona picta* MacFarland in Cockerell & Eliot, 1905, *Galeojanolus ionnae* Miller, 1971, *Hero formosa* Lovén, 1841, *Heterodoris antipodes* Willan, 1981, *Heterodoris robustus* Verrill, 1882, *Janolus australis* Bergh, 1884, *Janolus capensis* Bergh, 1907, *Janolus hyalinus* (Alder & Hancock, 1864), *Janolus longidentatus* Gosliner, 1981, *Madrella sanguinea* (Angas, 1864), *Marionia echinomuriceae* Jensen, 1994, *Pinufius rebus* Marcus & Marcus, 1960a, *Tritonia bollandi* Smith & Gosliner, 2003, and *Tritonia hombergi* Cuvier, 1803. In addition to the outgroup taxon, these taxa include four species of Doridina, three species of Dendronotina and 15 outgroup members of the Arminina not traditionally included in the Arminidae. All three Dendronotina were members of the Tritoniidae as members of this family have been traditionally considered as the most basal Dendronotina (Odhner, 1939).

**Table 2:** Data matrix of character states in the taxa examined for the phylogenetic analysis of Arminidae

	1	10	20	30	35	40	43
*Berthella canariensis*	000-000200021011000-----00011101----2010000						
*Actinocyclus verrucosus*	010-0100-0000000000----111000000----2011000						
*Bathydoris hodgsoni*	010-0100-10000000010-110000111001---0--0000						
*Calycidoris guentheri*	010-0100-0000000010-----0001111-----2010000						
*Diaphorodoris luteocincta*	010-0100-0000010010-----1001--1-----2010000						
*Marionia echinomuriceae*	1-1-00121002101100102110010111001---21?0000						
*Tritonia bollandi*	1-1-001210021000001101100?0???0000??2111000						
*Tritonia hombergi*	1-1-001210021000001111100?0???00?0??2?????0						
*Dirona picta*	1-1-0012000000000110-2101001--001---2000010						
*Hero formosa*	1-1-0011-0020??0011102101?0???0002????????0						
*Heterodoris antipodes*	1-100102000010000110-2000?0???001---2100000						
*Heterodoris robusta*	1-000102000010000111??00??0???001---2010000						
*Madrella sanguinea*	1-0-01120002100101111210110---001---???1000						
*Pinufius rebus*	1-0-00120002000001110210------00020?0001000						
*Bonisa nakaza*	1-0-0011-00000100111020010000-001---1011010						
*Caldukia affinis*	1-0-0011-00000000110-110000000000---?111010						
*Caldukia albolineata*	1-0-0011-0000000011101001000000002--?101000						
*Caldukia rubiginosa*	1-0-0011-0000000011101001000000002--?101010						
*Galeojanolus ionnae*	1-0-0011-1000010011101001000000002--1000100						
*Janolus australis*	1-0-0100-00000100110-200000111001---10?01?0						
*Janolus capensis*	1-0-0011-00000000110-000000111001---1010100						
*Janolus hyalinus*	1-0-0011-00000100110-000100111001---1000100						
*Janolus longidentatus*	1-0-0011-0000010011110000001110002211001100						
*Armina aoteana*	1-10100200111001101101101000000000000000000						
*Armina bayeri*	1-00100200111011101100101001110000000?????0						
*Armina californica*	1-00100200111001101101100000010000000010010						
*Armina comta*	1-10100201?11011101112101001110000000001000						
*Armina cordellensis*	1-100102?0?11001101112101000110000000001000						
*Armina elongata*	1-10100210111001101112101000000001000001000						
*Armina gilchristi*	1-10100200011001101101101?0???0001?00?????0						
*Armina joia*	1-10100200111011101111101000000000000?????0						
*Armina juliana*	1-10100200111001101112101000000000000111010						
*Armina loveni*	1-10100200111001101101101000000001000010000						
*Armina maculata*	1-10010200011001101101101001110000000010000						
*Armina magna*	1-10100200011001111101101000010000000000000						
*Armina mulleri*	1-10100200111011101101101000010001000110010						
*Armina neapolitana*	1-10100200111001101112101000000000000010000						
*Armina papillata*	1-10100210111001101112101000000000000001000						
*Armina paucifoliata*	1-10100200011001101101101000010000000010000						
*Armina punctulata*	1-10010200111001101112101000000000000000000						
*Armina variolosa*	1-10010210111101101111101000110000000001000						
*Armina xandra*	1-00010200111011101112101100110000000?????0						
*Dermatobranchus albopunctulatus*	1-1110020001100000111200100011001---0??????						
*Dermatobranchus albus*	1-21100200011000011112101211110000100001000						
*Dermatobranchus fortunatus*	1-21010200011000021111101210000002120011000						
*Dermatobranchus gonatophorus*	1-1110020001100000111110100000001---0001000						
*Dermatobranchus marginlatus*	1-1110020001100000111200100001000000???????						
*Dermatobranchus multistriatus*	1-1110020001100000111201100011001---???????						
*Dermatobranchus nigropunctatus*	1-1110020001100000111201100111001---0??????						
*Dermatobranchus ornatus*	1-1101020001100000111111100111001---0001001						
*Dermatobranchus otome*	1-21100200011000011112101211110000100011000						
*Dermatobranchus pulcherrimus*	1-1110020001110000111200100000000000000000?						
*Dermatobranchus pustulosus*	1-11100200011000001102001000010001100011000						
*Dermatobranchus rubidus*	1-1110020001110000111200100000000000000100?						
*Dermatobranchus semistriatus*	1-11100200011000001112101100110002100??????						
*Dermatobranchus striatus*	1-2110020001100001111210121111000010001100?						
*Dermatobranchus tongashanensis*	1-1110020001100000111201100011001---???????						
*Dermatobranchus albineus*	1-11100200011000021111101210110000120001001						
*Dermatobranchus arminus*	1-11100200011000101111101000010001000001000						
*Dermatobranchus caeruleomaculatus*	1-1110020001100000111201&00111001---0001000						
*Dermatobranchus caesitius*	1-11100200011000011112101210000000100001000						
*Dermatobranchus cymatilis*	1-21100200011000001102001001110001110011000						
*Dermatobranchus dendronephthyphagus*	1-1110020001100000102201000111001---0011001						
*Dermatobranchus diagonalis*	1-21100200011000011112101211110000200001000						
*Dermatobranchus earlei*	1-21100200011000021111101210010002100001000						
*Dermatobranchus fasciatus*	1-1110020001100000111&101000000000100011000						
*Dermatobranchus funiculus*	1-21100200011000021112101210010001000001000						
*Dermatobranchus kalyptos*	1-11010200011000001111101000010000100000000						
*Dermatobranchus kokonas*	1-21000200011000021112101210010002120001000						
*Dermatobranchus leoni*	1-1110020001100000111201100011001---0011010						
*Dermatobranchus microphallus*	1-21100200011000021111101210010002200001000						
*Dermatobranchus oculus*	1-21100200011000011112101211100000100001000						
*Dermatobranchus phyllodes*	1-11100200011000001112000001110001000001010						
*Dermatobranchus piperoides*	1-21000200011000021111101210110001120001010						
*Dermatobranchus semilunus*	1-21100200011000001112001001110001100001001						
*Dermatobranchus rodmani*	1-21000200011000021111101210010000120001000						
*Dermatobranchus tuberculatus*	1-21010200011000011112101100010000000001001						
*Histiomena marginata*	1-0010021001100110111100000111001---00?0000						

Data codes: 0, generally the presumed plesiomorphic condition; 1–3, apomorphic conditions or in some specific instances cited may represent the presumed plesiomorphic state; -, character not applicable; ?, missing data.

Synapomorphies were mapped using the character trace option in MacClade 4.08 (Maddison & Maddison, 2005) using the majority rule tree from the PAUP analysis.

Bremer analyses were performed on the strict consensus tree to estimate branch support (Bremer, 1994).

## RESULTS

### Taxonomic section

### Species descriptions

### Family Madrellidae Vayssière, 1909

### Genus*Madrella* Alder & Hancock, 1864

#### 

##### 

###### Type species

*Madrella ferruginosa* Alder & Hancock, 1864, by original designation.

### *Madrella ferruginosa* Alder & Hancock, 1864

#### 

##### 

###### Material examined

CASIZ 115806, two specimens, 10–12 mm alive, one dissected, Tengan Pier, Ishikawa City, Okinawa, Japan, collected 25.vi.1995 by R. Bolland (3218-D), 12 m depth. CASIZ 121200, one specimen, 5 mm preserved, dissected, Bigej-Mack Reef, Kwajalein Atoll, J. Johnson, 10.x.1995, 35 m depth. CASIZ 099104, seven specimens, 3–9 mm alive, Tengan Pier, Ishikawa City, Okinawa, Japan, collected 5.iii.1994 by R. Bolland (3218), 12 m depth.

###### Geographical distribution

This species is known from India (Alder & Hancock, 1864), Japan (Baba, 1949, as *M. sanguinea*; present study), South Africa (Fraser, 2001), the Marshall Islands (present study), and Madagascar (present study).

###### Buccal armature

The jaws are large and thickly cuticularized with a thick masticatory margin and two to three rows of long, pointed denticles. The radular formula of CASIZ 121200 is 48 × 1.1.1.1.1. The rachidian teeth are claw-shaped with a thick, projecting, pointed central cusp that has six pointed, flanking denticles on each side. The next lateral tooth is also claw-shaped with ten shorter, pointed denticles that decrease in size towards the rachidian tooth. The next lateral tooth is a long pointed hook with no denticles.

### Family Dironidae Eliot, 1910

### Genus*Dirona* MacFarland in Cockerell & Eliot, 1910

#### 

##### 

###### Type species

*Dirona picta* MacFarland in Cockerell & Eliot, 1905, by original designation.

### *Dirona picta* MacFarland in Cockerell & Eliot, 1905

#### 

##### 

###### Material examined

CASIZ 144725, four specimens, San Francisco Bay, California, collected 16.ix.1975 by K. Mauzey, 5 m depth.

### Family Arminidae Rafinesque 1814

### Genus*Armina* Rafinesque, 1814

#### 

##### 

###### Type species

*Armina tigrina* Rafinesque, 1814, by subsequent designation by Iredale & O'Donoghue (1923).

### Previously described species examined and included in the phylogenetic analysis

### *Armina californica* (Cooper, 1863)

*Pleurophyllidia californica* Cooper, 1863: 203.

*Armina californica* (Cooper, 1863) Marcus, 1961: 41, pl. 8, figures 147–150.

*Armina columbiana* O'Donoghue, 1924: 11, pl. 2, figures 13–17. Marcus, 1961: 43, pl. 8, figures 151–154. Steinberg, 1963; 64

*Armina vancouverensis* (Bergh, 1876) Steinberg, 1963: 64.

*Armina digueti* Pruvot-Fol, 1956: 464, figures 8–10. Steinberg, 1963: 64.

#### 

##### 

###### Material examined

CASIZ 171955, one specimen, 50 mm, dissected, Gulf of Alaska, 223 m depth, collected 22.vi.2001 by K. Palenscar.

###### Geographical distribution

This species is known from the west coast of America, from the Gulf of Alaska to Panama.

###### External morphology

The external morphology matches the description by Cooper (1863). That is, the body shape is ovate, rounded in front with approximately 15 elevated, parallel notal ridges.

###### Buccal armature

The jaws are large and thickly cuticularized, with a thick masticatory margin and multiple rows of triangular, pointed denticles. The radular formula is 38 × 58.1.1.1.58. The rachidian teeth are broadly triangular in shape, with five large, pointed denticles lengthening towards the sixth, central denticle. The first lateral tooth is bicuspid. The next 58 teeth are elongate hooks having narrow, feathery denticles near the tip. The last three to four of these are smaller than the others.

###### Reproductive system

As described and drawn by Marcus (1961) for *A.columbiana*.

###### Remarks

The ridges or ‘stripes’ that Cooper described do not interconnect at regular intervals on our specimens. O'Donoghue (1924) stated that the arrangement of the notal ridges is a distinguishing feature of *A. californica.* He thought that the ridges ‘start at middle, passing outwards and backwards at an acute angle with the mid-dorsal line’. However, the dorsal ridges of the specimens we examined lie parallel to the midline and mantle edge. Marcus (1961) thought that, because Bergh in his original description (1890) of *A. californica* did not mention oblique dorsal ridges, then this was not a distinguishing feature of this species. Marcus thought that O'Donoghue actually examined specimens of *A. columbiana.* The specimen we examined from the Gulf of Alaska matches the descriptions by Cooper (1863) and Marcus (1961) except for one notable difference. The Alaskan specimen has none of the ‘claw-like’ type of radular teeth that Marcus noted. The ‘brush-like’ lateral teeth are present, however. Marcus (1961) thought that the specimens examined by Bergh (1904) were actually *A. columbiana* O'Donoghue, 1924 because of the predominance of brush-like denticles. The presence of brush-like or claw-like denticles varies within this species and *A. columbiana* should remain as a synonym of *A. californica*.

### *Armina cordellensis* Gosliner & Behrens, 1996

#### 

##### 

###### Material examined

Holotype: CASIZ 105717, Cordell Bank, California, collected 20.x.1978, by R. Schmeider *et al.* 23–26 fathoms (fm) depth.

###### Geographical distribution

This species is known only from the Cordell Bank, California, USA (Gosliner & Behrens, 1996).

### *Armina magna* Baba, 1955 (Figs 1A, 2, 3)

*Armina magna* Baba, 1955:22, text figures 31, 32, pl. 11, figures 29, 30.

**Figure 1:**
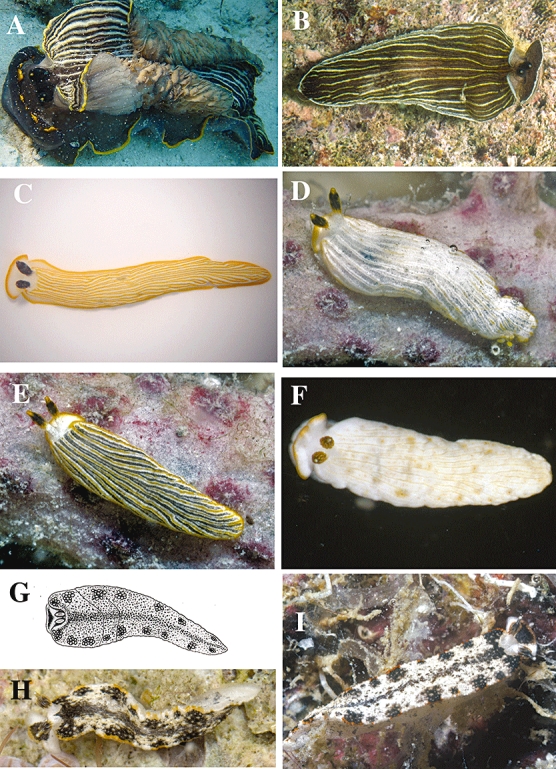
Living animals. A, *Armina magna* Baba, 1955, CASIZ 173357, Panglao, Philippines, photo by Marina Poddubetskaia. B, *Armina paucifoliata* Baba, 1955, CASIZ 171415, Panglao, Philippines, photo by T. Gosliner. C, *Dermatobranchus albus* (Eliot, 1904), Panglao, Philippines, photo by Marina Poddubetskaia. D, E, *Dermatobranchus albus* (Eliot, 1904), CASIZ 174129, Tulear, Madagascar, photos by T. Gosliner. F, *Dermatobranchus albus* (Eliot, 1904), CASIZ 068678, Madang, Papua New Guinea, photo by T. Gosliner. G, *Dermatobranchus fortunatus* (Bergh, 1888), after Bergh, 1888. H, *Dermatobranchus fortunatus* (Bergh, 1888), CASIZ 174198, Palmyra Atoll, photo by T. Gosliner. I, *Dermatobranchus fortunatus* (Bergh, 1888), CASIZ 074158, Mahe, Seychelles, photo by T. Gosliner.

#### 

##### 

###### Material examined

CASIZ 173357, one specimen dissected, 60 mm preserved, station (stn) T39, muddy sand 100–138 m, Cervera Shoal, west of Pamilacan Island, Bohol, Philippines, collected 6.vii.2004 by T. Gosliner, Y. Camacho, J. Templado, M. Malaquias, M. Poddubetskaia. CASIZ 174127, OT 607, one specimen, 130 mm alive, off San Isidro, Panglao Island Philippines, 9°33.4′N, 123°49.6′E–9°33.8′N, 123°51.5′E, mud and fine sand, stn T10, 117–124 m depth, collected 15.vi.2004 by T. Gosliner, Y. Camacho, J. Templado, M. Malaquias, M. Poddubetskaia.

###### Geographical distribution

This species is known only from Sagami Bay, Japan (Baba, 1955) and Panglao, Bohol Island, and Cervera Shoal, Philippine Islands (present study).

###### External morphology

The body shape of the living animal (Fig. 1A) is broad, flattened, and narrows at the posterior end. The wide foot projects beyond the distinct mantle margin and has a large ovoid pedal gland on its posteroventral surface in the larger specimen examined. The pedal gland is absent in the smaller specimen. There are up to 22 prominent longitudinal dorsal ridges with some additional shorter ridges in between, over the surface of the notum. The blunt oral veil extends forward and is rounded at the sides. Behind the oral veil are the closely spaced rhinophores. The rhinophores have a series of longitudinal lamellae on the rounded club. The stalk widens as it enters the dorsal cavity and there are no lamellae on the stalk. A caruncle is situated just posterior to the rhinophores. Marginal sacs are not visible along the mantle edge. Under the mantle there are 36–56 branchial lamellae at the anterior end of the body and at least 52–60 hyponotal lamellae at the posterior end. The genital opening is situated in the anterior third of the body wall and the anus opening is approximately half way along the body. The ground colour of the dorsum and foot is black and the dorsal ridge crests are pale yellow to opaque white. The mantle edge is bright yellow. The rhinophore lamellae are black and the stalk has black stripes perpendicular to the lamellae. The anterior hyponotal lamellae have dark spots and the hyponotal respiratory leaves have dark edges. The ventral surface of the oral veil, the foot sides, and the foot sole has dark mottled blotches and the anterior foot edge is white.

###### Buccal armature

**Figure 2:**
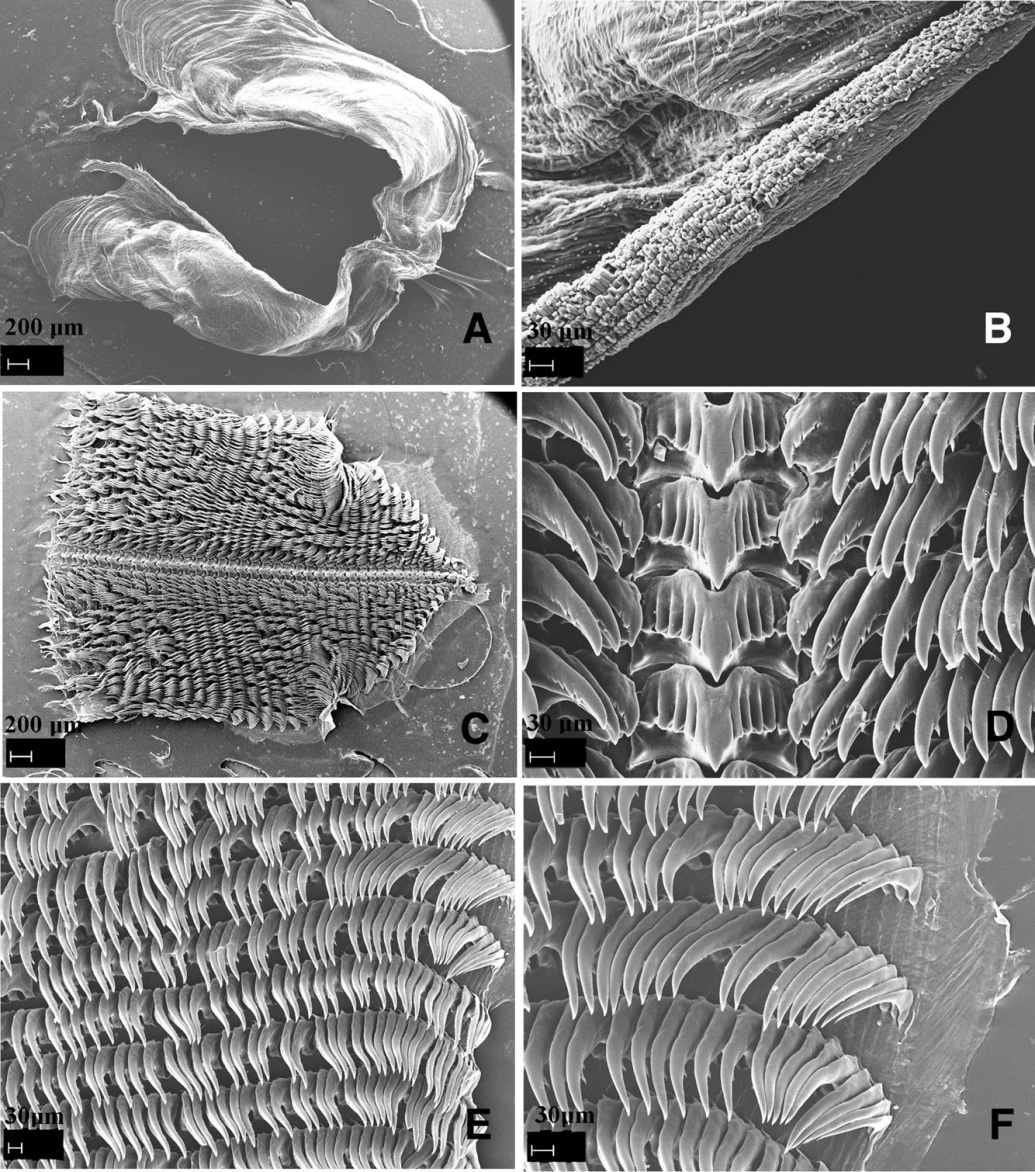
*Armina magna* Baba, 1955. Buccal armature, CASIZ 173357. A, jaws; B, masticatory margin; C, whole radula; D, central portion of radula; E, middle lateral teeth; F, outer lateral teeth.

The jaws are large and thickly cuticularized (Fig. 2A), with a thick masticatory margin and approximately seven rows of pointed denticles along the entire margin of each jaw (Fig. 2B). The radular formula (CASIZ 173357) is 40 × 8.36.1.1.1.36.8 (Fig. 2C). The rachidian teeth (Fig. 2D) are broad with a large, spear-shaped central cusp and four to five flanking denticles on each side. The flanking denticles are all of the same thickness. The inner lateral teeth have a narrow base and a cusp that is posteriorly directed. The cusp has three to four triangular denticles and two to three smaller secondary denticles along its outer side. The next 36 lateral teeth (Fig. 2E) are hook-shaped with up to three pointed denticles on the under side. The number of denticles decreases to one denticle on the teeth towards the outer edge of the radula. The outer approximately eight lateral teeth (Fig. 2F) are also hook-shaped, with no denticles and the outermost tooth is smaller than the other teeth.

###### Reproductive system

**Figure 3:**
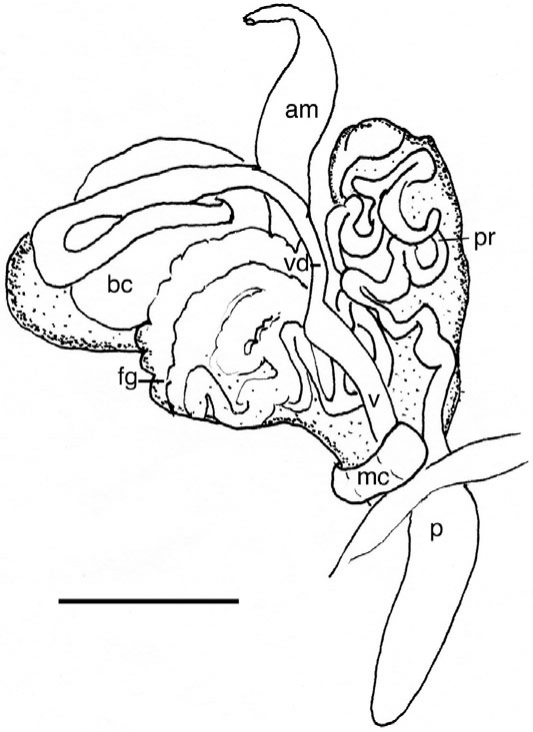
*Armina magna* Baba, 1955. Reproductive system, CASIZ 173357. am, ampulla; bc, bursa copulatrix; fg, female gland mass; mc, muscular collar; p, penis; pr, prostate; v, vagina; vd, vaginal duct. Scale bar = 1.17 mm.

The reproductive organ arrangement is androdiaulic. The long hermaphroditic duct leads from the ovotestis into the tubular ampulla (Fig. 3). The ampulla bifurcates near the distal end of the female gland mass into the well-defined albumen gland and into the very long, multi-coiled prostate. The prostate expands into the long, bulbous penial sheath. From the large, ovoid bursa copulatrix the very long, narrow vaginal duct emerges and continues to a wider vagina that exits adjacent to the penial opening. At the distal end of the vagina is a muscular collar.

###### Remarks

*Armina magna* is known only from the original description (Baba, 1955) from Japan. The external morphology, radula, and jaws were described but the reproductive system was not. One of its most distinctive features, in addition to its colour pattern, is the large ovoid pedal gland that is also present in the larger specimen of the Philippine material examined here. The incomplete orange margin of the oral veil is consistent between Baba's and the present material. The rachidian teeth of the Japanese material had six to seven denticles that extended well onto the median cusp, whereas the present material has five to six denticles that do not extend on to the cusp. The other radular teeth are very similar in their denticulation in specimens from both localities. The differences between Baba's and the present material are relatively minor, and there is little doubt that they are conspecific.

Externally, *A. magna* closely resembles *A. californica* (Cooper, 1863) and *Armina paucifoliata* Baba, 1955. All of these species have a very dark background colour and light coloured dorsal ridge crests and mantle margin. The dorsal ridges of *A. californica* are white, whereas those of *A. magna* are white to yellow and the ridges of *A. paucifoliata* are light yellow. The white mantle edge of *A. californica* is much wider with more solid pigmentation than the narrow, uneven white pigment along the edge of *A. paucifoliata*. *Armina magna* has a yellow to orange mantle edge.

For further morphological details of *A. californica* see the Remarks section of *A. paucifoliata*.

The oral veil of *A. magna* and *A. paucifoliata* differs (Fig. 1A, B). That of *A. magna* has blunt, rounded front corners, whereas the veil of *A. paucifoliata* is anvil-shaped and pointed. The caruncle of *A. magna* is wider than that of *A. paucifoliata*. Furthermore, *A. magna* has an ovoid pedal gland whereas *A. paucifoliata* has a linear one.

**Figure 4:**
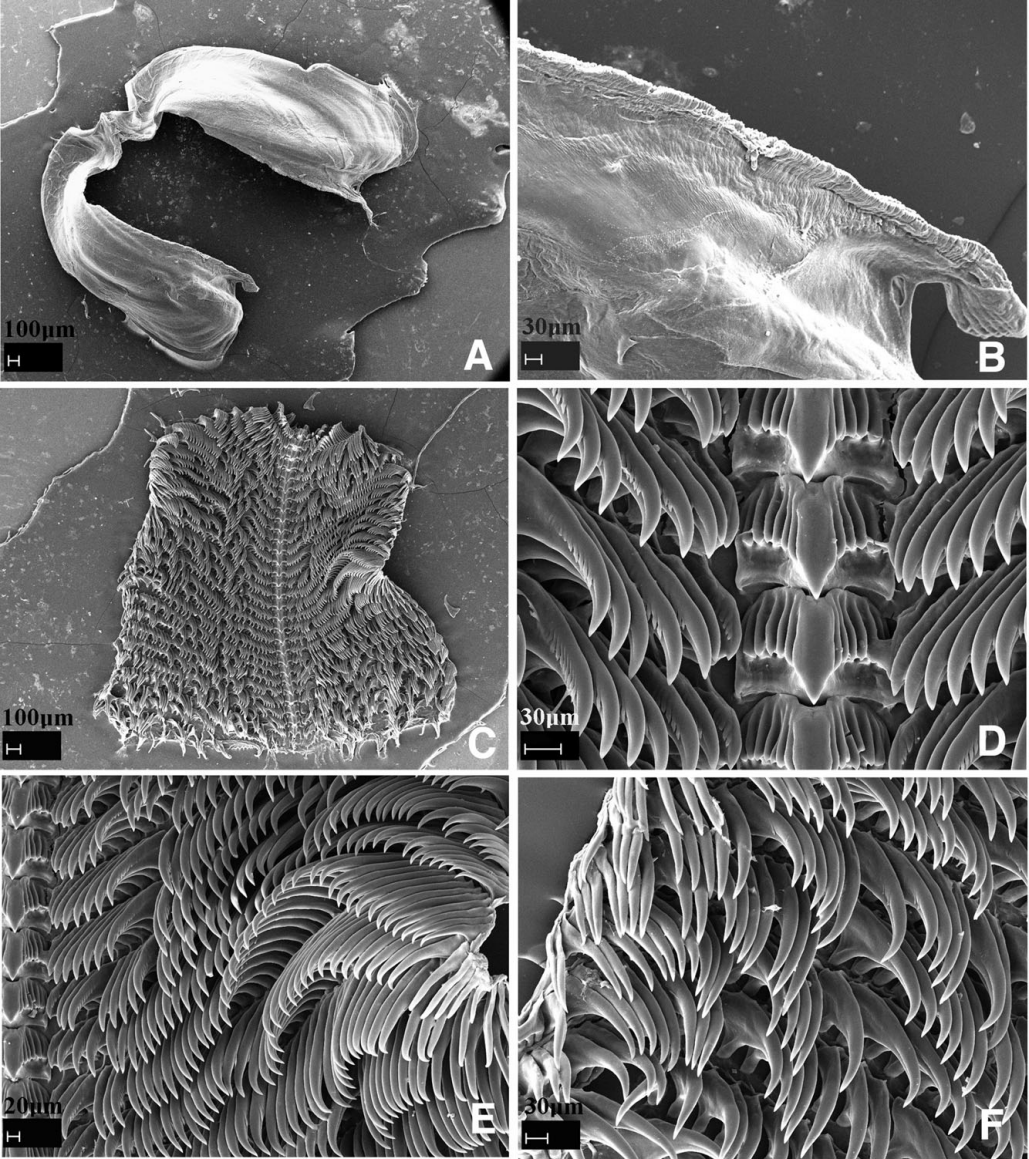
*Armina paucifoliata* Baba, 1955. Buccal armature, CASIZ 171415. A, jaws; B, masticatory margin; C, whole radula; D, central portion of radula; E, middle lateral teeth; F, outer lateral teeth.

The radular morphology differs between these species. The lateral teeth of *A. magna* (Fig. 2D) have long, pointed denticles that protrude from under each hook-shaped tooth. In contrast, the denticles of *A. paucifoliata* (Fig. 4D) are shorter and are attached at the outer sides of each lateral tooth. There are up to 15 smooth outer lateral teeth in *A. paucifoliata* and up to eight smooth teeth in *A*. *magna*.

**Figure 5:**
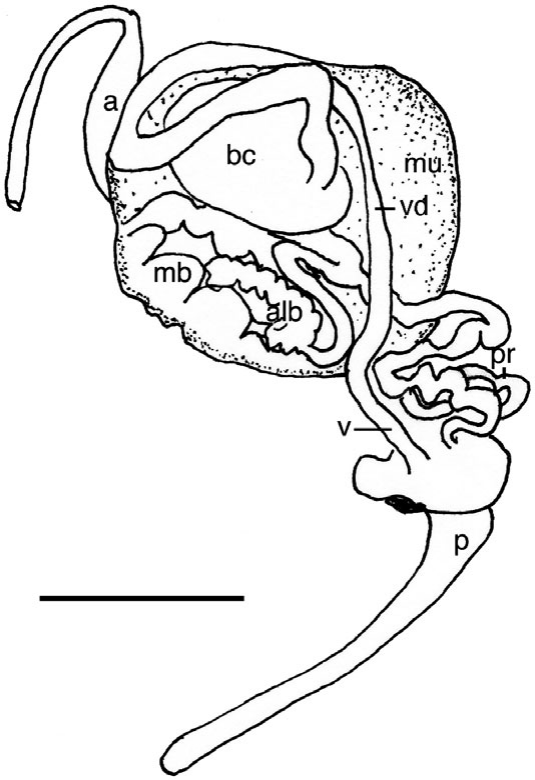
*Armina paucifoliata* Baba, 1955. Reproductive system, CASIZ 171415. a, ampulla; alb, albumen gland; bc, bursa copulatrix; mb, membrane gland; mu, mucous gland; p, penis; pr, prostate; v, vagina; vd, vaginal duct. Scale bar = 2.0 mm.

The major reproductive system difference between these two species is the presence of the muscular collar on the vagina of *A. magna*. *Armina magna* (Fig. 3) also has a shorter, wider penis than the elongate slender penis of *A. paucifoliata* (Fig. 5).

### *Armina paucifoliata* Baba, 1955 (Figs 1B, 4, 5)

*Armina paucifoliata* Baba, 1955: 23, text figures 32–33, pl. 11 figure 31.

#### 

##### 

###### Material examined

CASIZ 171415, two specimens, one dissected, 33 mm, 50 mm alive, between Panglao and Pamilacan Islands, 9°33.4′N, 123°51.0′E, fine sand and mud with echinoderms, stn T27, 106–137 m depth, collected 25.vi.2004 by T. Gosliner, Y. Camacho, J. Templado, M. Malaquias, M. Poddubetskaia. CASIZ 174128, OT 607, three specimens 55–90 mm alive, off San Isidro, Panglao Island Philippines, 9°33.4′N, 123°49.6′E–9°33.8′N, 123°51.5′E, mud and fine sand, stn T10 117–124 m depth, collected 15.vi.2004 by T. Gosliner, Y. Camacho, J. Templado, M. Malaquias, M. Poddubetskaia.

###### Geographical distribution

This species is known only from Japan (Baba, 1955) and Panglao, Philippine Islands (present study).

###### External morphology

The body shape of the living animal (Fig. 1B) is broad, flattened, and narrows at the posterior end. The wide foot projects beyond the distinct mantle margin. The posterioventral end of the foot of the larger specimens has a narrow elongate pedal gland that is one-quarter to one-third of the total body length. There are up to 15 prominent longitudinal dorsal ridges with some additional shorter ridges in between, on the dorsal surface of the notum. The distinct anvil-shaped oral veil extends well forward and is laterally pointed. Behind the oral veil are the closely spaced rhinophores. The rhinophores have a series of longitudinal lamellae on the rounded club. The stalk widens as it enters the dorsal cavity and there are no lamellae on the stalk. A caruncle is situated just posterior to the rhinophores. Marginal sacs are not visible along the mantle edge. Under the mantle there are at least 25–40 small, weakly formed branchial folds at the anterior end of the body and approximately 50 wavy rows of complete and incomplete hyponotal lamellae at the posterior end. The genital opening is situated in the anterior third of the body wall and the anus opening is approximately half way along the body.

The ground colour of the dorsum and foot is deep brown or black and the dorsal ridge crests are pale yellow to opaque white. The mantle edge is opaque white or pale yellow. The rhinophores are black. The ventral side of the oral veil and the foot sole has dark speckles and the edge of both is opaque white.

###### Buccal armature

The jaws are large and thickly cuticularized (Fig. 4A), with a thick masticatory margin and multiple rows of pointed denticles along the entire margin (Fig. 4B). The radular formula is 31 × 15.28.1.1.1.28.15. The rachidian teeth (Fig. 4C, D) are broad with a large, spear-shaped central cusp and five to seven flanking denticles on each side. The flanking denticles are all the same thickness. The narrow, posteriorly directed first lateral tooth has six blunt denticles and a broad base with a ‘beak’ on the inner side. The next 27 lateral teeth (Fig. 4E) are hook-shaped with up to nine denticles on the outer side that decrease in size and number towards the outer edge of the radula. The next 15 lateral teeth are also hook-shaped, with no denticles. The outer two teeth are smaller than the other teeth (Fig. 4F).

###### Reproductive system

The reproductive organ arrangement is androdiaulic. The long hermaphroditic duct leads from the ovotestis into the tubular ampulla (Fig. 5). The ampulla bifurcates near the distal end of the female gland mass into the well-defined albumen gland and into the very long, multi-coiled prostate. The prostate expands into the long penial sheath. The penis is thin and elongate and rounded at its apex. From the large, ovoid bursa copulatrix the narrow vaginal duct emerges and continues to a narrow vagina that exits adjacent to the penial opening.

###### Remarks

*Armina paucifoliata* was described from a single specimen collected from Sagami Bay, Japan. The external morphology, radula, and jaws were described, but the reproductive system was not. The colour pattern between the Japanese specimen and the present material is entirely consistent. In the figure of Baba's preserved animal (Baba, 1955: text fig. 32), the elongate pedal gland and the thin, elongate penis are visible and are consistent with the form in the present material. The rachidian teeth of the Japanese and present material have five to seven denticles that extend well onto the median cusp. The other radular teeth are very similar in their denticulation in specimens from both localities with the following exception: the material from the Philippines has up to nine denticles whereas the Japanese specimen based on the figure (Baba, 1955: text fig. 33B) appears to have fewer. The differences between Baba's specimen and the present material are relatively minor and there is little doubt that they are conspecific.

Externally, *A. paucifoliata* closely resembles *A. californica* (Cooper, 1863) and *A. magna*. All species have a very dark background colour and light coloured dorsal ridge crests and mantle margin. The white mantle edge of *A. californica* is much wider with more solid pigmentation than the narrow, uneven white pigment along the edge of *A. paucifoliata*. *Armina magna* has a yellow to orange mantle edge. For a complete comparison between *A. magna* and *A. paucifoliata*, see the Remarks section of *A. magna*.

#### Comparison between A. paucifoliata and A. californica

The oral veil is very different between these two species. The veil of *A. paucifoliata* is anvil-shaped and has a smooth margin. The veil of *A. californica* is rounded and has a scalloped edge. The pedal gland of *A. californica*, although elongate, is much shorter than that of *A. paucifoliata.* The anterior branchial folds are much more well-developed in *A*. *californica* than in *A. paucifoliata*.

The two species have similarities in their radular morphology. Both have a broad, denticulate rachidian tooth with a longer central cusp. Both have denticulate lateral teeth and some smooth outer teeth. The radular formula of *A. paucifoliata* is 31 × 1 5.28.1.28.15 but that of *A. californica* is 41 × 60.1.1.1.60. Marcus (1961) noted that only the outermost six lateral teeth are smooth in *A. californica* whereas the outer 15 lateral teeth are smooth in *A. paucifoliata*. In *A. californica*, the central cusp of the rachidian tooth is about as wide as the adjacent denticles and the denticles extend almost to the tip of the apex of the central cusp. In *A*. *paucifoliata*, the central cusp is much broader than the denticles and the cusp extends well beyond the denticles. The inner lateral tooth of *A. californica* is much shorter than that of *A*. *paucifoliata.* The denticles on the lateral teeth of *A. californica* are isolated to the tips of the teeth whereas the denticles on the lateral teeth of *A. paucifoliata* are numerous and are lined up on the outer side of each tooth.

The reproductive morphology of these two species has similarities. Both have a long, coiled prostate and an enlarged penial sheath, but *A. californica* has a much more bulbous sheath. The vaginal duct of *A. californica* is short and the vagina is large and bulbous. In contrast, the vaginal duct of *A. paucifoliata* is long and narrow, as is the vagina as it enters the genital atrium. The penis of *A. californica* is unusual in that it is highly coiled whereas that of *A. paucifoliata* is thin and straight.

### Genus*Histiomena*Örsted in Mörch, 1860

#### 

##### 

###### Type species

*Pleurophyllidia marginata*Örsted in Mörch, 1860 by monotypy.

### *Histiomena marginata* (Örsted in Mörch, 1860) (Figs 6, 7)

*Pleurophyllidia* (*Histiomena*) *marginata*Örsted in Mörch, 1860.

**Figure 6:**
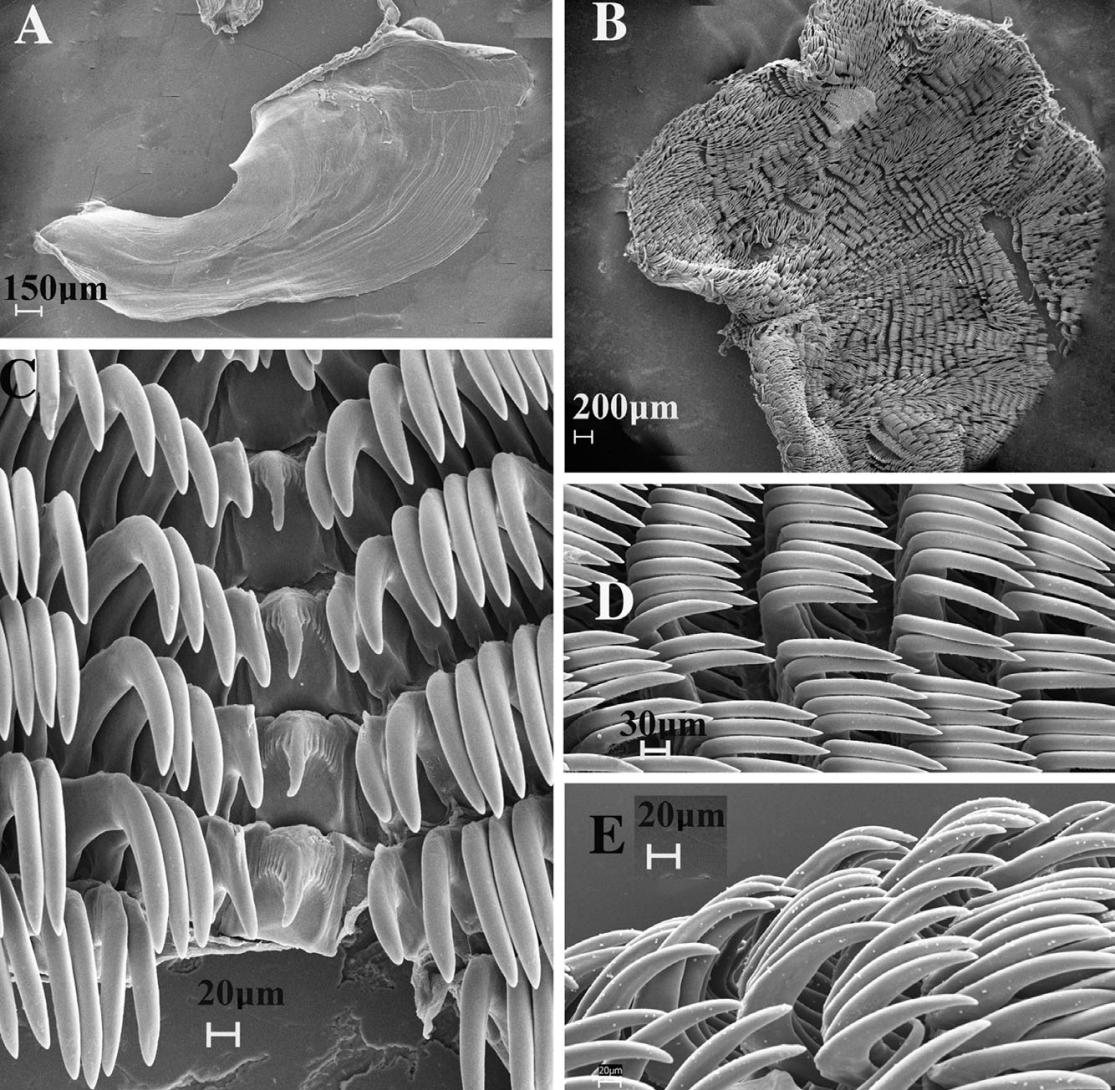
*Histiomena marginata* Örsted in Mörch, 1860, Buccal armature, CASIZ 074274. A, jaw; B, whole radula; C, central portion of radula; D, middle lateral teeth; E, outer lateral teeth.

*Camarga marginata* Bergh, 1866: 69, Marcus & Marcus, 1966: 189.

*Histiomena marginata*Örsted in Mörch, 1860, Marcus & Marcus, 1966: 189.

*Armina convolvula* Lance, 1962: 51, figures 1–6. **syn. nov.**

*Histiomena convolvula* (Lance, 1962) Marcus & Marcus, 1966: 189. **syn. nov.**

#### 

##### 

###### Material examined

CASIZ 074274, one specimen, 60 mm, dissected, Isla de Salsipuedes, Baja California, Mexico, 25 m depth, collected 15.v.1985 by L. Dunne. CASIZ 020300, holotype, 25 km south of San Felipe, Baja California, Mexico, intertidal, 1.iv.1962, collected by F. Wolfson.

###### Geographical distribution

This species is known from the Gulf of California, Costa Rica, and Panama (Camacho-Garcia *et al.*, 2005).

###### External morphology

The body is wide, flattened, and ovate with numerous discontinuous longitudinal ridges composed of small rounded tubercles. The wide foot projects beyond the distinct mantle margin. The oral veil is broad, bilobed with an undulating anterior margin. The foot is broad with tentacular anterior corners. Behind the oral veil are the closely spaced rhinophores. The rhinophores have a series of longitudinal lamellae on the rounded club, which has a rounded apex. The stalk widens as it enters the dorsal cavity and there are no lamellae on the stalk. A caruncle is situated just posterior to the rhinophores. Marginal sacs are not visible along the mantle edge. Under the mantle there are at least 30 branchial lamellae at the anterior end of the body and numerous, wavy rows of hyponotal lamellae at the posterior end. The genital opening is situated in the anterior third of the body wall and the anus opening is approximately half way along the body.

The ground colour of the dorsum and foot is deep brown with numerous opaque white spots. The mantle edge is orange with opaque white spots. The rhinophores are brown with a lighter apex. The oral veil has an orange margin and the dorsal surface of the foot has orange marginal and purple submarginal bands.

###### Buccal armature

The jaws are large and thickly cuticularized (Fig. 6A), with a thick masticatory margin and multiple rows of pointed denticles along the basal portion of the margin (the portion opposite the hinged end of the jaws). The radular formula is 49 × 106.1.1.1.106 (Fig. 6B). The rachidian teeth (Fig. 6C) are narrow with an elongate, rounded central cusp and eight to nine flanking denticles on each side. The flanking denticles are all the same thickness. The inner lateral tooth is posteriorly directed and narrow without denticles. The next several lateral teeth (Fig. 6D) are hook-shaped and lack denticles. The middle lateral teeth have a series of fine denticles along their inner face. The outer lateral teeth (Fig. 6E) lack denticles.

###### Reproductive system

**Figure 7:**
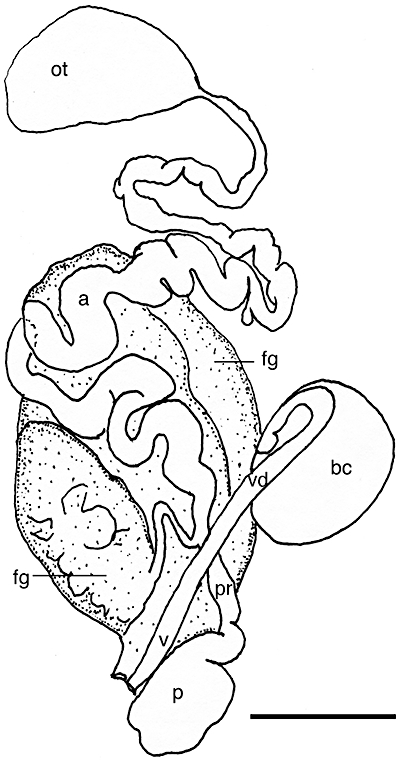
*Histiomena marginata* Örsted in Mörch, 1860, Reproductive system, CASIZ 074274. a, ampulla; bc, bursa copulatrix; fg, female gland mass; ot, ovotestis; p, penis; pr, prostate; v, vagina; vd, vaginal duct. Scale bar = 2.0 mm.

The reproductive organ arrangement is androdiaulic. The ovotestis is large and ovoid. The long hermaphroditic duct leads from the ovotestis into the very long and coiled ampulla (Fig. 7). The ampulla bifurcates near the posterior end of the female gland mass into the short, narrow prostate and the short oviduct. The prostate expands into the very large, bulbous, penial sheath. From the large, round bursa copulatrix the narrow vaginal duct emerges, coils once, and continues to a narrow vagina that exits adjacent to the penial opening.

###### Remarks

The type species of *Histiomena*, *H. marginata* (Örsted in Mörch, 1860), is poorly known and has not been collected since its original description from the Pacific coast of Nicaragua. Bergh (1866) described the radula of this species. Lance (1962) described and drew the external and radular features of *Armina convolvula*, but excluded any information regarding the reproductive system. This species was later transferred to *Histiomena* (Marcus & Marcus, 1966). Marcus & Marcus noted differences in lateral lamellar branching, notal texture, and the shape of the rachidian teeth between *H. marginata* and *H. convolvula.* The rachidian teeth illustrated by Lance (1962: fig. 6a) do not show the prominent median cusp of the rachidian tooth that is evident in the present material (Fig. 6C), also from Baja California, the type locality of *H. convolvula*. The other differences in the ornamentation of the notum and the elaboration of the branchial lamellae are likely to be preservational artefacts and/or differences in observation and description. With the rediscovery of living specimens of *Histiomena* from Costa Rica (near the type locality of *H. marginata*) with coloration identical to specimens from Mexico, the likelihood that these two species are synonymous is greatly increased and we here consider *H. convolvula* to be a junior synonym of *H. marginata*.

### Genus*Dermatobranchus*Hasselt, 1824

#### 

##### 

###### Type species

*Dermatobranchus striatus*, by subsequent designation by Gray, J.E. 1847 in Proceedings of the Zoological Society, London, pt. 15, p. 167.

### Previously described species examined and included in the phylogenetic analysis

### *Dermatobranchus albus* (Eliot, 1904) (Figs 1C–F, 8–10)

*Pleuroleura alba* Eliot, 1904: 104.

*Dermatobranchus albus* (Eliot, 1904) Edmunds & Thompson, 1972: 229.

*Dermatobranchus* sp. 3 Gosliner, 1987: 110, figure 213.

*Dermatobranchus albus* Gosliner, Behrens & Valdés, 2008:310, top three photos.

#### 

##### 

###### Material examined

CASIZ 087896, RFB 3008-B, 9 mm dissected, Seragaki Beach, ENE Maeki-zaki, Okinawa, Ryukyu Islands, Japan, 40 m depth, collected 17.vi.1992 by R. Bolland. CASIZ 105263, RFB 3389-A, 7 mm, dissected, Seragaki Tombs, Okinawa, Ryukyu Islands, Japan, 40 m depth, collected 14.iv.1995 by R. Bolland. CASIZ 174129, eight specimens, 5–10 mm, one 10 mm specimen dissected, Tulear, Madagascar, 3 m depth, collected 5.iv.1989 by T. Gosliner.

CASIZ 099230, one specimen, south side of mouth of Minazi Bay, Mtwara Region, Tanzania, 1 m depth, collected 3.xi.1994 by T. M. Gosliner. CASIZ 174130, 10 mm dissected (stn R42, OT606), Baclayon Takot, Bohol, Philippines, 9°37.1′N, 123°52.6′E, damaged coral reef, 8–22 m, collected 12.vi.2004 by T. Gosliner, Y. Camacho, J. Templado, M. Malaquias, M. Poddubetskaia. CASIZ 068678, one specimen, dissected, Barracuda Point, east side of Pig Island, Madang, Papua New Guinea, 7 m. depth, collected 27.viii.1989 by T. Gosliner. CASIZ 086642, one specimen, Horseshoe cliffs, 1 km west-north-west of Onna Village, Okinawa, Ryukyu Islands, Japan (26°30.0′N, 127°50.9′E), 30 m depth, collected 3.iv.1992 by R. Bolland. CASIZ 087943, one specimen, Seragaki Beach, east-north-east of Maeki-zaki, Okinawa, Ryukyu Islands, Japan (26°30.4′N, 127°52.6′E), 40 m depth, collected 17.vi.1992 by R. Bolland. CASIZ 070153, one specimen 11 mm alive, Horseshoe cliffs, 1 km west-north-west of Onna Village, Okinawa, Ryukyu Islands, Japan, 25 m depth, collected 2.v.1987 by R. Bolland. CASIZ 178237, one specimen, subsampled for DNA, 10 mm preserved, Tiger Point, Pulau Tioman, Malaysia, 25 m depth, collected 2.x.2007 by T. Gosliner. SAM A35756, three specimens, 5–7 mm preserved, two dissected, Adlam's Reef, Indian Ocean, South Africa, 1 m depth, collected 7.v.1982 by T. Gosliner. SAM A35752, one specimen, 7 mm preserved, one dissected, Adlam's Reef, Indian Ocean, South Africa, 1 m depth, collected 7.v.1982 by T. Gosliner.

###### Geographical distribution

This species has been reported from South Africa (present study), Tanzania (Eliot, 1904; Edmunds & Thompson, 1972; present study), Madagascar (present study), Singapore (Coleman, 2008), Sulawesi (Lindsay Warren, SeaSlug Forum), Papua New Guinea, eastern Malaysia, Philippine Islands, and Okinawa (present study).

###### External morphology

The body shape of the living animal (Fig. 1C–F) is slender, elongate, flattened, and narrows at the posterior end. The foot does not project beyond the distinct mantle margin. There is a series of longitudinal dorsal ridges. The oral veil extends forward and is rounded at the corners. The rhinophores are behind the oral veil. They have a series of longitudinal lamellae on the rounded club. The stalk narrows slightly. There are marginal sacs on the underside of the mantle edge. About halfway along the body on the right side is the genital opening. The anus is situated approximately halfway towards the posterior end of the body.

The ground colour of the dorsum, foot, and oral veil is highly variable (Fig. 1C–F); opaque white to grey or black as are the dorsal ridges. The depressions between the ridges range from bright orange to blue or black. The dorsal ridges of some specimens from the type locality, Tanzania, and from Tulear have an orange crest. The mantle edge is orange. The rhinophore stalk is white and the club is black, with white or pale blue longitudinal lamellae and an orange apex. The oral veil is opaque white with a bright yellow or orange margin.

###### Buccal armature

**Figure 8:**
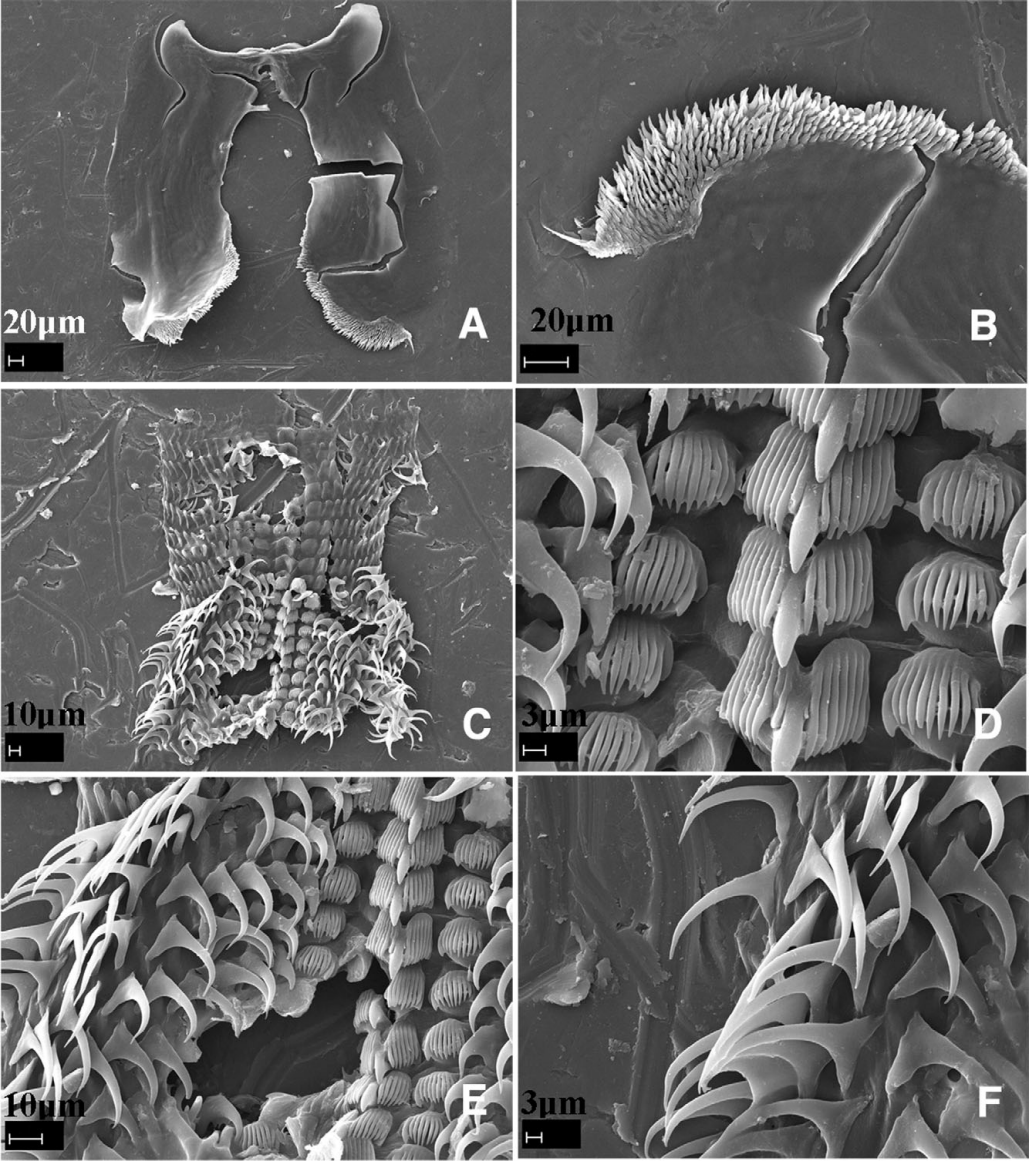
*Dermatobranchus albus* (Eliot, 1904). Buccal armature, CASIZ 174130. A, jaws; B, masticatory margin; C, whole radula; D, central portion of radula; E, middle lateral teeth; F, outer lateral teeth.

**Figure 9:**
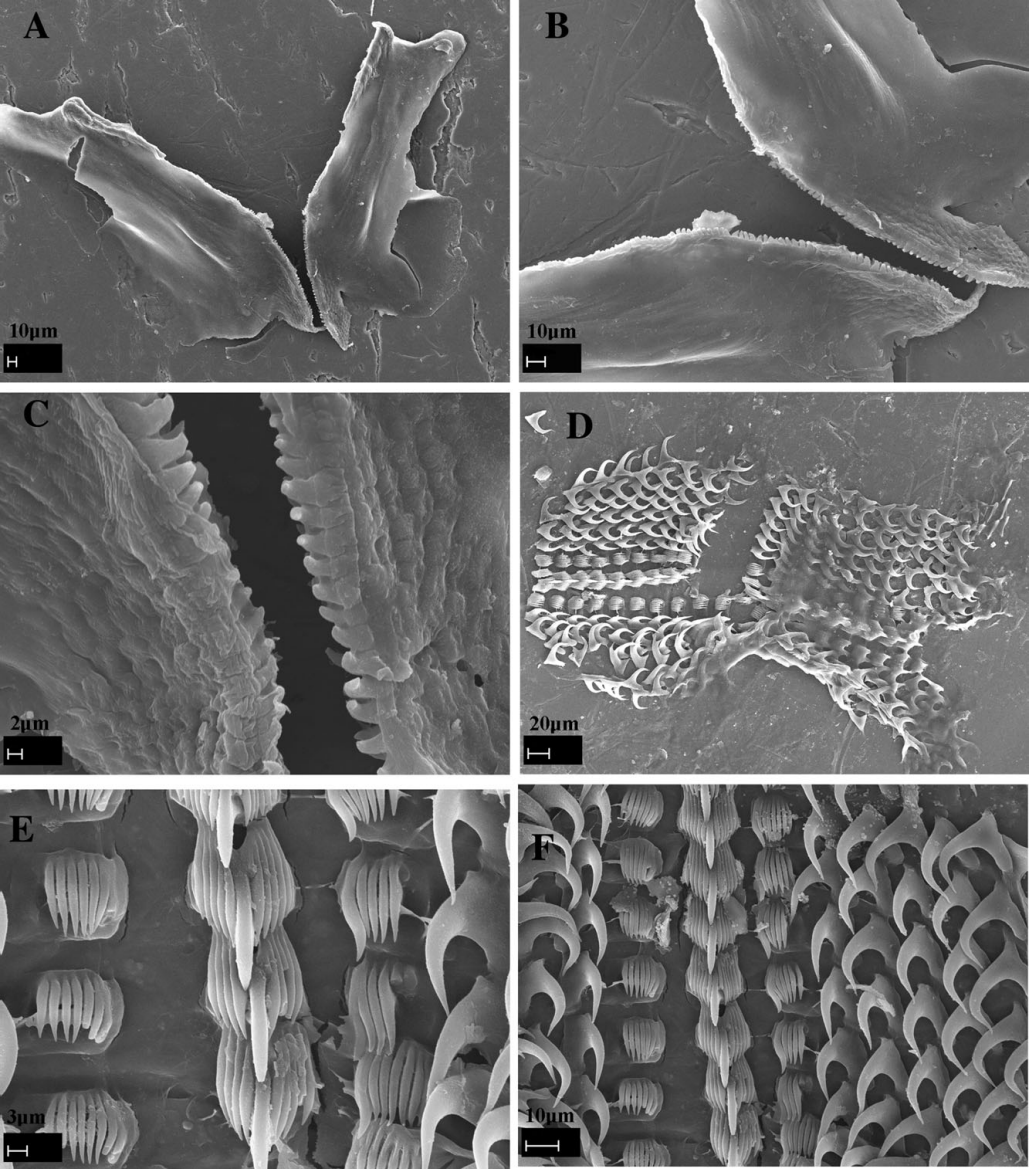
*Dermatobranchus albus* (Eliot, 1904). Buccal armature, CASIZ 174129, Tulear, Madagascar. A, B, jaws; C, masticatory margin; D, whole radula; E, central portion of radula; F, middle lateral teeth.

**Figure 10:**
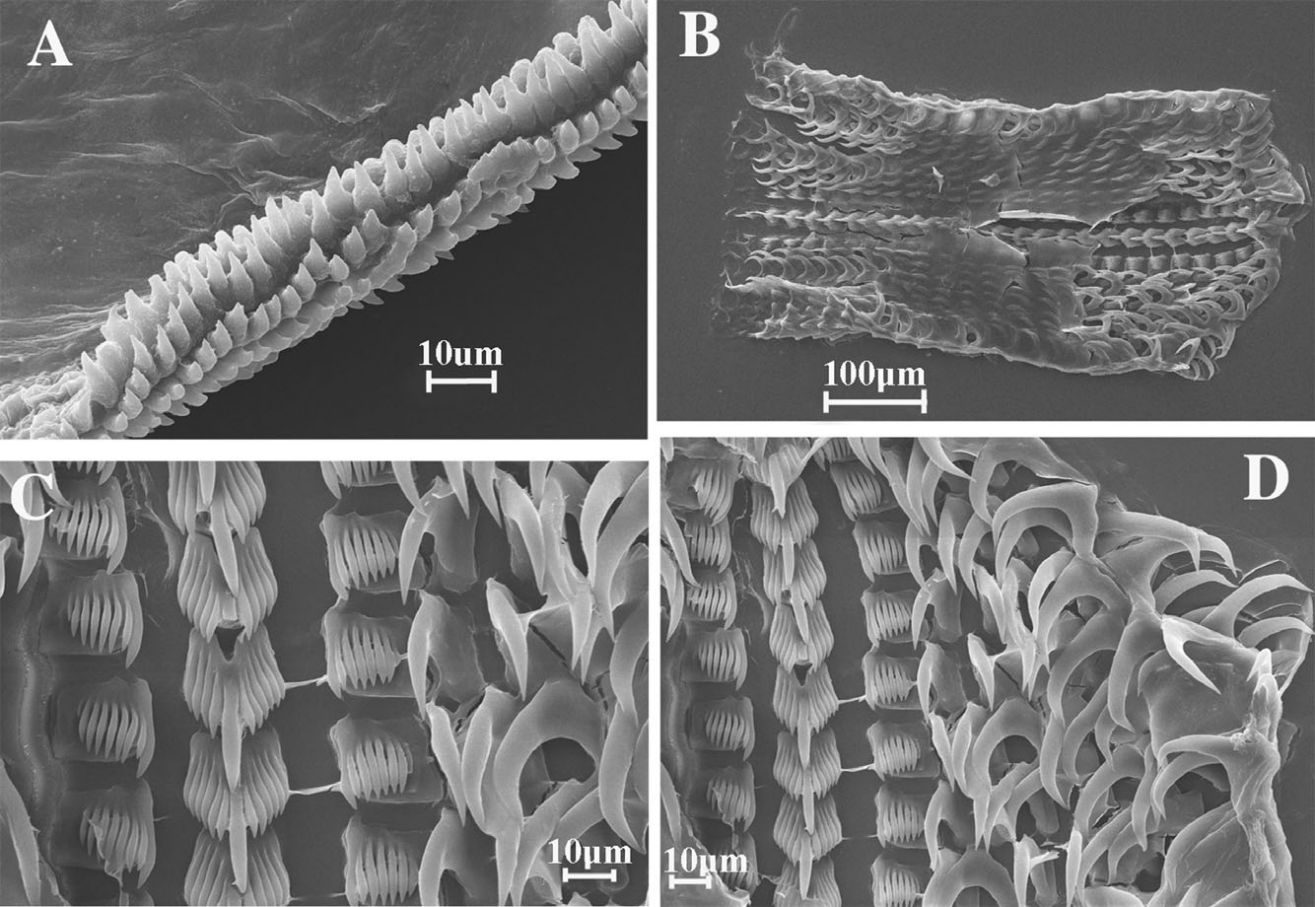
*Dermatobranchus albus* (Eliot, 1904). Buccal armature, CASIZ 068678, Madang, Papua New Guinea. A, masticatory margin; B, whole radula; C, central portion of radula; D, middle lateral teeth.

The jaws are large and thickly cuticularized (Figs 8A, 9A, B), with a thick masticatory margin and six to eight rows of simply pointed denticles along the basal margin (Figs 8B, 9C, 10A). The radula is slightly longer than wide (Figs 8C, 9D, 10B). The radular formula of a specimen from Okinawa (CASIZ 105263) is 21 × 7.1.1.1.7. The radular formula of (CASIZ 068678) is 28 × 7–9.1.1.17–9. The radular formula of a specimen from Madagascar (CASIZ 174129) is 23 × 7.1.1.1.7 and the formula of a specimen from the Philippines (CASIZ 174130) is 21 × 9–10.1.1.1.9–10. The radular formulae from two specimens from South Africa (SAM A 35756) are 25 × 7–8.1.1.1. 7–8 and 21 × 7.1.1.1.7. In all cases, the rachidian tooth (Figs 8D, 9E, 10C) is broad with a large, pointed central cusp that extends well beyond the five to nine flanking denticles on each side. The moderately wide, laterally directed inner lateral tooth (Figs 8D, 9E, 10C) is claw-shaped and has six to nine nearly equal-in-length pointed denticles. The next seven to ten lateral teeth (Figs 8E, 8F, 9F, 10D) are simple hooks with no denticles.

###### Reproductive system

**Figure 11:**
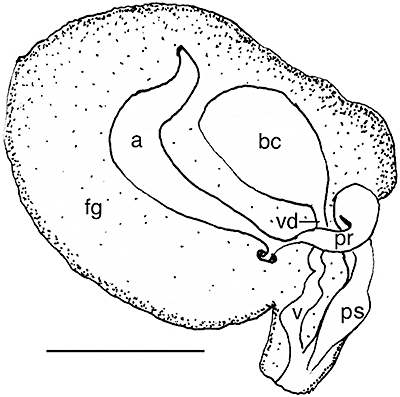
*Dermatobranchus albus* (Eliot, 1904), Reproductive system, CASIZ 174130, Panglao, Philippines. a, ampulla; bc, bursa copulatrix; fg, female gland mass; pr, prostate; ps, penial sheath; v, vagina; vd, vaginal duct. Scale bar = 1.0 mm.

The reproductive organ arrangement is androdiaulic. The short hermaphroditic duct leads into the tubular ampulla (Fig. 11). The ampulla bifurcates near the centre of the female gland mass into the short, narrow prostate and short oviduct. The prostate expands into the large, bulbous, penial sheath. From the round bursa copulatrix the narrow vaginal duct emerges and continues to a narrow vagina that exits adjacent to the penial opening.

###### Remarks

Eliot (1904) described the external and radular morphology of this species, but he did not describe the reproductive system. The specimens we examined from Madagascar, Okinawa, the Philippines, Papua New Guinea, and the type locality, Tanzania, agree with Eliot's description of the external and radula morphology and with some aspects of the coloration. However, we found considerable colour variation amongst specimens from within the same geographical region, such as Tulear, Madagascar and from distant geographical regions such as Okinawa, the Philippines, and Papua New Guinea.

With regard to the external morphology, all the specimens we examined have the same elongate, tapering body shape, distinct low ridges that are longitudinal but not parallel to the median line and a large velum as described by Eliot (1904). The primary difference that we found between Eliot's colour description and the specimens we examined is that not all specimens have a yellow line along the ridge crest. Some have orange or yellow in the depressions between white crested ridges and some specimens have black or blue in the depressions. Not all specimens have rhinophores with a yellow apex. Specimens from Okinawa for example have elongate, pale blue apices. However, all these specimens have the same radula and reproductive morphology.

Edmunds & Thompson (1972) provided further details of the external coloration of specimens they examined from Tanzania. They noted grey coloration between the ridges. They also noted that the ridges were ‘almost parallel’ and that some smaller specimens had discontinuous dorsal ridges.

Eliot (1904) noted that his new species was closely allied to *D. striatus* (as *Pleuroleura striata*) (Hasselt, 1824). He speculated that *D. albus* (as *Pleuroleura alba*) might ultimately prove to be a variety of *P. striata*. However, there are major differences between these two species, both external and internal. See the Remarks section of *D. striatus* for further details on this species.

*Dermatobranchus albus* is part of a complex of species that have a radula with a moderately broad inner lateral tooth, which possesses numerous comb-like denticles. The remaining laterals are devoid of denticles. Members of this complex include *D. albus*, *D. otome*, *D. striatus*, *D. diagonalis*, and *D. oculus*, all of which have this arrangement of radular teeth.

### *Dermatobranchus fortunatus* (Bergh, 1888) (Figs 1G–I, 12–14)

*Pleuroleura fortunata* Bergh, 1888: 353: pl. 10, pl. 11, figures 1–9.

**Figure 12:**
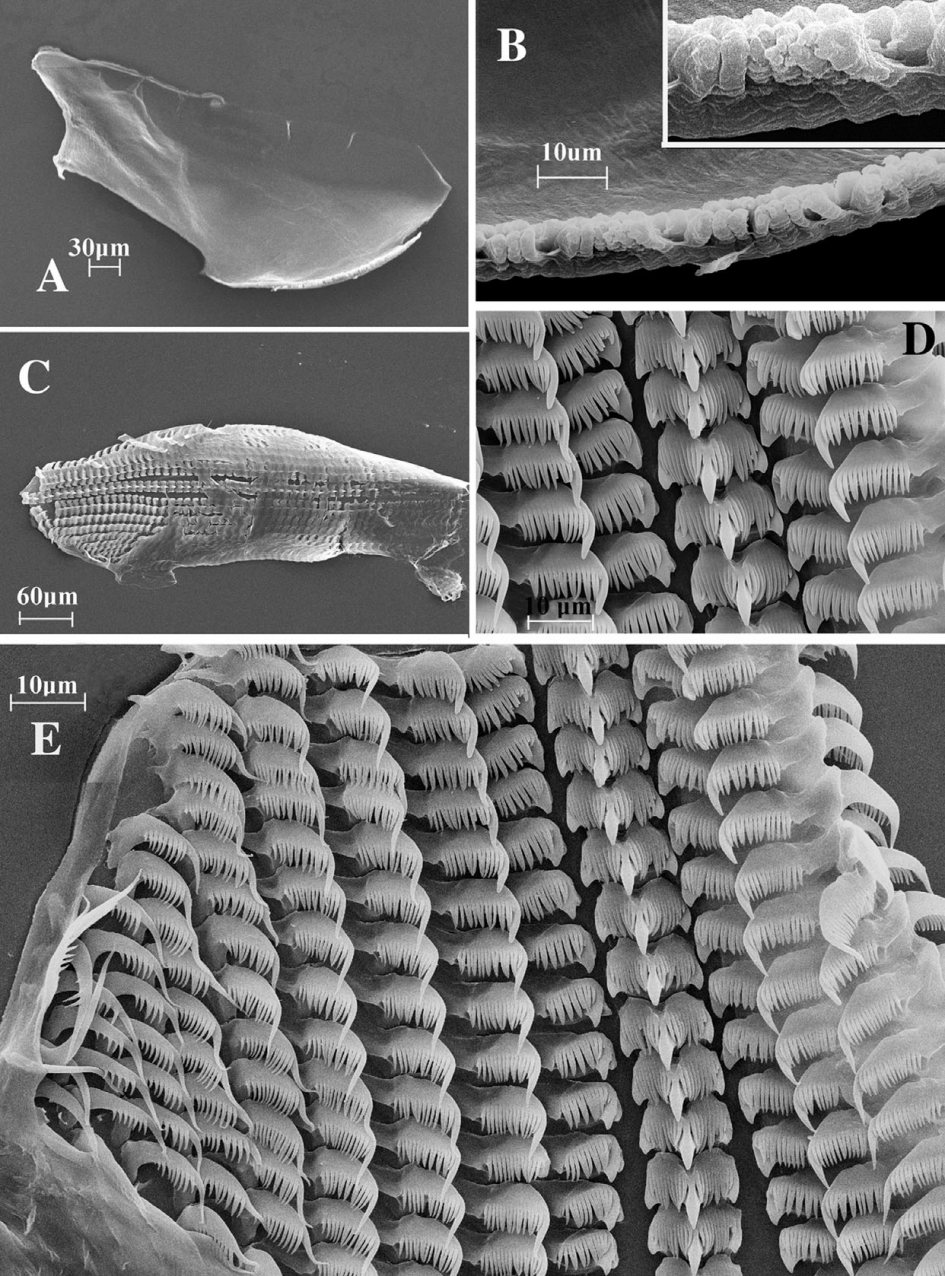
*Dermatobranchus fortunatus* (Bergh, 1888). Buccal armature, CASIZ 074158 Mahe, Seychelles. A, jaw; B, masticatory margin; C, whole radula; D, central portion of radula; E, middle and outer lateral teeth.

**Figure 13:**
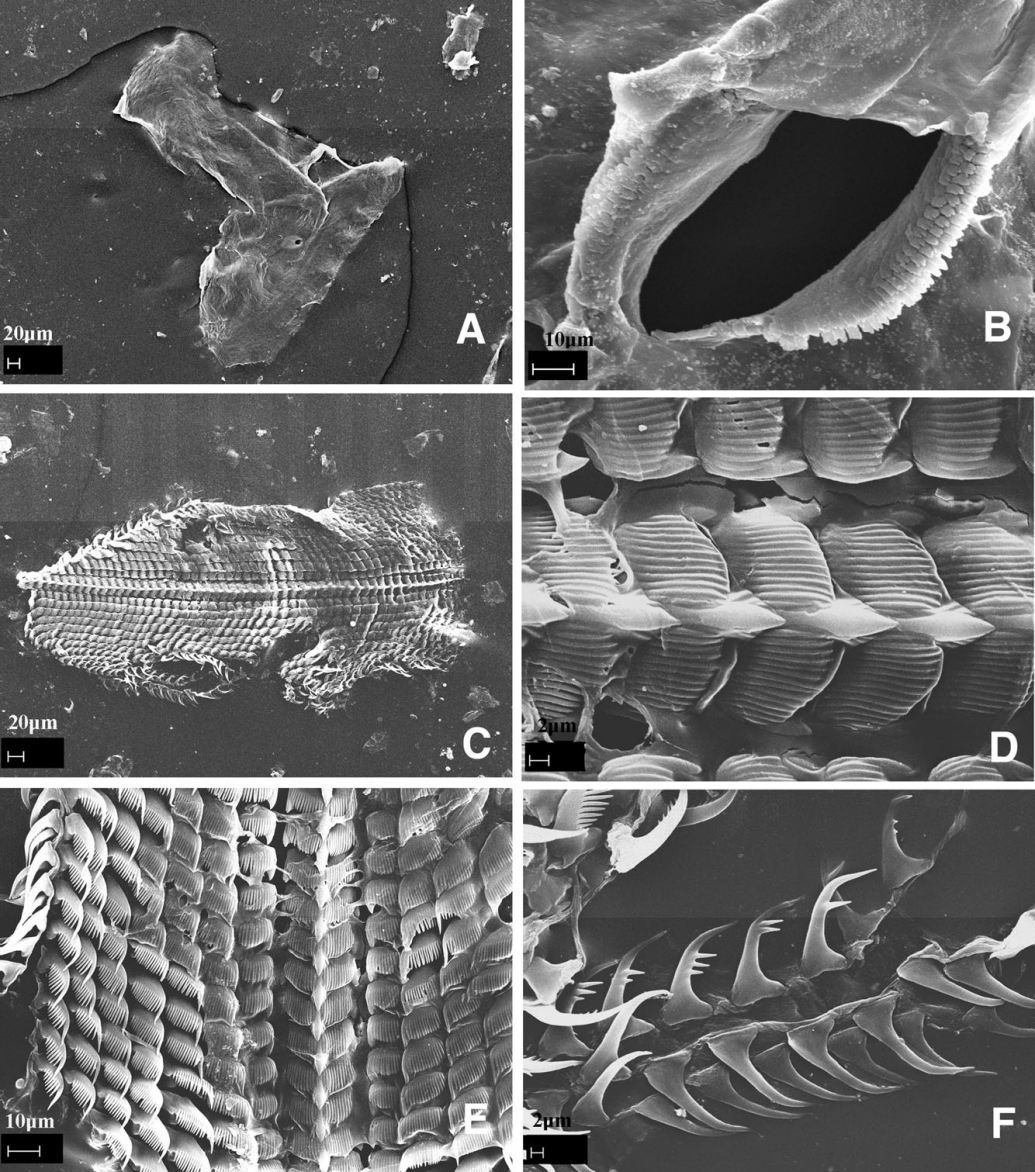
*Dermatobranchus fortunatus* (Bergh, 1888). Buccal armature, CASIZ 121167, Enewetak Atoll, Marshall Islands. A, jaws; B, masticatory margin; C, whole radula; D, central portion of radula; E, middle lateral teeth; F, outer lateral teeth.

**Figure 14:**
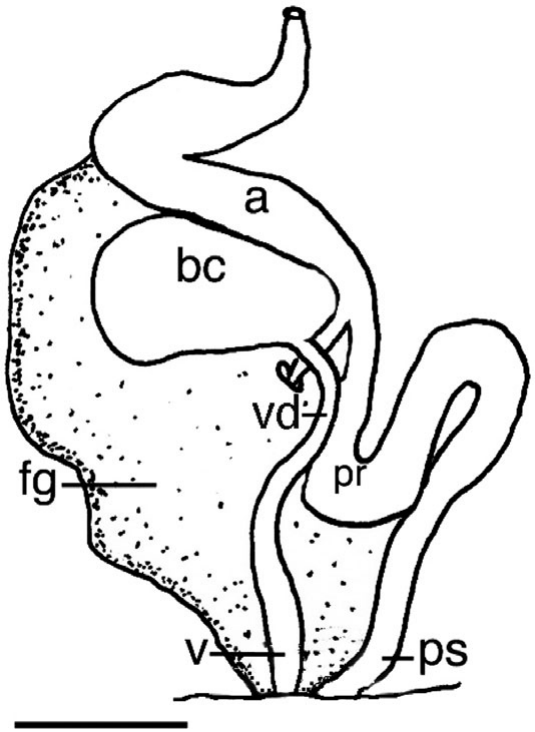
*Dermatobranchus fortunatus* (Bergh, 1888). Reproductive system, CASIZ 074158, Mahe, Seychelles. a, ampulla; bc, bursa copulatrix; fg, female gland mass; pr, prostate; ps, penial sheath; v, vagina; vd, vaginal duct. Scale bar = 0.5 mm.

*Dermatobranchus fortunatus* Gosliner, Behrens & Valdés, 2008:310, above bottom photo.

#### 

##### 

###### Material examined

CASIZ 065771, three specimens, one dissected, Jais Aben Jetty, Madang, Papua New Guinea, intertidal zone, collected 10.i.1988 by T. Gosliner. CASIZ 074158, one specimen, 5 mm preserved, dissected, Seychelles Airport, Mahe Island, Republic of Seychelles, 2 m depth, collected i.1991 by T. Gosliner. CASIZ 121167, one specimen, 9 mm preserved, Bokandretok Medren Reef, Enewetak Atoll, Marshall Islands, 1 m depth, collected 30.i.1982 by S. Johnson. CASIZ 171455, one specimen, Alona Reef, Panglao, Philippines, stn B2 (9°33.0′N, 123°46.5′E), reef slope, 5 m depth, collected 31.v.2004 by T. Gosliner, Y. Camacho, J. Templado, M. Malaquias, M. Poddubetskaia. CASIZ 174136, one specimen, stn M3, Tokong Kamundi, Malaysia, 15 m depth, collected 29.ix.2007 by T. Gosliner. CASIZ 177770, one specimen, Devil's Point, Maricaban Island, Batangas Province, Luzon, Philippines, 7 m depth, collected, 23.iv.2008 by T. Gosliner.

###### Geographical distribution

This species is known from the Indian Ocean of Java (Bergh, 1888), the Great Barrier Reef, Australia (Marshall & Willan, 1999), Okinawa, Japan (Bolland, 2003), the Seychelles, Marshall Islands, Indonesia, Papua New Guinea, eastern Malaysia, and the Philippines (present study).

###### External morphology

The specimens examined from Papua New Guinea match Bergh's (1888) description and drawings of his specimens collected from Indonesia.

###### Buccal armature

The jaws are large and thickly cuticularized (Figs 12A, 13A), with a thick masticatory margin and two to three rows of multifid, pointed denticles along the basal edges (Figs 12B, 13B). The radula (Figs 12C, 13C) is much longer than wide. The radular formula of (CASIZ 065771) is 54 × 11.1.1.1.11 and (CASIZ 074158) is 56 × 8.1.1.1.8. The rachidian teeth (Figs 12D, 13D) are broad with a large, spear-shaped central cusp that extends beyond the 10–14 flanking denticles on each side. The flanking denticles are all the same thickness. Each rachidian tooth extends outward from a broad base. The inner lateral teeth are extremely broad and laterally directed with 12–14 elongate denticles on the outer side of the short central cusp. The next six lateral teeth (Figs 12E, 13 E, F) are comb-shaped with a longer, blunt ended first cusp and up to nine smaller, pointed denticles. The next two lateral teeth are also comb-shaped, but the first cusp is longer and more pointed than the previous five lateral teeth. The next two teeth are very pointed hooks with long, sharp denticles on the outer edge. The last two teeth are hook-shaped, have no denticles and are the same length as the previous four lateral teeth.

###### Reproductive system

The reproductive organ arrangement is androdiaulic. The thick hermaphroditic duct leads into the curved, tubular ampulla (Fig. 14). The ampulla bifurcates into the oviduct, which enters the female gland mass and into the elongate prostate. The prostate thickens as it curves back and does not expand at all into a penial sheath. The ovoid bursa copulatrix is approximately the same size as the ampulla. From the bursa, the long, narrow vaginal duct leads into the narrow vagina, which exits into the genital aperture next to the penial sheath.

###### Remarks

Bergh (1888) provided only a brief description of external morphology, the radula, and the reproductive system. The external pattern of dorsal markings shown in Bergh's figure (Fig. 1G) closely matches that of the present material. With regard to reproductive anatomy, Bergh drew only the penis of this species. He showed the penis to be conical and enlarged from the prostate. The specimens we examined did not have an enlarged penial sheath.

The radular morphology of the specimens we examined matched Bergh's drawings and description.

This species is one of the more commonly encountered species of arminids in the Indo-Pacific tropics. Like the preceding species, it is widespread from the western Indian Ocean to the Central Pacific of the Marshall Islands.

### *Dermatobranchus gonatophorus* Hasselt, 1824 (Figs 15A, C, 16–17)

*Dermatobranchus gonatophorus* Hasselt, 1824:243.

**Figure 15:**
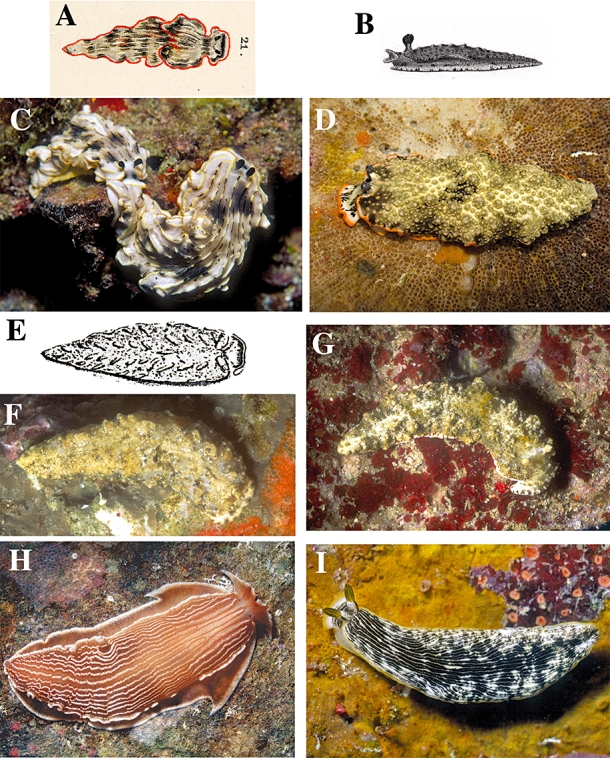
Living animals. A., *Dermatobranchus gonatophorus* Hasselt, 1824, after Bergh, 1905. B, *Dermatobranchus ornatus* (Bergh, 1874), after Bergh, 1874. C, *Dermatobranchus gonatophorus* Hasselt, 1824, Thailand, photo by Mark Strickland. D, *Dermatobranchus ornatus* (Bergh, 1874), Ligpo, Philippines, photo by T. Gosliner. E, *Dermatobranchus pustulosus* Hasselt, 1824, after Bergh, 1887. F, *Dermatobranchus pustulosus* Hasselt, 1824, CASIZ 083798, Mabini, Luzon, Philippines, photo by T. Gosliner. G, *Dermatobranchus pustulosus* Hasselt, 1824, CASIZ 085904, Dakak, Mindanao, Philippines, photo by T. Gosliner. H, *Dermatobranchus rubidus* (Gould, 1852), CASIZ 105696, Mabini, Luzon, Philippines, photo by T. Gosliner. I, *Dermatobranchus striatus* Hasselt, 1824, CASIZ 086314, Madang, Papua New Guinea, photo by T. Gosliner.

**Figure 16:**
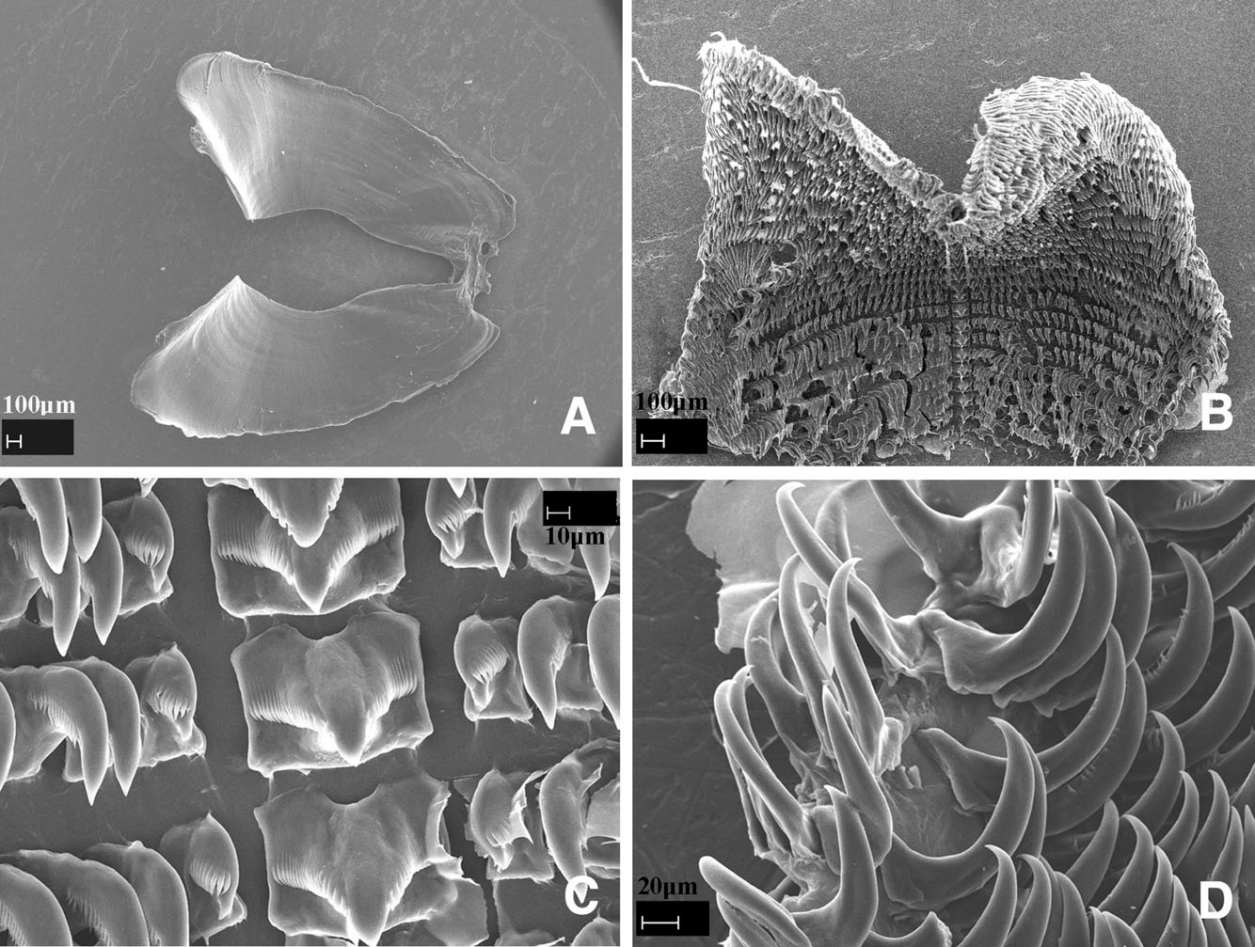
*Dermatobranchus gonatophorus* Hasselt, 1824. Buccal armature, CASIZ 105299, Okinawa, Japan. A, jaws; B, whole radula; C, central portion of radula; D, outer lateral teeth.

**Figure 17:**
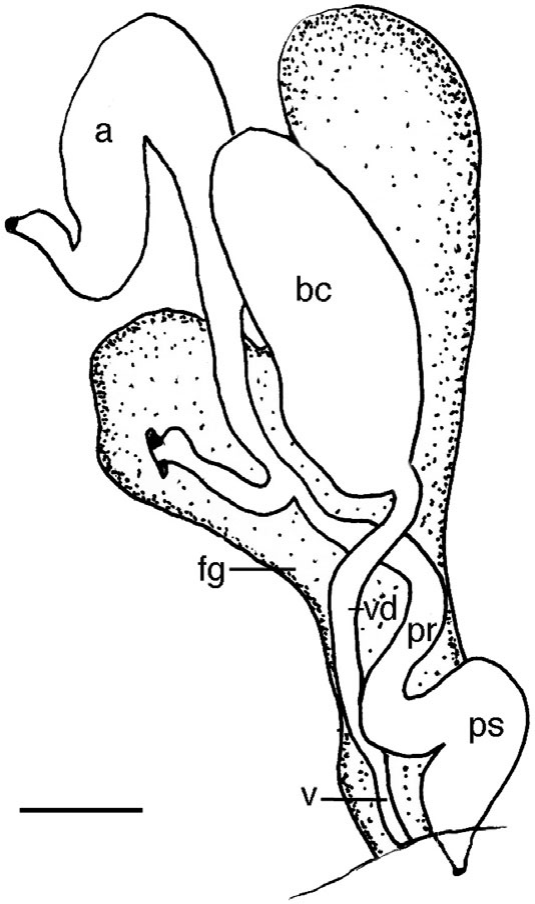
*Dermatobranchus gonatophorus* Hasselt, 1824. Reproductive system, CASIZ 105299, Okinawa, Japan. a, ampulla; bc, bursa copulatrix; fg, female gland mass; pr, prostate; ps, penial sheath; v, vagina; vd, vaginal duct. Scale bar = 0.67 mm.

*Pleuroleura gonatophora* (Hasselt), Bergh, 1888: 353.

*Dermatobranchus gonatophorus* Gosliner, Behrens & Valdés, 2008:307, just above bottom photo.

#### 

##### 

###### Material examined

CASIZ 105299, one specimen, dissected, Seragaki Tombs, Okinawa, Japan, 58 m depth, collected 14.iii.1995 by R. Bolland (RFB 3375).

###### Geographical distribution

This species is known from the western Indian Ocean of South Africa and the Red Sea to Thailand, Indonesia, Malaysia (Rudman, 2000a), and Okinawa, Japan (present study).

###### External morphology

The specimens examined from Okinawa match Hasselt's (1824) written description and Bergh's (1905) drawing. We did notice variation in the dorsal coloration between the specimen and photos (Fig. 15C; Rudman, 2000a) that we examined and the earlier descriptions. The primary difference we noted is the presence of yellow dorsal ridge ridges in our specimens. Specimens consistently have black rhinophores.

###### Buccal armature

The jaws are large and thickly cuticularized (Fig. 16A), with a thick masticatory margin that is devoid of denticles along the margin. The radular formula of one specimen (CASIZ 105299) is 19 × 5.30.1.1.1.30.5 (Fig. 16B). The rachidian teeth (Fig. 16C) are broad with a broad, pointed central cusp that extends beyond the 18–21 flanking denticles on each side. The flanking denticles are thicker closest to the central cusp, and gradually thinning towards the base. Each rachidian extends outward from a broad base that has a curved upper edge. The narrow inner lateral tooth (Fig. 16C) is posteriorly directed, compact, hooked, and denticulate with a longer pointed first cusp and up to nine smaller denticles. The next 30 lateral teeth are also compact hooks, with tiny denticles. The last five teeth (Fig. 16D) are hook-shaped, have no denticles, and are smaller than the other lateral teeth.

###### Reproductive system

The reproductive organ arrangement is androdiaulic. The hermaphroditic duct leads into the curved, tubular ampulla (Fig. 17). The ampulla bifurcates into the short oviduct, which enters the female gland mass and into the elongate, narrow prostate. The prostate curves once, and then expands into the bulbous penial sheath. The ovoid bursa copulatrix is much larger in size than the ampulla. From the bursa, the long, narrow vaginal duct leads into the narrow vagina, which exits into the genital atrium next to the penial sheath.

###### Remarks

Hasselt (1824) did not describe the radular or reproductive morphology of *D. gonatophorus,* but he did describe the external morphology and coloration. Bergh (1887, 1905) also presented a description and drawing of this species. The specimen examined for the present study matches the original description of the external morphology, with coloration variation as noted above.

### *Dermatobranchus ornatus* (Bergh, 1874) (Figs 15B, D, 18, 19)

*Pleuroleura ornata* Bergh, 1874: 278, pl. 25, figure 3, pl. 34 figures 27–32, pl. 35. Bergh 1888: 353, pl. 11, figure 22.

**Figure 18:**
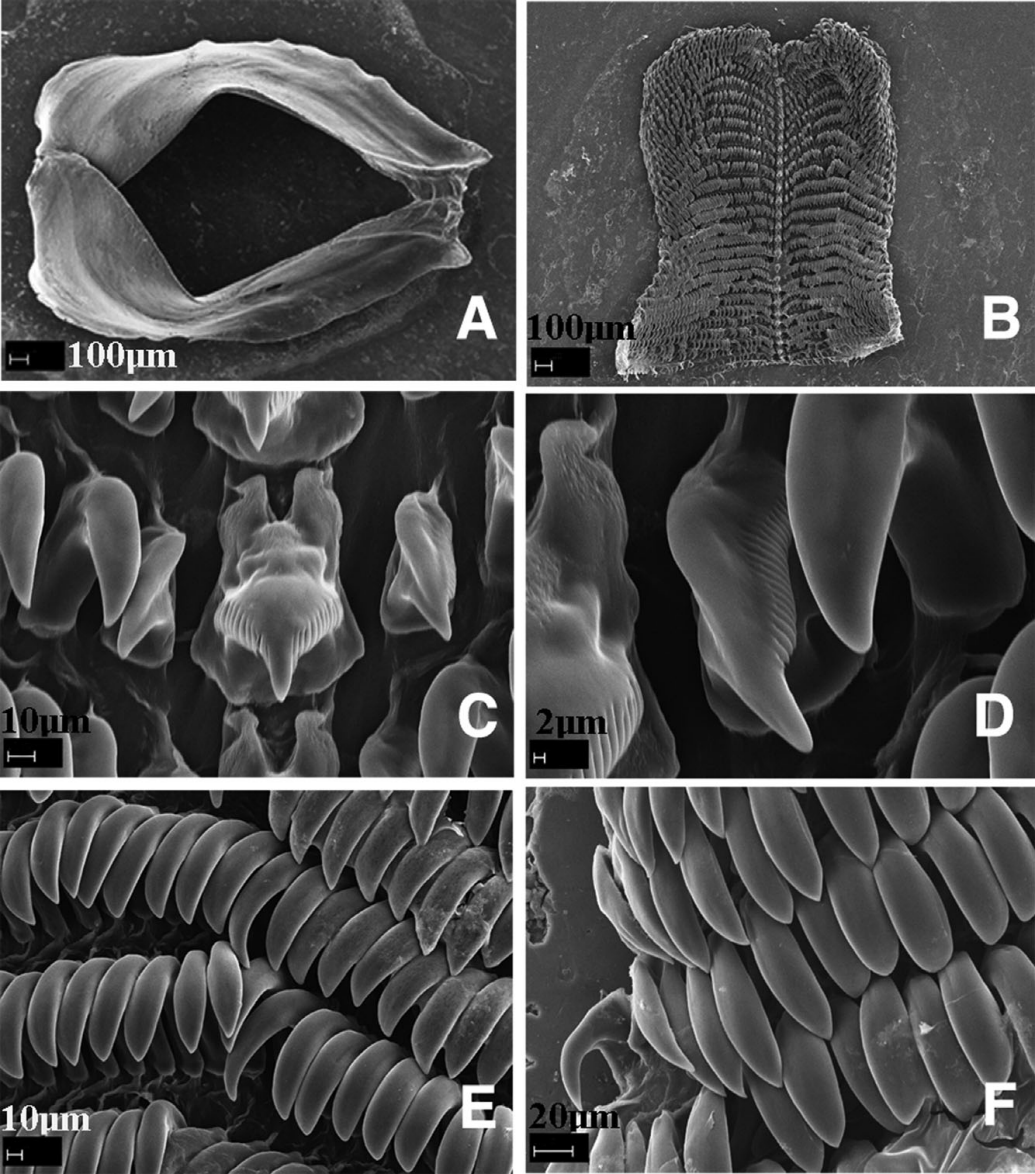
*Dermatobranchus ornatus* (Bergh, 1874). Buccal armature, CASIZ 144008, Queensland, Australia. A, jaws; B, whole radula; C, central portion of radula; D, inner lateral teeth; E, middle lateral teeth; F, outer lateral teeth.

**Figure 19:**
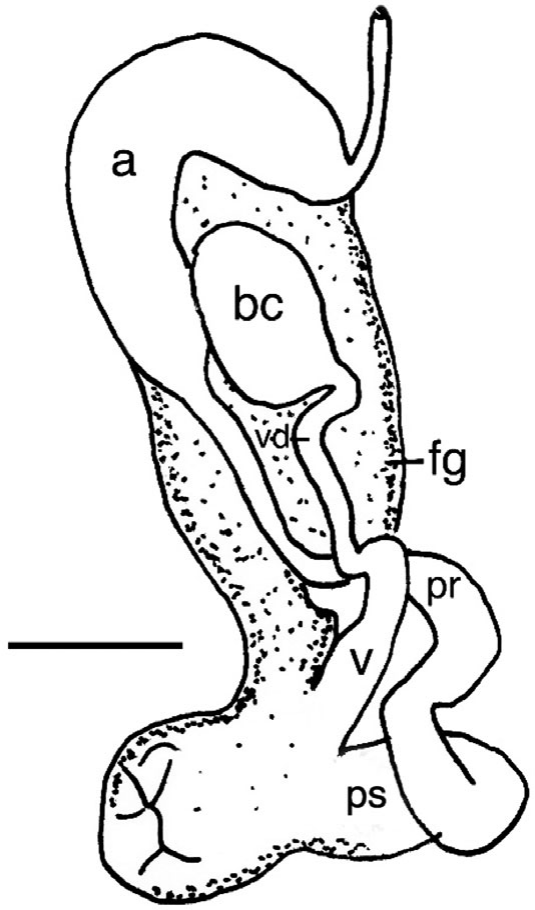
*Dermatobranchus ornatus* (Bergh, 1874). Reproductive system, CASIZ 144008, Queensland, Australia. a, ampulla; bc, bursa copulatrix; fg, female gland mass; pr, prostate; ps, penial sheath; v, vagina; vd, vaginal duct. Scale bar = 1.0 mm.

*Dermatobranchus ornatus* Gosliner, Behrens & Valdés, 2008:307, bottom photo.

#### 

##### 

###### Material examined

CASIZ 144033, one specimen, 28 mm preserved, Mooloolaba, Queensland, Australia, 15 m depth, collected 24.xii.2000 by S. Fahey.

CASIZ 144008, one specimen, 22 mm preserved, dissected, Mooloolaba, Queensland, Australia, 15 m depth, collected 27.xii.2000 by S. Fahey. CASIZ 159387, one specimen, 35 mm preserved, dissected, Mooloolaba, Queensland, Australia, 17 m depth, collected 15.i.2002 by Y. Valles. CASIZ 156334, one specimen, 40 mm preserved, Mooloolaba, Queensland, Australia, 15 m depth, collected 24.vi.2001 by S, Fahey. CASIZ 167458, one specimen, Manado, Sulawesi, Indonesia, collected iv.2003 by Constantinos Petrinos. CAS 177638, one specimen, Lipgo Island, Balayan Bay, Luzon, Philippines, 25 m depth, collected 18.iv.2008 by T. Gosliner. CAS 1776398, one specimen, Lipgo Island, Balayan Bay, Luzon, Philippines, 25 m depth, collected 18.iv.2008 by T. Gosliner.

###### Geographical distribution

This species is known from Oman, Japan, Thailand, Malaysia, the Philippine Islands, Indonesia (present study), and Australia (Gosliner *et al.*, 2008)

###### External morphology

The external morphology of the specimens examined from the south Queensland coast of Australia match Bergh's (1874) description and drawing. Some dorsal colour variation is found amongst specimens within the geographical range of this species (Rudman, 2000b). The background colour ranges from white to black and the dorsal tubercle colour ranges from white to orange to pink. The dorsal tubercles can be large and flattened or smaller and simply rounded. The rhinophores are black with opaque white lines on the margins of the rhinophoral lamellae.

###### Buccal armature

The jaws are large and thickly cuticularized (Fig. 18A), with a thick masticatory margin that is devoid of denticles along the margin. The radular formula of one specimen (CASIZ 144008) is 28 × 36.1.1.1.36 (Fig. 18B). The rachidian teeth (Fig. 18C) are broad with a large, pointed central cusp that extends well beyond the 11 flanking denticles on each side. Each rachidian extends outward from a broad base that has two elongate posterior flanges. The narrow, posteriorly directed inner lateral tooth (Fig. 18D) is hooked and denticulate with a large median bulge extending the length of the hook. The inner lateral tooth has about 21 denticles on its outer side. The next 36 lateral teeth (Fig. 18E) are also hooks, with no denticles or with a few tiny ones. The outermost two teeth (Fig. 18F) are smaller than the others.

###### Reproductive system

The reproductive organ arrangement is androdiaulic. The elongate hermaphroditic duct leads into the thick, tubular ampulla (Fig. 19). The ampulla bifurcates into a short oviduct, which enters the female gland mass, and into the elongate, narrow prostate. The prostate expands into the bulbous penial sheath. The round bursa copulatrix is much smaller in size than the ampulla. From the bursa, the long, narrow vaginal duct expands into the wider vagina, which exits into the genital atrium next to the penial sheath.

###### Remarks

The specimens we examined from the east coast of Australia match Bergh's (1874) original description and drawings of the external and radular morphology. Until now, no drawings were published of the reproductive organs.

### *Dermatobranchus otome* Baba, 1992 (Figs 20, 21)

*Pleuroleura striata* Eliot, 1913: 41, misidentification.

**Figure 20:**
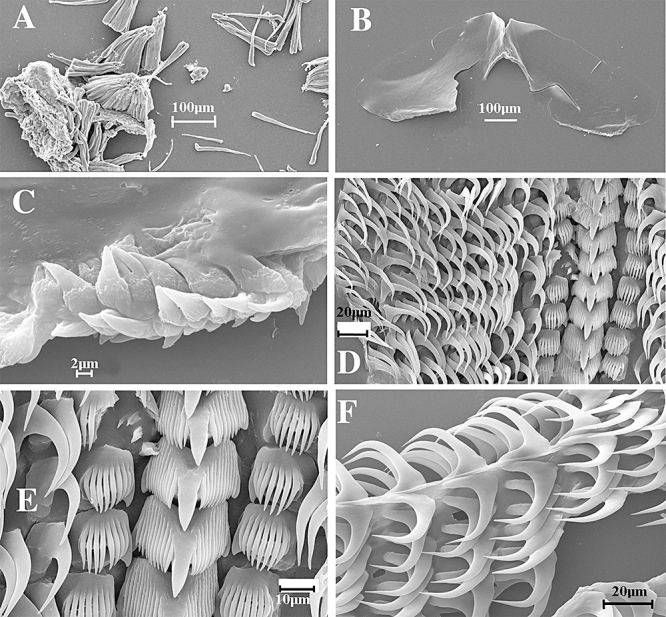
*Dermatobranchus otome* Baba, 1992. Notal and buccal armature, CASIZ 082031, Sagami Bay, Japan. A, marginal sac rodlets; B, jaws; C, masticatory margin; D, half radular width; E, central portion of radula; F, outer lateral teeth.

**Figure 21:**
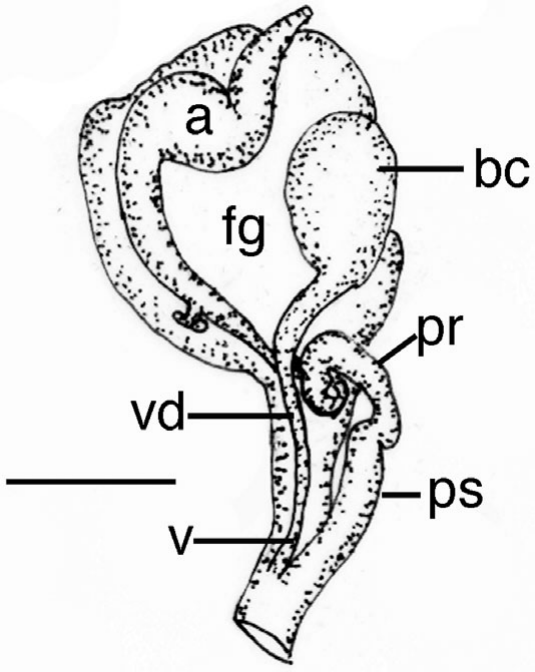
*Dermatobranchus otome* Baba, 1992. Reproductive system, CASIZ 082031, Sagami Bay, Japan. a, ampulla; bc, bursa copulatrix; fg, female gland mass; pr, prostate; ps, penial sheath; v, vagina; vd, vaginal duct. Scale bar = 0.6 mm.

**Figure 83:**
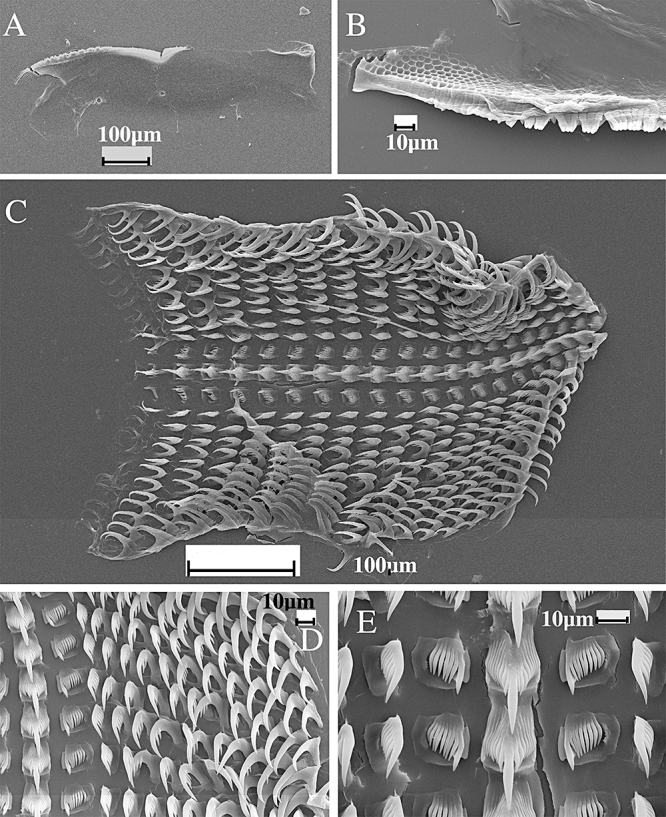
***Dermatobranchus tuberculatus* sp. nov.** Buccal armature, CASIZ 112297, Ligpo Island, Balayan Bay, Luzon, Philippines. A, jaw; B, masticatory margin; C, entire radula; D, half-row of radular teeth; E, central portion of radula.

*Dermatobranchus striatus* Baba, 1937: 316, pl. 2 figure 1, text figure 12; Baba, 1949: 73, 157, p. 29, figure 109, text figure 83; Baba, 1976: 4, figures 1, 2. misidentifications.

*Dermatobranchus otome* Baba, 1992: 242, figures 3, 5, pl. 1, figure 3.

#### 

##### 

###### Material examined

CASIZ 082031, three specimens, two dissected, 17 mm preserved, Chosaga Saki, Sagami Bay, Japan, 0.2 m depth, collected 3.vi.1970 by F. Steiner. CASIZ 082065, one specimen, 22 mm preserved, Chosaga Saki, Sagami Bay, Japan, collected 31.iii.1970 by F. Steiner. CASIZ 081833, two specimens, 14 mm preserved, Chosaga Saki, Sagami Bay, Japan, 10 m depth, collected 21.vi.1970 by F. Steiner.

###### Geographical distribution

This species is found only from the central portion of Japan (Eliot, 1913; Baba, 1937, 1949, 1976, 1992).

###### External morphology

The preserved specimens still retain black spots surrounded by clearer areas and a dark patch was present on the central portion of the notum. The remainder of the body is white.

Expanded marginal sacs are visible that contain masses of rodlets (Fig. 20A) and there are no branchial lamellae or hyponotal lamellae. The anal opening is situated approximately halfway along the body side and the genital opening is at the anterior third of the body wall.

###### Buccal armature

The jaws are large, thickly cuticularized (Fig. 20B), with a thick masticatory margin and three to four rows of long, pointed denticles (Fig. 20C), some of which are bifid. The radula formula (Fig. 20D) of two specimens from CASIZ 082031 is 31 × 16.1.1.1.16 and 32 × 15.1.1.1.15. The rachidian teeth (Fig. 20E) are wide with a broad, moderately elongate central cusp. There are 11–12 elongate denticles on either side of the cusp. The inner lateral teeth are moderately broad and laterally directed with 7–11 elongate denticles outside the primary, triangular cusp. The remaining lateral teeth are hook-shaped and are devoid of denticles (Fig. 20D, F).

###### Reproductive system

The reproductive organ arrangement is androdiaulic. The elongate hermaphroditic duct leads into the thick, tubular ampulla (Fig. 21). The convoluted ampulla bifurcates near the centre of the female gland mass into the short oviduct and the short, convoluted, wide prostate. The prostate expands slightly into a tubular penial sheath that is shorter than the prostate. The penis is curved with a subacute apex. The round bursa copulatrix is large, and comparable in size to the ampulla. From the bursa, the narrow vaginal duct extends into the equally narrow vagina, which exits into the genital atrium next to the penial sheath.

###### Remarks

We do not include a photo of this species as the material examined had been collected many years ago and did not include photographs. This species was traditionally misidentified as *D. striatus* (Hasselt, 1824), with which it is sympatric in Japanese waters (Eliot, 1913; Baba, 1937, 1949, 1976). Baba recognized this error in 1992 and described *D. otome* as a distinct species. Externally, the two species are quite different. Most of the body of *D. striatus* (Fig. 15I) is covered with brown pigment whereas *D. otome* is generally white with a brown patch on the notum and a few scattered dark brown spots surrounded by translucent rings of white (Rudman, 2003). Additionally, *D. otome* has bright red orange rhinophores whereas *D. striatus* has brown rhinophores with an opaque white apex. It also has an orange marginal band on the notum and oral veil that is absent in *D. otome*. *Dermatobranchus otome* is a member of the same species complex as *D. striatus, D. albus, D. diagonalis*, and *D. oculus.* All of these taxa that have a pectinate first lateral tooth and the remaining lateral teeth are hook-shaped and devoid of denticles. The rodlets of the marginal sacs are similar in shape to those described by Bergh (1888: pl. 10, fig. 6) for *D. fortunatus*.

**Figure 28:**
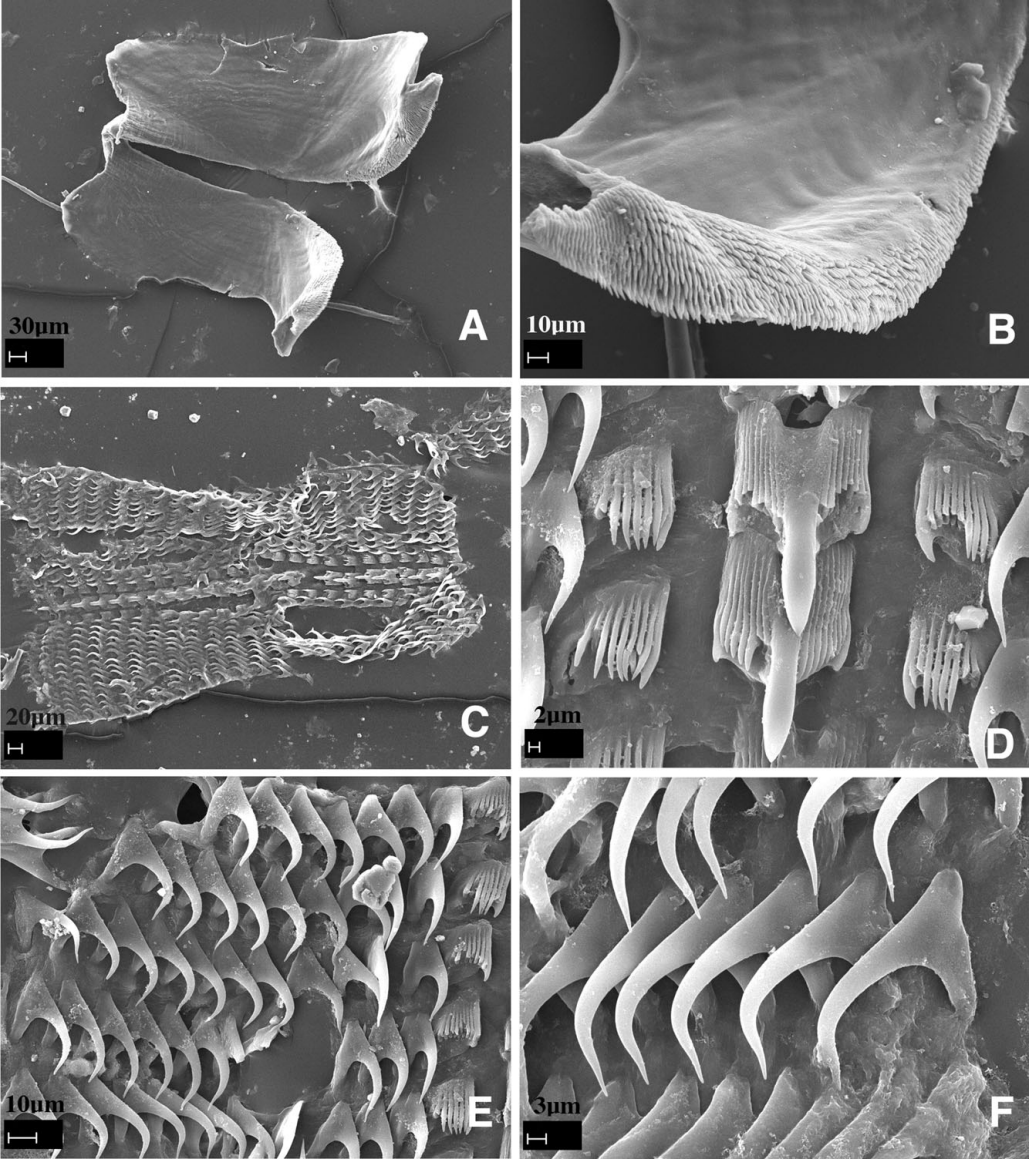
*Dermatobranchus striatus* Hasselt, 1824. Buccal armature, CASIZ 086314, Madang, Papua New Guinea. A, whole radula; B, central portion of radula; C, rachidian tooth; D, apical portion of rachidian tooth showing central cusp with denticles; E, middle lateral teeth; F, outer lateral teeth.

**Figure 47:**
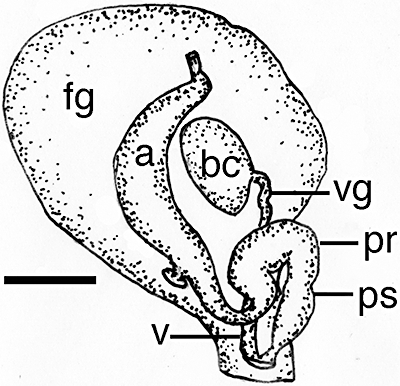
***Dermatobranchus diagonalis* sp. nov.** Reproductive system, CASIZ 070451, Rempi Lagoon, Madang, Papua New Guinea. a, ampulla; bc, bursa copulatrix; fg, female gland mass; pr, prostate; ps, penial sheath; v, vagina; vd, vaginal duct. Scale bar = 0.5 mm.

**Figure 68:**
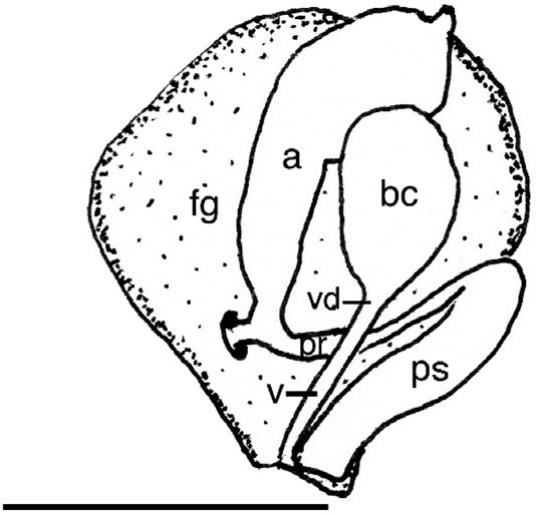
***Dermatobranchus oculus* sp. nov.** Reproductive system, CASIZ 079270, Okinawa, Japan. a, ampulla; bc, bursa copulatrix; fg, female gland mass; pr, prostate; ps, penial sheath; v, vagina; vd, vaginal duct. Scale bar = 1.2 mm.

The reproductive system of *D. otome* has not been described previously. The reproductive system is similar to other species of the *D. striatus* complex. *Dermatobranchus otome* has a relatively narrow penis (Fig. 21), whereas that of *D. albus* (Fig. 11), *D. diagonalis* (Fig. 47), *D. oculus* (Fig. 68) and *D. striatus* (Fig. 28) is more bulbous, and the penis is much wider than the adjacent prostate.

### *Dermatobranchus pustulosus* Hasselt, 1824 (Figs 15E–G, 22–24)

*Dermatobranchus pustulosus* Hasselt, 1824: 243.

*Pleuroleura pustulosa* (Hasselt): 357: pl. 11, figures 10–17.

*Dermatobranchus* sp. Coleman, 2008: 121, as tuberculed *Dermatobranchus*.

*Dermatobranchus pustulosus* Gosliner, Behrens & Valdés, 2008:308, top photo.

#### 

##### 

###### Material examined

CASIZ 069306, one specimen, 27 mm preserved, Arthur's Rock, Calumpan Peninsula, Batangas Province, Luzon Island, Philippine Islands, 18 m depth, collected 24.ii.1995 by T. Gosliner. CASIZ 157212, two specimens, 18–20 mm preserved, Arthur's Rock, Calumpan Peninsula, Batangas Province, Luzon Island, Philippine Islands, no depth recorded, collected 11.v.2001 by T. Gosliner. CASIZ 083865, two specimens, 17–21 mm preserved, Twin Rocks, Calumpan Peninsula, Batangas Province, Luzon, Philippines, no depth recorded, collected 26.ii.1992 by T. Gosliner. CASIZ 085963, three specimens, 20–25 mm preserved, Twin Rocks, Calumpan Peninsula, Batangas Province, Luzon, Philippines, 12 m depth, collected 26.iii.1993 by M. Miller. CASIZ 096293, one specimen, 20 mm preserved, Twin Rocks, Calumpan Peninsula, Batangas Province, Luzon, Philippines, 10 m depth, collected 14.iii.1994 by T. Gosliner. CASIZ 083798, one specimen, dissected, 20 mm preserved, Arthur's Rock, Calumpan Peninsula, Batangas Province, Luzon Island, Philippine Islands, 15 m depth, collected 22.ii.1992 by T. Gosliner. CASIZ 085904, one specimen, 63 mm preserved, Liuay Rock, Dakak Region, Mindanao Island, Philippine Islands, 16 m depth, collected 29.iii.1993 by M. Miller.

###### Geographical distribution

This species is known from the Philippine Islands, Solomon Islands, Japan, and Indonesia (present study), and Papua New Guinea (Coleman, 2008).

###### External morphology

The body shape of the living animal (Fig. 15E–G) is elongate, but stocky, slightly flattened, and narrows at the posterior end. The foot does not project beyond the distinct mantle margin. The dorsum has very low, broken longitudinal ridges and tubercles that give a lumpy appearance to the notum. The oral veil barely projects at the anterior end of the body. The rhinophores are behind the oral veil. They have a series of longitudinal lamellae on the rounded club. The stalk does not narrow noticeably and the club has a small projecting tip. Marginal sacs are readily visible along the mantle edge.

There are no hyponotal lamellae under the mantle margin. The genital opening is situated in the anterior quarter of the body. The anus is situated approximately half of the way to the posterior end of the body.

The ground colour of the dorsum and the foot is dominated by opaque white pigment, but there are areas of distinct pale blue or lavender, giving the overall appearance of marble. On the mantle, there are several distinct areas of mixed blue and tan pigment. There are two noticeable dark lines of pigment that divide the dorsum into approximate thirds. The dorsal ridges are mostly tan with some dark spots of colour randomly sprinkled. The depressions between the ridges are mixed white and pale blue. The rhinophore stalk is white with darker bands of tan and the club is entirely tan. The tip of the rhinophores is white with a dark band beneath the white. The oral veil is opaque white with dark spots along the margin. Dark speckling remains visible on the entire ventral surface of the preserved specimens.

###### Buccal armature

**Figure 22:**
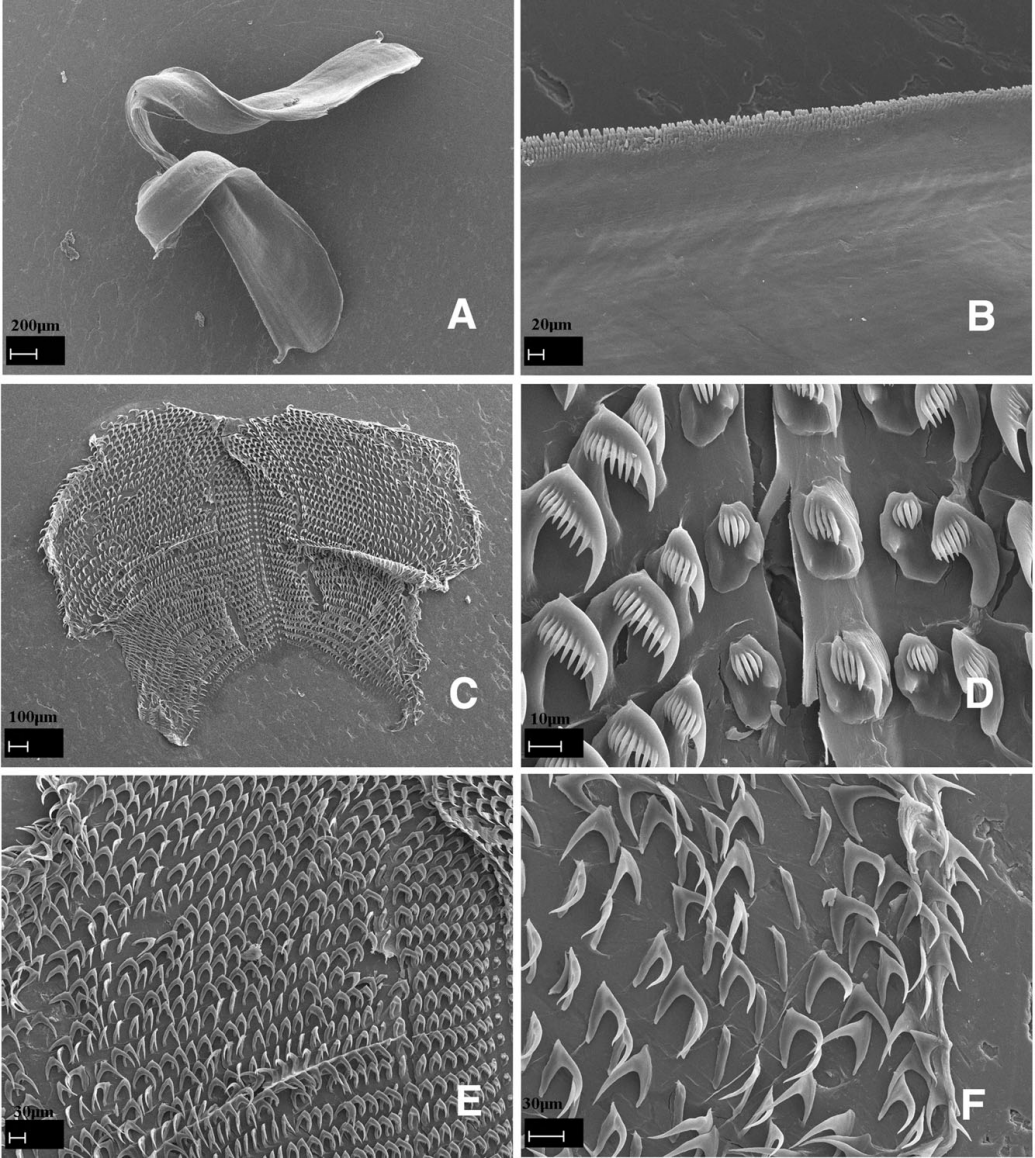
*Dermatobranchus pustulosus* Hasselt, 1824. Buccal armature, CASIZ 085963, Mabini, Luzon, Philippines. A, jaws; B, masticatory margin; C, whole radula; D, central portion of radula; E, F, outer lateral teeth.

**Figure 23:**
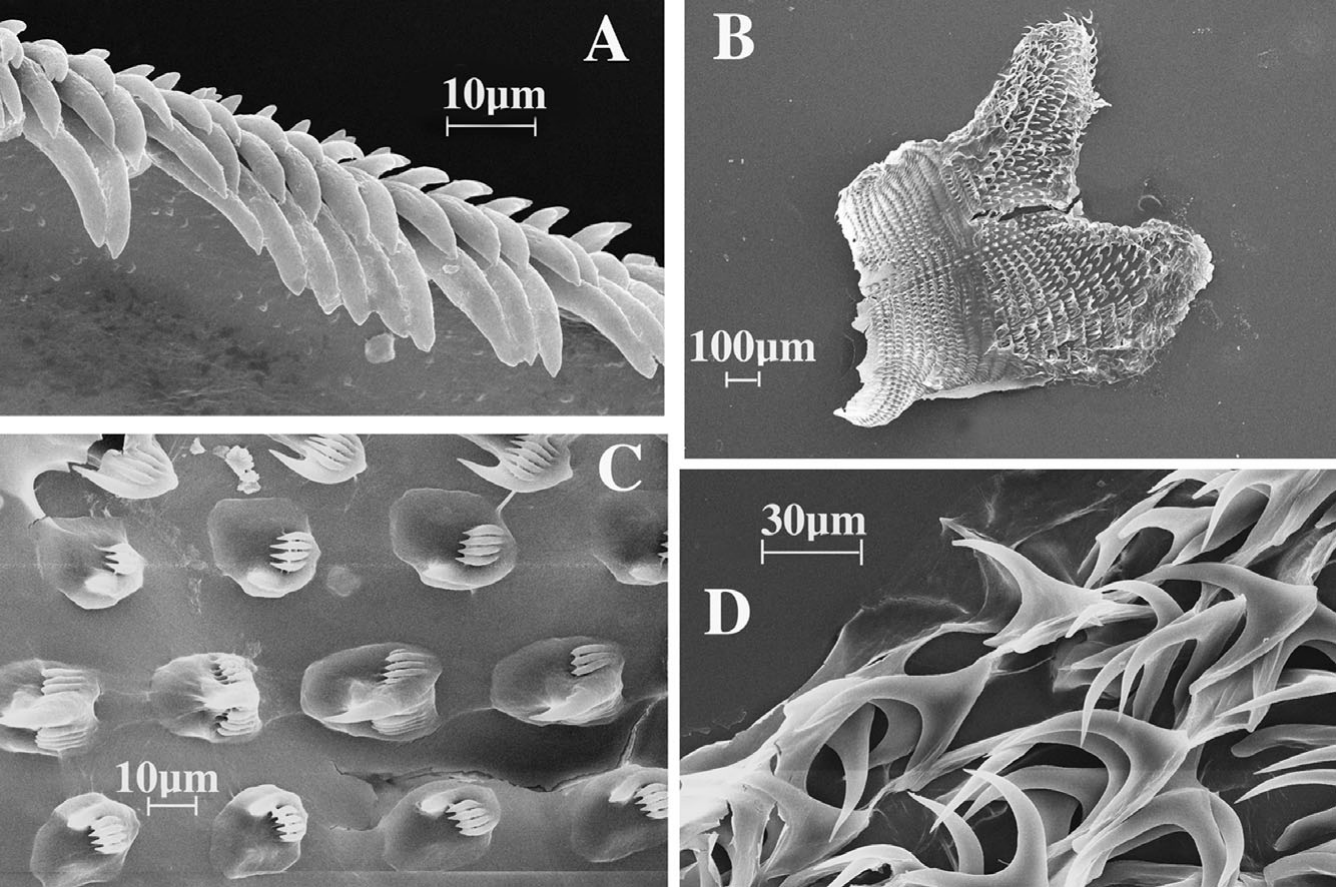
*Dermatobranchus pustulosus* Hasselt, 1824. Buccal armature, CASIZ 083798, Mabini, Luzon, Philippines. A, masticatory margin; B, whole radula; C, central portion of radula; D, outer lateral teeth.

The jaws are large and thickly cuticularized (Fig. 22A), with a thick masticatory margin and three to four rows of undivided, blunt tipped denticles (Figs 22B, 23A). The radular formula of two specimens (CASIZ 085963) is 39 × 44.1.1.1.44 (Fig. 22C) and (CASIZ 083798) 27 × 32.1.1.1.32 (Fig. 23B). The rachidian teeth (Figs 22D, 23C) have a narrow base with a projecting claw that has a thick, long central cusp. The central cusp is wider than the three to five shorter flanking denticles on each side. The innermost lateral tooth (Figs 22D, 23C) is claw-shaped with a relatively narrow base. Its central cusp is situated below the level of the adjacent denticles. It has three to five pointed denticles similar in length that make up the claw. The next approximately 33 lateral teeth (Fig. 22E) look like combs with handles. That is, the base is broad, which then narrows at the comb. The comb has a long central cusp and five to nine shorter, pointed denticles. The outer approximately 12 lateral teeth are long sharp hooks without denticles (Figs 22F, 23D).

###### Reproductive system

**Figure 24:**
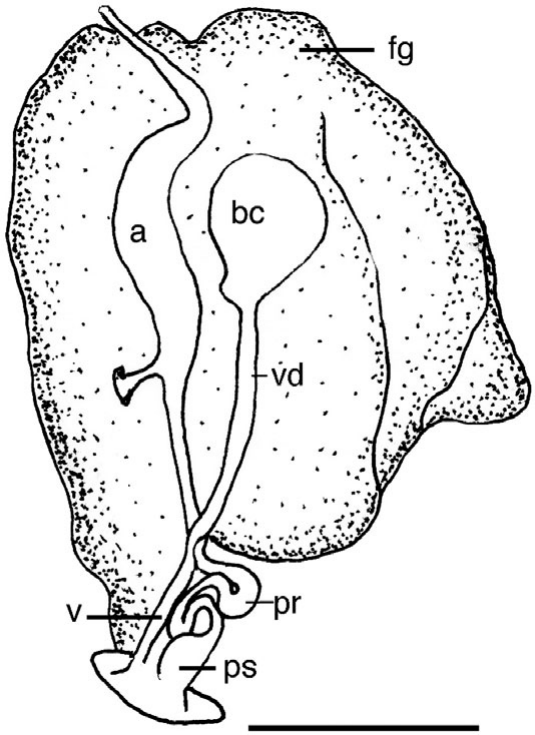
*Dermatobranchus pustulosus* Hasselt, 1824. Reproductive system, CASIZ 085963, Mabini, Luzon, Philippines. a, ampulla; bc, bursa copulatrix; fg, female gland mass; pr, prostate; ps, penial sheath; v, vagina; vd, vaginal duct. Scale bar = 2.3 mm.

The reproductive organ arrangement is androdiaulic. The long, thin hermaphroditic duct leads into the elongate, tubular ampulla (Fig. 24). The ampulla bifurcates into the female gland mass and into the elongate, thin prostate. The prostate coils back onto itself twice then expands into the short, bell-shaped penial sheath. From the round bursa copulatrix, the long, narrow vaginal duct extends into the equally narrow vagina, which exits into the genital atrium next to the penial sheath.

###### Remarks

The original description of *D. pustulosus* (van Hasselt, 1824) was very brief, limited to external features (Fig. 15E). Bergh (1887, 1888) provided details and drawings of the jaws and radula. The specimens examined for the present study match Bergh's descriptions in every respect, particularly in the denticulation of the radular teeth.

**Figure 42:**
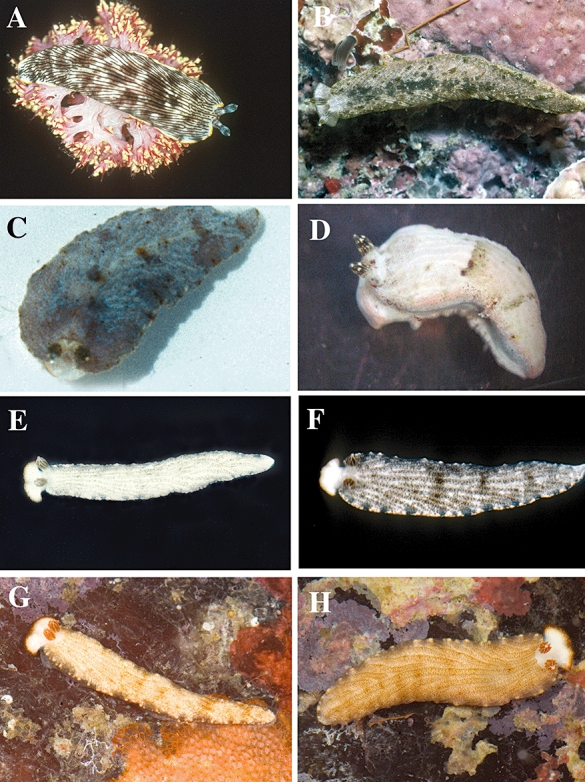
Living animals. A, ***Dermatobranchus dendronephthyphagus* sp. nov.**, living animal on prey, Okinawa, Ryukyu Islands, photo by Bob Bolland. B, ***Dermatobranchus diagonalis* sp. nov.**, CASIZ 068708, Rempi Lagoon, Papua New Guinea, photo T. M. Gosliner. C, ***Dermatobranchus earlei* sp. nov.**, photo by John Earle. D, ***Dermatobranchus fasciatus* sp. nov.**, Panglao, Bohol, Philippines, photo by Marina Poddubetskaia. E, F, ***Dermatobranchus funiculus* sp. nov.** living animals, Okinawa, Ryukyu Islands, photos by Bob Bolland. G, H, ***Dermatobranchus funiculus* sp. nov.** living animals, Maricaban Island, Luzon, Philippines, photos by T. Gosliner.

**Figure 58:**
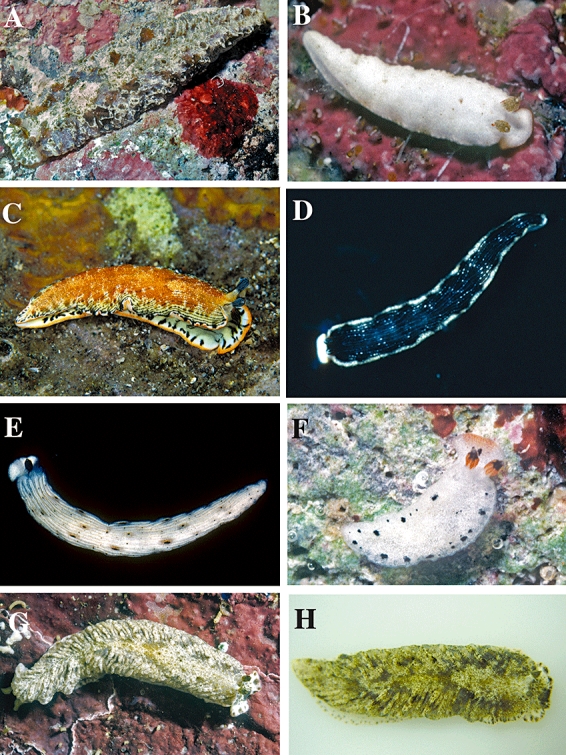
Living animals. A, ***Dermatobranchus kalyptos* sp. nov.**, Lombok Island, Indonesia, photo by Pauline Fiene. B, ***Dermatobranchus kokonas* sp. nov.**, CASIZ 075272, Bagabag Island, Papua New Guinea, photo by T. M. Gosliner. C, ***Dermatobranchus leoni* sp. nov.**, CASIZ 167455, Mabini Luzon, Philippines, photo by Constantinos Petrinos. D, ***Dermatobranchus microphallus* sp. nov.**, Flores, Indonesia, photo by Pauline Fiene. E, ***Dermatobranchus oculus* sp. nov.**, Okinawa, Japan, photo by Bob Bolland. F, ***Dermatobranchus piperoides* sp. nov.**, CASIZ 068708, Rempi Lagoon, Papua New Guinea, photo by T. M. Gosliner. G, ***Dermatobranchus phyllodes* sp. nov.**, CASIZ 070451, Bagabag Island, Papua New Guinea, photo by T. M. Gosliner. H, ***Dermatobranchus phyllodes* sp. nov.**, CASIZ 173351, Bohol, Philippines, photo by Marina Poddubetskaia.

**Figure 74:**
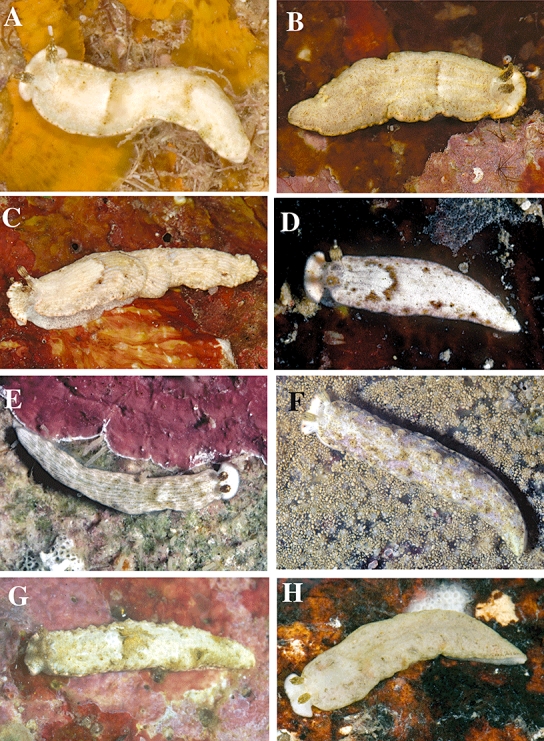
Living animals. A, ***Dermatobranchus rodmani* sp. nov.**, CASIZ 173400, Radama Islands, Madagascar, photo by T. M. Gosliner. B, ***Dermatobranchus rodmani* sp. nov.**, CASIZ 174170, Pulau Labus, Malaysia, photo by D. W. Behrens. C, ***Dermatobranchus semilunus* sp. nov.**, Pulau Chimbe, off Tioman, Malaysia, photo by T. M. Gosliner. D, ***Dermatobranchus semilunus* sp. nov.**, CASIZ 073045, Madang, Papua New Guinea, photo by T. M. Gosliner. E, ***Dermatobranchus semilunus* sp. nov.**, CASIZ 110407, Cabilao, Bohol, Philippines, photo by T. M. Gosliner. F, ***Dermatobranchus tuberculatus* sp. nov.**, CASIZ 110361, Ligpo Island, Luzon, Philippines, photo by T. M. Gosliner. G, ***Dermatobranchus tuberculatus* sp. nov.**, CASIZ 096332, Devil's Point, Maricaban Island, Luzon, Philippines, photo by T. M. Gosliner. H, ***Dermatobranchus tuberculatus* sp. nov.**, CASIZ 174171, Pulau Tenggol, Malaysia, photo by T. M. Gosliner.

Externally, *D. pustulosus* (Fig. 15E–G) has some similarities to *D. albus* (Fig. 1C–F), *D. semilunus* (CASIZ 071239) (Fig. 74C–E), *D. kalyptos* (Fig. 58A), *D. fasciatus* (Fig. 42D), and *D. tuberculatus* (Fig. 74F–H). The body shape of *D. pustulosus* is much stockier than the other species. All the other species basically have white ground colour, noticeable dark bands of colour crossing the dorsum, and darker coloured rhinophore clubs, whereas in *D. pustulosus* the rhinophore club is lighter brown and more bulbous in shape. However, both *D. pustulosus* and *D. kalyptos* have a more complex body colour composed of multiple pigments. Both *D. pustulosus* and *D. kalyptos* have blue or light lavender pigment with large brown blotches along the side of the mantle. Neither of the other two species have a complex colour pattern. The rhinophore clubs of *D. pustulosus* are light tan, whereas *D. kalyptos* has brown clubs and the other two species have black clubs.

**Figure 59:**
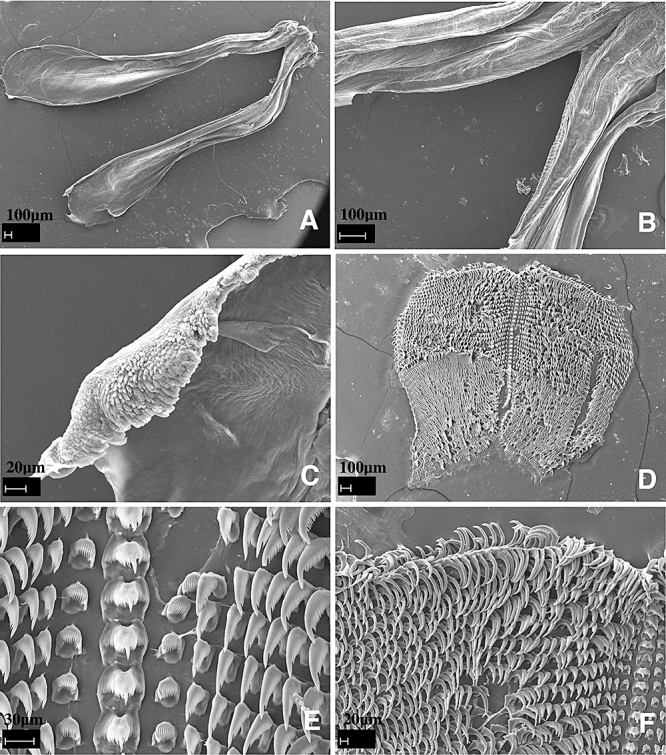
***Dermatobranchus kalyptos* sp. nov.** Buccal armature, CASIZ 107424, Lombok, Indonesia. A, jaws; B, masticatory margin; C, detail of masticatory denticles; D, entire radula; E, central portion of radula; F, middle and outer lateral teeth.

There are distinct radular differences amongst these species. Both *D. pustulosus* and *D. kalyptos* have a very complex radular formula with similar teeth on a broad radula (27–39 × 32–44.1.1.1. 32–44 and 41 × 57.1.1.1.57, respectively). However, the radular tooth morphology differs between these two. The rachidian tooth of *D. pustulosus* (Figs 22D, 23C) has three to six flanking denticles and a very long central cusp, whereas that of *D. kalyptos* (Fig. 59E) has 13–14 denticles and a shorter cusp as compared to the flanking denticles. The first lateral teeth also differ between species. *Dermatobranchus pustulosus* has only three to five comparable denticles making up a ‘claw’, whereas the first lateral teeth of *D. kalyptos* has 13–14 denticles, with a longer primary denticle. The jaws of these two species differ in the number of rows of rods. There are four rows of blunt denticles in *D. pustulosus* (Figs 22B, 23A) whereas *D. kalyptos* (Fig. 59C) has at least 12 rows of blunt denticles.

The radula of *D. albus* also has four rows of blunt denticles, but the rachidian tooth is very different and the formula is 21–28 × 7–10.1.1.1.7–10. In addition, only the first lateral tooth is denticulate whereas all others are smooth. The jaw of *D. semilunus* (CASIZ 071239) also has multiple rows of denticles, but they are all pointed. The radular formula is 23–40 × 29–73.1.1.1.29–73 and the rachidian tooth has 17 flanking denticles alongside a very long pointed central cusp. The first lateral tooth has one, long first denticle and up to nine shorter, pointed denticles. Thus, with regard to tooth morphology, this species does not match any of the compared species.

**Figure 60:**
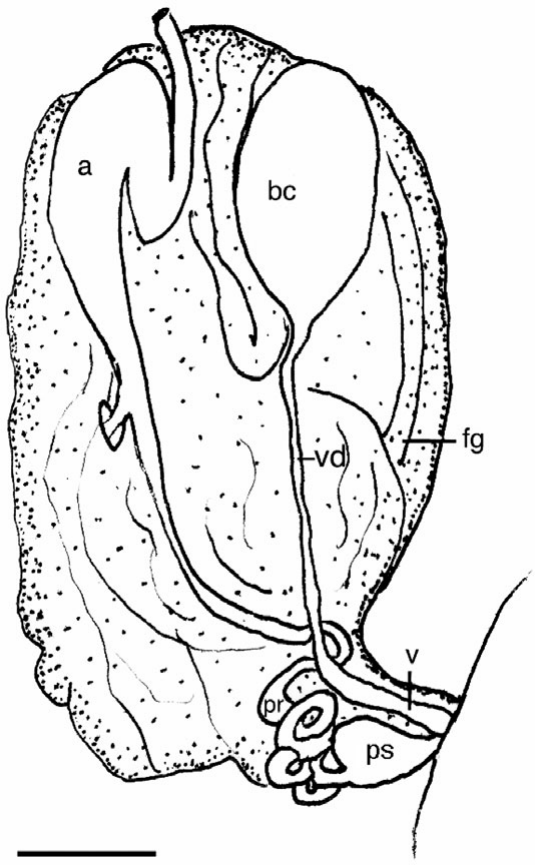
***Dermatobranchus kalyptos* sp. nov.** Reproductive system, CASIZ 107424, Lombok, Indonesia. a, ampulla; bc, bursa copulatrix; fg, female gland mass; pr, prostate; ps, penial sheath; v, vagina; vd, vaginal duct. Scale bar = 3.33 mm.

**Figure 81:**
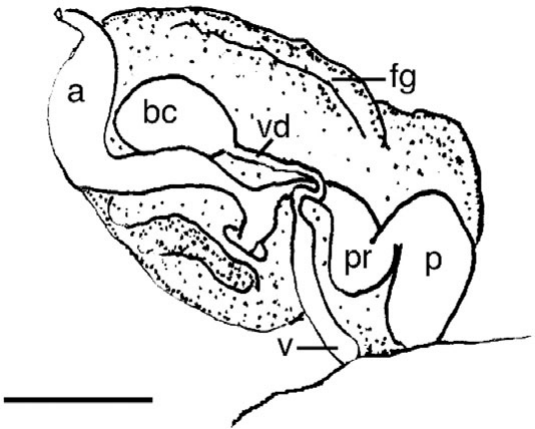
***Dermatobranchus semilunus* sp. nov.** Reproductive system, CASIZ 107424, Lombok, Indonesia. a, ampulla; bc, bursa copulatrix; fg, female gland mass; p, penis; pr, prostate; v, vagina; vd, vaginal duct. Scale bar = 1.0 mm.

**Figure 85:**
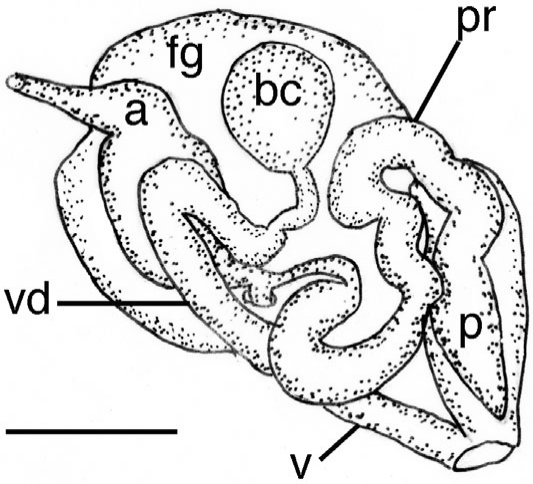
***Dermatobranchus tuberculatus* sp. nov.** Reproductive system, CASIZ 173400, Ligpo Island, Balayan Bay, Luzon, Philippines. a, ampulla; bc, bursa copulatrix; fg, female gland mass; p, penis; pr, prostate; v, vagina; vd, vaginal duct. Scale bar = 0.2 mm.

The reproductive systems of the five species compared here have some similarities. All species have an expanded or bulbous penis sheath and a long vaginal duct and vagina. The ampulla of all species is tubular. Several features distinguish these species. *Dermatobranchus pustulosus* (Fig. 24) has a very short, bell-shaped penis sheath and a twice-coiled prostate. *Dermatobranchus kalyptos* (Fig. 60) has a bell-shaped penis sheath and a very long, coiled, narrow prostate, a feature not shared by the other three species. The penis sheath of *D. semilunus* (Fig. 81) is very bulbous, long, and s-shaped, with a very short prostate. The penis sheath of *D. albus* (Fig. 11) is bulbous, nearly straight, and the prostate is short with no twists. In *D. tuberculatus* (Fig. 85), the prostate is elongate and convoluted and the penial papilla is short and conical.

### *Dermatobranchus rubidus* (Gould, 1852) (Figs 15H, 25–27)

*Diphyllidia rubida* Gould, 1852: 307, figures 406, 406a.

*Dermatobranchus rubidus* (Gould, 1852) Kay, 1979: 480: figure 154b.

*Dermatobranchus pulcherrimus* Miller & Willan, 1986: 384, figures 4, 5. **syn. nov.**

*Armina japonica* (Eliot, 1913) Suzuki, 2000: 133, no. 200, misidentification.

*Dermatobranchus rubidus* Gosliner, Behrens & Valdés, 2008:309, second photo.

#### 

##### 

###### Material examined

CASIZ 105696, one specimen, 26 mm preserved, Seafari, Batangas, Luzon, Philippine Islands, 7 m depth, collected 22.ii.1995 by T. Gosliner. CASIZ 116909, one specimen, dissected, Kihei, Maui, Hawaii, intertidal zone, collected 1.viii.1979 by S. Jazwinski.

###### Geographical distribution

This species is known from New Zealand, South Australia (Miller & Willan, 1986), the Philippine Islands (present study), Japan (Suzuki, 2000), and Hawaii (Gould,1852; Kay, 1979; present study).

###### External morphology

The body shape of the living animal (Fig. 15H) is broad, flattened, and narrows at the posterior end. The wide foot projects beyond the distinct mantle margin and the anterior foot corners extend in posterior-angled points. There are approximately five longitudinal dorsal ridges on each side of a larger median ridge. The distinct anvil-shaped oral veil extends well forward and laterally and the outside corners are elongate. Behind the oral veil are the closely spaced rhinophores. The rhinophores have a series of longitudinal lamellae on the rounded club. The stalk does not narrow and there are no lamellae on it. No marginal sacs are visible and there are no branchial lamellae or hyponotal lamellae. The anal opening is situated approximately half way along the body side and the genital opening is at the anterior third of the body wall.

The ground colour of the dorsum and foot is translucent red and the dorsal ridges are opaque white. The mantle, the oral veil, and the foot have a distinct white edge. The rhinophores are red.

###### Buccal armature

**Figure 25:**
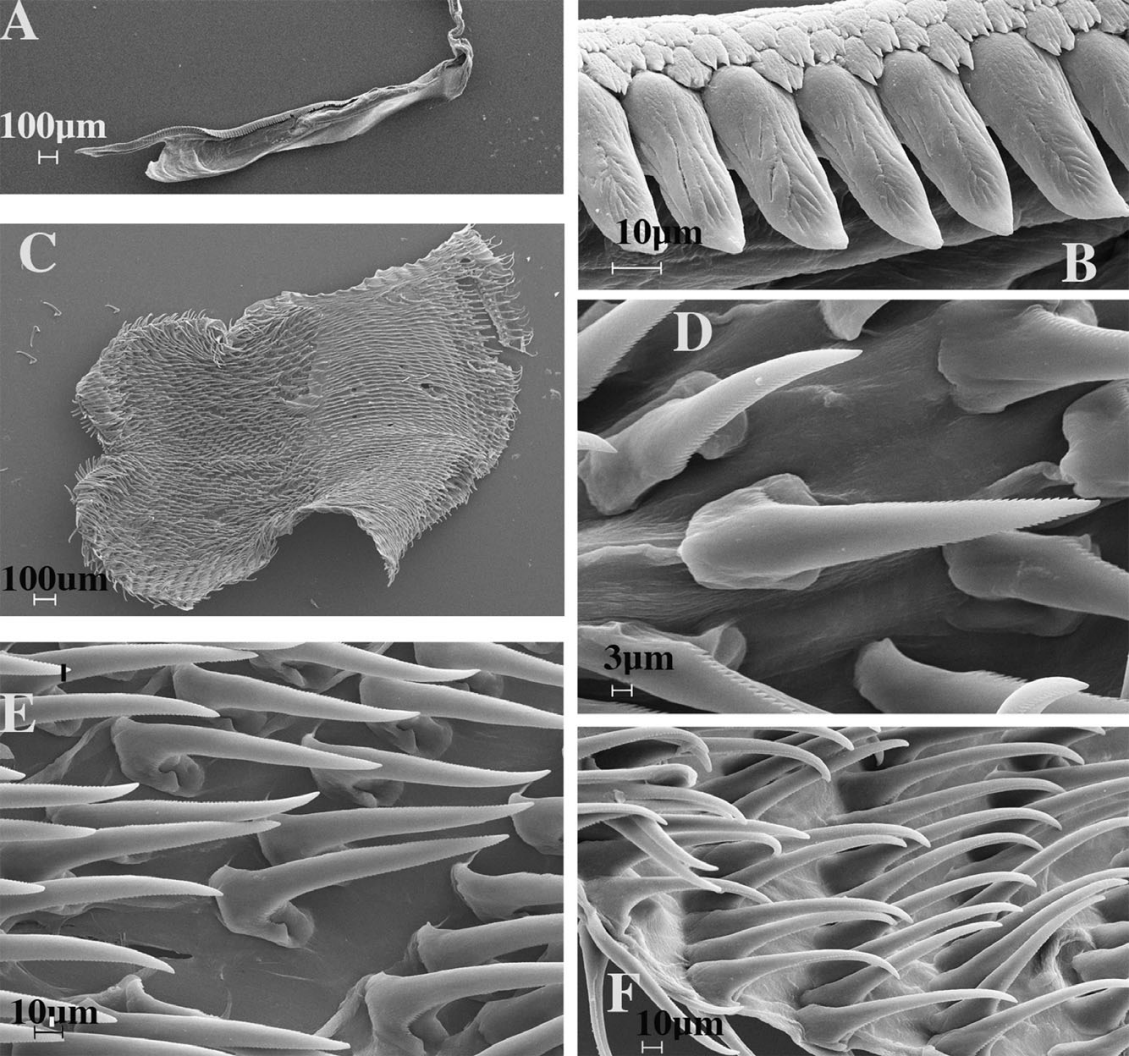
*Dermatobranchus rubidus* (Gould, 1852). Buccal armature, CASIZ 116909, Mabini, Luzon, Philippines. A, jaw; B, masticatory margin; C, whole radula; D, central portion of radula; E, middle lateral teeth; F, outer lateral teeth.

**Figure 26:**
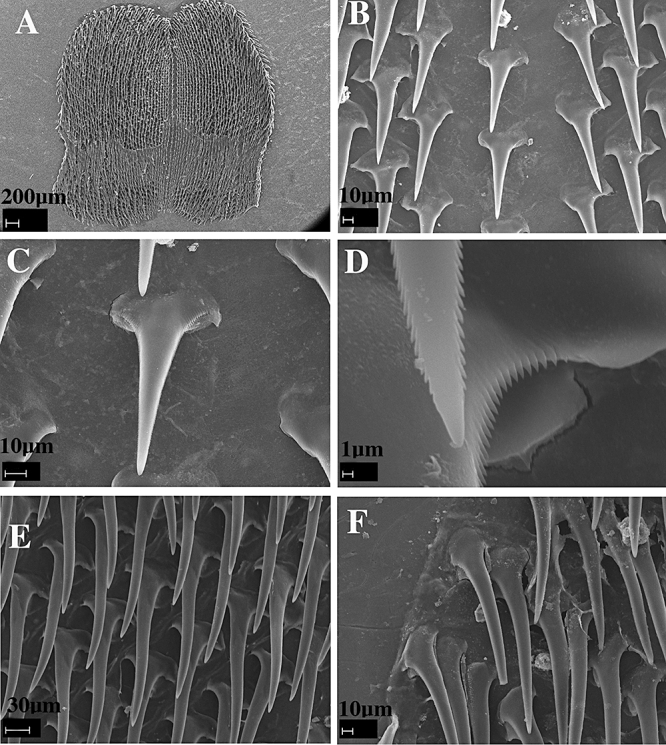
*Dermatobranchus rubidus* (Gould, 1852). Buccal armature, CASIZ 105696, Oahu, Hawai'i. A, whole radula; B, central portion of radula; C, rachidian tooth; D, apical portion of rachidian tooth showing central cusp with denticles; E, middle lateral teeth; F, outer lateral teeth.

The jaws are large, thickly cuticularized (Fig. 25A), with a thick masticatory margin and one row of long, pointed denticles (Fig. 25B). There are three rows of multi-tipped, scale-like denticles lying along the base of the single row of long denticles (Fig. 25B). The radula is almost as broad as it is long (Figs 25C, 26A). The radula formula of CASIZ 116909 is 48 × 51.1.51 (Figs 25C, 26A) and of CASIZ 105696 is 50 × 48.1.48. The rachidian teeth (Figs 25D, 26 B–D) are narrow spears, with about 70 minute denticles along both edges from the base to the point. The next 46–50 or more lateral teeth (Figs 25E, 26E) are elongate spears with approximately 40–70 tiny denticles along either edge. Towards the outer radula (Figs 25F, 26F), the lateral teeth become gradually more elongated hooks, but all have tiny denticles on the edges.

###### Reproductive system

**Figure 27:**
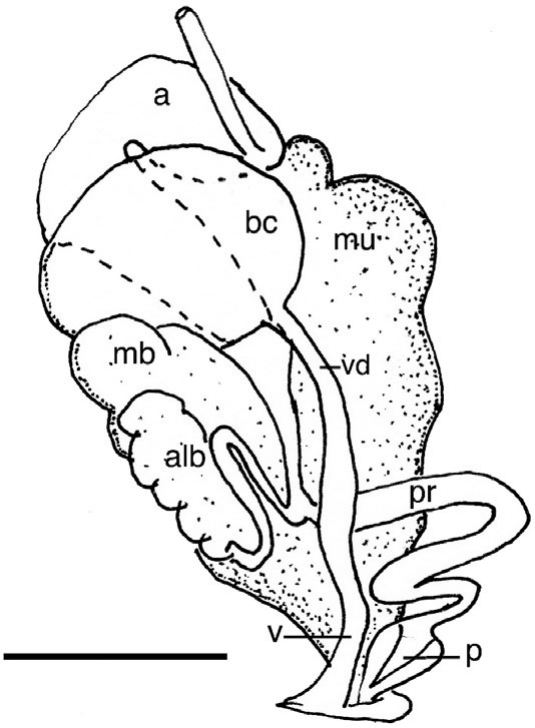
*Dermatobranchus rubidus* (Gould, 1852). Reproductive system, CASIZ 116909, Mabini, Luzon, Philippines. a, ampulla; alb, albumen gland; bc, bursa copulatrix; mb, membrane gland; mu, mucus gland; p, penis; pr, prostate; v, vagina; vd, vaginal duct, scale = 2.0 mm.

The reproductive organ arrangement is androdiaulic. The elongate hermaphroditic duct leads into the thick, tubular, curved ampulla (Fig. 27). The ampulla bifurcates near the posterior end of the female gland mass into the short oviduct and the long prostate. The prostate widens into the bulbous penial sheath. The round bursa copulatrix is large, and from it the long, narrow vaginal duct extends into the narrow vagina, which exits into the genital atrium next to the penial sheath.

###### Remarks

Miller & Willan (1986) described *D. pulcherrimus* from New Zealand and southern Australia. The external morphology and coloration of their specimens bear some resemblance to Gould's (1852) description of *D. rubidus* from Hawaii. Both Gould's drawing of *D. rubidus* and Miller & Willan's description indicate that the dorsal furrows are dark. Gould's external colour description was rather brief, limited to the foot and dorsal colour, along with a drawing showing black longitudinal ridges. The ridge colour remaining on the preserved specimen that we examined is white, not black.

Gould (1852) did describe *D. rubidus* as having an elongate oral ‘hood’, the posterior extremity tapering to a fine point, and ‘an expanded mantle’ that ‘turns up on each side, but not sufficiently to cover the back’. This last feature may be interpreted to mean anterior lateral extensions of the foot, all characteristics shared by *D. pulcherrimus*. The specimen that we examined from Hawaii (CASIZ 116909) was poorly preserved but did indeed have the distinctive tentacular extensions of the foot that are characteristic of *D. pulcherrimus*. Its radular morphology and reproductive system are also consistent with Miller & Willan's (1986) description and the specimen examined here from the Philippines (CASIZ 105696). The only minor difference noted is that the Hawaiian specimen had a slightly wider base to the rachidian teeth.

With regard to the external morphology, the specimen of *D. rubidus* examined for the present study matches both Miller & Willan's (1986) description of *D. pulcherrimus* and Gould's (1852) description of *D. rubidus*. However, the background colour of the specimen we examined from the Philippine Islands is red as described by Gould, whereas Miller & Willan's specimens were burnt sienna in colour. Our specimen also has the same radular morphology including a similar radular formula as Miller & Willan's specimens from New Zealand (37 × 46.1.1.46) compared to 48–50 × 48–51.1.48–51 for our two specimens.

It appears that *D. pulcherrimus* and *D. rubidus* are conspecific and that the background colour ranges from red to sienna. Although Gould (1852) provided no description or drawings of either the radula or reproductive system of *D. rubidus*, the examination of a specimen from Hawaii (the type locality of *D. rubidus*) confirms that *D. rubidus* and *D. pulcherrimus* are virtually indistinguishable and should be considered as synonyms. Gould's (1852) name *D. rubidus* takes precedence over *D. pulcherrimus* (1986).

Baba (1949) illustrated the radula of *D. sagamianus* (text fig. 90) and *D. nigropunctatus* (text fig. 89). Both species have similarities in the radular morphology with *D. rubidus.* All species have a narrow, spear-shaped rachidian with tiny denticles along both edges and denticulate marginal teeth. However, in *D. sagamianus* and *D. nigropunctatus* the outermost lateral teeth are not denticulate as they are in *D. rubidus.* Neither of Baba's two species is externally similar to *D. rubidus.*

Lin (1981) described *D. marginlatus* from the South China Sea. This species is known only from preserved specimens and the colour of the living animal is unknown. This species has 14 longitudinal dorsal ridges compared to 11 found in *D. rubidus*. The anatomy of the radula with an elongate rachidian cusp bearing many small denticles is similar to that of *D. rubidus*. The reproductive system of this species was not described. More detailed comparison of *D. marginlatus* with *D. rubidus* is necessary once additional material from China becomes available to further establish the validity of the former species.

### *Dermatobranchus striatus* Hasselt, 1824 (Figs 15I, 28, 29)

*Dermatobranchus* s*triatus* Hasselt, 1824: 243.

*Pleuroleura striata* (Hasselt) Bergh, 1888: 353.

*Dermatobranchus striatus* Gosliner, Behrens & Valdés, 2008:309, top photo.

#### 

##### 

###### Material examined

CASIZ 170100, one specimen, 13 mm preserved, dissected, Iriomote Island, Okinawa, Japan, 15 m depth, collected 30.iv.2002 by M. Kasai. CASIZ 170101, one specimen, 20 mm preserved, dissected, Iriomote Island, Okinawa, Japan, 15 m depth, collected 30.iv.2002 by M. Kasai. CASIZ 086314, one specimen, 20 mm, dissected, Coral Queen, north of Madang, Papua New Guinea, 30 m depth, collected 16.vi.992 by M. Jebb. CASIZ 107423, five specimens, 28–31 mm alive, one 30 mm dissected, Kalabahi Bay, Alor Islands, Lesser Sunda Islands, Indonesia, 7 m depth, collected 19.xi.1995 by P. Fiene.

###### Geographical distribution

This species is known from Japan (Baba, 1992 and present study), Papua New Guinea (present study), and Indonesia (Hasselt, 1824 and present study).

###### External morphology

The specimens examined (Fig. 15I) in the present study match Baba's (1992) description of *D. striatus* specimens from Okinawa. Our specimens have the same main colour features presented by Baba: black-brown oral veil with transverse white lines and orange margin, a black-brown ground colour on dorsum, deep yellow apices on rhinophores, black-brown on clubs and colourless stalks, yellow edges of oral veil, mantle and foot and white body sides and foot sole.

###### Buccal armature

The jaws are large and thickly cuticularized (Fig. 28A), with a thick masticatory margin and multiple rows of triangular, pointed denticles (Fig. 28B). The radular formula of CASIZ 086314 is 24 × 11–12.1.1.1.11–12 (Fig. 28C) and that of CASIZ 170101 is 31 × 16.1.1.1.16. The rachidian teeth (Fig. 28D) have a broad base with a large, pointed central cusp that extends well beyond the eight to ten flanking denticles on each side. On the outer edge of each rachidian is a blunt ended cusp that is longer than the eight to ten denticles, but shorter than the central cusp. The first lateral tooth (Fig. 28D) is broad with a hook-shaped first denticle that extends beyond the following nine long, pointed denticles. The next 11–12 (CASIZ 086314) or 16 (CASIZ 170101) lateral teeth are elongate hooks with no denticles (Fig. 28E, F).

###### Reproductive system

**Figure 29:**
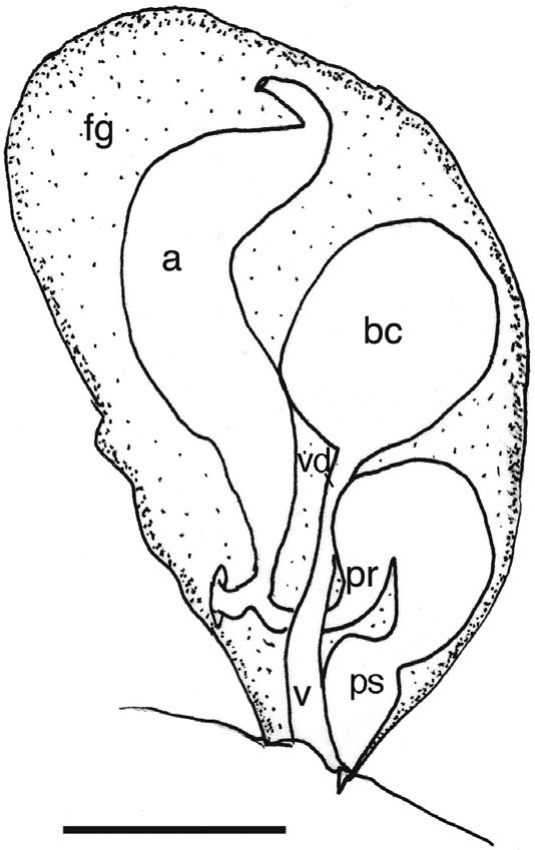
*Dermatobranchus striatus* Hasselt, 1824. Reproductive system, CASIZ 086314, Madang, Papua New Guinea. a, ampulla; bc, bursa copulatrix; fg, female gland mass; pr, prostate; ps, penial sheath; v, vagina; vd, vaginal duct. Scale bar = 0.55 mm.

The reproductive organ arrangement is androdiaulic. The elongate hermaphroditic duct leads into the thick, tubular ampulla (Fig. 29). The ampulla bifurcates near the centre of the female gland mass into the short oviduct and the short, narrow prostate. The prostate expands into the bulbous penial sheath that is as long as the prostate. The round bursa copulatrix is large, and comparable in size to the ampulla. From the bursa, the narrow vaginal duct extends into the equally narrow vagina, which exits into the genital atrium next to the penial sheath.

###### Remarks

Baba (1949, 1976) described specimens collected from Sagami and Tsuruga Bays as *D. striatus* van Hasselt, 1824. However, Baba (1992) then presented a critical review of this species, and compared the specimens collected for the 1976 study to other specimens collected from Okinawa. He concluded that his 1976 Suruga Bay specimens (and others he had identified as *D. striatus* in previous publications) were not *D. striatus* but in fact represented a new species, *D. otome* Baba, 1992. The main colour differences that Baba (1992) cited for *D. otome* are an orange-red rhinophore club, whitish tip, a black, semilunar band transverse across the anterior dorsum and ocellated spots arranged serially on the dorsal ridges.

There is one radular difference that Baba (1992) cited between the two species. He noted that *D. striatus* has denticles on the inner side of the first lateral tooth cusp, whereas *D. otome* lacks denticles. The specimens examined for the present study from Japan and Papua New Guinea also have denticles on the inner side of the first lateral tooth.

Descriptions and drawings are notably lacking for *D. striatus.* Baba (1992) did not describe the reproductive system of his specimens of *D. striatus* but he did present some characteristics of the reproductive system of *D. otome*. He noted and drew the ‘conical, unarmed penis’. The specimens examined for the present study had a bulbous penial sheath and no armament was noted.

### New species

### *Dermatobranchus albineus*sp. nov. (Figs 30A, B, 31, 32)

*Dermatobranchus* sp. 1 Gosliner, 1987:110, figure 211.

**Figure 30:**
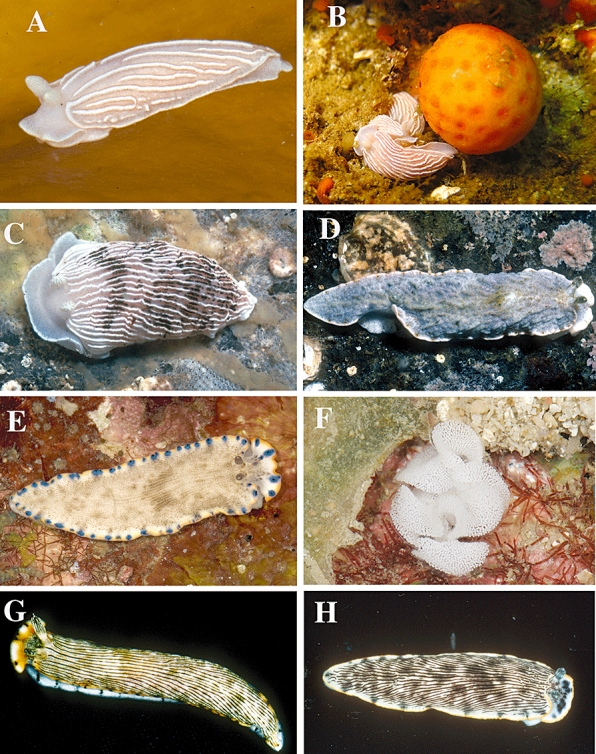
Living animals. A, ***Dermatobranchus albineus* sp. nov.**, Hottentot's Huisie, Cape Peninsula, South Africa, photo T. M. Gosliner. B, ***Dermatobranchus albineus* sp. nov.**, on host octocoral, Hottentot's Huisie, Cape Peninsula, South Africa, photo by T. M. Gosliner. C, ***Dermatobranchus arminus* sp. nov.**, Bakoven, South Africa, photo by T. M. Gosliner. D, ***Dermatobranchus caesitius* sp. nov.**, Umgazana, South Africa, photo by T. M. Gosliner. E, ***Dermatobranchus caeruleomaculatus* sp. nov.**, living animal, Tioman Island, Malaysia, photo by T. M. Gosliner. F, ***Dermatobranchus caeruloemaculatus* sp. nov.**, egg mass, Tioman Island, Malaysia, photo by T. M. Gosliner. G, ***Dermatobranchus cymatilis* sp. nov.**, Okinawa, Japan, photo by Bob Bolland. H, ***Dermatobranchus dendronephthyphagus* sp. nov.**, living animal, Okinawa, Ryukyu Islands, Japan, photo by Bob Bolland.

**Figure 31:**
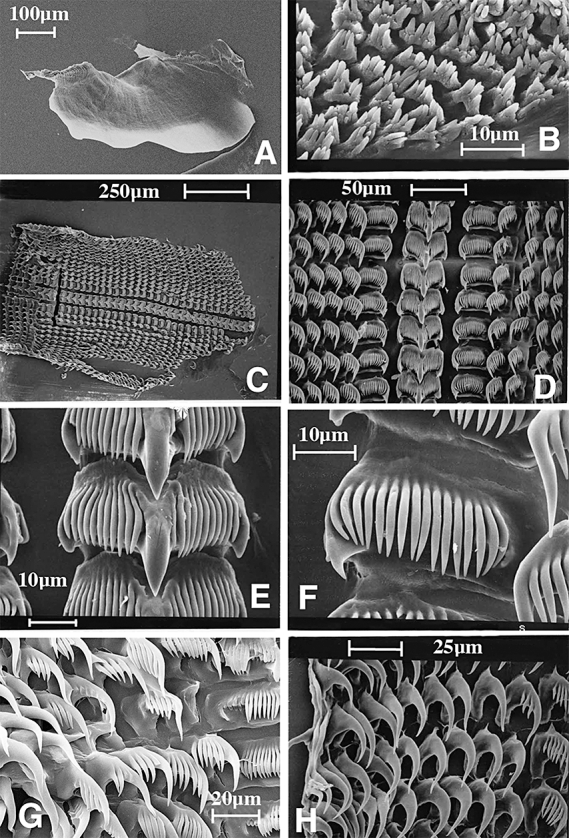
***Dermatobranchus albineus* sp. nov.** Buccal armature, SAM A357563, Llandudno, South Africa. A, jaw; B, masticatory margin; C, whole radula; D, central portion of radula; E, rachidian teeth; F, inner lateral teeth; G, middle lateral teeth; H, outer lateral teeth.

**Figure 32:**
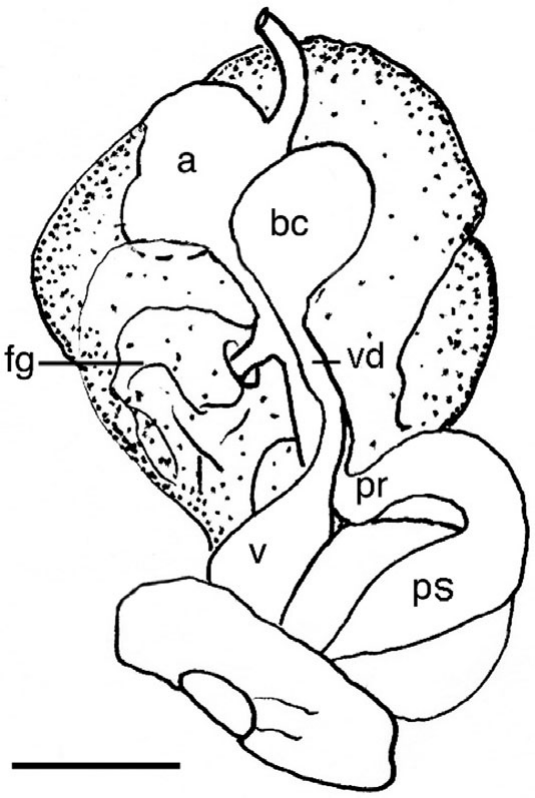
***Dermatobranchus albineus* sp. nov.** Reproductive system, CASIZ 073997, Llandudno Bay, Atlantic coast, Cape Peninsula. a, ampulla; bc, bursa copulatrix; fg, female gland mass; pr, prostate; ps, penial sheath; v, vagina; vd, vaginal duct. Scale bar = 0.63 mm.

#### 

##### 

###### Type material

Holotype: CASIZ 074050, 16 mm preserved, Hottentot Huisie, Atlantic coast, Cape Peninsula, Cape Province, South Africa (33°59.2407′S, 18°20.812244′E) 7 m depth, collected 5.xii.1980 by T. Gosliner. Paratypes. CASIZ 073997, two specimens, 11, 14 mm preserved, dissected, Llandudno Bay, Atlantic coast Cape Peninsula, Western Cape Province, South Africa, 7 m depth, collected 9.x.1982 by T. Gosliner. CASIZ 074008, five specimens, Philip's Reef, Algoa Bay, Port Elizabeth, Eastern Cape Province, South Africa, 11 m depth, collected 15.v.1984 by T. Gosliner. CASIZ 086855, two specimens, Hottentot Huisie, Atlantic coast, Cape Peninsula, Cape Province, South Africa, 7 m depth, date unknown, collected by T. Gosliner. CASIZ 086864, one specimen, Hottentot Huisie, Atlantic coast, Cape Peninsula, Cape Province, South Africa, 7 m depth, collected i.1981 by T. Gosliner. CASIZ 086855, one specimen, Bakoven, Atlantic coast, Cape Peninsula, Cape Province, South Africa, 7 m depth, collected 17.ix.1982 by T. Gosliner. CASIZ 176963, one specimen, Hottentot Huisie, Atlantic coast, Cape Peninsula, Cape Province, South Africa, 17.5 m depth, collected 14.i.2008 by Gosliner *et al.* CASIZ 176267, one specimen, Hottentot Huisie, Atlantic coast, Cape Peninsula, Cape Province, South Africa, 13.4 m depth, collected 5.i.2008 by Gosliner *et al.* CASIZ 176268, one specimen, Hottentot Huisie, Atlantic coast, Cape Peninsula, Cape Province, South Africa, 13.4 m depth, collected 5.i.2008 by Gosliner *et al.* CASIZ 176269, one specimen, Hottentot Huisie, Atlantic coast, Cape Peninsula, Cape Province, South Africa, 17.5 m depth, collected 5.i.2008 by Gosliner *et al.* CASIZ 176962, 1 specimen, Hottentot Huisie, Atlantic coast, Cape Peninsula, Cape Province, South Africa, 17.5 m depth, collected 14.i.2008 by Gosliner *et al.* CASIZ 176963, one specimen, Hottentot Huisie, Atlantic coast, Cape Peninsula, Cape Province, South Africa, 17.5 m depth, collected 14.i.2008 by Gosliner *et al.* CASIZ 176964, one specimen, Hottentot Huisie, Atlantic coast, Cape Peninsula, Cape Province, South Africa, 17.5 m depth, collected 14.i.2008 by Gosliner *et al.* CASIZ 176965, one specimen, Hottentot Huisie, Atlantic coast, Cape Peninsula, Cape Province, South Africa, 17.5 m depth, collected 14.i.2008 by Gosliner *et al.* CASIZ 176966, one specimen, Hottentot Huisie, Atlantic coast, Cape Peninsula, Cape Province, South Africa, 17.5 m depth, collected 14.i.2008 by Gosliner *et al.* CASIZ 176967, one specimen, Hottentot Huisie, Atlantic coast, Cape Peninsula, Cape Province, South Africa, 17.5 m depth, collected 14.i.2008 by Gosliner *et al.* CASIZ 176968, one specimen, Hottentot Huisie, Atlantic coast, Cape Peninsula, Cape Province, South Africa, 17.5 m depth, collected 14.i.2008 by Gosliner *et al.* CASIZ 176969, one specimen, Hottentot Huisie, Atlantic coast, Cape Peninsula, Cape Province, South Africa, 17.5 m depth, collected 14.i.2008 by Gosliner *et al.* CASIZ 176970, one specimen, Hottentot Huisie, Atlantic coast, Cape Peninsula, Cape Province, South Africa, 17.5 m depth, collected 14.i.2008 by Gosliner *et al.* CASIZ 176971, one specimen, Hottentot Huisie, Atlantic coast, Cape Peninsula, Cape Province, South Africa, 17.5 m depth, collected 14.i.2008 by Gosliner *et al.* SAM A35753, two specimens, one dissected, 12 and 15 mm preserved, Hottentot Huisie, Atlantic coast, Western Cape Province, South Africa, no depth recorded, collected 14.xii.1981 by T. Gosliner. SAM A35757, ten specimens, one dissected, 4–15 mm preserved, Hottentot Huisie, Atlantic coast, Western Cape Province, South Africa, no depth or date recorded collected by T. Gosliner. SAM A35751, two specimens, 5 and 7 mm preserved, Hottentot Huisie, Atlantic coast, Western Cape Province, South Africa, no depth recorded, collected 9.ix.1982 by T. Gosliner. SAM A35754, three specimens, 12, 13, and 15 mm preserved, Hottentot Huisie, Atlantic coast, Western Cape Province, South Africa, no depth recorded, collected ii.1981 by T. Gosliner. SAM A35728, seven specimens, 6–15 mm preserved, Llandudno and Hottentot Huisie, Atlantic coast, Western Cape Province, South Africa, no depth or date recorded, collected by T. Gosliner.

###### Geographical distribution

This species has only been reported from the Atlantic and Indian Ocean coasts of South Africa from Oudekraal to Algoa Bay (Gosliner, 1987; present study).

###### Natural history

This species is found in rocky habitats in relatively shallow water in cold temperate waters of the Cape of Good Hope region where it feeds (Fig. 30B) on the soft coral *Eleutherobia variabile* (Thomson, 1910) (Gosliner, 1987).

###### Etymology

The specific name *albineus* is a noun in apposition from a Latin word for ‘white’ to describe the whitish coloration of this *Dermatobranchus*.

###### External morphology

The body shape of the living animal (Fig. 30A, B) is elongate but broad, flattened, and narrows at the posterior end. The wide foot projects beyond the distinct mantle margin. There are approximately 16 longitudinal dorsal ridges with three shorter ridges between them on each side of the median towards the posterior. The distinct anvil-shaped oral veil extends well forward and laterally. Behind the oral veil are the closely spaced rhinophores. The rhinophores have a series of longitudinal lamellae on the rounded club. There are no lamellae on the stalk. Marginal sacs are readily visible on the mantle edge. There are no hyponotal or branchial lamellae. Approximately one-third along on the right side of the body is the genital opening. The anus is situated approximately half of the way along the body side.

The ground colour of the dorsum and oral veil is pinkish to greyish white. The dorsal ridges and the margin of the foot are opaque white. The rhinophores are creamy pale yellow with opaque white on the vertical lamellae.

###### Buccal armature

The jaws are large and thickly cuticularized (Fig. 31A), with a thick masticatory margin and seven to eight rows of multifid, triangular, pointed denticles (Fig. 31B). The radula (Fig. 31C) of three specimens have formulae of 37 × 13–17.1.1.1.13–17 (CASIZ 073997) and 39 × 11.1.1.1.11 in two additional specimens (SAM A35753). The rachidian teeth (Fig. 31D, E) are broad with a large, pointed central cusp that extends well beyond the 11 flanking denticles on each side. On the outer edge of each rachidian is a broad, blunt ended cusp that is longer than the 11 denticles, but shorter than the central cusp. The first lateral tooth (Fig. 31F) is extremely broad and laterally directed with a hook-shaped first denticle that extends beyond the following 16 long, pointed denticles. The next six to seven lateral teeth (Fig. 31G) are elongate with a primary denticle that is longer than the subsequent three to six denticles. The next eight to ten lateral teeth (Fig. 31H) are elongate hooks with no denticles, and the last two of these are smaller than the others.

###### Reproductive system

The reproductive organ arrangement is androdiaulic. The hermaphroditic duct is narrow and leads into the ovoid ampulla (Fig. 32). The ampulla bifurcates within the centre of the female gland mass into the short oviduct and the thick, tubular prostate. The prostate expands into the bulbous, muscular penial sheath. The round bursa copulatrix is somewhat smaller than either the ampulla or the penial sheath. From the bursa, the narrow vaginal duct expands into a bulbous vagina, which exits adjacent to the penial opening.

###### Remarks

Externally, *D. albineus* looks most similar to an *Armina* with white longitudinal ridges and the wide oral veil. However, the coloration is not similar to any described *Armina* or *Dermatobranchus*. *Armina gilchristi* Bergh, 1907 from South Africa has white ridges but the ground colour is grey and black at the anterior end of the dorsum (Bergh, 1907; Gosliner, 1987: fig. 210). *Armina loveni* (Bergh, 1860) has opaque white, wavy longitudinal ridges, but the ground colour of that species is tan or reddish (Thompson, 1988). These *Armina* species have branchial and hyponotal lamellae, as is typical for the genus, whereas *D. albineus* lacks lamellae and does not have a continuous anterior notal ridge separating the rhinophores from the notum.

Both *D. albineus* and its sympatric congener, *D. arminus* have opaque white longitudinal ridges and a wide oral veil, but the ground colour of *D. arminus* is light brown or tan, with dark spots between the ridges, not white as found in *D. albineus*.

**Figure 33:**
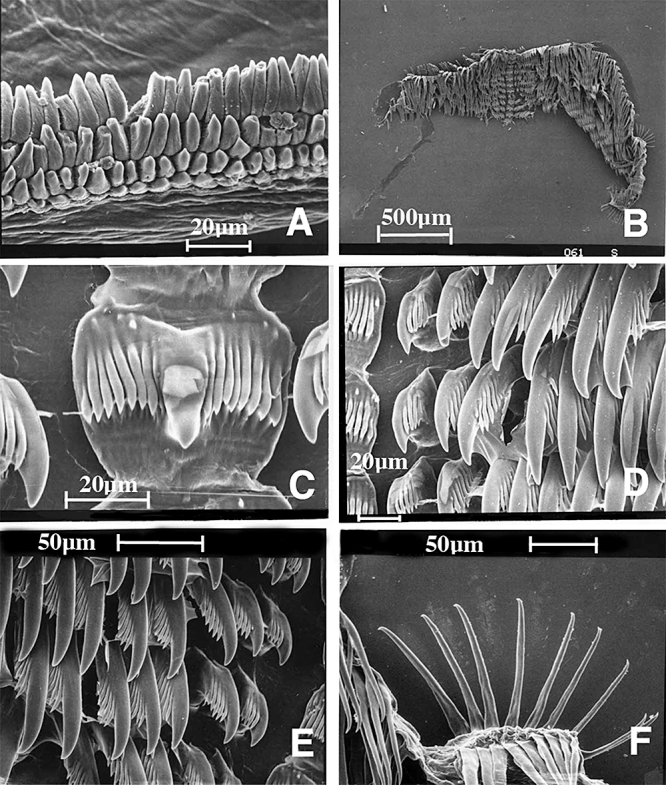
***Dermatobranchus arminus* sp. nov.** Buccal armature, SAM A35755, Bakoven, South Africa. A, masticatory border; B, entire radular width; C, rachidian tooth; D, E, inner lateral teeth; F, outer lateral teeth.

There are several other species with similar radular features such as broad rachidian and inner lateral teeth. These species include *D. fortunatus* (Bergh, 1888), *D. substriatus* Baba, 1949, *D. funiculus* sp. nov., *D. piperoides* sp. nov., *D. kokonas* sp. nov., *D. earlei* sp. nov., and *D. rodmani* sp. nov. None of these species have as many lateral teeth as *D. albineus* nor do any of these species have as many as seven rows of denticles on the masticatory surface of the jaw, and none of them have a white body colour with opaque white ridges. The radular teeth of *D. albineus* (Fig. 31) are not at all similar to the sympatric *D. arminus* sp. nov. (Fig. 33). Although the rachidian of both species is wide with an elongate median cusp, the rachidian tooth of *D. arminus* more resembles a species of *Armina* with a blunt median cusp and knob, with fewer denticles than in *D. albineus.* The inner lateral tooth differs between these two species. That of *D. albineus* is wider with 16 pointed denticles and that of *D. arminus* is shorter and has only four to six denticles. The 12 outer lateral teeth of *D. albineus* are hook-shaped, whereas in *D. arminus* the 9–12 lateral teeth are pointed and needle-like.

**Figure 34:**
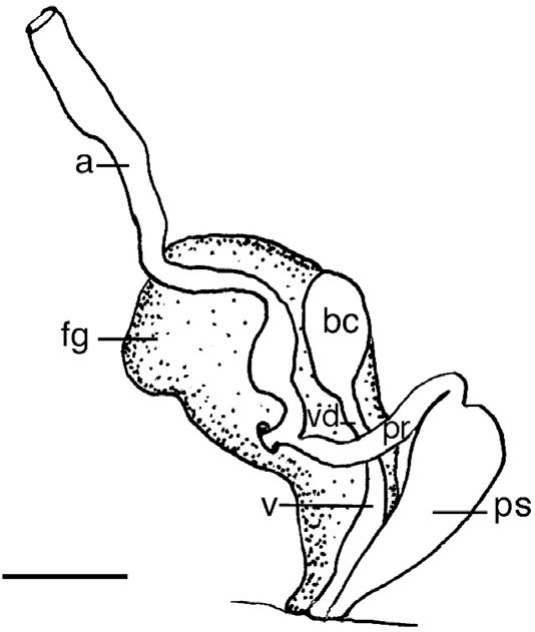
***Dermatobranchus arminus* sp. nov.** Reproductive system, SAM A35755, Bakoven, South Africa. a, ampulla; bc, bursa copulatrix; fg, female gland mass; pr, prostate; ps, penial sheath; v, vagina; vd, vaginal duct. Scale bar = 0.5 mm.

**Figure 57:**
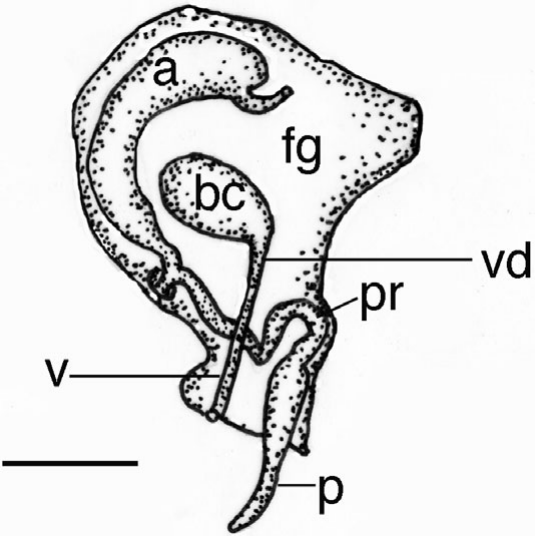
***Dermatobranchus funiculus* sp. nov.** Reproductive system, CASIZ 177377, Philippines. a, ampulla; bc, bursa copulatrix; fg, female gland mass; p, penis; pr, prostate; v, vagina; vd, vaginal duct. Scale bar = 0.5 mm.

Other *Dermatobranchus* species have similar reproductive characters such as a large, bulbous, penial sheath and slightly expanded vagina. These species include *D. albus* (Fig. 11), *D. semilunus* sp. nov. (Fig. 81), and *D. funiculus* sp. nov. (Fig. 57). However, none of these species, including *D. arminus*, have a large bulbous vagina as found in *D. albineus.* The ampulla of *D. albineus* (Fig. 32) is an ovoid mass, whereas in *D. arminus* (Fig. 34) the ampulla is elongate and narrow, in *D. albus* it is tubular, and in *D. funiculus* the ampulla is pear-shaped.

### *Dermatobranchus arminus*sp. nov. (Figs 30C, 33, 34)

*Dermatobranchus* sp. 4 Gosliner, 1987: 11, figure 214.

#### 

##### 

###### Type material

Holotype. SAM A35755, dissected, 14 mm preserved, The Mill, Bakoven, Atlantic coast, Western Cape Province, South Africa, no depth recorded, collected 16.ix.982 by W. Liltved.

###### Geographical distribution

This species has only been reported from the Atlantic coast of South Africa (present study).

###### Etymology

The specific name *arminus* is in reference to the decidedly *Armina*-like body form of this *Dermatobranchus* species.

###### External morphology

The body shape of the living animal (Fig. 30C) is broad, flattened, and narrows at the posterior end. The anterior end of the notum does not form a distinct ridge that separates the notum from the rhinophores and the rest of the oral veil. The foot does not project beyond the distinct mantle margin. There is a series of 27 longitudinal dorsal ridges. The oral veil extends forward and has blunt extensions at the corners. The wide-spaced rhinophores are behind the oral veil. They have a series of longitudinal lamellae on the rounded club. The stalk narrows noticeably. There are up to nine longitudinal hyponotal lamellae under the mantle. There are visible marginal sacs along the mantle edge. The genital opening is situated approximately one quarter of the distance along the body on the right side. The anus is situated approximately halfway towards the posterior end of the body. There are hyponotal lamellae at the anterior of the body.

The ground colour of the dorsum and foot is opaque white with a pale tan tint. The dorsal ridges are opaque white and there are dark markings between the ridges that are arranged in a band on the anterior dorsum, almost at the midline. The rhinophore stalk and club are both the same opaque white colour as the body. The oral veil is opaque white with a white margin.

###### Buccal armature

The jaws are large and thickly cuticularized, with a thick masticatory margin and four rows of triangular, pointed denticles. Part of the radula is shown in Figure 33A, and the remainder is mounted on a permanent microscope slide. The radular formula of the holotype is 32 × 40.1.1.1.40 (Fig. 33B). The rachidian teeth (Fig. 33C) have a broad base with a large, thick, and pointed central cusp that is substantially wider than the eight flanking denticles on each side. The next two lateral teeth (Fig. 33D, E) have a flat base with a large hook-shaped first denticle with four, much shorter and narrower, pointed denticles. The next several lateral teeth are long hooks with at least five pointed denticles. The outer 9–12 teeth (Fig. 33F) are pointed and needle-like.

###### Reproductive system

The reproductive organ arrangement is androdiaulic. The wide hermaphroditic duct leads into the thinner, elongate, tubular ampulla (Fig. 34). The ampulla bifurcates into the female gland mass via a short oviduct and into the short, narrow prostate. The prostate expands into the long, tubular, yet bulbous penial sheath that is as long as the prostate. The round bursa copulatrix is larger than the ampulla and smaller than the penial sheath. From the bursa, the narrow vaginal duct extends into the equally narrow vagina, which exits into the genital aperture.

###### Remarks

Externally, *D. arminus* has some features in common with species of *Armina*, including longitudinal ridges, the wide oral veil, and hyponotal lamellae. This species also has several characteristics in common with species of *Dermatobranchus*. There is no anterior notal margin and there are no branchial lamellae in *D. arminus*. In the subsequent phylogenetic analysis, *D. arminus* is clearly a member of the clade of *Dermatobranchus* species rather than a member of the *Armina* clade. The coloration of the new species also has some similarities to described *Armina* species. For example, several *Armina* have opaque white longitudinal dorsal ridges, such as *A. californica* (Cooper, 1863), but in that species, the ground colour is pinkish brown and there are no dark markings in between the ridges or arranged in a band on the anterior third of the dorsum. The oral veil of *A. californica* is narrower and has more greatly pointed corners than the wide, more blunt edged veil of *D. arminus*. *Armina loveni* (Bergh, 1860) has a yellow-tan ground colour and *Armina juliana* Ardila & Diaz, 2002 has a red background colour. Both of these species have different oral veil morphology (anvil-shaped) as compared to *D. arminus*.

*Dermatobranchus albineus* also has an opaque white ground colour with opaque white longitudinal ridges, but there are no dark markings on the dorsum. That species does not have hyponotal lamellae, whereas *D. arminus* has these lamellae.

These species also differ in their radular structure. *Dermatobranchus albineus* has a radular formula of 37–39 × 11–17.1.1. 1. 11–17, whereas the formula of *D. arminus* is 32 × 40.1.1.1.40 in a 14 mm specimen. Not only does the number of radular teeth differ markedly, but so does the form of the teeth. *Dermatobranchus albineus* has wide inner lateral teeth whereas they are narrow in *D. arminus*. The outer lateral teeth are curved in *D. albineus* and are much straighter in *D. arminus*.

These two species have reproductive differences as well. Although both have a bulbous penial sheath, the sheath of *D. arminus* is longer and more tubular than the wide, more rounded penial sheath of *D. albineus.* The vagina of *D. arminus* is much narrower than the bulbous vagina of *D. albineus.*

### *Dermatobranchus caeruleomaculatus*sp. nov. (Figs 30E, F, 35–37)

*Dermatobranchus* sp. 12 Gosliner, Behrens & Valdés, 2008: 312, bottom photos.

**Figure 35:**
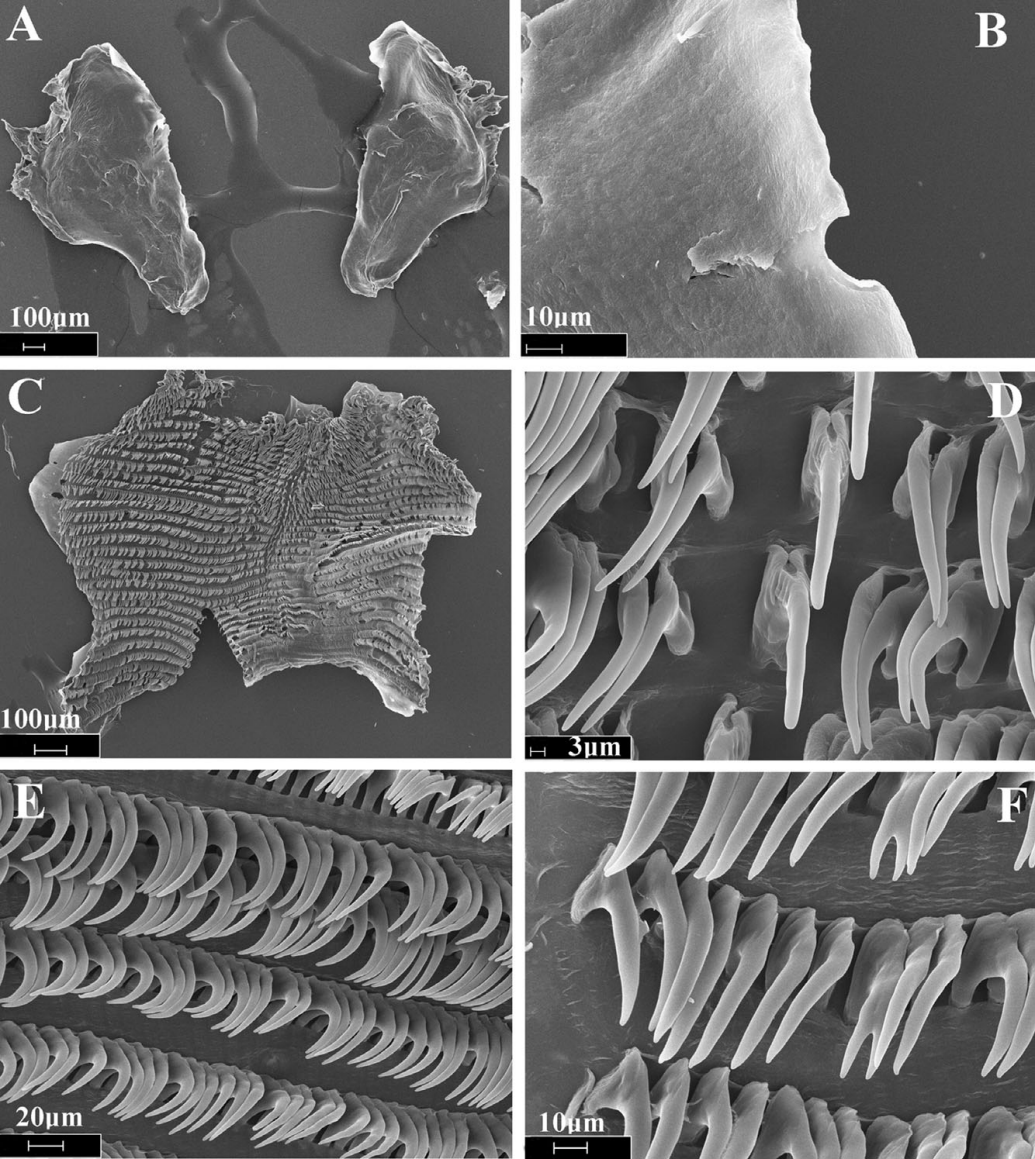
***Dermatobranchus caeruleomaculatus* sp. nov.** Buccal armature, CASIZ 174173, Tioman, Malaysia. A, jaws, B, masticatory margin; C, entire radula; D, central portion of radula; E, middle lateral teeth; F, outer radular teeth.

**Figure 36:**
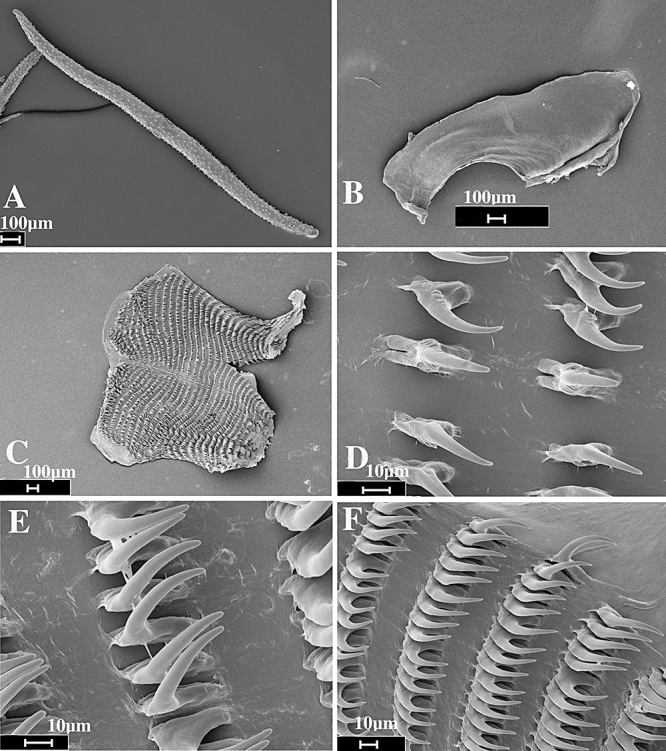
***Dermatobranchus caeruleomaculatus* sp. nov.** Prey sclerite and buccal armature, CASIZ 177481, Mabini, Philippines. A, sclerite of *Dendronephthya* sp. prey from digestive tract; B, jaw; C, entire radula; D, central portion of radula; E, middle lateral teeth; F, outer radular teeth.

**Figure 37:**
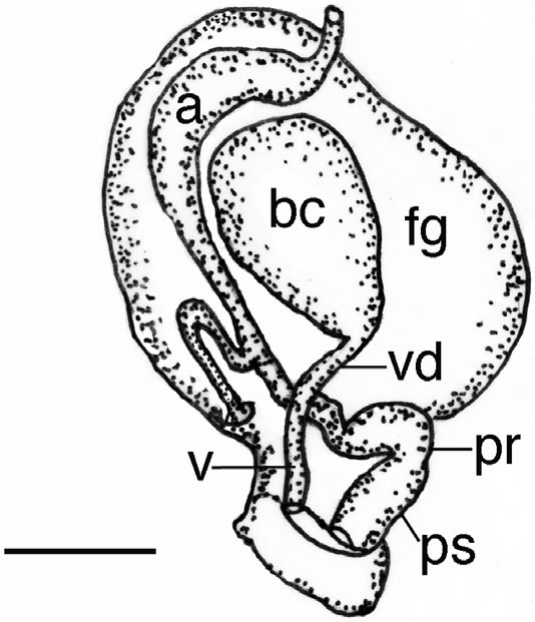
***Dermatobranchus caeruleomaculatus* sp. nov.** Reproductive system, CASIZ 174173, Tioman, Malaysia. a, ampulla; bc, bursa copulatrix; fg, female gland mass; pr, prostate; ps, penial sheath; v, vagina; vd, vaginal duct. Scale bar = 0.83 mm.

#### 

##### 

###### Type material

Holotype: CASIZ 174172, Waterfall Bay, south side Tioman Island, east Malaysia, 15 m depth, collected 4.x.2007 by T. Gosliner & D. Behrens. Paratypes; CASIZ 174173, two specimens, one dissected, Waterfall Bay, south side Tioman Island, east Malaysia, 15 m depth, collected 4.x.2007 by T. Gosliner & D. Behrens. CASIZ 178234, one specimen sampled for DNA, Waterfall Bay, south side Tioman Island, east Malaysia, 15 m depth, collected 4.x.2007 by T. Gosliner & D. Behrens. CASIZ 177481, one specimen, dissected, with subsample for DNA, Mainit Bubbles, Calumpan Peninsula, Batangas Bay, Luzon Philippines (13°41.325498′N, 120°53.8308′E), 10 m depth, collected 20.iii.2008 by Gosliner *et al.* CASIZ 177482, one specimen, sampled for DNA, Mainit Bubbles, Calumpan Peninsula, Batangas Bay, Luzon Philippines (13.6887583°N, 120.897180°E), 10 m depth, collected 20.iii.2008 by Gosliner *et al.*

###### Geographical distribution

Known from Indonesia, eastern Malaysia, Philippines (Gosliner *et al.*, 2008), and Papua New Guinea (Coleman, 2008)

###### Etymology

The specific name *caeruleomaculatus* is a noun in apposition and refers to the bright blue spots present on the oral veil and notal margins.

###### Natural history

This species was found feeding on the soft coral, *Dendronephthya* sp., on sandy slopes in 10–15 m of water. In one specimen (CASIZ 177481), the entire anterior end of the body cavity was full of sclerites of *Dendronephthya* sp. (Fig. 36A). The acutely pointed sclerites had completely penetrated the digestive tract and were present throughout the inside of the body wall. The egg mass (Fig. 30F) is a highly convoluted ribbon attached to the sand by a mucus thread. There is a single egg per egg capsule.

###### External morphology

The body shape of the living animal (Fig. 30E) is elongate, but broad, slightly flattened, and narrows at the posterior end. The foot does not project beyond the distinct mantle margin. The dorsum has approximately 24 well-elevated, longitudinal ridges on either side of the midline that may further divide posteriorly. The oral veil is large and expansive with slightly pointed corners. The well-separated, bulbous rhinophores are situated behind the oral veil. They have a series of longitudinal lamellae on the rounded club, which has a small rounded apex. The stalk does not narrow noticeably. Marginal sacs are readily visible along the mantle edge.

There are no hyponotal or branchial lamellae under the mantle margin. The genital opening is situated in the anterior quarter of the body. The anus is situated approximately half of the way to the posterior end of the body.

The ground colour of the dorsum is white with a series of brown spots arranged in longitudinal lines along the margin of the ridges. The oral veil and the margin of the notum have a series of blue spots, each with a black centre. There are also fine brown spots on the posterior end of the oral veil. There is a marginal yellow-orange band on both the notum and the oral veil. The rhinophore stalk is white with brown spots and the club is dark brown to black with opaque white pigment on the lamellae. The tip of the rhinophores is white.

###### Buccal armature

The jaws (Figs 35A, 36B) are short and thick. Along the inner edge of the jaws, the masticatory margin is slightly irregular and lacks any denticles (Fig. 35B). The radula (Figs 35C, 36C) is short and wide with formulae of 33 × 108.1.1.1.108 (CASIZ 174173) and 33 × 68.1.1.1. (CASIZ 177481). The rachidian teeth (Figs 35D, 36D) are narrow and elongate with a long central cusp with a rounded tip. At the base of each rachidian tooth are four to five short denticles on either side of the cusp. The inner laterals have a narrow base with an elongate cusp. They may have four to five denticles on their outer side (Fig. 36D), or may lack any basal denticles (Fig. 35D). The middle lateral teeth (Figs 35E, 36E) and the outer lateral teeth (Figs 35F, 36F) are all evenly arched with an elongate cusp and all lack any denticles.

###### Reproductive system (Fig. 37)

The ampulla is thick and simply curved. It bifurcates to the large female gland mass and the vas deferens. The majority of the female gland mass is composed of the mucous gland whereas the membrane and albumen glands are much smaller. The vas deferens is relatively wide and convoluted and widens further as it enters the short bulbous penial sheath. Within the penial sheath, the penis is wide and short and terminates in a somewhat acute apex. Adjacent to the penis is the narrow curved vagina, which terminates in a relatively large, pyriform bursa copulatrix.

###### Remarks

**Figure 43:**
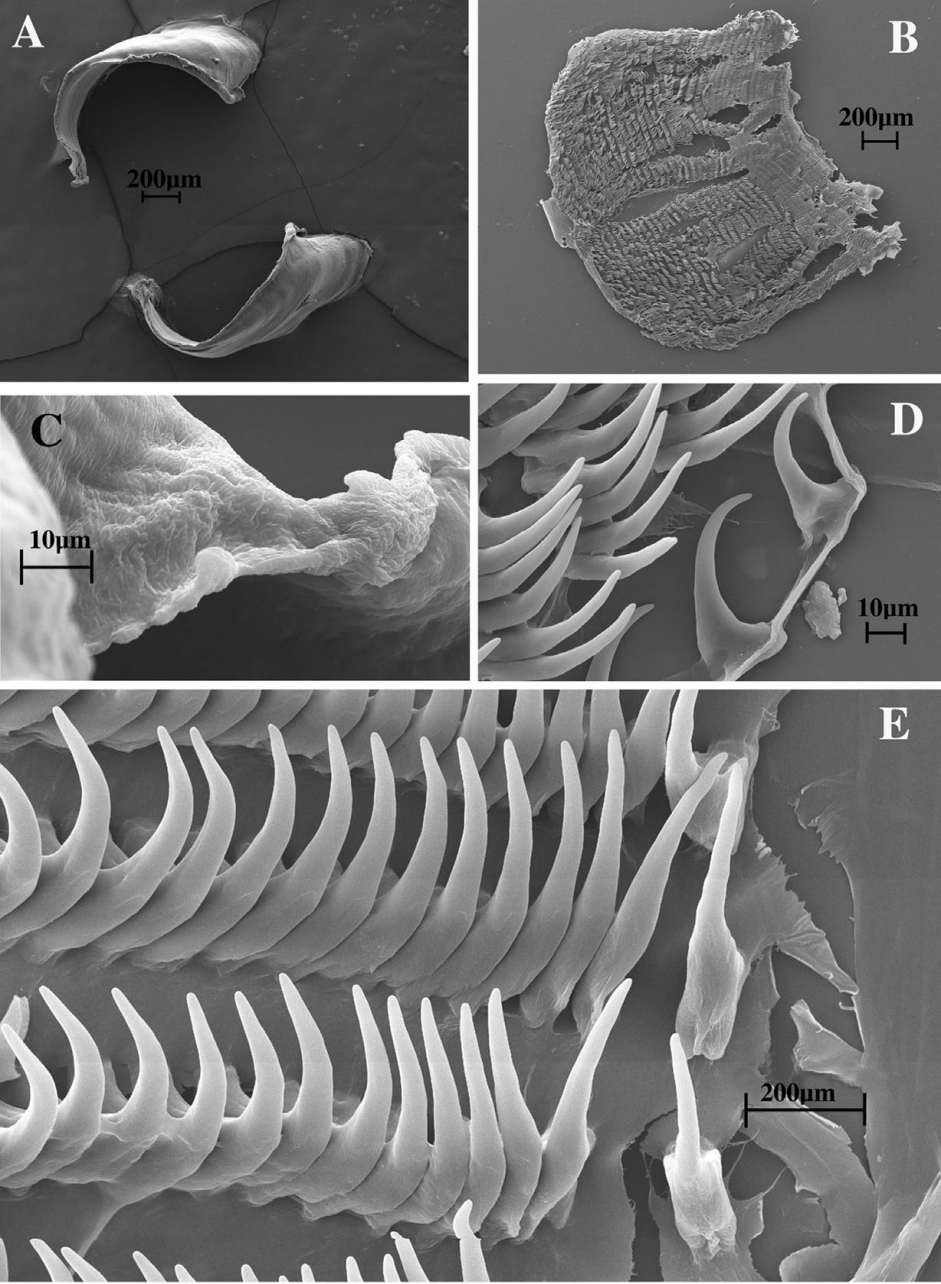
***Dermatobranchus dendronephthyphagus* sp. nov.** Buccal armature, CASIZ 115751, Okinawa, Ryukyu Islands. A, jaws; B, entire radula; C, masticatory margin; D, outer radular teeth; E, central portion of radula.

**Figure 44:**
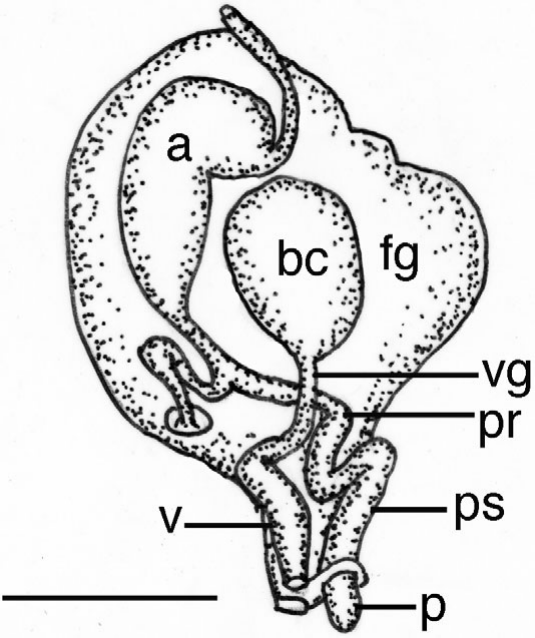
***Dermatobranchus dendronephthyphagus* sp. nov.** Reproductive system, CASIZ 115751, Okinawa, Ryukyu Islands. a, ampulla; bc, bursa copulatrix; fg, female gland mass; p, penis; pr, prostate; ps, penial sheath; v, vagina; vd, vaginal duct. Scale bar = 1.0 mm.

*Dermatobranchus caeruleomaculatus* is one of several large species with a broad body, smooth jaws, and a wide radula consisting of numerous teeth. These taxa include *D. gonatophorus*, *D. ornatus*, *D. nigropunctatus*, *D. sagamianus*, *D. multidentatus*, *D. tongshanensis*, *D. multistriatus, D. leoni*, and *D. dendronephthyphagus*. Of these taxa, *D. caeruleomaculatus* is the only species that has fine brown spotting and blue spots on the notal and oral veil margins. Of these taxa only *D. caeruleomaculatus* and *D. dendronephthyphagus* have a narrow rachidian tooth with an elongate, narrow cusp. Both of these species also have thin, elongate lateral teeth that lack denticles. In *D. caeruleomaculatus* (Figs 35D, 36D), the rachidian tooth has three to four basal denticles whereas denticles are entirely absent in *D. dendronephthyphagus* (Fig. 43E). It is interesting to note that these two species with similarly shaped radular teeth have been observed feeding on soft corals of the genus *Dendronephthya*. The reproductive system of *D. caeruleomaculatus* (Fig. 37) differs from that of *D. dendronephthyphagus* (Fig. 44) in two significant regards. In *D. caeruleomaculatus*, the penial sac is bulbous and the vagina is thin throughout its length, whereas in *D. dendronephthyphagus* the penial sac is cylindrical and the vagina is wide basally and much narrower distally.

### *Dermatobranchus caesitius*sp. nov. (Figs 30D, 38, 39)

*Dermatobranchus* sp. 2 Gosliner, 1987:110, figure 212.

**Figure 38:**
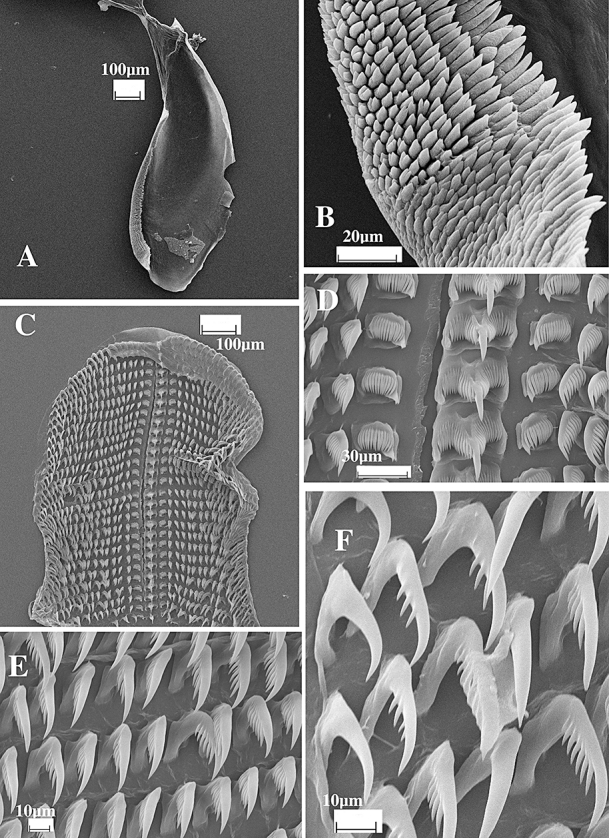
***Dermatobranchus caesitius* sp. nov.** Buccal armature, SAM A35750, Umgazana, South Africa. A, jaw; B, masticatory margin; C, entire radular width; D, central portion of the radula; E, middle lateral teeth; F, outer lateral teeth.

**Figure 39:**
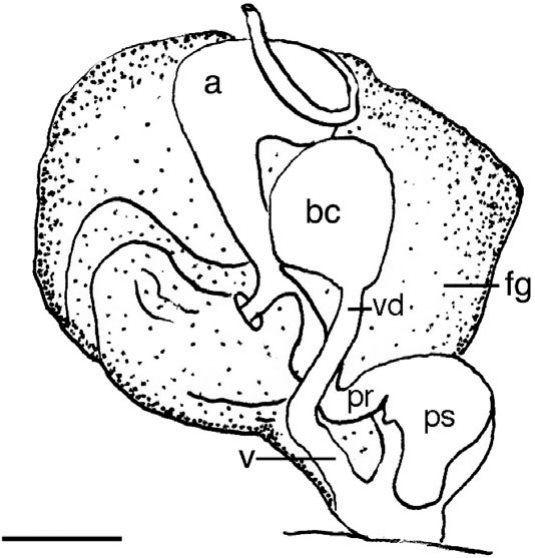
***Dermatobranchus caesitius* sp. nov.** Reproductive system, SAM A35750, Umgazana, South Africa. a, ampulla; bc, bursa copulatrix; fg, female gland mass; pr, prostate; ps, penial sheath; v, vagina; vd, vaginal duct. Scale bar = 1.0 mm.

*Dermatobranchus* sp. 5 Gosliner, Behrens & Valdés, 2008:309, bottom photo.

#### 

##### 

###### Type material holotype

CASIZ 174137, 18 mm preserved, Umgazana, Indian Ocean, South Africa, intertidal zone, collected 23.iv.1982 by T. Gosliner. Paratypes: SAM A35750, 14 specimens, 10–20 mm preserved, three dissected, Umgazana, Indian Ocean, South Africa, intertidal zone, collected 23.iv.1982 by T. Gosliner, no depth recorded. SAM A35760, one specimen, 10 mm preserved, Umgazana, Indian Ocean, South Africa, collected 28.x.1981 by T. Gosliner.

###### Geographical distribution

This species has been reported from only a single locality, Umgazana on the Indian Ocean coast of South Africa (Gosliner, 1987, present study).

###### Natural history

This species is found in the subtropical waters of the Indian Ocean coast of South Africa, from the lower intertidal zone, where it feeds upon the blue soft coral, *Sansibia flava* (May, 1899).

###### Etymology

The specific name *caesitius* is an adjective, from the Latin word for ‘bluish’, referring to the colour of the dorsum.

###### External morphology

The body shape of the living animal (Fig. 30D) is elongate, flattened, and narrows at the posterior end. The wide foot does not project beyond the distinct mantle margin. There is a series of broken longitudinal dorsal ridges with dorsal tubercles clustered between the ridges. The oral veil extends forward and is pointed at the corners. The rhinophores are situated behind the oral veil. They have a series of longitudinal lamellae on the rounded club. The stalk narrows noticeably. There are no lamellae on the stalk. Marginal sacs are readily visible along the mantle edge. There are no branchial or hyponotal lamellae. The genital opening is situated approximately one-quarter of the way along the body side and the anus is situated about halfway along the body side.

The ground colour of the dorsum, the dorsal ridges, and the foot is opaque blue with a white mantle margin containing brown regularly spaced spots and an orange edge. The dorsal ridges have a yellowish tint particularly noticeable along the median. The rhinophore stalk is the same blue colour and the club is black. The oral veil is blue with opaque white along the margin.

###### Buccal armature

The jaws are large and thickly cuticularized (Fig. 38A), with a thick masticatory margin and eight to ten rows of simple, triangular, pointed denticles (Fig. 38B). The radular formulae of the two paratypes (SAM A35750) are 27 × 21.1.1.1.21 and 28 × 18.1.1.1.18 (Fig. 38C). The rachidian teeth (Fig. 38D) are moderately broad and laterally directed, with a large, pointed central cusp that is twice as long as the 13–16 flanking denticles on each side. On the outer edge of each rachidian tooth is a broad, rounded cusp that is longer than the adjacent denticles, but shorter than the central cusp. The inner lateral teeth (Fig. 38D) are broad with a pointed first denticle that is longer than the following 10–14 long, pointed denticles. The next 16–19 lateral teeth (Fig. 38E) are elongate hooks with up to 5–11 denticles, the number decreasing on the teeth furthest from the rachidian. The outer two teeth (Fig. 35F) have no denticles and the outermost is smaller than all the other lateral teeth.

###### Reproductive system

The reproductive organ arrangement is androdiaulic. The hermaphroditic duct is long and narrow and leads into the elongate, pear-shaped ampulla (Fig. 39). The ampulla bifurcates near the centre of the female gland mass into a short oviduct and the short, tubular prostate. The prostate expands into the large, bulbous, muscular penial sheath. The round bursa copulatrix is smaller than the ampulla and the same size as the penial sheath. From the bursa, the narrow vaginal duct extends into a slightly wider vagina, which exits into the genital atrium.

###### Remarks

Externally, *D. caesitius* (Fig. 30D) looks most similar to *D. earlei* (Fig. 42C). Both species have a pale blue overall appearance with a white mantle margin and white oral veil and have rhinophores with black pigment on the club. However, *D. earlei* has large brown blotches scattered on the dorsum and along the mantle margin and an orange margin of the oral veil.

**Figure 48:**
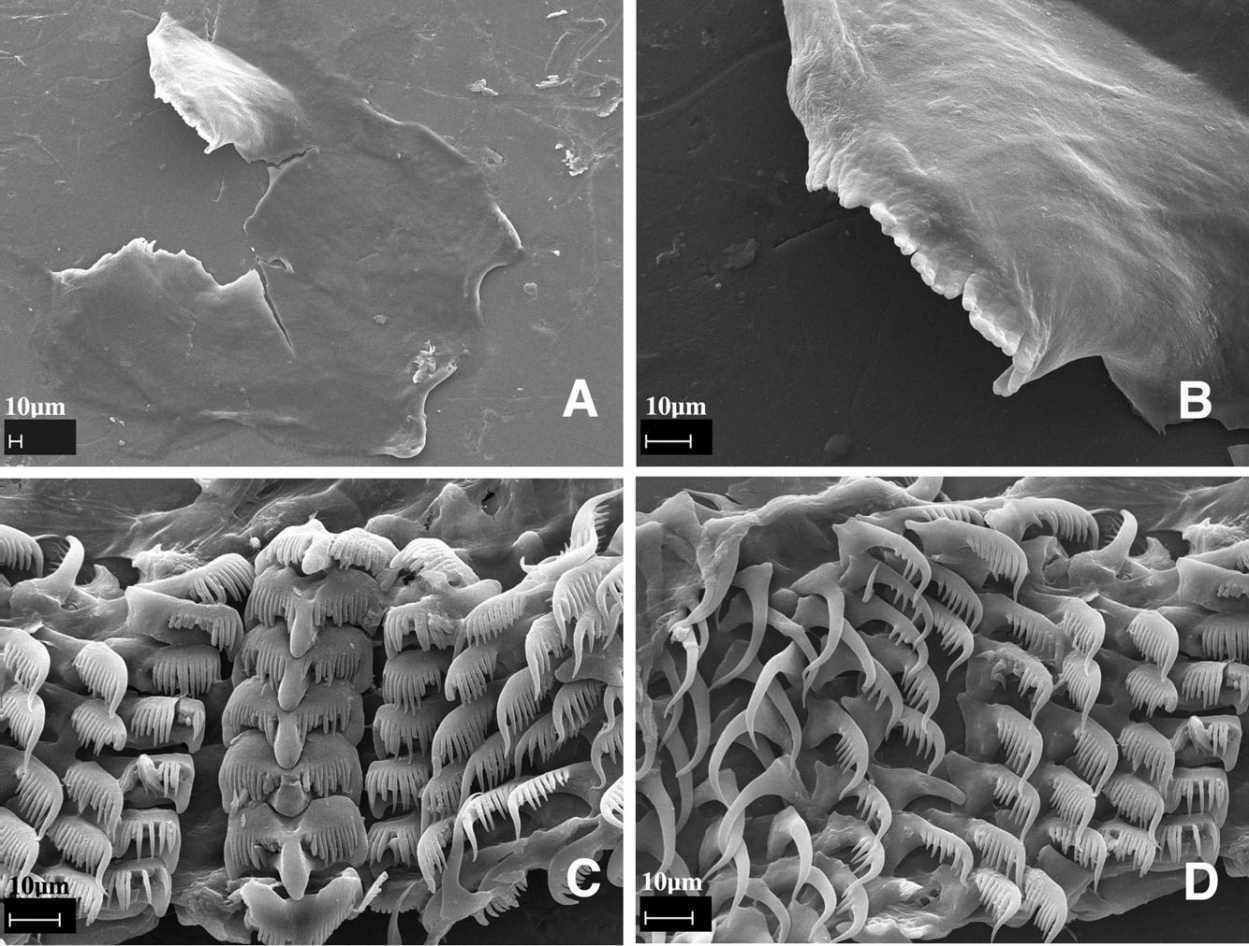
***Dermatobranchus earlei* sp. nov.** Buccal armature, CASIZ 097441, Oman. A, jaws; B, masticatory margin; C, central portion of the radula; D, middle and outer lateral teeth.

These two species differ markedly internally, as well. For example, the radular formula of these species is quite different. The formula of *D. caesitius* is 27–28 × 18–21.1.1.18–21, whereas that of *D. earlei* is ×9.1.1.1.9, with an unknown number of radular rows. The rachidian tooth of *D*. *caesitius* (Fig. 38D) has a narrower base and is more claw-shaped, with a much longer central cusp than in *D. earlei* (Fig. 48C).

**Figure 49:**
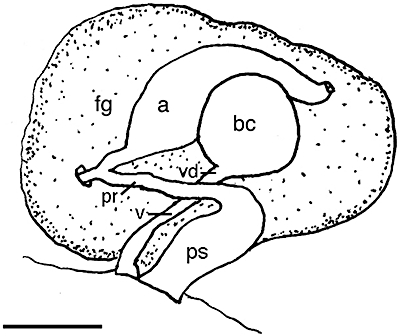
***Dermatobranchus earlei* sp. nov.** Reproductive system. CASIZ 097441, Oman. a, ampulla; bc, bursa copulatrix; fg, female gland mass; pr, prostate; ps, penial sheath; v, vagina; vd, vaginal duct. Scale bar = 0.35 mm.

There are reproductive system differences as well. The penial sheath of *D. caesitius* (Fig. 39) is much shorter and wider than that of *D. earlei* (Fig. 49). The prostates of *D. earlei* and *D. caesitius* are similar in length but slightly longer in *D. earlei*.

**Figure 50:**
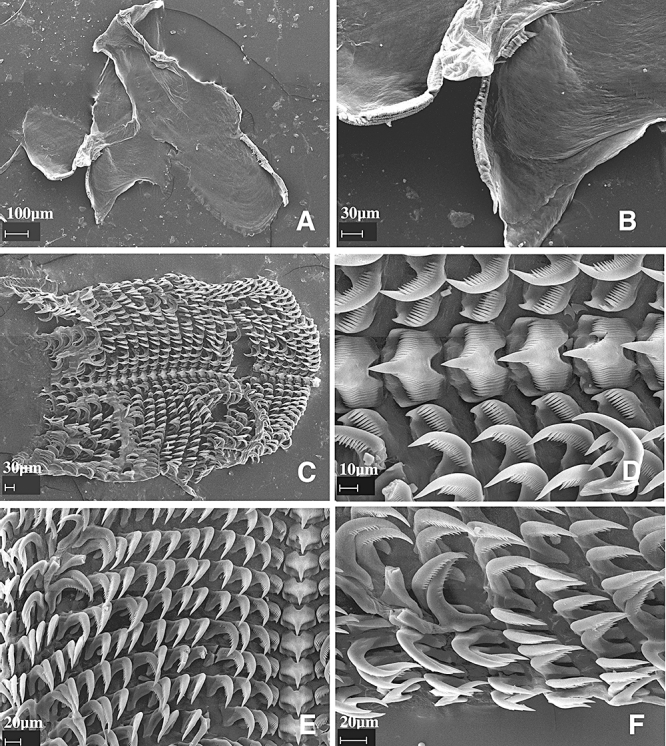
***Dermatobranchus fasciatus* sp. nov.** Buccal armature, CASIZ 171384, Panglao, Bohol, Philippines. A, jaws; B, masticatory margin; C, entire radula; D, central portion of radula; E, middle lateral teeth; F, outer lateral teeth.

**Figure 51:**
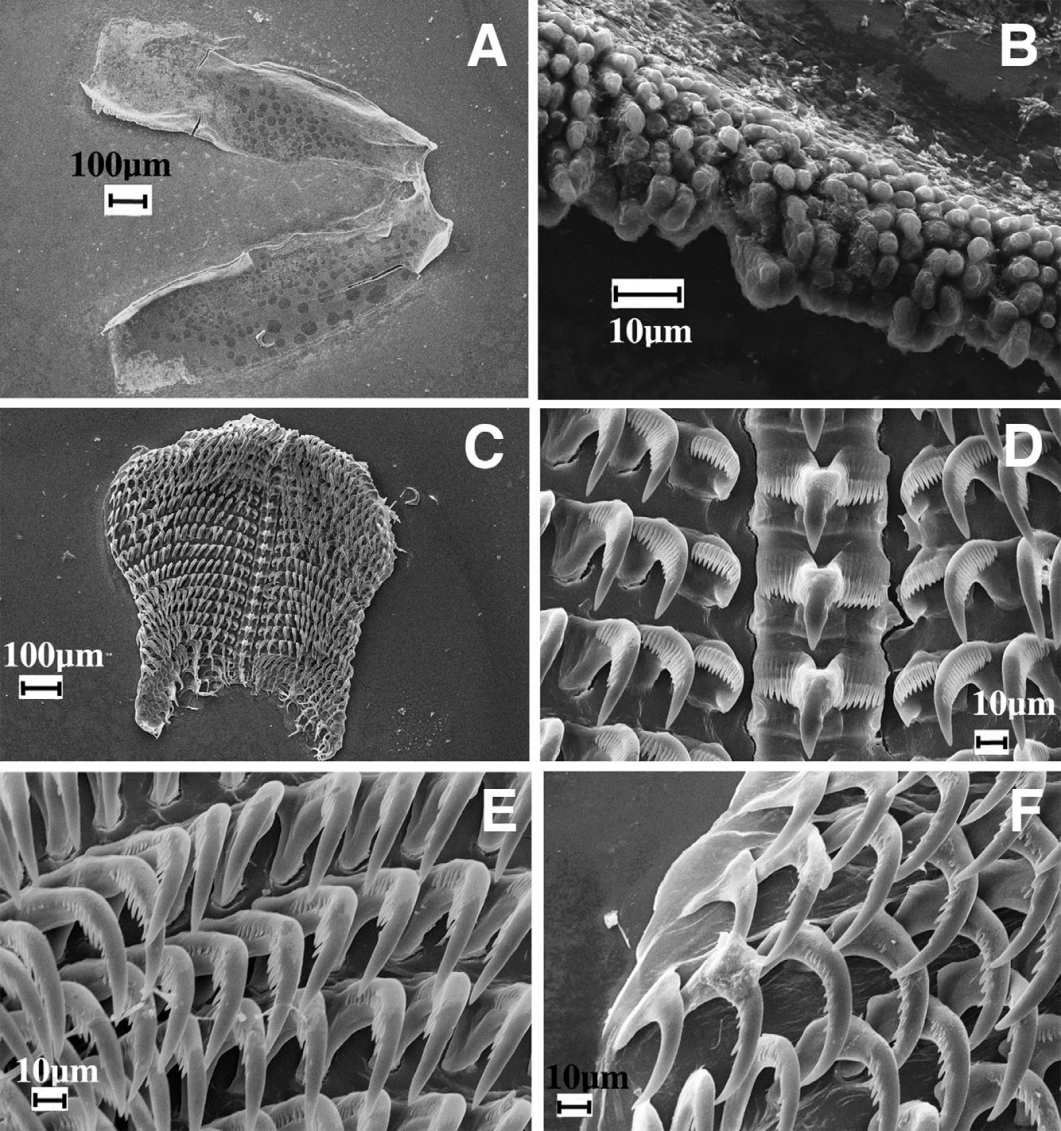
***Dermatobranchus fasciatus* sp. nov.** Buccal armature, CASIZ 073049, Madang, Papua New Guinea. A, jaws; B, masticatory margin; C, entire radula; D, central portion of radula; E, middle lateral teeth; F, outer lateral teeth.

**Figure 52:**
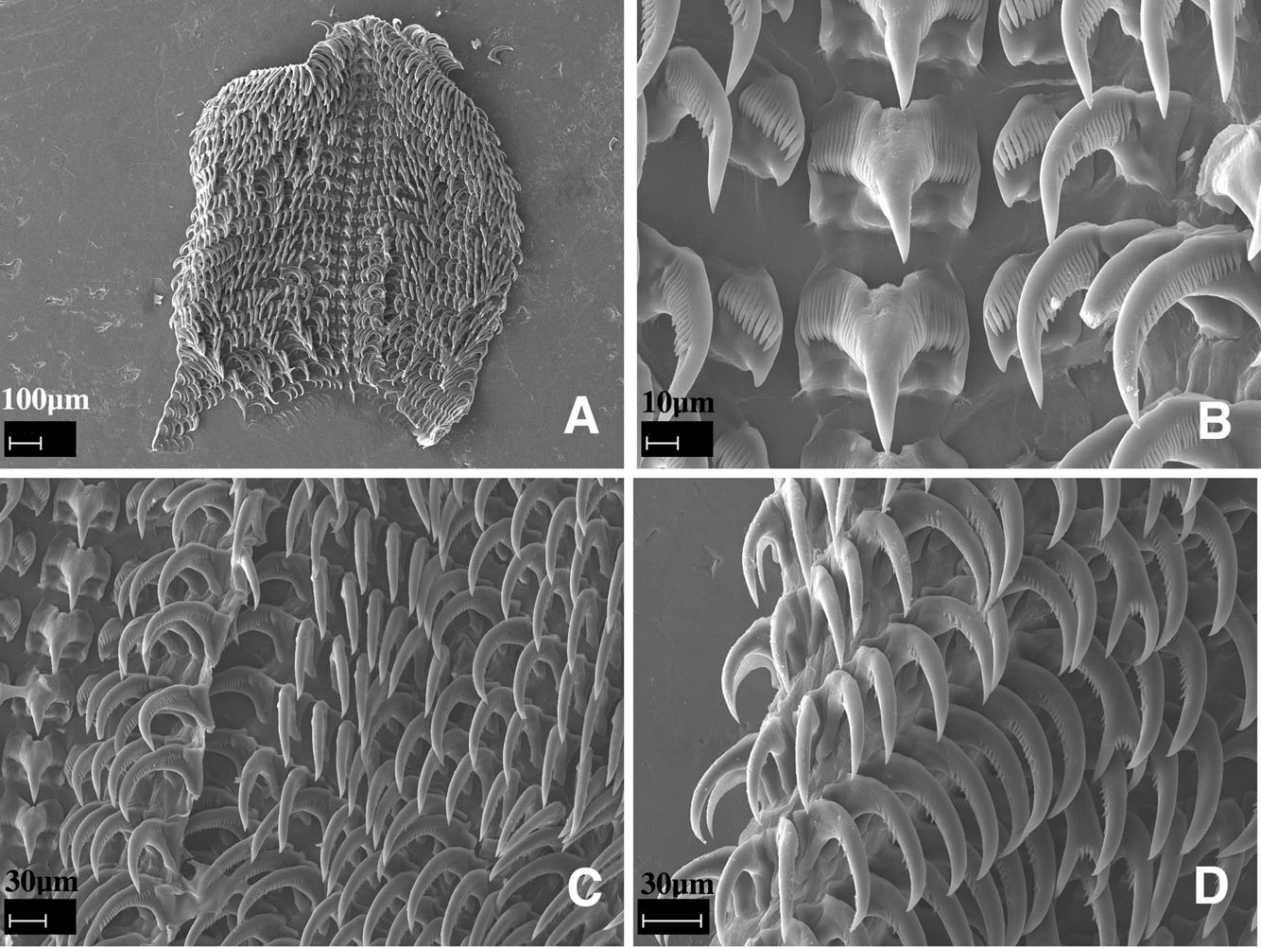
***Dermatobranchus fasciatus* sp. nov.** Buccal armature, CASIZ 171387, Panglao, Bohol, Philippines. A, entire radula; B, central portion of radula; C, middle lateral teeth; D, outer lateral teeth.

The formula and shape of the radular teeth of *D. caesitius* are similar to those found in *D. fasciatus* (Figs 50–52). Both species have a wide rachidian tooth with numerous fine denticles and an acutely pointed, elongate central cusp. The inner lateral tooth of *D. caestitius* is broader and laterally directed whereas that of *D. fasciatus* is narrower and more posteriorly directed. The middle lateral teeth of *D. caesitius* have 5–11 denticles whereas those of *D. fasciatus* have 15–17 denticles.

**Figure 53:**
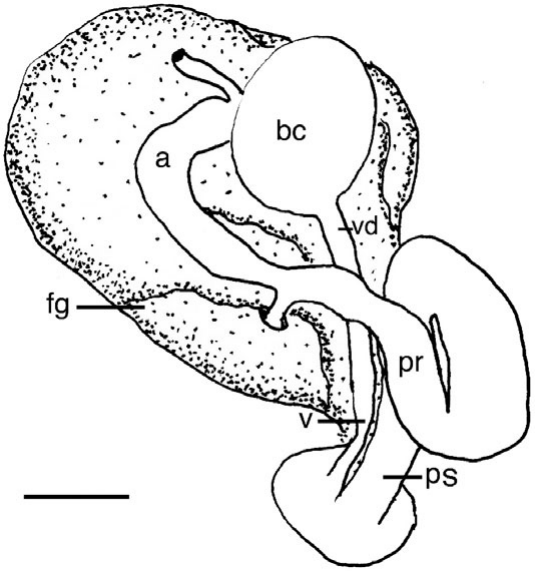
***Dermatobranchus fasciatus* sp. nov.** Reproductive system, CASIZ 107424, Lombok, Indonesia. a, ampulla; bc, bursa copulatrix; fg, female gland mass; pr, prostate; ps, penial sheath; v, vagina; vd, vaginal duct. Scale bar = 0.6 mm.

*Dermatobranchus caesitius* has a blue colour with prominent irregular longitudinal ridges on the notum. In contrast, *D. fasciatus* is white with a brown transverse saddle and irregular tubercles on the notum. The reproductive anatomy also differs consistently. In *D. caesitius*, the penial sheath is much wider than the prostate, whereas in *D. fasciatus* (Fig. 53) they are approximately the same diameter.

Several other *Dermatobranchus* species have reproductive similarities to *D. caesitius*, such as a large, bulbous penial sheath and slightly expanded vagina. These species include *D. albus* and *D. semilunus.* However, neither of these species have similar radular or external morphology.

### *Dermatobranchus cymatilis*sp. nov. (Figs 30G, 40, 41)

*Dermatobranchus* sp. 10 Gosliner, Behrens & Valdés, 2008: 312, top photo.

#### 

##### 

###### Type material

Holotype: CASIZ 074694, 17 mm preserved, dissected, Horseshoe Cliffs, west-north-west of Onna Village, Okinawa, Ryukyu Islands, Japan, 60 m depth, collected 27.ix.1989 by R. Bolland. Paratype: CASIZ 104706, one specimen, 12 mm preserved, dissected. Horseshoe Cliffs, Okinawa, Ryukyu Islands, Japan, 53 m depth, collected 20.vii.1994 by R. Bolland (RFB3286-A).

###### Geographical distribution

This species is only known from Okinawa (present study).

###### Etymology

The specific name *cymatilis* is from the Latin adjective meaning ‘sea-coloured or blue’ to describe the deep blue foot margin on this species.

###### External morphology

The body shape of the living animal (Fig. 30G) is broad, flattened, and narrows at the posterior end. The foot does not project beyond the distinct mantle margin. There is a series of longitudinal dorsal ridges. The oral veil extends forward and has blunt extensions at the corners. The wide-spaced rhinophores are behind the oral veil. They have a series of longitudinal lamellae on the rounded club. The stalk does not narrow noticeably. Marginal sacs are visible along the mantle edge. Approximately one-quarter of the way down on the right side of the body is the genital opening. The anus is situated approximately one-third of the way from the posterior end of the body.

The ground colour of the dorsum and foot is opaque white, although the foot has a blue margin. There is a pinkish-orange patch about midway on the dorsum that extends the width of the dorsum. Along the mantle margin and perpendicular to the edge, there are dark rays of colour that extend both downward onto the foot and upward into dark patches along the mantle edge. The dark patches extend up and across the dorsum as broken, dark bands. The dorsal ridges have dark crests, although the colour is broken along the length of the ridges. The rhinophore stalk is white and the club is dark. The oral veil is opaque white with dark perpendicular rays. There is an orange margin along a dark patch on the posterior side of the veil, close to the body.

###### Buccal armature

**Figure 40:**
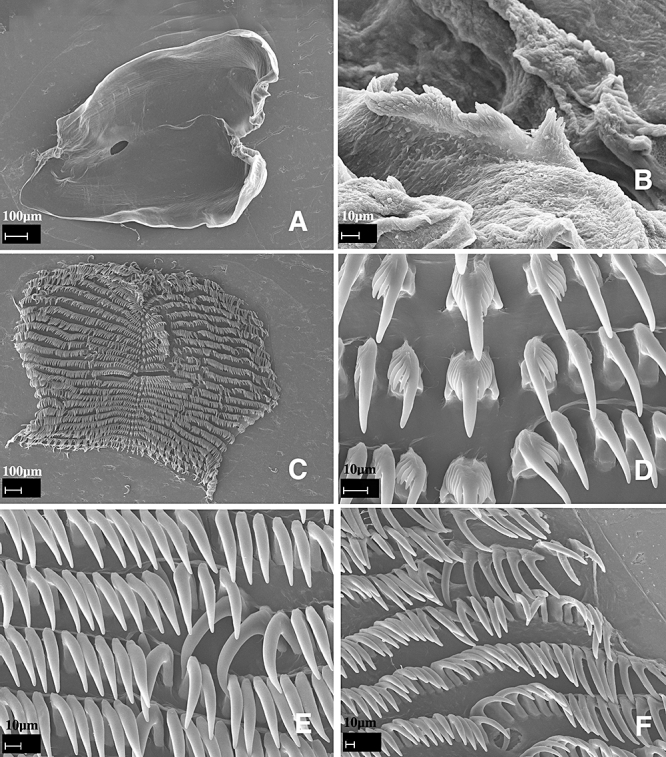
***Dermatobranchus cymatilus* sp. nov.** Buccal armature, CASIZ 104706, Okinawa, Japan. A, jaws; B, masticatory margin; C, entire radular width; D, central portion of the radula; E, middle lateral teeth; F, outer lateral teeth.

The jaws are large and thickly cuticularized (Fig. 40A), with a thick masticatory margin and multiple rows of long, pointed denticles (Fig. 40B). The radular formula of the holotype is 27 × 66.1.1.1.66 (Fig. 40C). The rachidian teeth (Fig. 40D) have a narrower base than most *Dermatobranchus,* with a large, thick, and very long central cusp that is substantially wider than the up to seven flanking denticles on each side. The inner lateral teeth (Fig. 40D) each have a triangular base with a very prominent, long first denticle with four, much shorter and narrower, pointed denticles. The next 66 lateral teeth are long hooks without denticles (Fig. 40E, F).

###### Reproductive system

**Figure 41:**
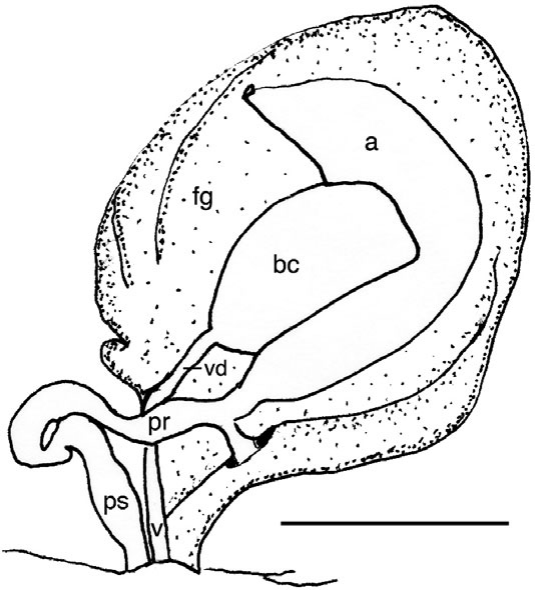
***Dermatobranchus cymatilus* sp. nov.** Reproductive system, CASIZ 104706, Okinawa, Japan. a, ampulla; bc, bursa copulatrix; fg, female gland mass; pr, prostate; ps, penial sheath; v, vagina; vd, vaginal duct. Scale bar = 0.86 mm.

The reproductive organ arrangement is androdiaulic. The hermaphroditic duct leads into the wide tubular ampulla (Fig. 41). The ampulla bifurcates into the female gland mass via a short oviduct and into the short, narrow prostate. The prostate curves once then expands into the bulbous penial sheath. The round bursa copulatrix is smaller than the ampulla and much larger than the penial sheath. From the bursa, the very narrow vaginal duct extends into the equally narrow vagina, which exits into the genital atrium alongside the penial sheath.

###### Remarks

There are several *Dermatobranchus* species with white ground colour and dark crested dorsal ridges [*D. striatus* Hasselt, 1824 (Fig. 15I), *D*. *dendronephthyphagus* (Figs 30H, 42A), and *D. semistriatus* (Baba, 1949: pl. 30, fig. 111)]. *Dermatobranchus cymatilis* has dark patches and ‘rays’ of dark coloration along the mantle edge and dark perpendicular lines along the foot edge. These rays of dark colour are absent in *D. striatus, D. semistriatus*, and *D*. *dendronephthyphagus.* The yellow coloration on the posterior portion of the oral veil of *D. cymatilis* differs from the fine orange margin of the veil found in *D. semistriatus*, *D. striatus*, and *D*. *dendronephthyphagus.* In addition, *D. semistriatus* has scattered small black spots on the notum, oral veil, and foot that are absent in *D. cymatilus*. Although the two species have a similar radular formula, *D. cymatilus* has much longer cusps on the radular teeth with fewer denticles on the teeth. The only other species with blue and yellow or orange colour on the oral veil is *D. caeruleomaculatus*. In this species, the blue spots are on the oral veil and the mantle margin, whereas in *D. cymatilus* the blue pigment is on the foot margin. The two differ internally as well. In *D*. *caeruloemaculatus*, the jaws have a smooth masticatory margin whereas those of *D. cymatilus* are denticulate. Additionally, the inner lateral tooth of *D. caeruleomaculatus* is smooth whereas that of *D. cymatilus* is denticulate.

**Figure 78:**
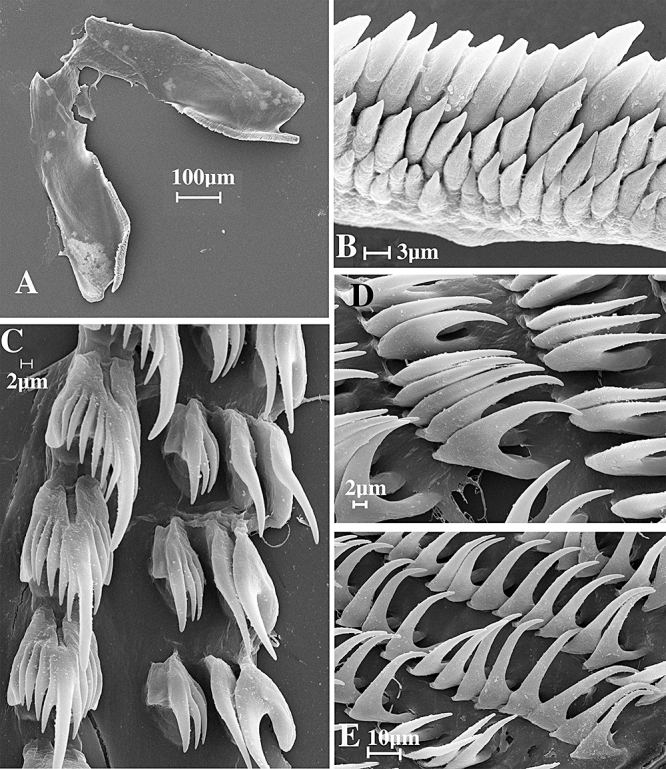
***Dermatobranchus semilunus* sp. nov.** Buccal armature, CASIZ 073045, Madang, Papua New Guinea. A, jaws; B, masticatory margin; C, central portion of radula; D, middle lateral teeth; E, outer lateral teeth.

**Figure 79:**
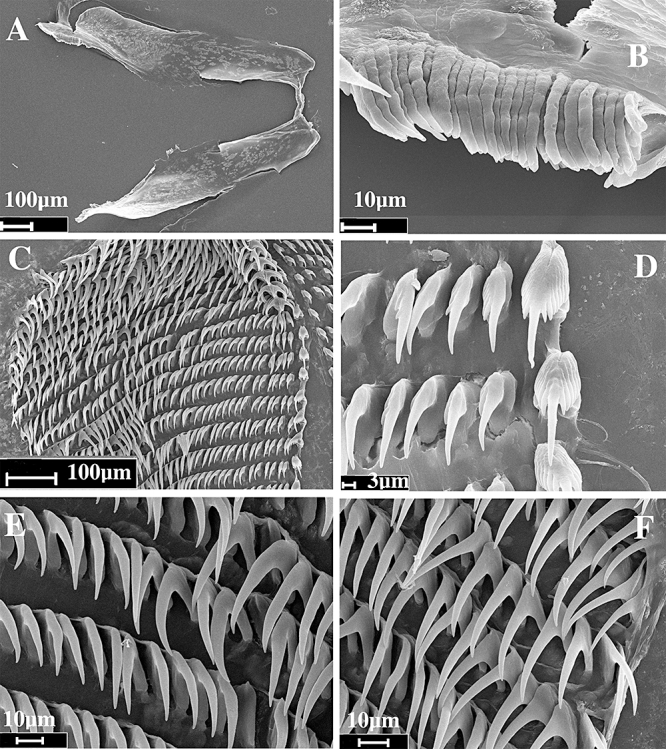
***Dermatobranchus semilunus* sp. nov.** Buccal armature, CASIZ 174142, Pulau Varella, Malaysia. A, jaws; B, masticatory margin; C, entire radula; D, central portion of radula; E, middle lateral teeth; F, outer lateral teeth.

**Figure 80:**
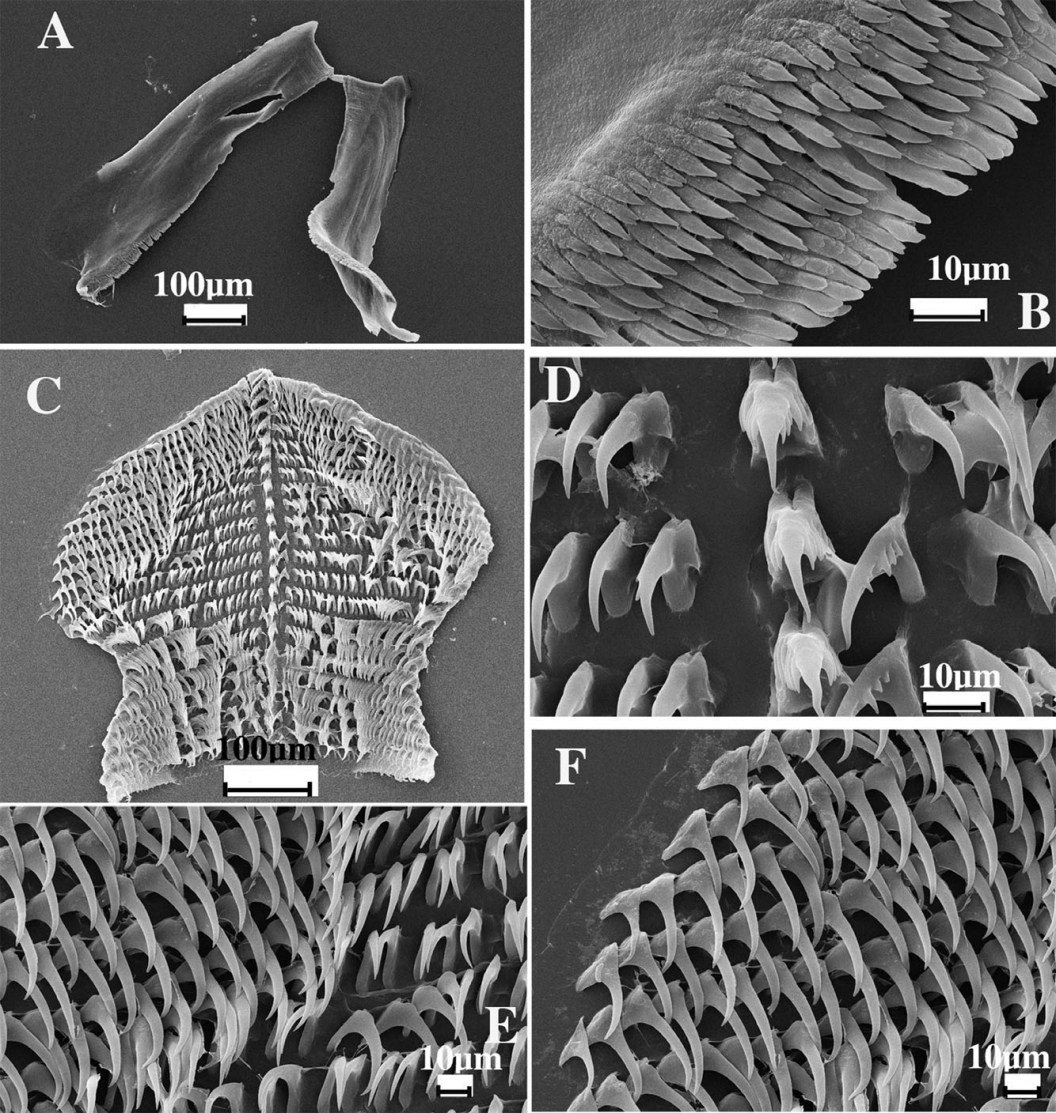
***Dermatobranchus semilunus* sp. nov.** Buccal armature, CASIZ 110407, Cabilao, Bohol, Philippines. A, jaws; B, masticatory margin; C, half-row of radular teeth; D, central portion of radula; E, middle lateral teeth; F, outer lateral teeth.

The radular morphology of *D. cymatilis* differs from all the externally similar species, but is similar to that of *D. semilunus*. Both *D. cymatilus* (Fig. 40) and *D. semilunus* have a broad radula, but *D. cymatilus* has many more teeth per half row (67) than does *D. semilunus* (30–34) (Figs 78–80). In both of these species, the rachidian tooth has a much narrower base and a very prominent central cusp that is much longer than the central cusp of the rachidian tooth. In both species the inner lateral teeth have an elongate central cusp that is much longer than the adjacent denticles and the remaining teeth are devoid of denticles. In *D. cymatilus*, the outermost teeth are elongate but sharply curved whereas in *D. semilunus* they are straighter and more acutely pointed. The rachidian tooth of *D. primus* Baba, 1976 is also narrower than most other *Dermatobranchus* and it has a long, pointed median cusp. However, the first lateral tooth is not denticulate as found in *D. cymatilis.*

The reproductive system is also similar between *D. cymatilus* (Fig. 41) and *D. semilunus* (Fig. 81). In *D. cymatilus* the penial sheath and vagina are far narrower than in *D. semilunus*.

### *Dermatobranchus dendronephthyphagus*sp. nov. (Figs 30H, 42A, 43, 44)

*Dermatobranchus* sp. 15 Gosliner, Behrens & Valdés, 2008: 313, above bottom photo.

*Dermatobranchus nigropunctatus* Baba 1949 (Rudman, 2005), misidentification.

#### 

##### 

###### Type material

Holotype. CASIZ 115751, dissected, Horseshoe Cliffs, 1 km west-north-west of Onna Village, Okinawa, Ryukyu Islands, Japan (26°30.00′N, 127°57.90′E), 43 m depth, collected 13.xii.1996 by R. F. Bolland.

###### Geographical distribution

This species is known from southern Japan and probably also from New South Wales, Australia (Rudman, 2005).

###### External morphology

The body shape of the living animal (Figs 30H, 42A) is elongate, but broad, slightly flattened, and narrows at the posterior end. The foot does not project beyond the mantle margin. The dorsum has approximately 14 well-elevated, longitudinal ridges on either side of the midline that may further branch posteriorly. The oral veil is large and expansive with rounded corners. The well-separated, bulbous rhinophores are situated behind the oral veil. They have a series of longitudinal lamellae on the rounded club, which has a small rounded apex. The stalk does not narrow noticeably. There are noticeable marginal sacs along the mantle edge.

There are no branchial or hyponotal lamellae under the mantle margin. The genital opening is situated in the anterior quarter of the body. The anus is situated approximately half of the way to the posterior end of the body.

The ground colour of the dorsum is white with a series of dark brown to black lines situated between the dorsal ridges. There are areas of denser dark pigment scattered over the surface of the notum. The oral veil has a series of large black spots that are more diffuse around the margins. There is a marginal yellow-orange band on both the notum and the oral veil. The rhinophore stalk is white with black pigment and the club is white with black pigment on the lamellae. The tips of the rhinophores are white.

###### Buccal armature

The shape of the buccal mass is broad and highly muscular. The jaws are large and thickly cuticularized (Fig. 43A), with a thick masticatory margin. The masticatory margin is irregular, with a few rounded tubercles (Fig. 43C). The radular formula is 42 × 85.1.1.1.85 (Fig. 43B). The rachidian teeth (Fig. 43E) are narrow with an elongate, bluntly pointed central cusp that extends beyond the basal portion, which lacks any basal denticles. The first lateral tooth (Fig. 43E) is elongate, with an elongate cusp. No basal denticles are present. The middle and outer lateral teeth (Fig. 43D, E) are elongate and curved and all teeth are smooth and devoid of denticles.

###### Reproductive system (Fig. 44)

The ampulla is thick and simply curved. It bifurcates to the large female gland mass via a short oviduct and to the vas deferens. The majority of the female gland mass is composed of the mucous gland whereas the membrane and albumen glands are much smaller. The prostatic portion of the vas deferens is narrow and slightly convoluted and enters the cylindrical penial sheath. Within the penial sheath, the penis is elongate terminating in a rounded apex. Adjacent to the penis is the wide vagina, which curves and narrows and terminates in a relatively large, spherical bursa copulatrix.

###### Remarks

Externally, *D. dendronephthyphagus* resembles *D. striatus* with its dark brown to black lines and mottling. However, *D. striatus* has orange pigment on its rhinophores and a series of dark brown concentric arches on the oral veil, whereas *D. dendronephthyphagus* has a series of black mottlings. More significantly, the jaws and radula of these species differ markedly. In *D. striatus*, the jaws have a well-developed masticatory margin with several rows of well-developed denticles (Fig. 28B), whereas in *D. dendronephthyphagus* the masticatory margin (Fig. 43C) is entirely smooth with no trace of denticles. The rachidian and inner lateral teeth of *D. striatus* are highly denticulate (Fig. 28) whereas in *D. dendronephthyphagus* (Fig. 43) both the rachidian and inner lateral teeth entirely lack denticles. The reproductive systems of these species also differ. In *D. striatus*, the penial sheath is bulbous (Fig. 29) and the vaginal duct gradually narrows distally. In *D. dendronephthyphagus* (Fig. 44), the penis is cylindrical and the vaginal duct is wide basally with an abrupt narrowing more distally.

Rudman (2005) depicted an animal from New South Wales, Australia, which he identified as *D. nigropunctatus.* However, the specimen differs externally in many respects from *D. nigropunctatus*. *Dermatobranchus nigropunctatus* has white longitudinal lines on the notum with black spots and orange rhinophores whereas *D. dendronephthyphagus* has black lines on a white notum and has white rhinophores with black on the lamellae. Internally, *D. nigropunctatus* (Baba, 1949: fig. 89) has a rachidian and inner lateral tooth with multiple denticles whereas in *D. dendronephthyphagus* these teeth lack denticles. It is likely, based on an identical colour pattern, that Rudman's specimen is identifiable with *D. dendronephthyphagus*.

Similarly, *D. tongshanensis*, *D. multistriatus*, and *D. leoni* have some similarities in colour pattern, but each with different combinations of orange, white, and black pigment (see Remarks for *D. leoni*). These three species also have denticles on the rachidian and inner lateral teeth that are absent in *D. dendronephthyphagus.* In the Remarks section for *D. caeruleomaculatus* it was noted that both that species and *D. dendronephthyphagus* have similarly elongated narrow cusps on their rachidian teeth, but *D. caeruleomaculatus* differs in having basal denticles on the rachidian teeth. Other reproductive differences between the two species are also noted.

### *Dermatobranchus diagonalis*sp. nov. (Figs 42B, 45–47)

*Dermatobranchus* sp.1 Ono, 1999:149, no. 249.

*Dermatobranchus* sp. 3 Gosliner, Behrens & Valdés, 2008: 308, bottom photo.

#### 

##### 

###### Type material

Holotype: CASIZ 174167, Rempi Lagoon, 25 km north of Madang, Papua New Guinea, collected 8.viii.1989 by T. M. Gosliner. Paratypes: CASIZ 068708, three specimens, one dissected, Rempi Lagoon, 25 km north of Madang, Papua New Guinea, 15 m depth, collected 8.viii.1989 by T. M. Gosliner. CASIZ 144082, three specimens, one dissected, south-west side Zamami Island, Kerama Islands, Japan, 5 m depth, collected 30.i.2000 by Atsushi Ono.

###### Geographical distribution

This species is known only from Papua New Guinea and the Kerama Islands of Japan (present study).

###### Etymology

The specific name *diagonalis* refers to the numerous diagonal ridges found on the surface of the notum and is a Latinized noun in apposition.

###### External morphology

The body shape of the living animal (Fig. 42B) is elongate, flattened, and narrows at the posterior end. The foot does not project beyond the mantle margin. There is a series of about 30 shallow dorsal ridges diagonal to the mantle edge, giving the dorsum a distinct leaf-like appearance. Many of these ridges bifurcate along their length. The oral veil extends forward and has rounded corners. The narrow, widely spaced rhinophores are behind the oral veil and are almost cylindrical rather than bulbous. They have a series of longitudinal lamellae on the rounded club. The stalk does not narrow noticeably. Marginal sacs are visible along the mantle edge. Hyponotal and branchial lamellae are absent. The genital opening is on the right side of the body, near the anterior quarter of the body. The anus is situated approximately one**-**third of the way from the posterior end of the body.

The ground colour of the dorsum, the oral veil, and the foot is brown and is much darker along the medial portion of the notum. There are opaque white speckles and spots of various sizes scattered randomly on the dorsum. There is a pale yellow band present along the margin of the notum. The rhinophore stalk is white basally with a brown club with opaque white on the lamellae. Some orange pigment is present just below the opaque white apex. The oral veil is opaque white with a pale orange pigment band along the anterior margin. The dorsal surface of the foot margin is covered with dense reddish brown speckles.

###### Buccal armature

**Figure 45:**
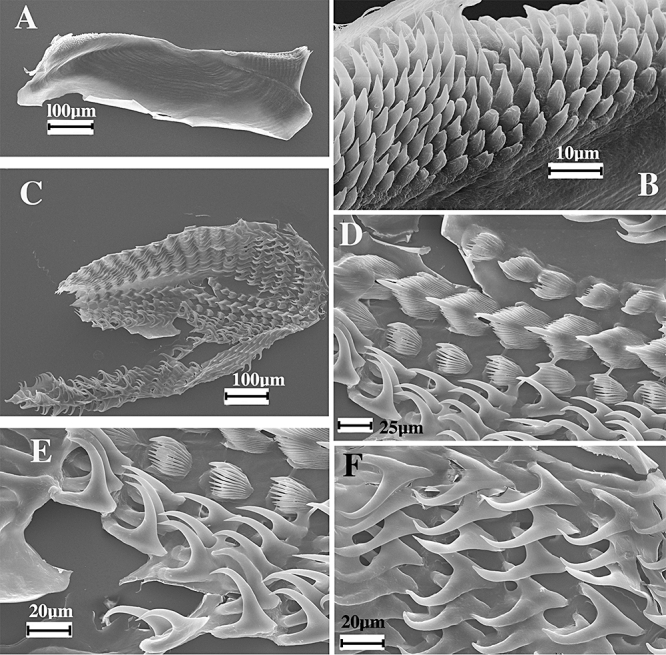
***Dermatobranchus diagonalis* sp. nov.** Buccal armature, CASIZ 070451, Rempi Lagoon, Madang, Papua New Guinea. A, jaw; B, masticatory margin; C, entire radula; D, central portion of radula; E, middle lateral teeth; F, outer lateral teeth.

**Figure 46:**
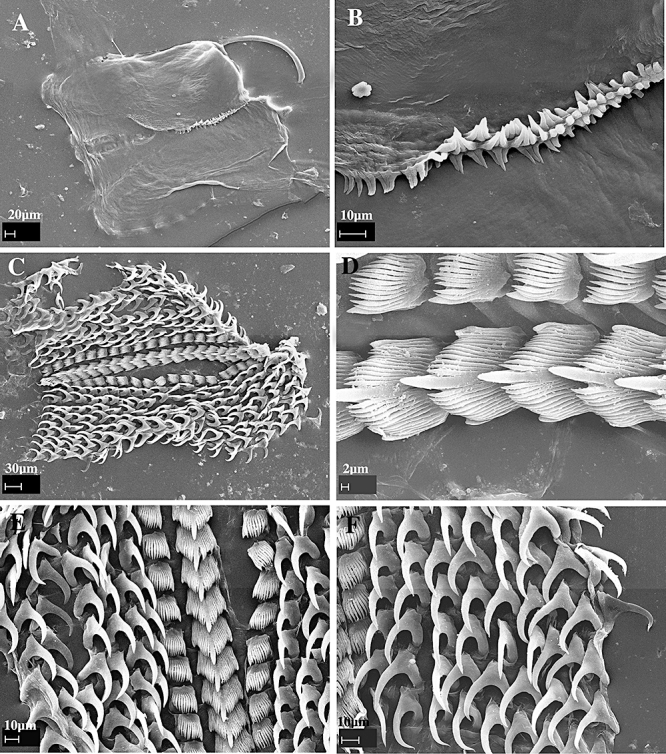
***Dermatobranchus diagonalis* sp. nov.** Buccal armature, CASIZ 144082, Kerama Islands, Japan. A, jaws; B, masticatory margin; C, entire radula; D, central portion of radula; E, half-row of radular teeth; F, outer lateral teeth.

The jaws are large and thickly cuticularized (Figs 45A, 46A), with a thin masticatory margin that has denticles along the lower third of the jaw. Up to nine rows of pointed denticles are largely unifid, with a few bifid ones (Figs 45B, 46B). The entire radula is wider than it is long (Fig. 45C). The radular formula of a paratype (CASIZ 068708) is 22 × 11.1.1.1.11., whereas in a second paratype (CASIZ 144082) it is 21 × 9.1.1.1.9 (Fig. 46C). The rachidian teeth (Figs 45D, 46D) are broad with a large, pointed central cusp, 13–14 elongate flanking denticles on each side. The inner lateral tooth (Figs 45D, E, 46D–F) is a narrow comb with a pointed central cusp with nine to ten pointed denticles. The remaining 11 lateral teeth are hook-shaped without any denticles (Figs 45E, F, 46E, F).

###### Reproductive system

The reproductive organs are androdiaulic (Fig. 47). The hermaphroditic preampullary duct exits from the ovotestis and widens into the ampulla. The entire ampulla is curved and enveloped by the female glands. A short vas deferens widens into a curved prostatic section. The penial sheath is short and conical. Adjacent to the penial sheath is a thin vagina that leads to a relatively small bursa copulatrix inside the folds of the female glands.

###### Remarks

The numerous diagonal ridges of *D. diagonalis* are distinctive and are not found in any other species of *Dermatobranchus*. The colour pattern is most similar to *D. phyllodes*, but that species has short ridges and the ridges are situated perpendicular to the longitudinal body axis. These two species have major differences in radular morphology (see Remarks for *D. phyllodes*).

The radular morphology of *D. diagonalis* is similar to other taxa that have a broad rachidian tooth, and comb-shaped inner lateral teeth and the remaining teeth devoid of denticles (*D. striatus*, *D. albus*, *D. otome*, and *D. oculus*). Members of this group also have numerous rows of acutely pointed denticles on the posterior base of the jaws. None of these species has a colour pattern similar to *D. diagonalis* and they all have longitudinal rather than diagonally radiating notal ridges. The reproductive system of members of this group is similar anatomically. The reproductive system of *D. diagonalis* (Fig. 47) is virtually identical to that of *D. striatus* (Fig. 29), where both species have a short, curved prostate and short, wide conical penial sheath.

### *Dermatobranchus earlei*sp. nov. (Figs 42C, 48, 49)

#### 

##### 

###### Type material

Holotype: CASIZ 097441, one specimen, dissected, 10 mm preserved, Makhallah Bay, Arabian Sea, Oman, 10 m depth, collected 30.x.1993 by J. Earle.

###### Geographical distribution

This species is only known from Oman (present study).

###### Etymology

The specific name *earlei* is named for John Earle, who provided the type specimen of this species from the Arabian Sea.

###### External morphology

The body shape of the living animal (Fig. 42C) is wedge-shaped (wide anteriorly and gradually narrowing at the posterior end, similar in body shape to that of most species of *Armina*). The foot does not project beyond the distinct mantle margin. The dorsum has very low, broken ridges that angle from along the midline to the mantle margin. The oral veil projects only slightly at the anterior end. The rhinophores are situated behind the oral veil. They have a series of longitudinal lamellae on the rounded club. The stalk does not narrow noticeably and the club has a small projecting tip. Marginal sacs are visible along the mantle edge.

There are no longitudinal hyponotal or branchial lamellae under the mantle margin. The genital opening is situated at the anterior quarter of the body on the right side. The anus is situated approximately half of the way to the posterior end of the body.

The ground colour of the dorsum and the foot is pale blue. On the mantle, there are several distinct areas of brown pigment. The dorsal ridge crests are tan. The mantle margin is pale white. The rhinophore stalk is pale white with tan pigment. The club is black. The tip of the rhinophores is white. The oral veil is pale white with an orange margin.

###### Buccal armature

The jaws are large and thickly cuticularized (Fig. 48A), with a thick masticatory margin and a row of irregular denticles (Fig. 48B). The radular formula of the holotype is 4.5.1.1.1.5.4. The entire radula was not observed; thus, the number of rows of teeth could not be determined. The rachidian teeth (Fig. 48C) are broad with a large, spear-shaped central cusp and ten flanking denticles on each side. The base of each rachidian has two humps, one on either side at the upper edge. The first lateral tooth (Fig. 48C) is a broad comb with a blunt pointed base and 9–12 pointed denticles. The next four to five lateral teeth are comb-shaped with a longer, pointed first denticle and up to 4–11 long pointed denticles (Fig. 48D). The last four lateral teeth are hooks without denticles.

###### Reproductive system

The reproductive organ arrangement is androdiaulic. The elongate hermaphroditic duct leads into the wide, tubular ampulla (Fig. 49). The ampulla bifurcates into the female gland mass via a short oviduct, and into the short, curved prostate. The prostate expands into the wide, bulbous penial sheath. From the large, round bursa copulatrix the short, narrow vaginal duct leads to the slightly wider vagina. The vagina exits into the genital atrium next to the penial sheath.

###### Remarks

Externally, *D. earlei* most closely resembles *D. caesitius* from South Africa. See the Remarks section of *D. caestitius* for comparison of these taxa.

*Dermatobranchus earlei* is internally similar to other species with an elongate radula, which has relatively few teeth per row. These taxa include *D. substriatus* Baba, 1949, *D. striatellus* Baba, 1949, *D. fortunatus*, *D. microphallus* sp. nov., *D. funiculus* sp. nov., *D. rodmani* sp. nov., *D. piperoides* sp. nov., and *D. kokonas* sp. nov. None of these species has a bluish colour pattern as in *D. earlei*. Of these species, *D. fortunatus*, *D. piperoides*, and *D. kokonas* lack prominent dorsal ridges that are evident in *D. earlei* and the remaining taxa. Both *D. microphallus* and *D. rodmani* have less prominent ridges than those of *D. earlei*. In *D. substriatus*, *D. striatellus*, and *D. funiculus*, the ridges are less crowded, wider, and continuous rather than interrupted, whereas in *D. earlei* they are narrow, congested, and frequently interrupted.

### *Dermatobranchus fasciatus*sp. nov. (Figs 42D, 50–53)

*Dermatobranchus* sp. 6 Gosliner, Behrens & Valdés, 2008: 310, bottom photo.

#### 

##### 

###### Type material

Holotype: CASIZ 174140, 5 mm preserved, Pamilacan Island off Bohol Island, Panglao, Philippine Islands (9°29.4′N, 123°56.0′E), coral plateau with fine sand, 6–8 m depth, collected 14.vi.2004 by T. Gosliner, Y. Camacho, J. Templado, M. Malaquias, M. Poddubetskaia. Paratypes: CASIZ 171384, three specimens, 5–6 mm, two specimens, both 6 mm, dissected, Pamilacan Island off Bohol Island, Panglao, Philippine Islands (9°29.4′N, 123°56.0′E), coral plateau with fine sand, 6–8 m depth, collected 14.vi.2004 by T. Gosliner, Y. Camacho, J. Templado, M. Malaquias, M. Poddubetskaia. CASIZ 171387, two specimens, 7–15 mm preserved, one dissected, Pamilacan Island, off Bohol Island, Panglao, Philippine Islands (9°29.4′N, 123°56.0′E), coral plateau with fine sand, 6–14 m depth, collected 11.vi.2004 by T. Gosliner, Y. Camacho, J. Templado, M. Malaquias, M. Poddubetskaia. CASIZ 073049, one specimen, 10 mm preserved, Daphne's Reef, north coast of Madang, Papua New Guinea, 13 m depth, collected 7.x.1986 by T. Gosliner.

###### Geographical distribution

This species is known from the Philippine Islands and Papua New Guinea (present study).

###### Etymology

The specific name *fasciatus* is a noun in apposition, from the Latin word meaning ‘band’. This is in reference to the distinguishing dark band across the notum of this species.

###### External morphology

The body shape of the living animal (Fig. 42D) is elongate, flattened, and narrows at the posterior end. The foot does not project beyond the distinct mantle margin. There is a series of low longitudinal dorsal ridges. The oral veil extends forward and the corners protrude slightly. The rhinophores are situated behind the oral veil. They have a series of longitudinal lamellae on the rounded club. The stalk does not narrow noticeably. No marginal sacs were visible. There are no branchial or hyponotal lamellae under the mantle margin. The genital opening is situated about one-quarter of the distance along the anterior body side. The anus is situated approximately half of the way to the posterior end of the body. The ground colour of the dorsum and the foot is opaque white with dark spots scattered randomly. Both the mantle and foot have a pink edge. On the mantle, there are two transverse bands of tan coloration with darker spots scattered randomly within the bands. The bands of dark colour divide the dorsum into approximate thirds. The dorsal ridges are the same white as the ground colour and the depressions between the ridges are grey. The rhinophore stalk is white with some random dark spots and the club is dark brown to black with opaque white spots. The oral veil is opaque white with dark spots and a pink margin.

###### Buccal armature

The buccal mass is thick and muscular. The jaws are large and thickly cuticularized (Figs 50A, 51A), with a thick masticatory margin and five to six rows of long, pointed denticles (Figs 50B, 51B). The radular formula of the holotype (CASIZ 171384) is 26 × 18.1.1.1.18 (Fig. 52C), whereas in two paratypes the formulae are (CASIZ 073049) 21 × 18.1.1.18 (Fig. 51C) and (CASIZ 171387) 26 × 23.1.1.1.23 (Fig. 52A).

The rachidian teeth (Figs 50D, 51D, 52B) are broad with a large, pointed central cusp that is twice as long and wide as the 11–14 flanking denticles on each side. The outer edge of each rachidian tooth is rounded with a wide notch near its centre. The inner lateral tooth (Figs 50D, 51D, 52B) has a broad base and 11 pointed denticles that are equal in length. The next 18 lateral teeth have a comb-like appearance with a broad base and a projecting hook with 15–16 denticles (Figs 50E, 51E, 52C). The outermost one to three teeth are curved, acutely pointed, and lack denticles (Figs 50F, 51F, 52D).

###### Reproductive system

The reproductive organ arrangement is androdiaulic. It was examined in two specimens (CASIZ 171384 and 171387) (Fig. 53). The latter specimen is larger, more mature, and is illustrated. The former had the same general morphology but had a less well-developed female gland mass and a less convoluted prostate. In the more mature specimen, the hermaphroditic duct leads into the long, curved, ovoid ampulla. The ampulla bifurcates into the female gland mass via a short oviduct and into the thick, tubular prostate, which forms one complete loop before it enters the wide elongate penial sheath. The round bursa copulatrix is larger than the ampulla. From the bursa, the long, narrow vaginal duct extends into an even narrower vagina, which exits into the genital atrium next to the penial sheath.

###### Remarks

Externally, *D. fasciatus* (Fig. 42D) most closely resembles *D. semilunus* (Fig. 74C–E) from the western Pacific. Both species have an opaque white ground colour with a band of tan or white across the dorsum. *Dermatobranchus semilunus* has a single u-shaped brown marking whereas *D. fasciatus* has two broader bands of brown. The rhinophores of *D. fasciatus* are dark brown to black with opaque white spots, whereas those of *D. semilunus* have white lines along each vertical lamella.

The radular morphology of *D. fasciatus* and *D. semilunus* is markedly different. *Dermatobranchus fasciatus* (Figs 50–52) has fewer radular teeth per half row (18–23) than does *D. semilunus* (29–73; Figs 78–80). The rachidian tooth of *D. fasciatus* is wide with a wide central cusp and 11–14 denticles on either side. The rachidian of *D. semilunus* is much narrower with up to six on either side. The inner lateral teeth of *D. fasciatus* are broad and laterally directed with a series of denticles above a triangular cusp whereas in *D. semilunus*, there are three to four denticles on the outer side of an elongate central cusp. In *D. fasciatus*, the majority of teeth are denticulate, whereas in *D. semilunus* all of the remaining teeth other than the rachidian and first lateral teeth are smooth.

The reproductive system of these two species has some similarities such as the tubular ampulla and a thickened prostate. However, the prostate of *D. fasciatus* (Fig. 53) is more elongate and convoluted than that of *D. semilunus* (Fig. 81). Additionally, the vagina of *D. semilunus* widens near the genital aperture, whereas that of *D. fasciatus* is narrow near the aperture.

**Figure 82:**
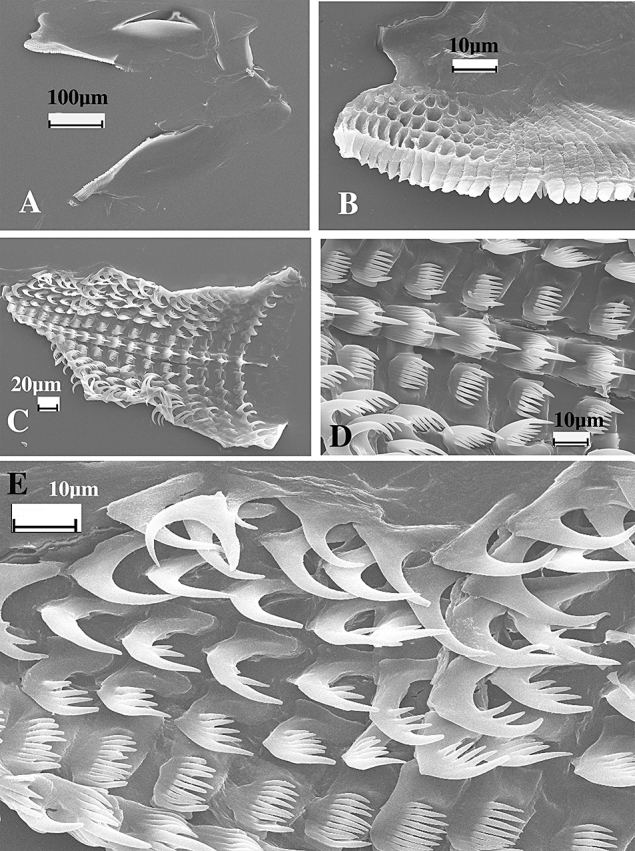
***Dermatobranchus tuberculatus* sp. nov.** Buccal armature, CASIZ 103780, Ligpo Island, Balayan Bay, Luzon, Philippines. A, jaws; B, masticatory margin; C, entire radula; D, central portion of radula; E, outer radular teeth.

**Figure 84:**
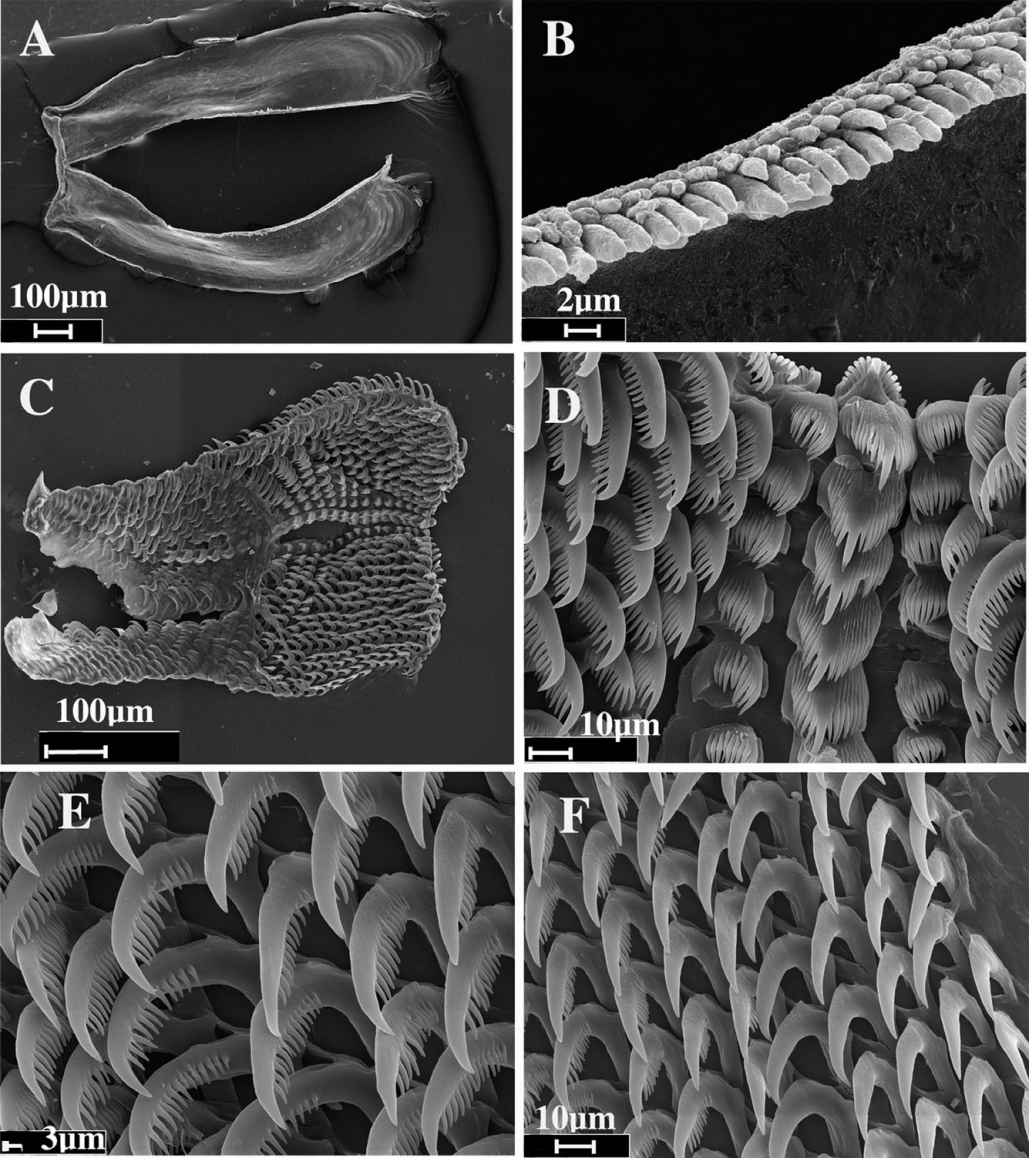
***Dermatobranchus tuberculatus* sp. nov.** Buccal armature, CASIZ 173400, Tokong Kamundi, Malaysia. A, jaws; B, masticatory margin; C, entire radula; D, central portion of radula; E, middle radular teeth; F, outer radular teeth.

*Dermatobranchus fasciatus* also bears an external and internal resemblance to some specimens of *D. tuberculatus*. Both species have a whitish body with some transverse brown areas on the notum. *Dermatobranchus tuberculatus* has irregular tubercles on the notum, but lacks distinct longitudinal ridges. The rhinophores are lighter in colour in *D. tuberculatus* (Fig. 74F–H) than in *D. fasciatus* and lack the opaque spots on the upper surface of the rhinophore club that are present in *D. fasciatus*. The radula of *D. fasciatus* is also similar in morphology to that of *D. tuberculatus*. Both species have a rachidian tooth with long lateral denticles. The central cusp of *D. fasciatus* has a much wider base than that found in *D. tuberculatus*. The rachidian tooth of *D. tuberculatus* (Figs 82D, 83E, 84D) is narrower than that of *D. fasciatus* and has six to ten denticles, whereas there are 14–18 denticles in *D. fasciatus*. Similarly, the posterior notch of *D. tuberculatus* is much narrower that that of *D. fasciatus*. The inner lateral teeth of *D. tuberculatus* are broad with a distinct prominent cusp at the same height as the adjacent denticles. In *D. fasciatus*, the cusp is located separately, below the level of the adjacent denticles. The second lateral tooth of *D. tuberculatus* has 5–11 denticles whereas that of *D. fasciatus* has 15–17 denticles. In *D. tuberculatus* the outer three to four teeth lack denticles whereas in *D. fasciatus* only the outer one to three teeth are smooth.

The reproductive system of *D. fasciatus* (Fig. 53) differs from that of *D. tuberculatus* (Fig. 85). In *D. fasciatus*, the vagina is straight and narrow and nears further near the genital aperture. In *D. tuberculatus*, the vagina is convoluted and has a thicker basal portion and a thinner distal portion that connects to the bursa copulatrix. Additionally, the prostate of *D. tuberculatus* is longer and more highly convoluted than that of *D. fasciatus*.

### *Dermatobranchus funiculus*sp. nov. ( Figs 42E–H, 54–57)

*Dermatobranchus* sp. 9 Gosliner, Behrens & Valdés, 2008: 311, bottom photo.

*Dermatobranchus* sp. 18 Gosliner, Behrens & Valdés, 2008: 314, below top photo.

*Dermatobranchus* sp. 21 Gosliner, Behrens & Valdés, 2008: 315, top photo.

*Dermatobranchus* sp. Coleman, 2008: 122, as purple spots *Dermatobranchus*.

*Dermatobranchus* sp. Coleman, 2008: 123, as Milne Bay *Dermatobranchus*.

#### 

##### 

###### Type material

Holotype: CASIZ 177375, subsampled for DNA, Bethlehem, Maricaban Island, Batangas Province, Luzon, Philippines (13°40.3338′N, 120°50.51166′E), 19 m depth, collected 18.iii.2008 by T. Gosliner *et al.* Paratypes: CASIZ 177379, one specimen, subsampled for DNA, Bethlehem, Maricaban Island, Batangas Province, Luzon, Philippines (13.672230°N, 120.841861°E), 19 m depth, collected 18.iii.2008 by T. Gosliner *et al.* CASIZ 177377, one specimen, dissected, Bethlehem, Maricaban Island, Batangas Province, Luzon, Philippines (13.672230°N, 120.841861°E), 19 m depth, collected 18.iii.2008 by T. Gosliner *et al.* CASIZ 177608, one specimen, subsampled for DNA, Bethlehem, Maricaban Island, Batangas Province, Luzon, Philippines (13.672230°N, 120.841861°E), 21 m depth, collected 17.iv.2008 by T. Gosliner. CASIZ 177413, one specimen, subsampled for DNA, Bethlehem, Maricaban Island, Batangas Province, Luzon, Philippines (13.672230°N, 120.841861°E), 21 m depth, collected 19.iii.2008 by T. Gosliner *et al.* CASIZ 104726, five specimens, 5–9 mm, two 9 mm specimens dissected, Tengan Pier, Ishikawa City, Okinawa, Ryukyu Islands, 13 m depth, collected 9.vii.1991 by R. Bolland. CASIZ 079259, five specimens, two dissected, Tengan Pier, Ishikawa City, Okinawa, Ryukyu Islands, Japan, 13 m depth, collected 9.vii.1991 by R. Bolland.

###### Geographical distribution

This species is known only from Okinawa, the Philippines (present study), and Papua New Guinea (Coleman, 2008).

###### Etymology

The specific name *funiculus* is from the Latin word for ‘thin rope or cord’ to describe the appearance of the longitudinal ridges of this species.

###### External morphology

The body shape of the living animal (Fig. 42E–H) is elongate, flattened, and narrows at the posterior end. The dorsal ridges are nearly parallel along the midline, but angle towards the mantle margin along the sides of the notum. The foot does not project beyond the distinct mantle margin. There is a series of 12–23 longitudinal dorsal ridges. The oral veil extends forward and has blunt extensions at the corners. The wide-spaced rhinophores are behind the oral veil. They have a series of longitudinal lamellae on the rounded club. The stalk does not narrow noticeably. There are visible marginal sacs along the mantle edge. The genital opening is on the right side of the anterior quarter of the body. The anus is situated approximately one third of the way to the posterior end of the body. There are no branchial or hyponotal lamellae under the mantle margin. The ground colour of the dorsum and dorsal ridges is white to bluish or orange. The oral veil and the foot are opaque white with an orange frontal margin. The depressions between the dorsal ridges are light grey or orange with dark dots. There are dark bands of colour across the notum, beginning approximately halfway from the anterior end. Along the mantle edge are evenly spaced, dark spots of colour. The mantle has a pinkish-orange border. The rhinophore stalk is white and the club is dark brown with opaque white to cream lamellae and apex.

###### Buccal armature

**Figure 54:**
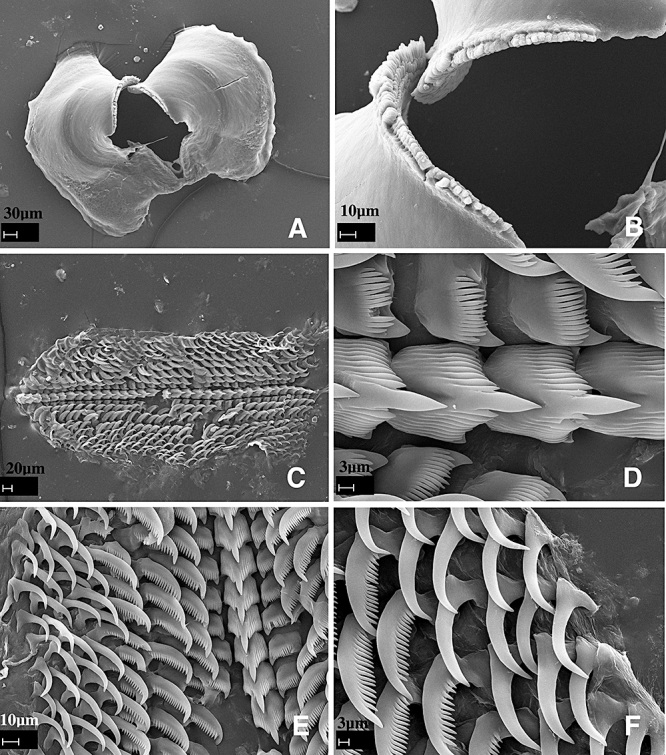
***Dermatobranchus funiculus* sp. nov.** Buccal armature, CASIZ 104726. Okinawa, Japan. A, jaws; B, masticatory margin; C, entire radular width; D, central portion of the radula; E, half-row of radular teeth; F, outer lateral teeth.

**Figure 55:**
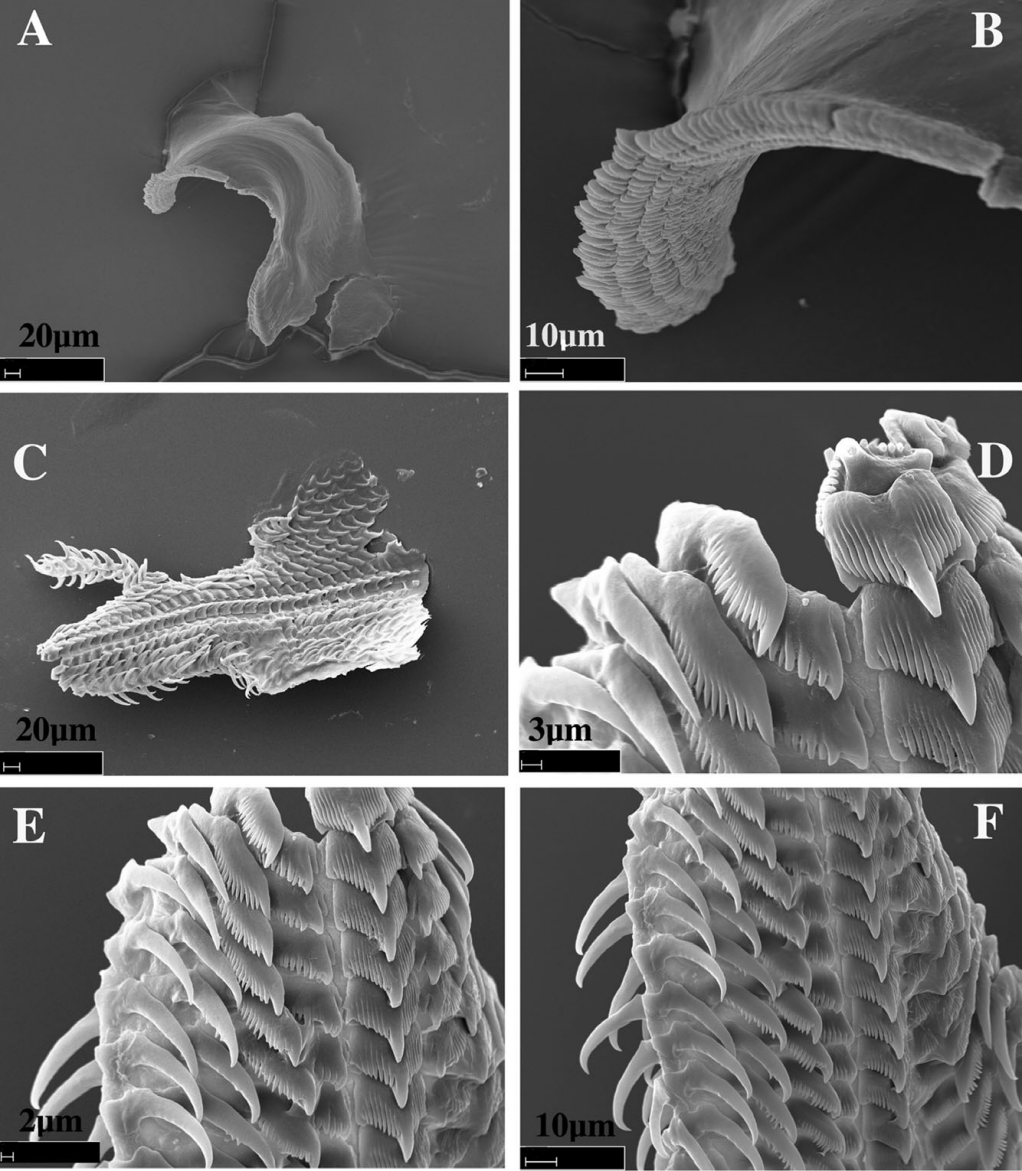
***Dermatobranchus funiculus* sp. nov.** Buccal armature, CASIZ 177377, Maricaban Island, Luzon, Philippines. A, jaw; B, masticatory margin; C, entire radular width; D, central portion of the radula; E, F, half-row of radular teeth.

**Figure 56:**
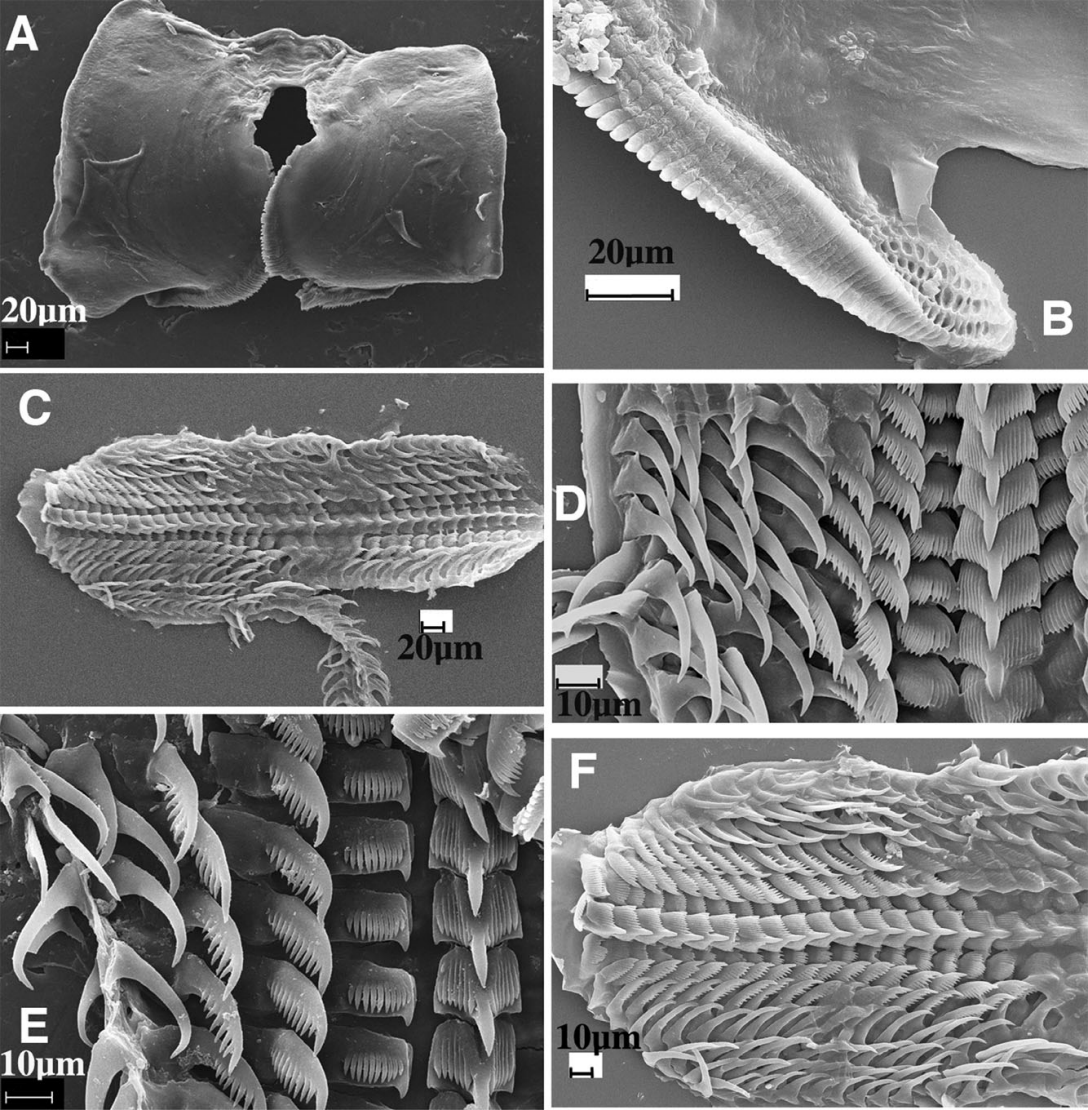
***Dermatobranchus funiculus* sp. nov.** Buccal armature, CASIZ 079259, Okinawa, Japan. A, jaws; B, masticatory margin; C, entire radular width; D, central portion of the radula; E, half-row of radular teeth; F, outer lateral teeth.

The buccal armature was examined in five paratype specimens [CASIZ 104726, 177377 (two specimens), 079259 (two specimens)]. The jaws are large and thickly cuticularized (Figs 54A, 55A, 56A), with a thick masticatory margin and five to seven rows of long, pointed denticles (Figs 54B, 55B, 56B). The radula is long and narrow (Figs 54C, 55C, 56C) with a formula in four paratypes of +17 × 6.1.1.1.6 (CASIZ 079259), 33 × 6.1.1.1.6 (CASIZ 079259), 34 × 6–8.1.1.1.6–8 (CASIZ 104726), and 31 × 6.1.1.1.6 (CASIZ 177377). The rachidian teeth (Figs 54D, E, 55D–F, 56D–F) have a broad base with a large, thick, and long central cusp that is wider than the six to nine flanking denticles on each side. The inner lateral tooth (Figs 54D, E, 55D–F, 56D–F) has a wide base with a prominent cusp with 7–16 short, narrow, pointed denticles on the outside surface of the base. The next two lateral teeth are distinct, having a wide base, a long, projecting hook-shaped first denticle and up to 16 smaller, pointed denticles. The last four lateral teeth (Figs 54F, 55E, F, 56D–F) are hook-shaped without denticles.

###### Reproductive system (Fig. 57)

The reproductive organ arrangement is androdiaulic. The hermaphroditic duct leads into the wide ampulla. The ampulla is thick and simply curved. It bifurcates into the large female gland mass via a short oviduct and the vas deferens. The majority of the female gland mass is composed of the mucous gland whereas the membrane and albumen glands are much smaller. The prostatic portion of the vas deferens is relatively narrow and slightly convoluted and widens slightly as it enters the elongate, bulbous penial sac. Within the penial sac, the penis is elongate and wide distally terminating in a rounded apex. Adjacent to the penis is the thin, straight vagina, which terminates in a relatively large, pyriform bursa copulatrix. The vagina is about the same diameter throughout.

###### Remarks

Externally, *D. funiculus* looks most similar to two species from Japan, *D. cymatilis* (see the Remarks above for *D. cymatilis*) and *D. striatellus* Baba, 1976. Other *Dermatobranchus* species have the dark bands of colour across the notum, such as *D. albus* and *D. otome* Baba, 1992, but *D. albus* can have tiny dark spots within the band of colour, and the dorsal ridges are white or orange, as are the depressions between the ridges. *Dermatobranchus otome* has ocellated spots on the dorsal ridges whereas *D. funiculus* does not. The dorsal ridges of *D. striatellus* are arranged in a reticulated pattern and there are no dark bands of colour perpendicular to the dorsal ridges as found in *D. funiculus.* There are tiny black dots of colour on the dorsum of *D. funiculus* and the rhinophore club is dark brown. This is in contrast to *D. striatellus* that has no dark dorsal dots and has orange red rhinophores.

*Dermatobranchus funiculus* (Figs 54–56) has some similarities in radular morphology to *D. striatellus* (Baba, 1949: text fig. 86). For example, both species have a broad-based rachidian tooth with a projecting, spear-shaped median cusp. Both have a similarly shaped, denticulate first lateral tooth. However, in *D. funiculus*, the second and third lateral teeth are denticulate, unlike the simple hooks of *D. striatellus.* The radular formula of *D. striatellus* is 32 × 11–12.1.1.1.11–12 in contrast to *D. funiculus* with a radular formula of 31–34 × 4.2.1.1.1.2.4. Neither of the externally similar species *D. otome* nor *D. albus* have distinctive, denticulate second and third lateral teeth, whereas *D. funiculus* does.

Baba (1992) did not describe the reproductive system of *D. otome*. However, we examined specimens of *D. otome* (Fig. 21), and there are several differences. In *D. otome* the prostate is more convoluted than in *D. funiculus* (Fig. 57), and the bursa copulatrix duct of the former is more elongate and curved. Additionally, a comparison can be made between *D. funiculus* and *D. albus* (Fig. 11). Both species have similar features such as a wide ampulla, narrow vaginal duct, and a large, bulbous penial sheath. However, the hermaphroditic duct is comparatively longer in *D. albus*, the ampulla is much larger and more tubular than that of *D. funiculus.* In addition, the penial sheath in *D. funiculus* is much larger, proportional to the other reproductive organs than is the penial sheath in *D. albus*. Finally, the prostate of *D. funiculus* is longer and more coiled than that of *D. albus*.

*Dermatobranchus funiculus* should be compared to other species with a long narrow radula, namely *D. fortunatus*, *D. substriatus*, *D. earlei*, *D. kokonas*, *D. piperoides*, *D. rodmani*, and *D. albineus*. *Dermatobranchus fortunatus*, *D. rodmani*, *D. kokonas*, and *D. piperoides* all lack longitudinal ridges and have very different colour patterns from *D. funiculus*.

Of the species with longitudinal ridges all differ consistently from *D. funiculus*. The external coloration of *D. substriatus* remains unknown. However, it has fewer (nine) longitudinal and diagonal ridges than does *D. funiculus* (12). Internally, the radula of *D. substriatus* has few teeth per row (nine) versus 15 in *D. funiculus*. The reproductive anatomy of *D. substriatus* remains unknown. Further comparison of the two species must await the rediscovery of additional material from the type locality of *D. substriatus* (Sagami Bay region of Japan).

The colour of *D. albineus* is very different from that of *D. funiculus*. It has pink pigment between the white ridges as compared to the grey of *D. funiculus*. Internally, *D. albineus* has many more (13–17) outer lateral teeth per row than does *D. funiculus* (six).

*Dermatobranchus earlei* has a bluish body colour with brown pigment on the notum. The central cusp of the rachidian tooth of *D. earlei* is much broader than that of *D. funiculus* and there are more lateral teeth per half row of *D. earlei* (nine) than in *D. funiculus* (six). In the reproductive system of *D. funiculus* (Fig. 57) the prostate is more convoluted than in *D. earlei* (Fig. 49).

### *Dermatobranchus kalyptos*sp. nov. (Figs 58A, 59, 60)

*Dermatobranchus* sp. 1 Gosliner, Behrens & Valdés, 2008:308, second photo.

#### 

##### 

###### Type material

Holotype: CASIZ 174139, 40 mm preserved, east side Lombok Island, Indonesia, 10 m depth, collected 23.xi.1995 by P. Fiene.

###### Paratypes

CASIZ 107424, two specimens, 28–30 mm, both dissected, east side Lombok Island, Indonesia, 10 m depth, collected 23.xi.1995 by P. Fiene.

###### Geographical distribution

This species is known from Indonesia (present study) and possibly Australia (Coleman, 2008, as warty *Dermatobranchus*).

###### Etymology

The specific name *kalyptos* is the Greek word meaning to cover or conceal, to hide, or a covering. This is in reference to the camouflage colour pattern of this species that allows it to blend in with the substrate.

###### External morphology

The body shape of the living animal (Fig. 58A) is elongate and narrows at the posterior end. The foot does not project beyond the distinct mantle margin. The dorsum has no ridges but has irregularly-spaced dorsal humps. There are some rounded tubercles irregularly spaced along the posterior mantle sides. The oral veil projects forward. The rhinophores are situated behind the oral veil and appear to be partially joined at their inner base. They have a series of longitudinal lamellae on the rounded club. The stalk does not narrow and the club has a small projecting tip. There are visible marginal sacs along the mantle edge.

There are no longitudinal branchial or hyponotal lamellae under the mantle margin. The genital opening is situated near the oral veil. The anus is situated approximately half of the way to the posterior end of the body.

The ground colour of the dorsum and the foot is pale blue or lavender. On the mantle, there are several large patches of white and tan pigment and some raised brown areas. There are some smaller, dark brown spots scattered randomly. Around the rhinophores are mottled patches of light brown. The rhinophore stalk is opaque white with a dark line on the anterior side. The club is dark brown and the tip is white. The oral veil is white with a mix of blue and brown spots. On the ventral side of the mantle and on the foot are small blotches of dark pigment.

###### Buccal armature

The jaws are large and thickly cuticularized (Fig. 59A, B), with a thick masticatory margin and multiple rows of long, pointed denticles (Fig. 59C). The radular formula of one of the paratypes (CASIZ 107424) is 40 × 57.1.1.1.57 (Fig. 59D). The rachidian teeth (Fig. 59E) have a broad base with a large, pointed central cusp, which projects beyond the 14–21 flanking denticles on each side. The inner lateral tooth is claw-shaped with up to 8–14 denticles that are nearly equal in length. The next 17 lateral teeth are long and pointed with 23–27 denticles. The remaining 40 lateral teeth (Fig. 59F) are long, pointed hooks without denticles.

###### Reproductive system

The reproductive organ arrangement is androdiaulic. The hermaphroditic duct leads into the long, tubular ampulla (Fig. 60). The ampulla bifurcates into the female gland mass via a short oviduct and into the very long, coiled prostate. The prostate twists and spirals several times then expands into the wide, bulbous penial sheath. From the large, round bursa, the long, narrow vaginal duct extends into the slightly wider vagina, which exits into the genital atrium adjacent to the penial sheath.

###### Remarks

Externally, *D. kalyptos* most closely resembles *D. pustulosus* from the Philippine Islands and Indonesia. Both species have a tuberculate dorsum and some pale blue or lavender pigment. However *D. pustulosus* has low, broken ridges whereas *D. kalyptos* does not. The rhinophores of *D. pustulosus* are lighter and more bulbous than those of *D. kalyptos*. Internal differences between these species are included in the Remarks section for *D. pustulosus*.

### *Dermatobranchus kokonas*sp. nov. (Figs 58B, 61–62)

*Dermatobranchus* sp. 14 Gosliner, Behrens & Valdés, 2008: 313, below top photo.

#### 

##### 

###### Type material

Holotype: CASIZ 075272, one specimen, dissected, Christmas Bay, Bagabag Island, Madang, Papua New Guinea (4°49.15416′S, 146°13.98558′E), 20 m depth, collected 26.xi.1990 by T. M. Gosliner.

###### Geographical distribution

This species is known only from the north coast of Papua New Guinea (present study).

###### Etymology

The specific name *kokonas* comes from the New Guinea pidgin word kokonas, for coconut, because of the resemblance of the colour and texture of the notum of this animal to the meat of the coconut.

###### External morphology

The body shape of the living animal (Fig. 58B) is slender, elongate, flattened, and narrows to the rounded posterior end. The foot does not project beyond the distinct mantle margin. Its mantle is devoid of longitudinal ridges and small, low, randomly distributed conical tubercles are present on the surface. The oral veil extends forward and is rounded at the corners. The rhinophores are situated behind the oral veil. They have a series of longitudinal lamellae on the rounded club with a short extension apically. The stalk narrows slightly. There are marginal sacs on the underside of the mantle edge. The genital opening is about halfway along the body on the right side. The anus is situated approximately half of the way towards the posterior end of the body. There are no branchial or hyponotal lamellae under the mantle margin.

The ground colour of the dorsum, foot and oral veil is opaque white as are the tubercles. A few brown spots are present on the notum. The rhinophore stalk is white and the club is brown. The oral veil is opaque white with a bright orange margin.

###### Buccal armature

**Figure 61:**
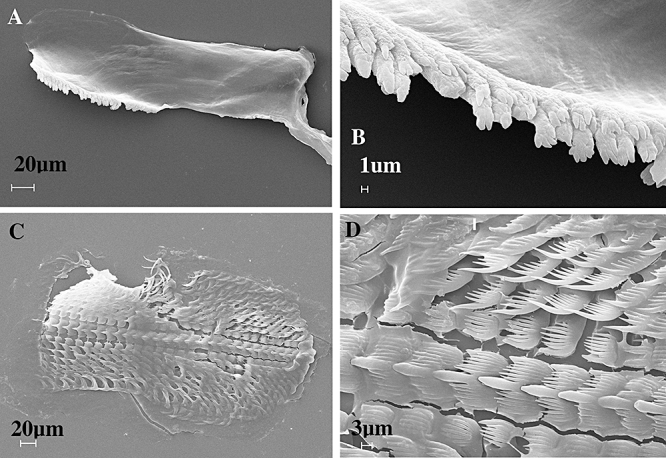
***Dermatobranchus kokonas* sp. nov.** Buccal armature, CASIZ 075272, Bagabag Island, Papua New Guinea. A, jaw; B, masticatory margin; C, entire radula; D, central portion of radula.

The cuticular jaws (Fig. 61A) are thin and elongate with one to two rows of highly divided denticles (Fig. 61B) along the lower third of the jaw. The radular formula of the holotype (CASIZ 075272) is 28 × 9.1.1.1.9 (Fig. 61C). The rachidian teeth (Fig. 61D) are broad with a broad base and an elongate, wide central cusp. The cusp is flanked by seven to eight elongate denticles on either side. The inner lateral tooth is broad with five to seven elongate denticles on the outer side of the central cusp. The next five laterals bear one to seven denticles on the outer side of the sharp cusp. The outer two teeth lack denticles (Fig. 61C).

###### Reproductive system

**Figure 62:**
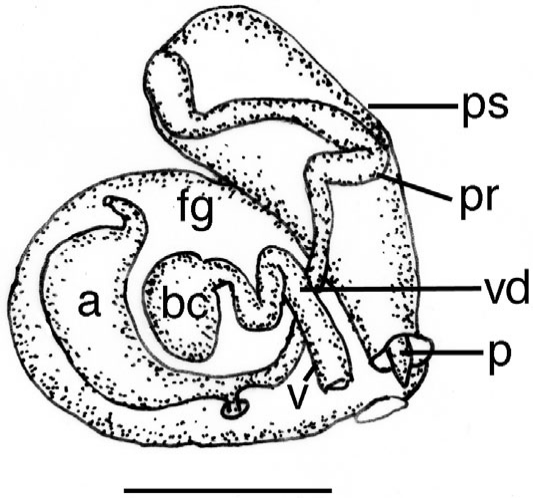
***Dermatobranchus kokonas* sp. nov.** Reproductive system, CASIZ 075272, Bagabag Island, Papua New Guinea. a, ampulla; bc, bursa copulatrix; fg, female gland mass; p, penis; pr, prostate; ps, penial sheath; v, vagina; vd, vaginal duct. Scale bar = 0.67 mm.

The reproductive organs are androdiaulic (Fig. 62). The ampulla is curved and saccate, branching to the female gland mass via a short oviduct and as a narrow, elongate convoluted vas deferens that terminates in a large, broad penial sheath. The penial papilla has an acutely pointed apex. The well-developed female gland mass is well differentiated into a large mucous gland and smaller albumen and membrane glands. Adjacent to the penis is a thin convoluted vagina that leads to a relatively small bursa copulatrix inside the folds of the female glands.

###### Remarks

Externally, this species is similar to white forms of *D. albus*, with an opaque white notum and an orange line on the edge of the oral veil. However, *D*. *kokonas* lacks longitudinal ridges and lacks the orange pigment on the apices of the rhinophores and along the notal margin. Internally, the two species differ in the dentition of the jaws. Both have elongate jaws with denticles near the basal portion opposite the hinged junction of the jaws. In *D. albus*, there are six to seven rows of acutely pointed denticles (Figs 8B, 10A), whereas in *D*. *kokonas* there are two rows with multifid apices (Fig. 61A, B). Both species have a narrow radula with a multidenticulate rachidian and a broad, denticulate inner lateral. In *D. albus*, the remaining laterals are all hammate without denticles, whereas in *D*. *kokonas*, the first six laterals are denticulate with only the outer two being smooth. The penial sheath is proportionately much longer in *D. kononas* than in *D. albus*, as is the prostate.

**Figure 69:**
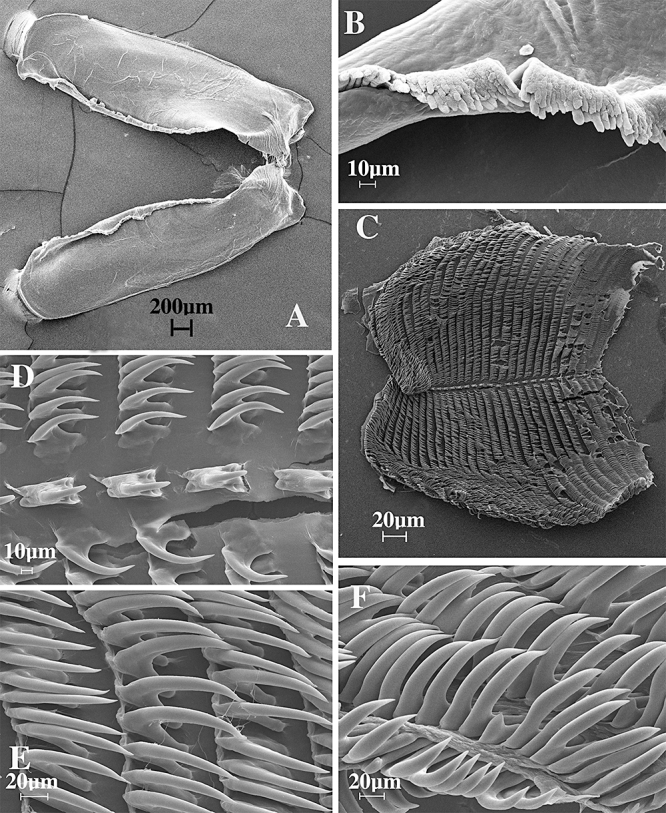
***Dermatobranchus phyllodes* sp. nov.** Buccal armature, CASIZ 070451, Bagabag Island, Papua New Guinea. A, jaws; B, masticatory margin; C, entire radula; D, central portion of radula; E, middle lateral teeth; F, outer lateral teeth.

The jaws and radula of *D. kokonas* have distinctive characteristics in common with several other species of *Dermatobranchus*: *D. fortunatus*, *D. earlei*, *D. rodmani*, *D. piperoides*, and *D. microphallus*. The jaws of all of these species have one or two rows of masticatory denticles with divided apices. Additionally, all of these species have an elongate radula with a wide inner lateral tooth. Two of these species, *D. earlei* and *D. microphallus*, have prominent longitudinal ridges, whereas the other species lack ridges. *Dermatobranchus rodmani* may have three low ridges that are not always evident. Of the species without ridges, *D. kokonas* is the only species with a uniformly white body colour. The only other white species is *D. piperoides*, which also has black spots on the body and black apices on the orange rhinophores, whereas *D. kokonas* has brown rhinophores. The oral veil of *D. kokonas* has a fine orange marginal line whereas in *D. piperoides* there is an orange spot over much of the anterior portion of the veil. The radular configuration also differs between the two species. In *D. kokonas* (Fig. 61) there are nine outer lateral teeth per side whereas in *D. piperoides* (Fig. 69) there are five to six teeth per side. The inner lateral teeth of *D. kokonas* have five to seven denticles, whereas there are 14–15 denticles on those of *D. piperoides*. Differences in the reproductive system also separate these two species. In *D. kokonas*, the prostate is narrow and elongate, whereas in *D. piperoides* it is shorter and thicker. The penial papilla of *D. kokonas* is elongate and conical whereas it is shorter and blunter in *D. piperoides*.

### *Dermatobranchus leoni*sp. nov. (Figs 58C, 63, 64)

*Dermatobranchus* sp. 19 Gosliner, Behrens & Valdés, 2008: 314, above bottom photo.

**Figure 63:**
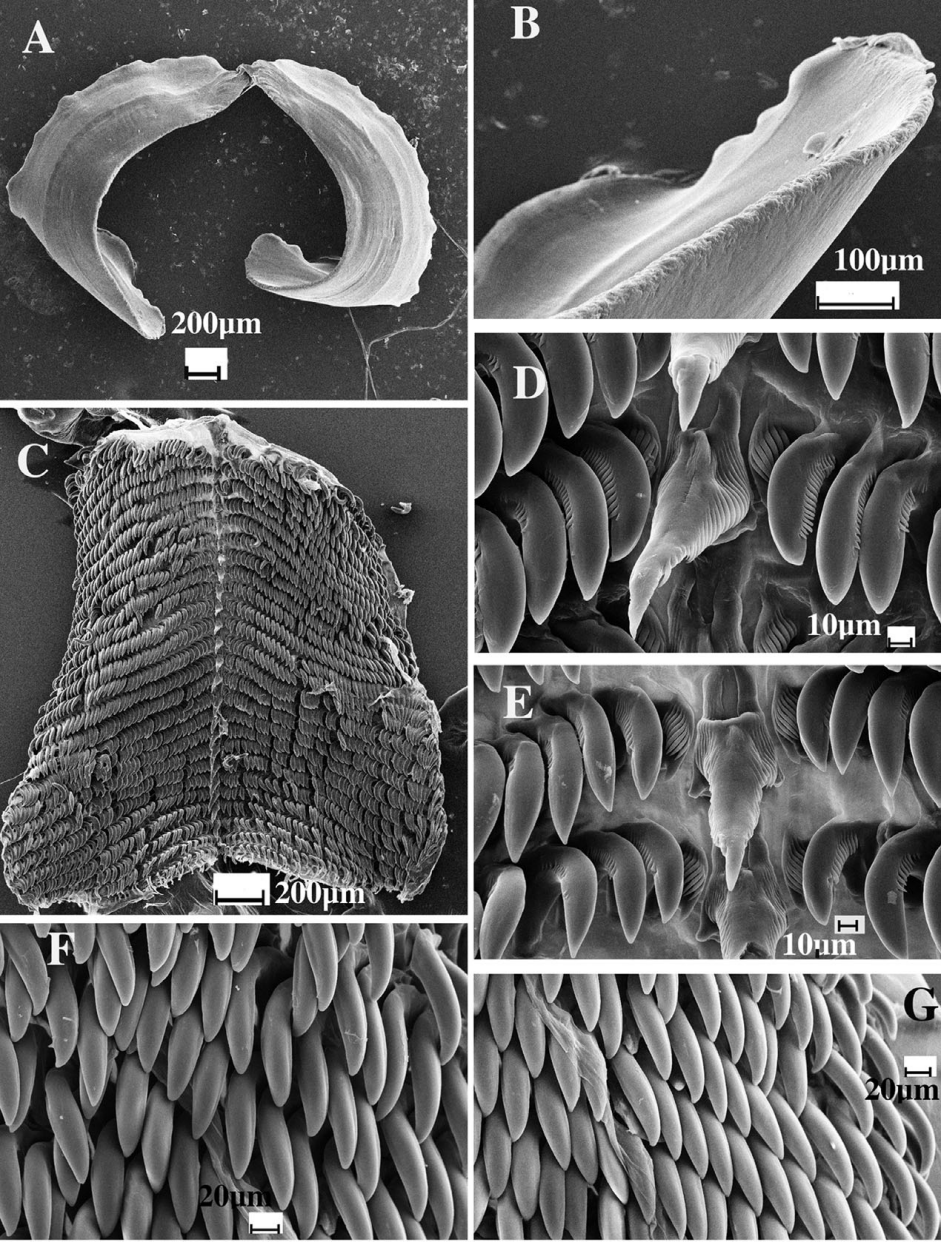
***Dermatobranchus leoni* sp. nov.** Buccal armature, CASIZ 167453, Mabini, Luzon, Philippines. A, jaws; B, masticatory margin; C, entire radula; D, E, central portion of radula; F, middle radular teeth; G, outer radular teeth.

**Figure 64:**
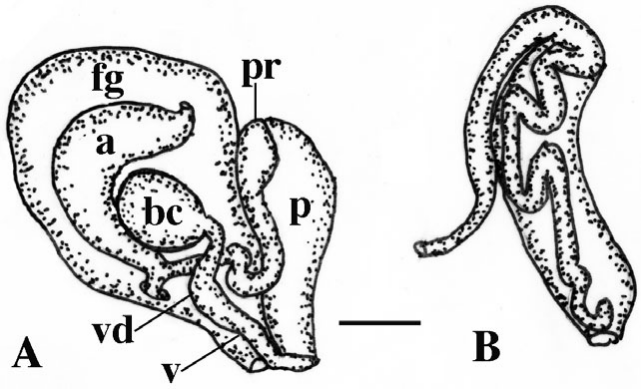
***Dermatobranchus leoni* sp. nov.** Reproductive system, CASIZ 167453, Mabini, Luzon, Philippines. A, reproductive system; B, detail of penial papilla. a, ampulla; bc, bursa copulatrix; fg, female gland mass; p, penis; pr, prostate; ps, penial sheath; v, vagina; vd, vaginal duct. Scale bar = 0.66 mm.

#### 

##### 

###### Type material

Holotype: CASIZ 167453, dissected, Ligpo Island, Balayan Bay, Luzon, Philippines, 42 m depth, collected iv.2003 by C. Petrinos.

###### Geographical distribution

Thus far, this species is known only from the Philippines (Gosliner *et al.*, 2008).

###### Etymology

This species was collected by our friend and colleague, Constantinos Petrinos. The specific name *leoni* honours his son, Leon.

###### External morphology

The body shape of the living animal (Fig. 58C) is elongate, but broad, slightly flattened, and narrows at the posterior end. The foot does not project beyond the distinct mantle margin. The dorsum has very low, longitudinal ridges and tubercles that give a lumpy appearance to the notum. The oral veil is large and expansive with slightly pointed corners. The well-separated, bulbous rhinophores are situated behind the oral veil. They have a series of longitudinal lamellae on the rounded club, which has a small rounded apex. The stalk does not narrow noticeably. There are noticeable marginal sacs along the mantle edge.

There are no hyponotal or branchial lamellae under the mantle margin. The genital opening is situated in the anterior quarter of the body. The anus is situated approximately half of the way to the posterior end of the body.

The ground colour of the dorsum is bright reddish orange with a series of black longitudinal lines along the margin of the ridges. The dorsal pigment is not evenly distributed. Each black line is flanked by longitudinal rows of opaque white spots. The foot is opaque white with a broad orange marginal band. A series of black, dashed lines are present on the dorsal surface of the foot and extend to the marginal band. The rhinophore stalk is white with darker bands of tan and the club is entirely tan. The rhinophores have a black rhinophoral club with opaque white lines along the lamellae. The rhinophoral apex is opaque white. The oral veil is opaque white with black lines and an orange anterior margin.

###### Buccal armature

The shape of the buccal mass is broad and highly muscular. The jaws are large and thickly cuticularized (Fig. 63A), with a thick masticatory margin. The masticatory margin is irregular, but no distinct denticles are evident (Fig. 63B). The radular formula (CASIZ 167453) is 29 × 40.1.1.1.40 (Fig. 63C). The rachidian teeth (Fig. 63D, E) are broad with a large, bluntly pointed central cusp that extends beyond the 23–25 denticles on either side. These denticles are present along most of the length of the tooth. Each rachidian tooth extends outward from a broad base that has two elongate extensions from the posterior margin. The first lateral tooth (Fig. 63D, E) is compact, hooked, and denticulate with a longer pointed central cusp with nine to ten smaller denticles. The next five to six lateral teeth are also large hooks with a large central cusp and 12–20 elongate, narrow denticles. The remaining middle and outer lateral teeth are hook-shaped, without denticles (Fig. 63F, G).

###### Reproductive system (Fig. 64)

The ampulla is thick and simply curved. It bifurcates into the large female gland mass via a short oviduct and into the vas deferens. The majority of the female gland mass is composed of the mucous gland, whereas the membrane and albumen glands are much smaller. The prostatic portion of the vas deferens is relatively wide and convoluted and widens further as it enters the bulbous penial sheath. Within the penial sheath, the narrow penis is highly convoluted and terminates in a slightly rounded apex. Adjacent to the penis is the wide curved vagina, which terminates in a relatively large, spherical bursa copulatrix.

###### Remarks

*Dermatobranchus leoni* is similar in appearance to *D. semistriatus* Baba, 1949, but the notum of *D. leoni* is covered with red pigment and the oral veil has large thick black lines rather than small spots. Additionally, the border of the foot of *D. leoni* has a wide orange margin, whereas that of *D. semistriatus* does not have a distinctly pigmented border. The masticatory border of the jaws of *D. leoni* (Fig. 63B) is smooth, whereas that of *D. semistriatus* has several rows of denticles (Baba, 1949: text fig. 87). The rachidian tooth of *D. leoni* has a longer cusp with denticles occurring almost all the way to the end of the cusp, whereas in *D. semistriatus* they are all basal. *Dermatobranchus leoni* has 40 rows of outer laterals whereas *D. semistriatus* has 50–60.

*Dermatobranchus nigropunctatus* Baba, 1949, *D. tongshanensis* Lin, 1981, and *D. multistriatus* Lin, 1981 also bear some external resemblance to *D. leoni*. The colour pattern of *D. nigropunctatus* is similar with black and white pigment, but this species has white lines with black spots whereas *D. leoni* has black lines with white spots. *Dermatobranchus leoni* has black rhinophores with white lines, whereas *D. nigropunctatus* has orange rhinophores. The colour of *D. tongshanensis* differs from that of *D. leoni* in several significant ways. The dorsal surface of *D. tongshanensis* is olive with scattered brown patches whereas *D. leoni* is predominantly reddish orange. The longitudinal lines of *D. tongshanensis* are white and black, whereas in *D. leoni* there are fine black lines and opaque white spots. The rhinophores of *D. tongshanensis* are blue with white lamellae whereas they are black with white lamellae in *D. leoni*. The sides of the body and dark oral veil of *D. tongshanensis* have small black spots whereas there are large black dashes in *D. leoni*. *Dermatobranchus multistriatus* lacks any black lines and has yellowish white ridges with small black spots on the oral veil and side of the body. It is difficult to compare the colour descriptions provided by Lin with the present species and the black and white plate of living animals sheds little additional light on the colour pattern of the animals. All four of these species have a jaw that lacks denticles. The most significant internal difference is in the shape of the radular teeth. In *D. nigropunctatus*, *D. tongshanensis*, and *D. multistriatus*, the inner lateral teeth are large with an elongate cusp. In contrast, *D. leoni* has a very short inner lateral tooth with a short cusp, more similar to that found in *D. ornatus*. Similarly, the other lateral teeth of *D. leoni* have a blunt cusp whereas the laterals of the other three species are acutely pointed. The reproductive system of the other three species was not described. On the basis of the colour differences noted and the very different shape of the radular teeth, we have decided it is more prudent to describe *D. leoni* as a new taxon until fresh material from China permits a more detailed comparison.

### *Dermatobranchus microphallus*sp. nov. (Figs 58D, 65, 66)

#### 

##### 

###### Type material

Holotype: CASIZ 086655, one specimen, dissected, 8 mm preserved, dissected, Unjuran Reef, Flores, Indonesia, 1 m depth, collected 29.iv.1992 by P. Fiene.

###### Geographical distribution

This species is known only from Flores, Indonesia (present study).

###### Etymology

The specific name *microphallus* refers to the small size of the fully mature penis of this species.

###### External morphology

The body shape of the living animal (Fig. 58D) is narrow and elongate, flattened, and narrows at the posterior end. The wide foot does not project beyond the distinct mantle margin. There is a series of low longitudinal dorsal ridges running the length of the body. The oral veil extends forward and is rounded at the corners. The rhinophores are situated behind the oral veil. They have a series of longitudinal lamellae on the rounded club. The stalk narrows noticeably. There are no lamellae on the stalk. Marginal sacs are readily visible along the mantle edge and are smaller and more numerous than in most other species observed. There are no branchial or hyponotal lamellae. The genital opening is situated approximately one-quarter of the way along the body side and the anus is situated about halfway along the body side.

**Figure 65:**
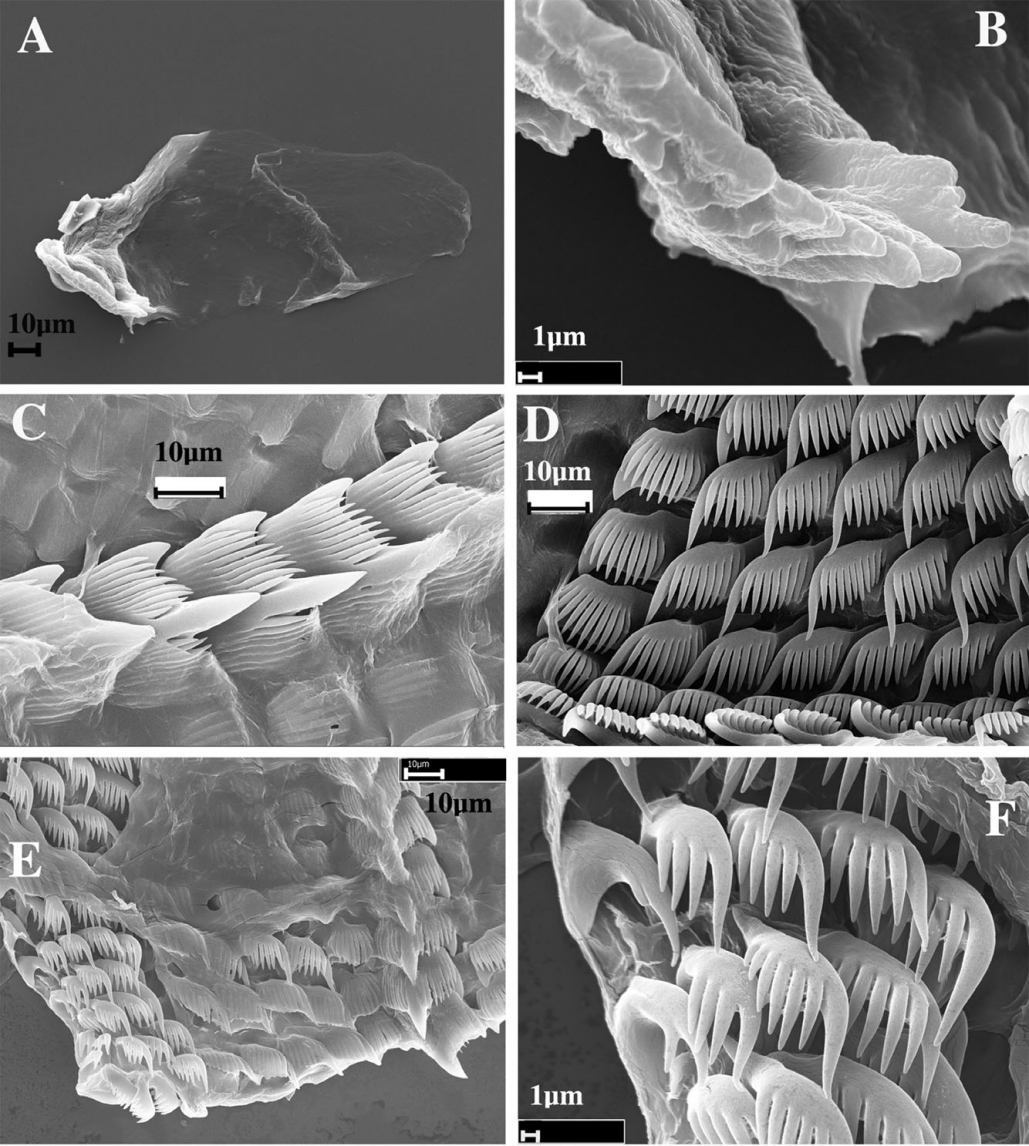
***Dermatobranchus microphallus* sp. nov.** Buccal armature, CASIZ 086655, Flores, Indonesia. A, jaw; B, masticatory margin; C, central portion of the radula; D, middle lateral teeth; E, half-row of radular teeth; F, outer lateral teeth.

The ground colour of the notum is black with about eight fine, opaque white longitudinal lines that are isolated on either side of the midline of the notum. Many of these also have larger opaque white spots along their length, especially on the lateral portions of the notum. The entire notal margin is opaque white. The rhinophore stalk is the same black colour as the notum as is the club, which has opaque white lines. The oral veil is opaque white throughout.

###### Buccal armature

The jaws are ovoid in shape and are thinly cuticularized (Fig. 65A), with a thin masticatory margin and one to two rows of rounded denticles, some of which have irregular apices (Fig. 65B). The radula is long and narrow with a formula of 31 × 9.1.1.1.9 in the holotype (CASIZ 086655). The rachidian teeth (Fig. 65C, E) are broad with large lateral cusps at the base of each tooth. There is a wide, pointed central cusp that is longer than the nine to ten long, flanking denticles on each side. The inner lateral teeth (Fig. 65D) are broad with a pointed first denticle that is longer than the following nine to ten long, narrow pointed denticles. The next eight lateral teeth (Fig. 65D–F) are elongate hooks with three to ten denticles, the number decreasing on the teeth furthest from the rachidian. The outermost teeth (Fig. 65F) have a single denticle adjacent to the elongate cusp.

###### Reproductive system

**Figure 66:**
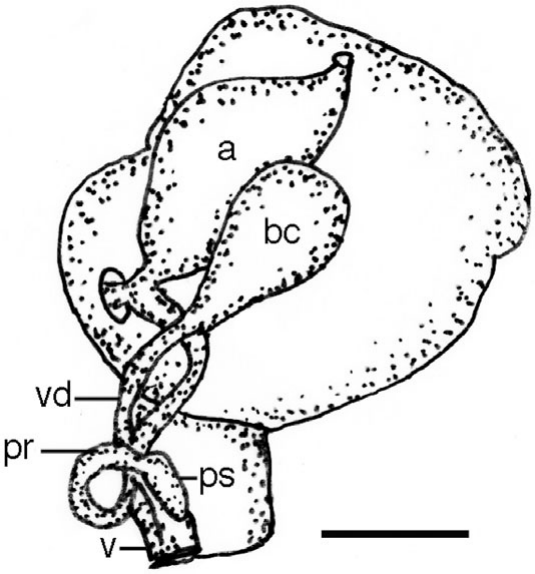
***Dermatobranchus microphallus* sp. nov.** Reproductive system CASIZ 086655, Flores, Indonesia. a, ampulla; bc, bursa copulatrix; pr, prostate; ps, penial sheath; v, vagina; vd, vaginal duct. Scale bar = 0.33 mm.

The reproductive organ arrangement is androdiaulic. The hermaphroditic duct is long and narrow and leads into the elongate, rounded, saccate ampulla (Fig. 66). The ampulla bifurcates near the centre of the female gland mass into the short oviduct and the long, tubular prostate, which coils once. The prostate expands into the small, conical, muscular penial papilla. The pyriform bursa copulatrix is slightly smaller than the ampulla and larger than the penial sheath. From the bursa, the long, narrow vaginal duct extends into the narrow vagina, which exits into the genital atrium, adjacent to the penis. The specimen was determined to be fully mature by the presence of large fully developed female reproductive glands.

###### Remarks

This species is immediately recognizable by its narrow elongate body with black pigment and fine white longitudinal lines. It is similar in colour to many species of *Armina* and to *D. albineus* and *D. arminus*, but has a much darker body colour. Internally, its radula is much narrower than any of these species but is most similar to *D. albineus*. This species also has broad rachidian tooth and a broad inner lateral. The most significant difference is the greater number of lateral radular teeth per row (12–18) in *D. albineus* (Fig. 31) than in *D. microphallus* (ten) (Fig. 65). Additionally, the eight to ten outer lateral teeth of *D. albineus* lack any denticles, whereas all of the teeth of *D. microphallus* have at least one denticle. The reproductive system of these two species differs markedly. In *D. albineus* (Fig. 32) the penial sheath is wide and elongate, whereas in *D. microphallus* (Fig. 66) it is small and conical. Additionally, *D. albineus* has a wide vaginal base whereas it is narrow in *D. microphallus*.

**Figure 75:**
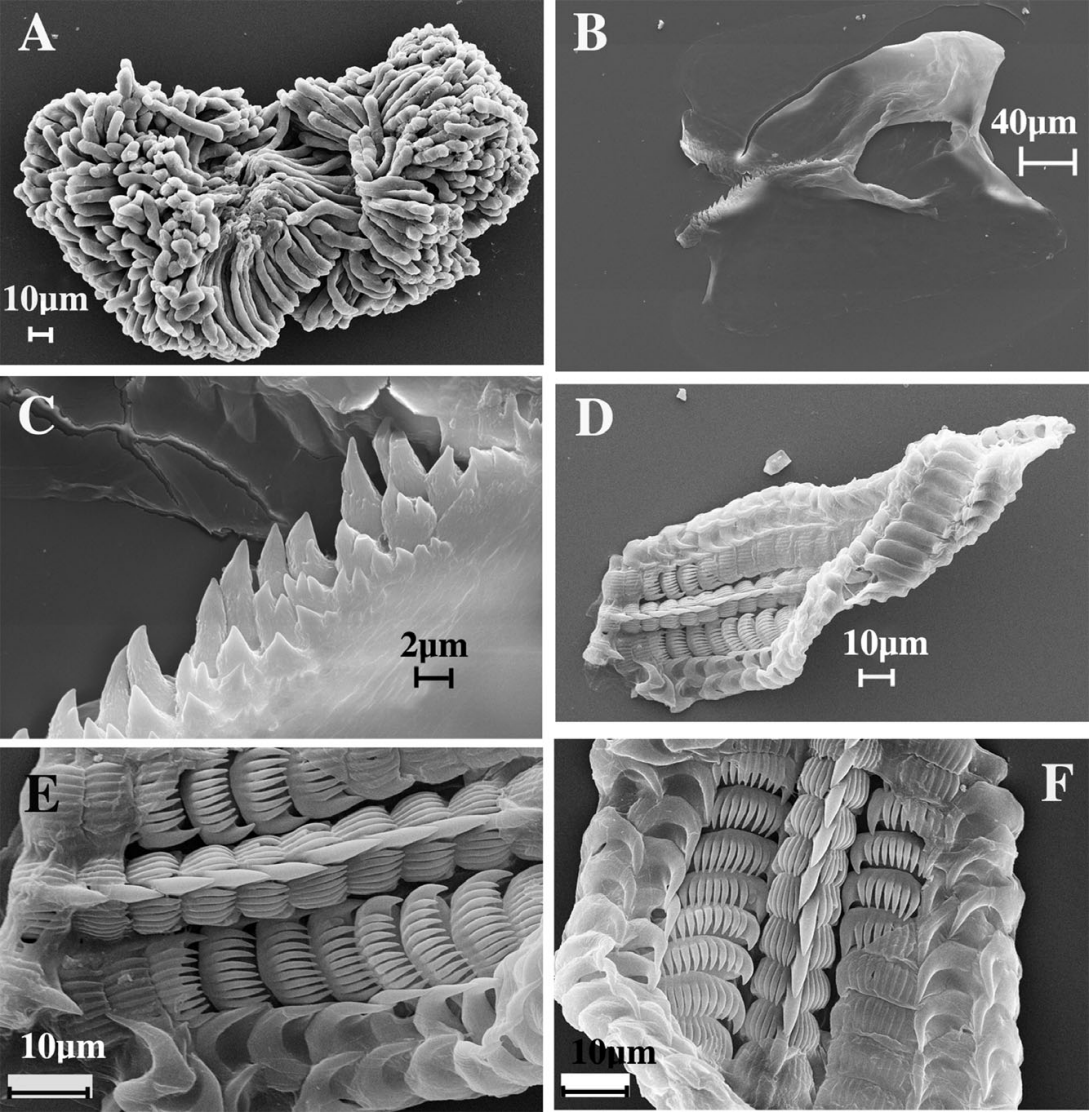
***Dermatobranchus rodmani* sp. nov.** Buccal armature, CASIZ 173400, Radama Islands, Madagascar. A, defensive rodlets; B, jaws; C, masticatory margin; D, entire radula; E, F, central portion of radula.

**Figure 76:**
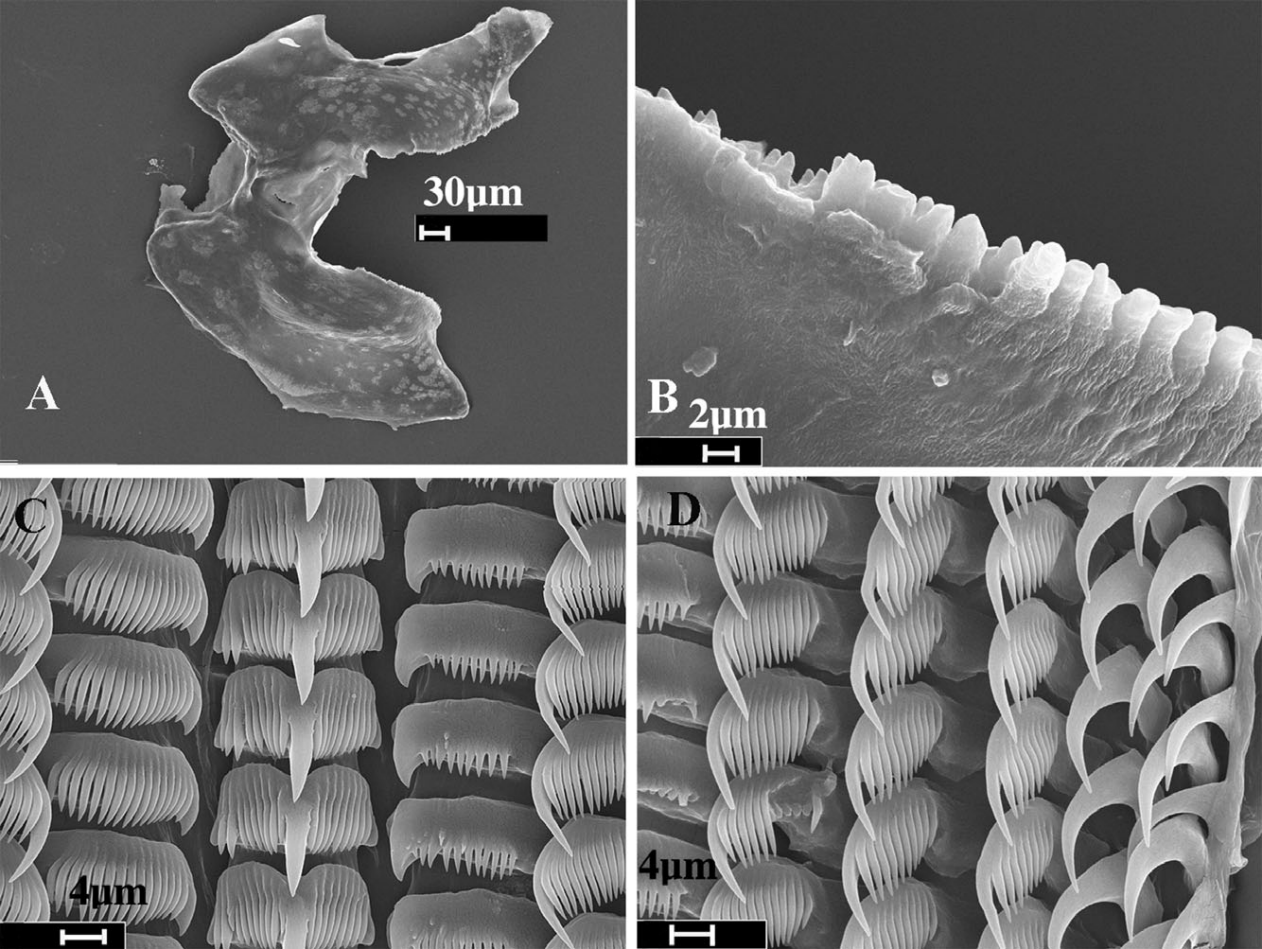
***Dermatobranchus rodmani* sp. nov.** Buccal armature, CASIZ 174170, Pulau Labus. Tioman, Malaysia. A, jaws; B, masticatory margin; C, central portion of radula; D, outer radular teeth.

*Dermatobranchus microphallus* is also similar in internal morphology to several other members that are characterized by having a long narrow radula with broad, denticulate inner lateral teeth: *D. substriatus D. striatellus*, *D. fortunatus*, *D. earlei*, *D. funiculus*, *D. rodmani, D. piperoides*, and *D. kokonas*. The black colour with white lines is unique to *D*. *microphallus.* Of these species only *D. rodmani* may have low ridges on the notum in larger individuals. The other species either entirely lack ridges (*D. fortunatus*, *D. piperoides*, *D. kokonas*) or have more prominent ridges (*D. substriatus D. striatellus*, *D. fortunatus*, *D. earlei*, *D. funiculus*). *Dermatobranchus rodmani* (Figs 75F, 76D) differs from *D. microphallus* by its light, mottled body colour, the presence of fewer dorsal ridges (maximum of three), and by its outer two lateral teeth devoid of denticles.

### *Dermatobranchus oculus*sp. nov. (Figs 58E, 67, 68)

*Dermatobranchus* sp. Coleman 2001: 103.

*Dermatobranchus* sp. Coleman 2008: 120, as Okinawa *Dermatobranchus*.

*Dermatobranchus* sp. 8 Gosliner, Behrens & Valdés, 2008:311, above bottom photo.

#### 

##### 

###### Type material

Holotype: CASIZ 174138, 8 mm, Okinawa, Ryukyu Islands, Japan, 13 m depth, collected 9.vii.1991 by R. Bolland (RFB 2622-K). Paratypes: CASIZ 079270, three specimens, 7–8 mm, one 8 mm dissected, Okinawa, Ryukyu Islands, Japan, 13 m depth, collected 9.vii.1991 by R. Bolland (RFB 2622-K).

###### Geographical distribution

This species is known only from Okinawa (present study).

###### Etymology

The specific name *oculus* is from the Latin word meaning ‘eye’ or ‘bud’ referring to the evenly spaced oscillated spots on the dorsum.

###### External morphology

The body shape of the living animal (Fig. 58E) is elongate and narrows at the posterior end. The foot does not project beyond the distinct mantle margin. There is a series of longitudinal dorsal ridges. The oral veil extends forward and has blunt extensions at the corners. The widely spaced rhinophores are behind the oral veil. They have a series of longitudinal lamellae on the rounded club. The stalk does not narrow noticeably. There are visible marginal sacs along the mantle edge. There are no branchial or hyponotal lamellae. The genital opening is situated at the anterior quarter of the body side and the anus is situated in the anterior half.

The ground colour of the dorsum and foot is opaque white. The depressions between the dorsal ridges are light grey. There are dark centred ocellated spots on the dorsum surrounded by pink pigment. The rhinophore stalk is white and the club is black. The oral veil is opaque white with a light grey border.

###### Buccal armature

**Figure 67:**
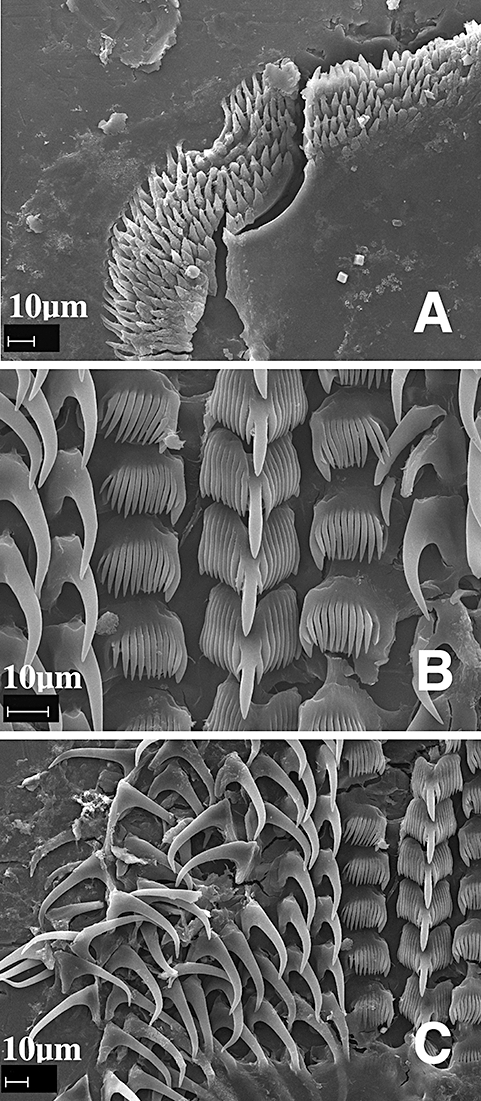
***Dermatobranchus oculus* sp. nov.** Buccal armature, CASIZ 079270, Okinawa, Japan. A, masticatory margin; B, central portion of the radula; C, half-row of radular teeth.

The jaws are large and thickly cuticularized, with a thick masticatory margin and seven rows of long, pointed denticles (Fig. 67A). The radular formula of a paratype (CASIZ 079270) is 23 × 10–8.1.1.1.8–10. Additional rows of teeth may have been lost during preparation of the radula. The rachidian teeth (Fig. 67B) have a broad base with a large, thick, and long central cusp that is wider than the up to seven flanking denticles on each side. The central cusp may have two attached, smaller denticles; one per side. The inner lateral teeth (Fig. 67B) have a wide base with a slightly longer first denticle with a small secondary denticle on the outside edge and ten shorter and narrower, pointed denticles adjacent. The next eight to ten lateral teeth are long hooks without denticles (Fig. 67C).

###### Reproductive system

The reproductive organ arrangement is androdiaulic. The hermaphroditic duct leads into the wide, tubular ampulla (Fig. 68). The ampulla bifurcates into the female gland mass via a short oviduct and into the short, narrow prostate. The prostate curves, then expands into the elongate, tubular penial sheath that is as long as the entire prostate. The round bursa copulatrix is smaller than the ampulla. From the bursa, the short, narrow vaginal duct extends into the equally narrow vagina, which exits into the genital aperture adjacent to the penial sheath.

###### Remarks

Externally, *D. oculus* most closely resembles *D. otome*, *D. semistriatus* Baba, 1949, and *D. primus* Baba, 1976. All of these species have a white ground colour, raised longitudinal ridges with darker coloration in the depressions between the ridges, and dark ocellated spots on the dorsum. Of these, *D. otome* has orange rhinophores, whereas the remaining species have black or dark brown pigment on the rhinophores. In *D. primus*, the dark pigment is on the base of the rhinophore stalk and the apex is orange, whereas the black pigment is on the club in both *D. semistriatus* and *D. oculus*. Additionally, the frontal margin of the oral veil of *D. semistriatus* and *D. primus* is orange whereas it is grey in *D. oculus*. *Dermatobranchus otome* lacks a marginal band, and the entire oral veil is white.

The radular morphology of these species has some similarities. The number of rows of teeth is similar, about 20 in a 10 mm specimen of *D. primus,* 25 in a 20 mm specimen of *D. semistriatus* and 23 or more in an 8 mm specimen of *D. oculus*. The rachidian tooth of all species has a long, thick central cusp with flanking denticles (up to seven in *D. oculus,* five to six in *D. primus*, and 11–14 in *D. semistriatus*). Both *D. oculus* and *D. semistriatus* have a differentiated first lateral tooth, whereas *D. primus* does not and all of its lateral teeth lack denticulation. The morphology of the remaining lateral teeth differs between that of *D. oculus* and *D. semistriatus*. The succeeding four to five lateral teeth of *D. semistriatus* are denticulate, followed by 50–60 nondenticulate teeth, but only the first lateral tooth of *D. oculus* is denticulate, and the remaining eight to ten teeth are hook-shaped and smooth. The lateral teeth of *D. primus* have a rounded knob on the top but the lateral teeth of *D. oculus* have either straight, or slightly depressed tops. The jaw of these species also differs in the number of rows of rods. Baba (1949) described one to two rows of spiny denticles for *D. semistriatus* and three to four rows of ‘scale-like’ denticles for *D. primus* (Baba, 1976). *Dermatobranchus oculus* has seven rows of long, pointed denticles.

The radular configuration of *D. oculus*, with only the inner lateral tooth denticulate, is similar to *D. otome*, *D. striatus*, *D. albus*, and *D. diagonalis*. Additionally, all of these species have jaws with multiple rows of denticles situated only at the base of the jaws. All of these species have colour patterns that are very different from that of *D. oculus*, and their reproductive systems differ markedly, as well. In *D. oculus*, the prostate is relatively narrow and expands into a much wider elongate penial sheath. In the remaining species the penial sheath is only slightly wider than the prostate and is relatively shorter and conical.

*Dermatobranchus oculus* also externally resembles some specimens of *D. funiculus* from Okinawa. Both species have white ground colour and raised longitudinal ridges with darker coloration in the depressions between the ridges. However, there are no ocellated spots on the dorsum of *D. funiculus* as there are in *D. oculus*.

The radular formula of these two species is also very different, with *D. funiculus* having a formula of 31–34 × 8.1.1.8 as opposed to 23 × 10–8.1.1.1.8–10 in *D. oculus*. Even more significantly, the majority of the lateral teeth of *D. funiculus* (Figs 54–56) are denticulate whereas only the inner laterals of *D. oculus* are denticulate (Fig. 67).

The reproductive morphology of these two species shares some similarities, such as a wide penial sheath and a narrow vaginal duct and vagina, but the prostate in *D. funiculus* (Fig. 57) is coiled and longer than that of *D. oculus* (Fig. 68).

### *Dermatobranchus phyllodes*sp. nov. (Figs 58G, H, 69–71)

*Dermatobranchus* sp., lobed *Dermatobranchus* Coleman, 2008: 121.

*Dermatobranchus* sp. 2 Gosliner, Behrens & Valdés, 2008: 308, above bottom photo.

*Dermatobranchus* sp. 6 Rudman, 2001.

#### 

##### 

###### Type material

Holotype: CASIZ 174164, 40 mm preserved, Christmas Bay, Bagabag Island, off Madang, Papua New Guinea, 7 m depth, collected 10.ii.1988 by T. M. Gosliner. Paratypes: CASIZ 070451, four specimens, one dissected, Christmas Bay, Bagabag Island, off Madang, Papua New Guinea, 7 m depth, collected 10.ii.1988 by T. M. Gosliner. CASIZ 174165, two specimens, 27, 29 mm preserved, one dissected, Pamilacan Island, Philippine Islands, 6–37 m depth, collected 11.vi.2004 by T. Gosliner, Y. Camacho, J. Templado, M. Malaquias, M. Poddubetskaia. CASIZ 084278, one specimen, 11 mm, dissected, Devil's Point, Batangas, Luzon Island, Philippine Islands, 4 m depth, collected 23.ii.1992 by M. Miller. CASIZ 070290, one specimen, 8 mm, dissected, Manado, Sulawesi, Indonesia, 12 m depth, collected 20.v.1989 by P. Fiene (M75). CASIZ 078587, one specimen, 14 mm, Bunaken Island, Manado, Sulawesi, Indonesia, 5 m depth, collected 21.v.1991 by P. Fiene (M75). CASIZ 173351, two specimens, 45 mm preserved, Pamilacan Island, Philippine Islands, 10–41 m depth, collected 7.vi.2004 by T. Gosliner, Y. Camacho, J. Templado, M. Malaquias, M. Poddubetskaia. CASIZ 174166, three specimens, 29, 30, 40 mm preserved, Pamilacan Island, Philippine Islands, 2–4 m depth, collected 11.vi.2004 by T. Gosliner, Y. Camacho, J. Templado, M. Malaquias, M. Poddubetskaia. CASIZ 083872, two specimens, 4–8 mm preserved, Devil's Point, south-west of Maricaban Island, Batangas Province, Luzon Island, Philippine Islands, no depth recorded, collected 26.iii.1992 by T. Gosliner.

###### Geographical distribution

This species is known only from Indonesia, the Philippine Islands, and Papua New Guinea (present study), and the Solomon Islands (Rudman, 2001).

###### Etymology

The specific name *phyllodes* is from the Greek word meaning ‘leaflike: chlorophyll’ in reference to the distinctive leaf-like appearance of this species' dorsum.

###### External morphology

The body shape of the living animal (Fig. 58G, H) is elongate, flattened, and narrows at the posterior end. The foot projects only slightly beyond the distinct mantle margin. There is a series of deep dorsal ridges nearly perpendicular to the mantle edge, giving the dorsum a distinct leaf-like appearance. The oral veil extends forward and has blunt corners and often is notched medially. The widely spaced rhinophores are situated behind the oral veil. They have a series of longitudinal lamellae on the rounded club. The stalk does not narrow noticeably. Marginal sacs are visible along the mantle edge. There are no branchial or hyponotal lamellae. The genital opening is on the right side of the body, near the anterior of the body. The anus is situated approximately one third of the way from the anterior end to the posterior end of the body.

The ground colour of the dorsum, the oral veil, and the foot is pale green and pinkish blotches are also prevalent. There are dark green speckles and spots of various sizes scattered randomly on the dorsum. There is a U-shaped band of dark green pigment at the anterior third of the notum. The rhinophore stalk is white and the club is dark. The oral veil is opaque white with dark spots along the anterior margin and a pale orange edge. The foot margin has small dark speckles.

###### Buccal armature

**Figure 70:**
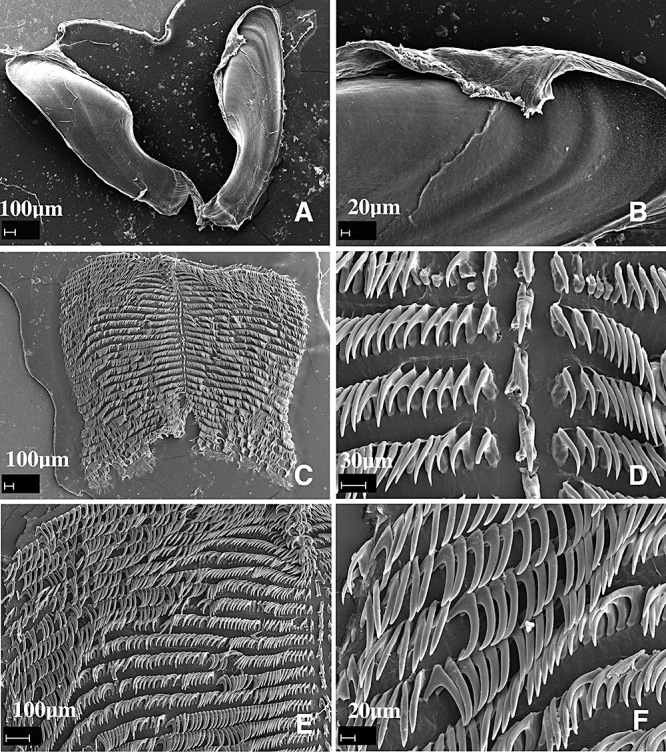
***Dermatobranchus phyllodes* sp. nov.** Buccal armature, CASIZ 174165, Pamilacan Island, Bohol, Philippines. A, jaws; B, masticatory margin; C, entire radula; D, central portion of radula; E, half-row of radular teeth; F, outer lateral teeth.

The jaws are large and thickly cuticularized (Figs 69A, 70A), with a thick masticatory margin and multiple rows of pointed denticles (Figs 69B, 70B). The radular formulae of four paratypes are: (CASIZ 070451) 32 × 99.1.99 (Fig. 69C), (CASIZ 174165) 32 × 85.1.85 (Fig. 70C), (CASIZ 082278) 16 × 39.1.39, and (CASIZ 070290) 21 × 50.1.50. The rachidian teeth (Figs 69D, 70D) have a narrow base with a large, projecting, hook-shaped, pointed central cusp that has no denticles on either side, although on some teeth there are three grooves on each side of the central cusp. The next 39–99 lateral teeth are long, pointed hooks with no denticles (Figs 69E, F, 70E, F).

###### Reproductive system

**Figure 71:**
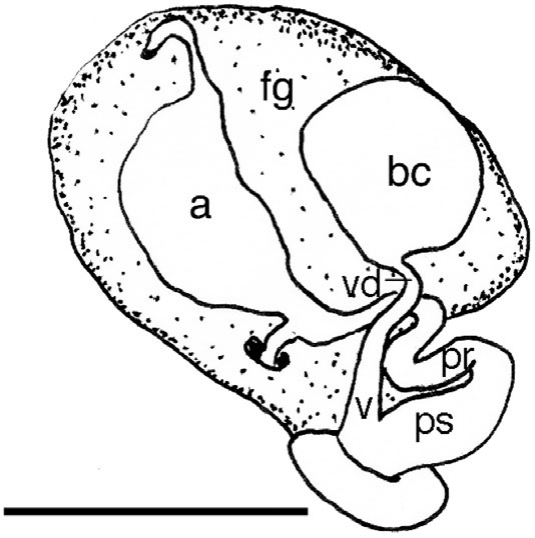
***Dermatobranchus phyllodes* sp. nov.** Reproductive system, CASIZ 107424, Lombok, Indonesia. a, ampulla; bc, bursa copulatrix; fg, female gland mass; pr, prostate; ps, penial sheath; v, vagina; vd, vaginal duct. Scale bar = 3.5 mm.

The reproductive organ arrangement is androdiaulic. The elongate hermaphroditic duct leads into the wide, thick, tubular ampulla (Fig. 71). The ampulla bifurcates into the female gland mass, via a short oviduct and into the short, curved prostate. The prostate expands into the wide, bulbous penial sheath. From the large, round bursa copulatrix the short, narrow vaginal duct leads to the wider vagina. The vagina exits into the genital atrium next to the penial sheath.

###### Remarks

Externally, this species does not closely resemble any other *Dermatobranchus*. No other species has the nearly perpendicular, deep dorsal ridges or the leaf-green background colour. *Dermatobranchus funiculus* from Okinawa and the Philippines has dorsal ridges that angle towards the mantle margin, but that species has a mid-dorsum longitudinal ridge, lacking in *D. phyllodes.* The background colour of these two species is very different. *Dermatobranchus phyllodes* is deep green with dark green and black spots, whereas *D. funiculus* has a white, blue or rust ground colour with blue-grey spots. Both species have a pale orange oral veil edge.

The radula of these two species is very different. *Dermatobranchus phyllodes* (Figs 69, 70) has a broad radula with a radular formula of 21–32 × 39–99.1.39–99, whereas that of *D. funiculus* (Figs 54–56) is 31–34 × 4.2.1.1.1.2.4.

The reproductive system of these two species is somewhat similar in that both have a bulbous penial sheath, although that of *D. funiculus* (Fig. 57) is much longer, as is the prostate.

Another species with ridges angling towards the notal margin is *D. diagonalis*, but in this species there is no wide medial area devoid of ridges. The radular morphology of *D. phyllodes* is unique amongst species of *Dermatobranchus*. It is the only species where all lateral teeth lack denticles. In contrast, *D. diagonalis* has a denticulate inner lateral tooth as in *D. otome*, *D. striatus*, *D. albus*, and *D. oculus.*

### *Dermatobranchus piperoides*sp. nov. (Figs 58F, 72, 73)

*Dermatobranchus* sp. 14 Gosliner, Behrens & Valdés, 2008: 313, top photo.

#### 

##### 

###### Type material

Holotype: CASIZ 174168, 1 km south of Casuarina, St. Leu, Reunion Island, 1 m depth, collected 21.iv.1989 by T. M. Gosliner. Paratypes: CASIZ 174169, eight specimens, two dissected, 1 km south of Casuarina, St. Leu, Reunion Island, 1 m depth, collected 21.iv.1989 by T. M. Gosliner.

###### Geographical distribution

This species is known only from the west coast of Reunion Island (present study).

###### Etymology

The specific name *piperoides* is a noun in apposition and comes from the Latin, *Piper*, the genus of black pepper, owing to the resemblance of the black markings on the notum of this species to ground pepper.

###### External morphology

The body shape of the living animal (Fig. 58F) is short and triangular, flattened, and narrows at the posterior end. The foot projects only slightly beyond the distinct mantle margin. The notum is granular and lacks any traces of distinct ridges. The oral veil extends forward and has pointed corners. The widely spaced rhinophores are behind the oral veil. They have a series of longitudinal lamellae on the rounded club. The stalk does not narrow noticeably. Marginal sacs are visible along the mantle edge. There are no branchial or hyponotal lamellae. The genital opening is on the right side of the body, near the anterior quarter of the body. The anus is situated approximately one**-**third of the way to the posterior end of the body.

The ground colour of the dorsum, the oral veil, and the foot is opaque white. There are a few black spots of various sizes scattered in the submarginal area of the notum and a single black spot is also present on the centre of the oral veil. The rhinophoral lamellae are orange with a black rhinophoral apex. The oral veil has a large orange blotch on its anterior margin. The foot is uniformly opaque white.

###### Buccal armature

**Figure 72:**
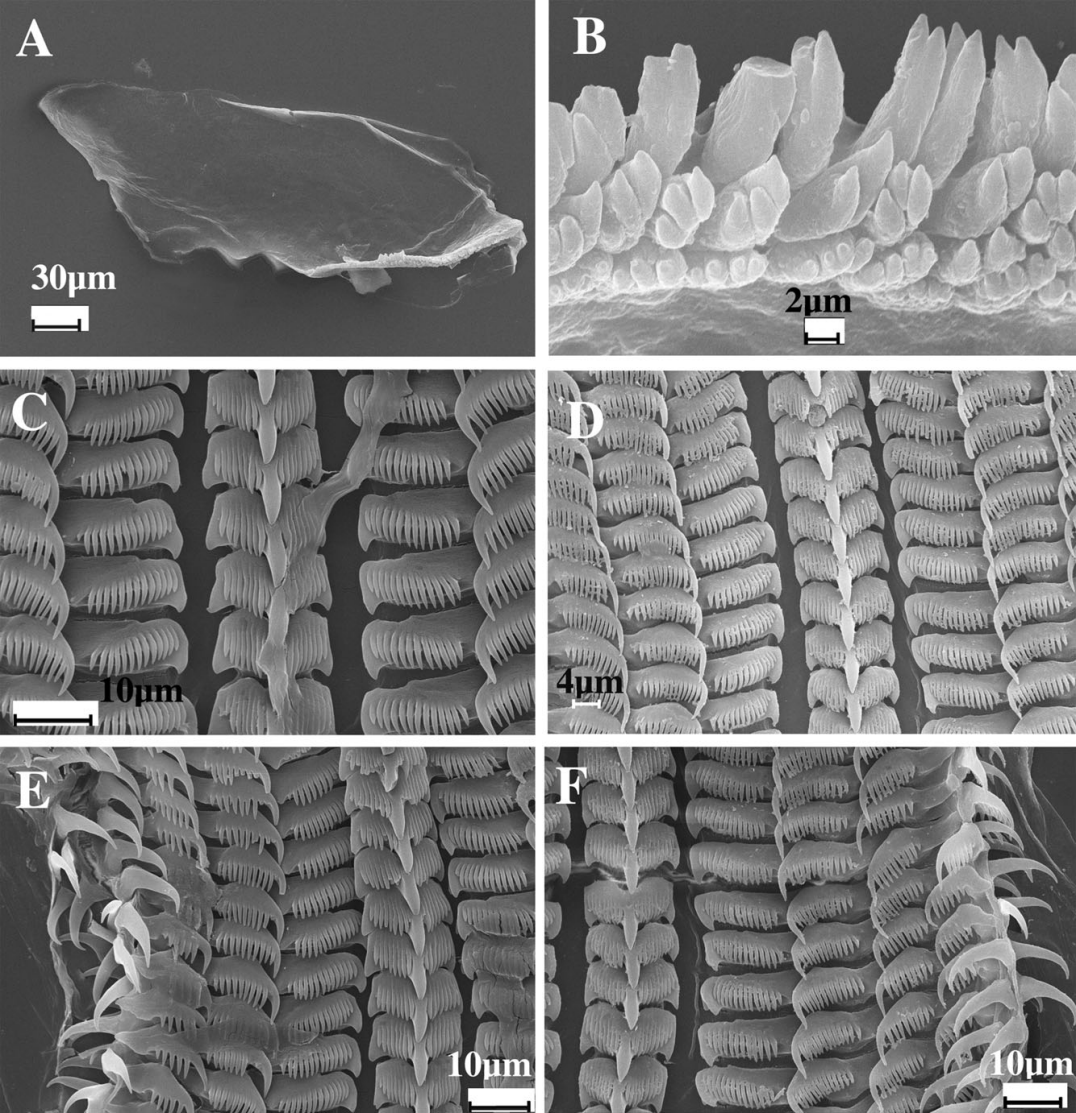
***Dermatobranchus piperoides* sp. nov.** Buccal armature, CASIZ 174169, St Leu, Reunion Island. A, jaw; B, masticatory margin; C, D, central portion of radula; E, F, half-row of teeth.

The jaws (Fig. 72A) are elongate with one to two rows of highly divided denticles (Fig. 72B) along the lower third of the jaw. The radular formulae of two paratypes (CASIZ 174169) are 46 × 6.1.1.1.6 and 44 × 5.1.1.1.5. The rachidian teeth (Fig. 72C, D) are broad with a broad base and an elongate, wide central cusp. The cusp is flanked by seven to nine elongate denticles on either side. The inner lateral tooth is extremely broad with 14–15 elongate denticles on the outer side of the central cusp. The next two to three laterals bear 8–12 denticles on the outer side of the sharp cusp. The outer two to three teeth lack denticles (Fig. 72D, E).

###### Reproductive system

**Figure 73:**
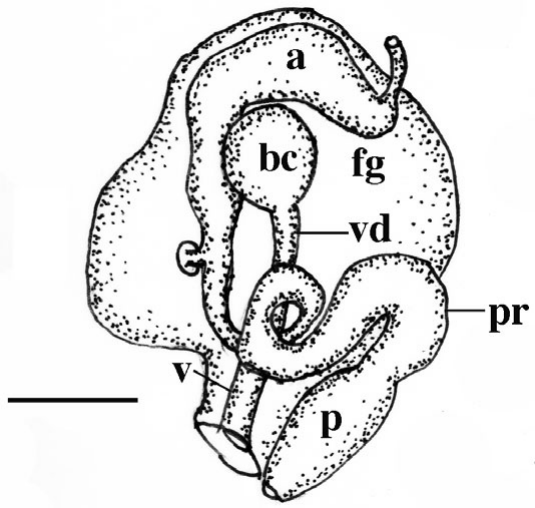
***Dermatobranchus piperoides* sp. nov.** Reproductive system, CASIZ 174169, St Leu, Reunion Island. a, ampulla; bc, bursa copulatrix; fg, female gland mass; pr, prostate; ps, penial sheath; v, vagina; vd, vaginal duct. Scale bar = 0.2 mm.

The reproductive organs are androdiaulic (Fig. 73). The ampulla is curved and saccate, branching to the female gland mass via a short oviduct and to a thick, convoluted prostatic vas deferens that terminates in a short, conical penial papilla. The papilla has an acutely pointed apex. The well-developed female gland mass is well differentiated into a large mucous gland and smaller albumen and membrane glands. Adjacent to the penis is a thin, straight vagina that leads to a relatively smallspherical or pyriform bursa copulatrix inside the folds of the female glands.

###### Remarks

*Dermatobranchus piperoides* is most similar to *D. kokonas* and the differences are described above under the Remarks for this species. This species is similar to other species with divided masticatory cusps and a long radula.

### *Dermatobranchus rodmani*sp. nov. (Figs 74A, B, 75–77)

*Dermatobranchus* sp. 16 Gosliner, Behrens & Valdés, 2008: 313, bottom photo.

#### 

##### 

###### Type material

Holotype: CASIZ 173400, dissected, west of Nosy Valiha, Radama Islands, Madagascar,collected 20.x.2005 by T. Gosliner. Paratypes: CASIZ 174170, one specimen, dissected, Pulau Labus, Tioman, eastern Malaysia, 10 m depth, collected 2.x.2007 by D. W. Behrens. CASIZ 178238, piece of 174170 taken for DNA examination, Pulau Labus, Tioman, eastern Malaysia, 10 m depth, collected 2.x.2007 by D. W. Behrens. CASIZ 069745, one specimen, dissected, reef north of Pig Island, Madang, Papua New Guinea, 24 m depth, collected 24.viii.1989 by T. Gosliner. CASIZ 173425, one specimen, west of Nosy Kalakajoro, Radama Islands, Madagascar (13°58.37′S, 47°41.76′E), 15–17 m depth, collected 17.x.2005 by S. Fahey.

###### Geographical distribution

This species is known from the north-west coast of Madagascar to eastern Malaysia and Papua New Guinea (present study).

###### Etymology

The specific name *rodmani* is in honour of James Rodman, long-time Program Officer of the Division of Environmental Biology of the National Science Foundation, who had the vision and determination to realise the creation of several programmes that have fostered the resurgence of systematic biology in the United States. The Partnerships for Enhancing Expertise in Taxonomy programme that was his brain-child has been critical in providing support for training the next generation of opisthobranch mollusc systematists.

###### External morphology

The body shape of the living animal (Fig. 75A, B) is elongate and triangular, flattened, and narrows at the posterior end. The foot projects only slightly beyond the distinct mantle margin. The notum is smooth and fleshy. There are three low, distinct longitudinal ridges in the larger specimen from Malaysia and no ridges are evident in the material from Madagascar. The oral veil extends forward and has rounded corners. The widely spaced, club-shaped rhinophores are situated behind the oral veil. They have a series of longitudinal lamellae on the rounded club. The stalk does not narrow noticeably. Marginal sacs are visible along the mantle edge and each contains elongate, stiff rodlets (Fig. 75A). There are no branchial or hyponotal lamellae. The genital opening is on the right side of the body, near the anterior quarter of the body. The anus is situated approximately one**-**third of the way to the posterior end of the body.

The ground colour of the notum, the oral veil, and the foot is pale pinkish white to tan. One or two transverse brown patches are present near the anterior and posterior thirds of the body. The notum and oral veil have a yellowish margin, which may contain fine punctations of brown. The rhinophores, including the base, lamellae, and apex, are brown with a few opaque white spots on their surface.

###### Buccal armature

The jaws (Figs 75B, 76A) are elongate with one to two rows of denticles with highly divided apices (Figs 75C, 76B) along the lower third of the jaw. The radula is elongate and narrow (Fig. 75D). The radular formula of the holotype (CASIZ 173400) is 24 × 2.1.1.1.2 and that of one paratype (CASIZ 174170) is 40 × 5–6.1.1.1.5–6. The rachidian teeth (Figs 75E, F, 76C) are broad with a broad base and an elongate, wide central cusp. The cusp is flanked by six to nine elongate denticles on either side. The inner lateral tooth is extremely broad with 7–15 elongate denticles on the outer side of the central cusp. The next zero to three laterals bear 10–13 denticles on the outer side of the sharp cusp. The outer two to three teeth lack denticles (Figs 75E, F, 76D).

###### Reproductive system

**Figure 77:**
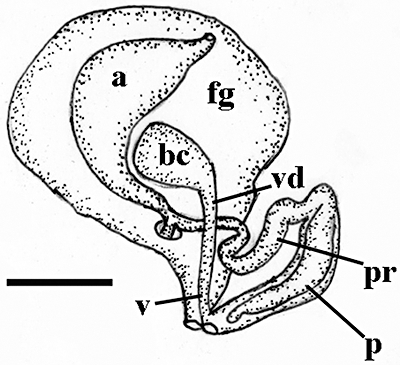
***Dermatobranchus rodmani* sp. nov.** Reproductive system, CASIZ 173400, Radama Islands, Madagascar. a, ampulla; bc, bursa copulatrix; fg, female gland mass; p, penis; pr, prostate; v, vagina; vd, vaginal duct. Scale bar = 0.37 mm.

The reproductive organs are androdiaulic (Fig. 77). The ampulla is curved and saccate, with a short-branched oviduct connecting to the female gland mass and a second branch becoming the thin, convoluted vas deferens that terminates in an elongate, curved conical penial papilla. The papilla has an acutely pointed apex. The well-developed female gland mass is well differentiated into a large mucous gland and smaller albumen and membrane glands. Adjacent to the penis is a thin, straight vagina that leads to a relatively small spherical or pyriform bursa copulatrix inside the folds of the female glands.

###### Remarks

*Dermatobranchus rodmani* is externally similar to other species with brownish bands or horizontal blotches including *D. fasciatus*, *D. semilunus*, and *D. tuberculatus*. Of these species, it differs from *D. fasciatus* and *D. semilunus* in lacking numerous prominent longitudinal ridges. *Dermatobranchus tuberculatus* has elevated tubercles on the notum that are not present in *D. rodmani*. The radula of *D. rodmani* differs from all of these species in that it is thin and elongate with relatively few teeth and with a broad inner lateral tooth with numerous denticles.

The radula is similar in shape and dentition to *D. substriatus D. striatellus*, *D. fortunatus*, *D. earlei*, *D. funiculus*, *D. microphallus, D. piperoides*, and *D. kokonas*. Of these species, *D. substriatus, D. striatellus, D. earlei*, and *D. funiculus* all have prominent dorsal ridges that are not evident in the other species. Both *D. rodmani* and *D. microphallus* have relatively low ridges on the notum whereas the remaining species entirely lack ridges. The body of *D. microphallus* is black with faint white longitudinal lines along the low ridges. In contrast, *D. rodmani* is pale pink to tan with brown patches on the notum. The radular formula of *D. microphallus* is 31 × 9.1.1.1.9 whereas it is 24 × 2.1.1.1.2 and 40 × 5–6.1.1.1.5–6 in *D. rodmani*. The inner seven teeth of *D. microphallus* (Fig. 65) are denticulate whereas in *D. rodmani* (Figs 75–76) only the inner one to four teeth are denticulate. In *D. microphallus* (Fig. 66) the small penial sheath is at the distal end of a long prostatic section whereas in *D. rodmani* (Fig. 77), the short prostate leads to a much longer penial papilla and sheath.

### *Dermatobranchus semilunus*sp. nov. (Figs 74C–E, 78–81)

*Dermatobranchus* sp. 7 Gosliner, Behrens & Valdés, 2008: 311, top three photos.

#### 

##### 

###### Type material

Holotype: CASIZ 073045, dissected, 5 mm preserved, north end Kranket Island, Madang, Papua New Guinea, 11 m depth, collected 1.x.1986 by T. Gosliner. Paratypes: CASIZ 086679, one specimen, dissected, 12 mm alive, Wair Mitak, Flores, Indonesia, 5 m depth, collected 25.iv.1992 by P. Fiene. CASIZ 110407, one specimen, dissected, north-west end of Cabilao Island, Bohol Strait, Bohol, Philippines, 30 m depth, collected 28.iv.1997 by T. M. Gosliner. CASIZ 174141, one specimen, dissected, Pulau Chimbe, off Tioman, Malaysia, collected 5.x.2007 by T. Gosliner. CASIZ 174142, four specimens, one dissected, Pulau Varella, Malaysia, 15 m depth, collected 1.x.2007 by T. Gosliner. CASIZ 178236, one specimen, sampled for DNA, Pulau Varella, Malaysia, 15 m depth, collected 1.x.2007 by T. Gosliner. CASIZ, 178239, piece of specimen CASIZ 174141, sampled for DNA, Pulau Chimbe, off Tioman, Malaysia, collected 5.x.2007 by T. Gosliner.

###### Geographical distribution

This species is known only from Papua New Guinea, Indonesia, eastern Malaysia, and the Philippines (present study).

###### Etymology

The specific name *semilunus* is from the combined Latin words meaning half-moon, in reference to the dark, semi-lunar shaped marking on the anterior third of the dorsum of this species.

###### External morphology

The body shape of the living animal (Fig. 74C–E) is elongate, flattened, and narrows at the posterior end. The foot does not project beyond the distinct mantle margin. There is a series of 10–14 low longitudinal dorsal ridges. The oral veil extends forward and has blunt extensions at the corners. The widely spaced rhinophores are behind the oral veil. They have a series of longitudinal lamellae on the rounded club. The stalk does not narrow noticeably. Marginal sacs are visible along the mantle edge. There are no branchial or hyponotal lamellae. The genital opening is in the anterior quarter of the body wall on the right side. The anus is situated approximately half of the way to the posterior end of the body.

The ground colour of the dorsum, the oral veil, and the foot is opaque white. The depressions between the dorsal ridges are light grey with dark dots. There is a single dark U-shaped band of pigment at the anterior third of the notum. Along the mantle edge are evenly spaced, dark ocellated spots of pigment. The posterior edge of the mantle has a yellowish or tan hue. The rhinophore stalk is white and the club is dark. The oral veil is opaque white with some dark irregular spots.

###### Buccal armature

The jaws are large and thickly cuticularized (Figs 78A, 79A, 80A), with a thick masticatory margin and multiple rows of long, pointed denticles (Figs 78B, 79B, 80B). The radula is wide (Figs 79C, 80C). The radular formula of the holotype (CASIZ 073045) is 24 × 33.1.1.1.33 (Fig. 78). Four paratypes have a radular formula of 23 × 33.1.1.1.33 (CASIZ 086679), 23 × 29.1.1.129 (CASIZ 110407), 29 × 40.1.1.1.40 (CASIZ 174142), and 35 × 73.1.1.1.73 (CASIZ 174141), respectively. The rachidian teeth (Figs 78C, 79D, 80D) have a narrow base with a large, projecting, pointed central cusp that has one to eight pointed, flanking denticles on each side. There is a narrow posterior notch on the top of each rachidian tooth. The inner lateral tooth (Figs 78C, 79D, 80D) has a flat base with a large hook-shaped first denticle with one to six much shorter and narrower, pointed denticles. The next five lateral teeth are long pointed hooks with up to 14–15 pointed denticles on the outer side or they may be entirely devoid of denticles. The remaining 17–70 lateral teeth (Figs 78D, E, 79E, F, 80E, F) are long hooks lacking denticles, with the outermost teeth being less arched and more elongate than the others.

###### Reproductive system

The arrangement of the reproductive organs is androdiaulic. The hermaphroditic duct leads into the elongate, tubular ampulla (Fig. 81). The ampulla bifurcates neat the centre of the female gland mass and into a short oviduct and the very short prostate. The prostate expands into the wide, s-shaped penial sheath. From the small, round bursa copulatrix the narrow vaginal duct extends and then narrows further before expanding into the long, wider vagina. The vagina exits into the genital aperture next to the penial sheath.

###### Remarks

Externally *D. semilunus* looks most similar to *D. rodmani, D. tuberculatus*, and *D. fasciatus.* All four species have a basically white ground colour, a noticeable dark brown band or bands of pigment crossing the dorsum and darker coloured rhinophore clubs. Of these, *D. rodmani* and *D. tuberculatus* lack numerous prominent longitudinal ridges found in *D. fasciatus* and *D. semilunus*. For a more complete comparison of *D. fasciatus* and *D. semilunus*, see the Remarks section for *D. fasciatus*.

As noted in the Remarks for *D. cymatilus*, the radular morphology of *D. semilunus* is most similar to that species, but with consistent differences.

### *Dermatobranchus tuberculatus*sp. nov. (Figs 74F–H, 82–85)

*Dermatobranchus* sp. 17 Gosliner, Behrens & Valdés, 2008: 314, top photo.

*Dermatobranchus* sp. Coleman, 2008: 122, as pink-edged *Dermatobranchus*.

#### 

##### 

###### Type material

Holotype: CASIZ 112297, one specimen, dissected, 10 mm preserved, Ligpo Island,Batangas Province, Luzon Island, Philippine Islands, 28 m depth, collected 18.iv.1996 by T. Gosliner. Paratypes: CASIZ 103780, one specimen, 5 mm preserved, Ligpo Island, Batangas Province, Luzon Island, Philippine Islands, 36 m depth, collected 3.iv.1995 by T. Gosliner. CASIZ 110363, one specimen, 5 mm preserved, Ligpo Island, Batangas Province, Luzon Island, Philippine Islands, no depth recorded, collected 23.iv.1997 by C. Carlson. CASIZ 096332, one specimen, 11 mm preserved, Devil's Point, south-west of Maricaban Island, Batangas Province, Luzon Island, Philippine Islands, 25 m depth, collected 17.iii.1994 by T. Gosliner. CASIZ 110361, two specimens, 7–9 mm preserved, Ligpo Island, Batangas Province, Luzon Island, Philippine Islands, 25 m depth, collected 23.iv.1997 by T. Gosliner. CASIZ 071239, three specimens, 2–12 mm preserved, the Quarry, Cape Croiselles, north of Madang, Papua New Guinea, 30 m depth, collected 11.ii.1988 by T. Gosliner. CASIZ 105640, one specimen, 5 mm preserved, Medio Island, Verde Passage, Mindoro Island, Philippine Islands, no depth recorded, collected 28.ii.1995 by T. Gosliner. CASIZ 174171, one specimen, dissected, Tokong Kamundi, east Malaysia, 15 m depth, collected 29.ix.2007 by T. Gosliner. CASIZ 178236, one specimen, piece of CASIZ 174171 sampled for DNA extraction, Tokong Kamundi, east Malaysia, 15 m depth, collected 29.ix.2007 by T. Gosliner. CASIZ 068699, one specimen, dissected, Hole in the Wall, near Hussein Village, Madang Province, Papua New Guinea, 7 m depth, collected 21.vii.1989 by J. Markham.

###### Geographical distribution

Known from Papua New Guinea, east Malaysia, and the Philippines (present study).

###### Etymology

The specific name *tuberculatus* refers to the presence of rounded tubercles on the dorsal surface of the notum that characterize this species. It is a noun in apposition

###### External morphology

The body shape of the living animal (Fig. 74F–H) is elongate and triangular, flattened, and narrows at the rounded posterior end. The foot projects only slightly beyond the distinct mantle margin or may be entirely covered by the notum. The notum is rough and tuberculate with randomly distributed rounded tubercles on the surface. There are no distinct longitudinal ridges in any of the specimens examined. The oral veil extends forward and has rounded corners. The widely spaced, club-shaped rhinophores are behind the oral veil. They have a series of longitudinal lamellae on the rounded club and a short apex above it. The stalk does not narrow noticeably. Marginal sacs are visible along the mantle edge. There are no branchial or hyponotal lamellae. The genital opening is on the right side of the body, near the anterior of the body. The anus is situated approximately one**-**third of the way from the posterior end of the body.

The ground colour of the notum, the oral veil, and the foot is pale white to tan or purple. The tubercles are generally light brown with darker brown speckling. A light brown triangular mark may be present mid-dorsally on the anterior half of the notum. The oral veil is white with a few dark brown specks and has a pink to purple margin. The rhinophores are light brown with darker brown pigment just below the white apex.

###### Buccal armature

The jaws (Figs 82–84A) are elongate with five to seven rows of undivided denticles (Figs 82–84B) along most of the length of the jaw. The radula is moderately short and wide (Figs 70–72C). The radular formula of the holotype (CASIZ 112297) is 18 × 8.1.1.1.8 and those of four paratypes are 24 × 16.1.1.1.16 (CASIZ 174171), 19 × 12.1.1.1.12 (CASIZ 112297), 25 × 14.1.1.14 (CASIZ 068699), and 22 × 16.1.1.1.16 (CASIZ 096332). The rachidian teeth (Figs 82D, 83E, 84D) are narrow with a narrow, elongate central cusp. The cusp is flanked by five to ten elongate, sharp denticles on either side. The inner lateral tooth (Figs 82D, 83E, 84D) is broad with seven to ten elongate denticles on the outer side of the central cusp. The next two to nine laterals bear 5–11 denticles on the outer side of the sharp cusp (Figs 82E, 83D, 84E–F). The outer three to five teeth lack denticles.

###### Reproductive system (Fig. 85)

The ampulla is thick and simply curved. It bifurcates into the large female gland mass via a short oviduct and the vas deferens. The majority of the female gland mass is composed of the mucous gland whereas the membrane and albumen glands are much smaller. The vas deferens is initially relatively narrow and widens into a convoluted prostatic duct that enters the bulbous penial sac. Within the penial sac, the penis is wide and slightly curved and terminates in an acutely pointedapex. Adjacent to the penis is the elongate, wide, curved vagina, which narrows sharply distally and terminates in a relatively large, spherical bursa copulatrix.

###### Remarks

This species is externally and internally most similar to *D. pustulosus*. Both species are characterized by an irregular body surface, but in *D. pustulosus* these form distinct nodules rather than rounded tubercles as in *D. tuberculatus*. The rhinophores are far more bulbous in *D. pustulosus* and the oral veil has dark brown spots on its outer margin whereas it is uniformly pink to purple in *D. tuberculatus*.

Internally, there are several consistent differences between the two species. The radula of *D. pustulosus* (Figs 22, 23) contains 32–44 lateral teeth on each outer side of the inner lateral teeth, whereas in *D. tuberculatus* (Figs 82–84) there are 8–16 teeth on either outer edge of the inner lateral teeth. In *D. tuberculatus*, there are three to five short denticles on either side of the central cusp of the rachidian teeth, whereas in *D. pustulosus* there are five to ten elongate denticles on either side. In *D. pustulosus*, the inner lateral tooth is narrow with three to five denticles on the outer side of the short central cusp, whereas in *D. tuberculatus* the inner laterals are wide with seven to ten denticles on the outer side of the elongate central cusp. In *D. pustulosus* the outer 12 lateral teeth lack denticles whereas in *D. tuberculatus* the outer three to five teeth lack denticles.

The reproductive system of *D. pustulosus* has a short penial sac whereas it is elongate in *D. tuberculatus*. In *D. pustulosus* (Fig. 24) the vagina is relatively narrow throughout its length whereas in *D. tuberculatus* (Fig. 85), it is very wide in the basal portion and narrows abruptly before entering the bursa copulatrix.

## PHYLOGENETIC RELATIONSHIPS

### Characters examined

The following characters were considered for use in the analyses of Arminidae. The character states are indicated as follows: 0, generally the presumed plesiomorphic condition; 1,2, apomorphic condition. In some cases, where clearly stated, 1 or 2 is the presumed plesiomorphic state. For character states that are not applicable, ‘-’ was used and for missing data, ‘?’ was used. The distribution of plesiomorphic and apomorphic character states is presented in Table 2. Character states for the taxa examined for the present study can be found in Table 2. ‘Outgroup taxa’ refers to the combination of species identified in the Material and Methods section.

1. *Gill:* Present (0), absent (1). A gill is present in the outgroup taxon (*Berthella canariensis*) and is therefore considered plesiomorphic. The branchial and hyponotal lamellae of the Arminidae and dorsal gills of the Tritoniidae located along the margins of the notum are symmetrical along the body as opposed to the single gill of *B. canariensis* and the various Doridina taxa. Therefore, they are not considered homologous to that of basal opisthobranchs and are considered secondarily derived. In the taxa with these secondarily derived respiratory structures, a gill is considered absent.
2. *Gill position:* Lateral (0), dorsal (1). In the outgroup, the gill is situated along the right side of the body as in most basal opisthobranchs and is considered as plesiomorphic. The dorsal gill of dorid nudibranchs is considered homologous to the gill of *Berthella* and is considered derived.
3. *Body shape:* Oval (0), wedge (1), Elongate (2). The body shape of *B. canariensis* is oval, as is that of dorid nudibranchs. This shape is considered plesiomorphic. Members of the Arminidae are either wedge-shaped (Fig. 1B) or elongate (Fig. 1C), as are members of other ingroups.
4. *Rhinophoral ridge:* Present (0), absent (1). In Arminidae, there is a distinct notal ridge behind the rhinophores in some taxa (Fig. 1B). This ridge is also present in all outgroup and ingroup taxa exclusive of the Arminidae. It is therefore considered plesiomorphic. In other Arminidae, including all species of *Dermatobranchus*, the ridge is absent (Fig. 1D) and is considered the apomorphic condition.
5. *Notal ridges:* Absent (0), present (1). In some Arminidae, prominent notal longitudinal ridges are present on the body (Fig. 1D), whereas in other taxa no ridges are evident (Fig. 1I). The absence of notal ridges is considered plesiomorphic, as they are not found in *Berthella*, or other ingroup taxa other than some species of *Armina* and *Dermatobranchus*.
6. *Notal tubercles:* Absent (0), Present (1). The dorsal surface of the outgroup taxon is relatively smooth. This arrangement is considered plesiomorphic. Other taxa (Fig. 1H) have warty tubercles over the body surface, which is considered apomorphic.
7. *Notal cerata:* Absent (0), present (1). *Berthella canariensis* lacks any notal cerata (finger-like extensions of the body that contain extensions of the digestive gland). Members of several Dendronotina and armininids of the Proctonotidae, Madrellidae, and Heroidae all have notal cerata with the digestive gland, which is considered apomorphic.
8. *Oral structures:* None (0), tentacles (1), veil (2). Traditionally (Odhner, 1939), the presence of an oral veil has been considered the characteristic feature that unites members of the Arminina into a natural group. An oral veil is present in the outgroup taxon, *B. canariensis* and is considered plesiomorphic (2). The presence of this structure in Arminina cannot be a synapomorphy for this group as it is a plesiomorphic feature. Other taxa such as Doridina lack these oral structures. Other taxa, such as Proctonotidae, have tentacles on the anterior portion of the body, which is also considered apomorphic.
9. *Oral veil:* Smooth, no projections (0), projections (1). In most taxa with an oral veil, the veil is smooth as in *B. canariensis*. In other taxa, such as *Armina papillata* there is a series of elongate papillae found on the oral surface of the veil (Jensen, 1997: fig. 1). This later condition is considered apomorphic.
10. *Cephalic structure:* Absent (0), present (1). There are no additional cephalic structures present in the outgroup, *B. canariensis*. A few other taxa including *Galeojanolus ionnae* and *Armina comta* have additional cephalic structures present such as a hood or larger papillae. This latter condition is considered apomorphic. Subsequent analysis demonstrated that these structures are not homologous.
11. *Caruncle:* Absent (0), present (1). A caruncle is a sensory ridge located near the rhinophores. It is absent in the outgroup taxon but is present as a longitudinal structure located between the rhinophores of most Proctonotidae and as a transverse structure anterior to the rhinophores in most species of *Armina*. Its presence is considered apomorphic. Subsequent evaluation of the homology of this structure in the Proctonotidae and Armina is found in the discussion.
12. *Rhinophoral lamellae:* Horizontal (0), vertical (1), none (2). In the outgroup taxon, there are no rhinophoral lamellae (2), which is the presumed plesiomorphic state. In most other taxa there are either vertical or horizontal rhinophoral lamellae, which are considered apomorphic states.
13. *Anus position:* Above pallial margin (0), below pallial margin (1). In the outgroup taxon and the majority of other taxa studies, the anus is located ventral to the pallial margin (1) and is considered plesiomorphic. In dorid nudibranchs and Proctonotidae, the anus is located centrally near the posterior end of the body. This is considered apomorphic (0).
14. *Foot tentacles:* Absent (0), present (1). The outgroup and vast majority of taxa lack tentacles on the anterior end of the foot. This condition is considered plesiomorphic. In *D. rubidus* and *D. pulcherrimus*, tentacles are present.
15. *Anterior foot border:* Not notched (0), notched (1). In the outgroup, the anterior border of the foot is notched (1). This is the presumed plesiomorphic condition. Other taxa have a smooth anterior border of the foot, which is considered apomorphic (0).
16. *Branchial lamellae:* Absent (0), present (1). The outgroup taxon lacks secondary respiratory folds and has only the lamellae on the true gill. This is considered plesiomorphic. Members of *Armina* have branchial lamellae whereas all other taxa lack them. Their presence in *Armina* is considered apomorphic.
17. *Hyponotal lamellae:* Absent (0), present (1). The outgroup taxon lacks secondary hyponotal lamellae and has only the lamellae on the true gill. This is considered plesiomorphic. Members of *Armina* and *D. arminus* have hyponotal lamellae whereas all other taxa lack them. Their presence in these taxa is considered apomorphic.
18. *Radula shape:* Broader than long (0), slightly longer than broad (1), much longer than broad (2). In the outgroup, the radula is much broader than long and is considered plesiomorphic. Many species of Arminidae have a radula that is broader than long (Fig. 22C) (0). Others have a radula that is only slightly longer than broad (1) (Fig. 28C) whereas other taxa have a radula that is much longer than broad (2) (Fig. 12C). These latter two states are considered apomorphic.
19. *Rachidian tooth:* Absent (0), present (1). The outgroup lacks a central rachidian row of teeth in the radula, which is considered plesiomorphic. Presence of a rachidian tooth is considered apomorphic (Fig. 2D).
20. *Rachidian tooth denticles:* None (0), denticulate (1). The rachidian tooth may entirely lack denticles adjacent to the central cusp (Fig. 43E) (0), whereas the majority of taxa have well-developed denticles (Fig. 2D) (1). As the outgroup entirely lacks rachidian teeth, there is no presumed plesiomorphic state within the ingroup taxa.
21. *Rachidian tooth denticle number:* Few ≤ 5 (0), many > 5 (1). The rachidian tooth may have fewer than five denticles (Fig. 2D) (0) on each side of the central cusp or may have more than five denticles (Fig. 8D) (1). As the outgroup entirely lacks rachidian teeth, there is no presumed plesiomorphic state within ingroup taxa.
22. *Rachidian central cusp:* Not projecting (0), slightly projecting (1), greatly projecting (2). The central cusp of the rachidian tooth may not project further than the adjacent denticles. In Arminidae it may project slightly beyond the adjacent denticles (Fig. 2D) or may be greatly projecting (Fig. 35D). As the outgroup entirely lacks rachidian teeth, there is no presumed plesiomorphic state within ingroup taxa.
23. *Rachidian tooth shape:* Narrow (0), broad (1). The rachidian tooth may be relatively narrow (Fig. 35D) (0) or very broad (Fig. 2D) (1). As the outgroup entirely lacks rachidian teeth, there is no presumed plesiomorphic state within ingroup taxa.
24. *Top of rachidian tooth:* Flat or slightly indented (0), with elongate bases and deep notches (1). The top side of the rachidian tooth may be flat or slightly indented (Fig. 4D) (0), or it may deeply incised (Fig. 18C) (1). As the outgroup entirely lacks rachidian teeth, there is no presumed plesiomorphic state within ingroup taxa.
25. *Inner lateral tooth:* Smooth (0), denticulate (1). In the outgroup, the inner lateral tooth is smooth and is considered plesiomorphic. Some ingroup taxa also have smooth inner lateral teeth (Fig. 43E) (0) whereas others have denticulate inner lateral teeth (Fig. 2D) (1).
26. *Inner lateral tooth width:* Narrow (0), medium (1), broad (2). The outgroup taxon has narrow inner lateral teeth, as do several ingroup taxa (Fig. 43E) (0). This is considered the plesiomorphic condition. Other taxa have a moderately wide inner lateral tooth (Fig. 8D) (1), whereas other taxa have a very broad inner lateral tooth (Fig. 31F) (2). Both of these states are considered apomorphic.
27. *Inner lateral tooth shape:* Hammate, directed downward (0), elongate, laterally directed (1). In the outgroup taxon and several ingroup taxa, the cusp of the inner lateral tooth is directed downward (Fig. 69C) (0). This is the presumed plesiomorphic condition. In other taxa the denticles are directed laterally (Fig. 9E), which is considered the apomorphic state (1).
28. *Second lateral tooth:* Denticulate (0), smooth (1). In the outgroup taxon and several ingroup taxa (Fig. 9F), the second lateral tooth is smooth (1). This is the presumed plesiomorphic state. In other taxa the second lateral is denticulate (Fig. 12D), which is considered apomorphic (0).
29. *Middle lateral teeth:* Denticulate (0), smooth (1). In the outgroup taxon and several ingroup taxa (Fig. 9F), the teeth in the middle of the radular row are smooth (1). This is the presumed plesiomorphic state. In other taxa the middle lateral teeth are denticulate (Fig. 12D), which is considered apomorphic (0).
30. *Outer lateral teeth:* Denticulate (0), smooth (1). In the outgroup taxon and several ingroup taxa (Fig. 9F), the outer lateral teeth are smooth (1). This is the presumed plesiomorphic state. In other taxa the second lateral is denticulate (Fig. 25F), which is considered apomorphic (0).
31. *Jaw:* Present (0), absent (1). In the outgroup, and most ingroup taxa, the jaws are thickly cuticularized and divided into two discrete portions connected by a hinge (Fig. 2A) (0). This is the presumed plesiomorphic state. In other taxa the jaws are absent and only a thin membranous labial cuticle is present (1).
32. *Masticatory border:* Distinct (0), indistinct (1). In the outgroup taxon the masticatory border is indistinct (1) whereas in the ingroup taxa where jaws are present the masticatory border is distinct (0) (Figs 2A, B). An indistinct border is considered plesiomorphic.
33. *Masticatory margin:* Denticulate (0), smooth (1). The masticatory border of the jaw may be denticulate (Fig. 2B) (0) or smooth (Fig. 16A) (1). As the outgroup lacks a distinct masticatory margin, there is no presumed plesiomorphic state.
34. *Jaw denticles:* Seven to ten rows (0), three to six rows (1), one to two rows (2). The masticatory margin of the jaws may have a variable number of rows of denticles. There may be numerous rows (seven to ten) (Fig. 8B) (0), a moderate number of rows (three to six) (Fig. 22B) (1), or few rows (one to two) (Fig. 12B) (2). As the outgroup lacks a distinct masticatory margin, there is no presumed plesiomorphic state.
35. *Denticle location:* Along entire margin (0), basally (1), near hinge (2). Denticles may be present along the entire margin of the jaw (Fig. 4A, B) (0), may be located only basally (opposite the hinge) (Fig. 8A) (1), or may be located near the hinge (Fig. 45A) (2). As the outgroup taxon lacks a distinct masticatory margin, there is no presumed plesiomorphic state.
36. *Denticle shape:* Acutely pointed (0), rounded (1), multifid (2) The denticles along the masticatory margin may be either acutely pointed apices (Fig. 8B) (0), rounded apices (Fig. 40B) (1), or multifid apices (Fig. 31B) (2). As the outgroup lacks a distinct masticatory margin, there is no presumed plesiomorphic state.
37. *Reproductive system:* Androdiaulic (0), diaulic (1), triaulic (2). The outgroup taxon, Tritoniidae, and some dorids and Arminina have a diaulic arrangement or organs (1), which is considered plesiomorphic. In Proctonotidae there is a triaulic arrangement (2) whereas *Bathydoris* and Arminidae (0) have an androdiaulic arrangement of reproductive organs.
38. *Ampulla:* Tubular (0), mass (1). In the ingroup taxon and the vast majority of taxa studied the ampulla is tubular in shape, which is considered plesiomorphic (0). A few taxa, including the Tritoniidae, have a more massive ampulla (1).
39. *Penial sheath:* Bulbous (0), narrow (1). The outgroup taxon and many ingroup taxa have a penial sheath that is relatively narrow (Fig. 14) (1). This is the presumed plesiomorphic state. In other taxa the penial sheath is more bulbous in shape (Fig. 19) (0).
40. *Deferent duct:* Long (0), short (1). In the outgroup and many other ingroup taxa the deferent duct is elongate (Fig. 3) (0), which is considered plesiomorphic. In other taxa it is relatively short (Fig. 17) (1).
41. *Bursa duct:* Distinct duct present (0), bursa duct absent (1). In the outgroup and most ingroup taxa, the bursa copulatrix connects directly with the vagina (0). This arrangement is considered plesiomorphic. In some Proctonotidae the bursa is situated basally, adjacent to the vagina (1) without a distinct duct.
42. *Vaginal duct:* Long (0), short (1). In the outgroup taxon and most ingroup taxa, the vaginal duct is long (Fig. 3) (0), which is presumed to be plesiomorphic. In a few ingroup taxa, the vagina is short (Fig. 64) (1).
43. *Vaginal width:* Uniform (0), basally wider (1). In *B. canariensis* and most ingroup taxa the vagina is of a uniform diameter (Fig. 3) (0). This is considered to be the plesiomorphic state. In some ingroup taxa, the vagina is wider at the base, near the genital aperture (Fig. 32) (1).

### Phylogenetic analysis

**Figure 86:**
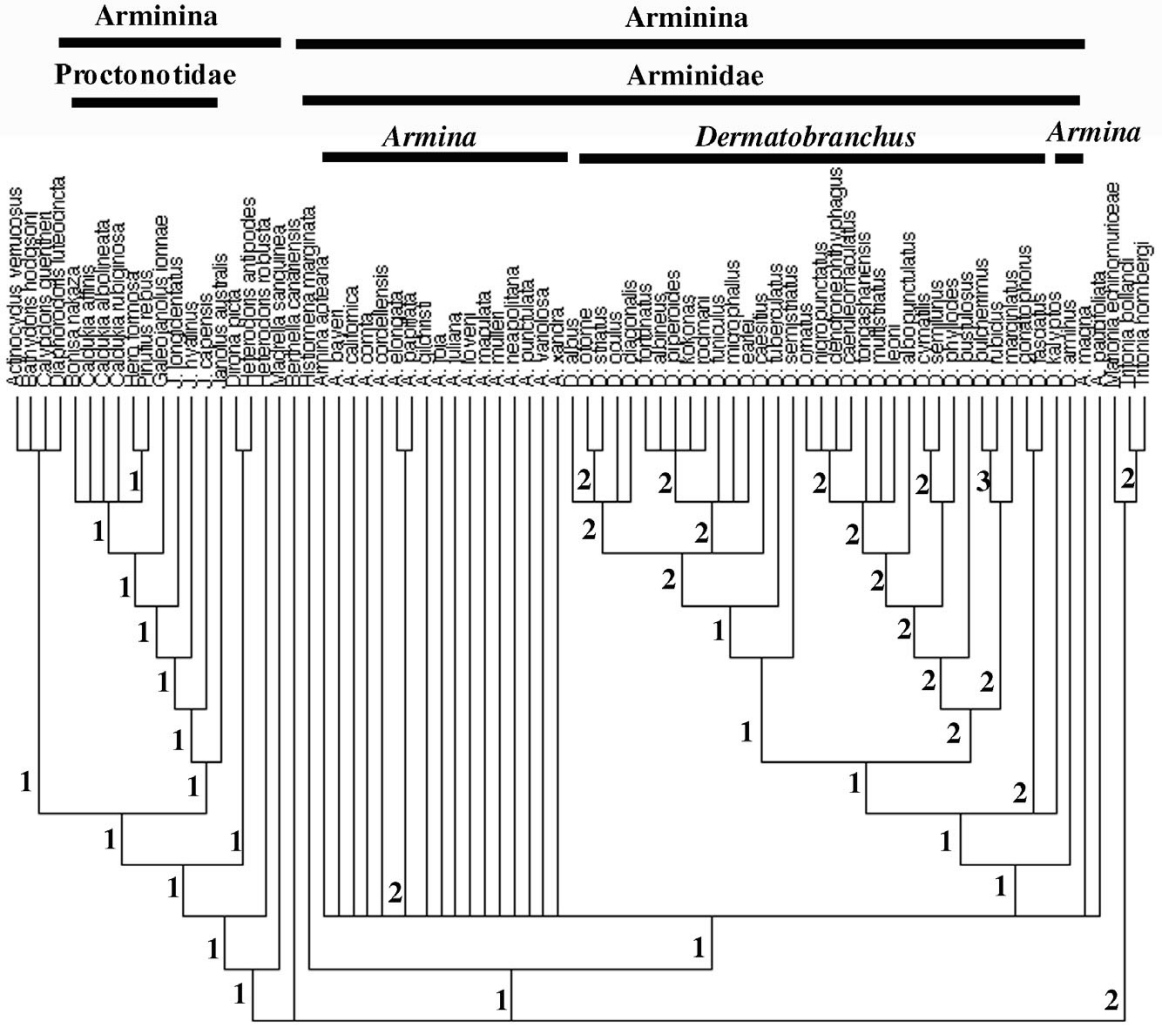
Phylogenetic relationships of Arminina relative to other nudibranch clades. Strict consensus tree showing Bremer support at each node and monophyly of Doridina, Arminidae, *Dermatobranchus*, and Tritoniidae.

**Figure 87:**
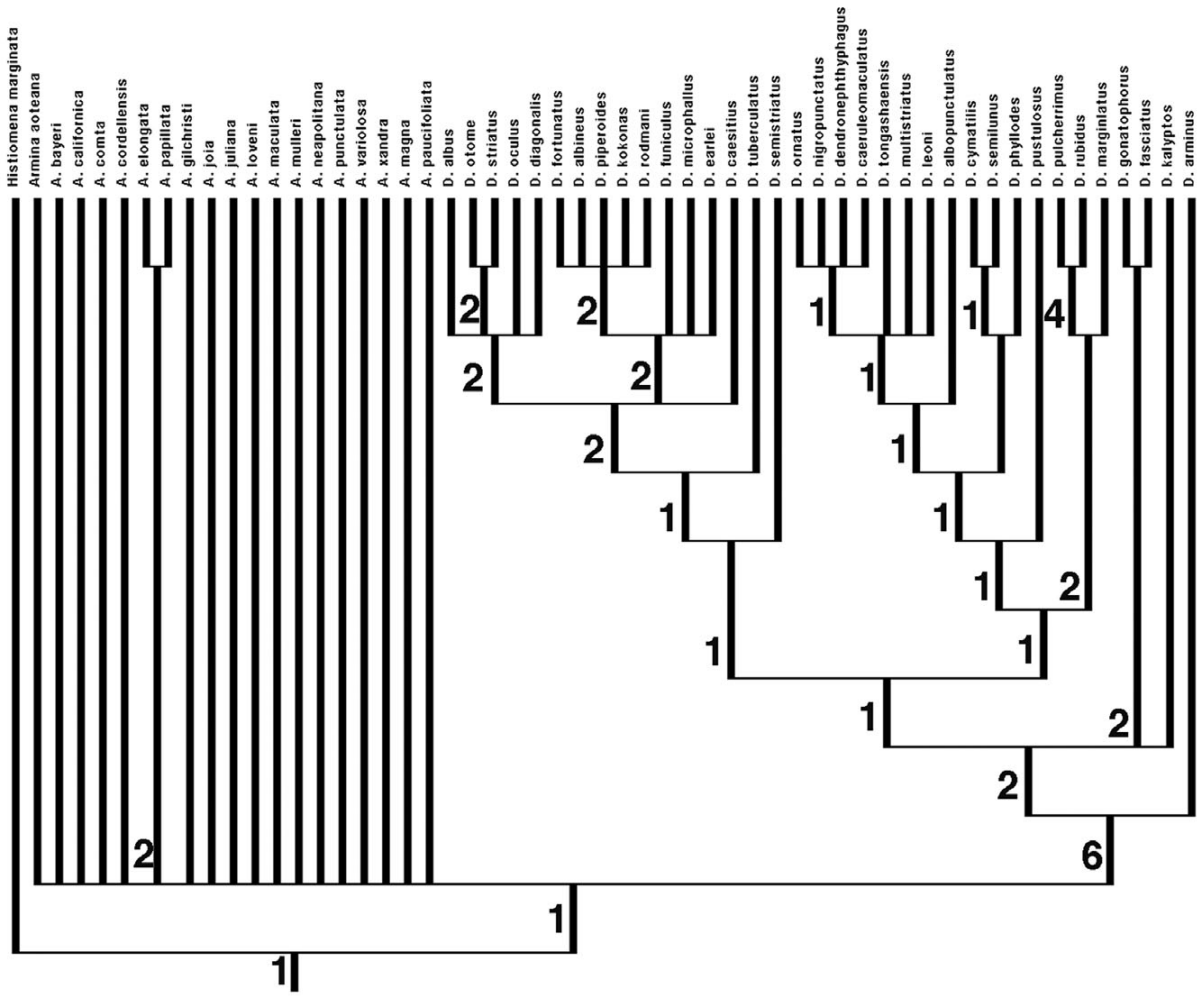
Phylogenetic relationships within Arminidae. Strict consensus tree showing Bremer support at each node.

**Figure 88:**
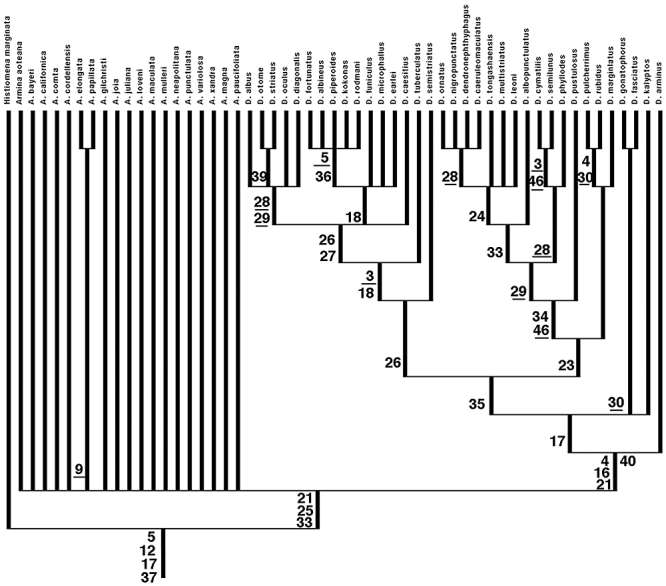
Phylogenetic relationships within Arminidae. Strict consensus tree showing distribution of synapomorphies used in analysis. Numbers correspond to character number in character list and data matrix and homoplasies are indicated by underlining of character number.

The analysis included 78 taxa. In order to test the relationships of members of Arminina to other nudibranchs, all taxa, except *B. canariensis*, were included in the ingroup. Six described species of *Dermatobranchus* were excluded from the analysis as they are poorly described and no specimens were available for the present study. These taxa are: *Dermatobranchus glaber* (Eliot, 1908), *D. striatellus* Baba, 1949, *D. multidentatus* Baba, 1949, *D. sagamianus* Baba, 1949, *D. primus* Baba, 1976, and *D. substriatus* Baba, 1949. The resulting phylogeny produced more than 173 200 most parsimonious trees of a length of 236 steps. Owing to the large data set and number of trees produced, only a single replicate was undertaken at a time, but the analysis was completed 20 times using different random seed start trees. All of the replicates produced trees with an identical topology. The strict consensus of these trees is shown in Figure 86. These trees had a consistency index of 0.229 and a retention index of 0.715. A Bremer decay analysis showed that most nodes are only weakly supported, with stronger support for clades within *Dermatobranchus*. Bremer support values are included in Figure 86. Based on the first analysis, a more constrained analysis was undertaken using *Histiomena* as the outgroup taxon, based on its more basal position within the Arminidae. All the remaining arminid taxa were also included in this analysis. The analysis was conducted with 100 replicates. This analysis produced 54 439 trees of a length of 133 steps. These trees had a consistency index of 0.301 and a retention index of 0.730. The strict consensus of these trees is included in Figure 87, with the Bremer support values indicated on the tree. Once again, there is weak to moderate support for most nodes, with the exception of the clade of all *Dermatobranchus* species, which has a support value of 6. It is interesting to note that the tree topology for species of *Dermatobranchus* is identical in both analyses. Synapomorphies that support clades in this analysis are shown in Figure 88. A majority rule tree was not shown because it would convey a false sense of robustness, especially given the relatively low Bremer support values for strict consensus trees.

The resulting trees support the monophyly of several traditional nudibranch taxa, namely Doridina Odhner, 1934, Proctonotidae Gray, 1853, Arminidae Iredale & O'Donoghue, 1923, and Tritoniidae Cuvier, 1797. Arminina is not monophyletic, with Proctonotidae and Arminidae forming separate clades (Fig. 86). Within the Arminidae, *Histiomena* is the most basal taxon and *Armina* lacks resolution, except that *A. elongata* Ardila & Valdés, 2004 and *A. papillata* Baba, 1933 appear to be sister taxa. Of the species included in *Dermatobranchus*, *D. arminus* is the most basal taxon. This species lacks branchial lamellae but possesses hyponotal lamellae. At the next highest node is a trichotomy that includes a large clade that contains most *Dermatobranchus* species, a clade that contains sister taxa *D. gonatophorus* and *D. fasciatus*, and a lineage that includes only *D. kalyptos*. Of these taxa, the clade that contains the majority of species of *Dermatobranchus* is composed of two subclades of 16 and 15 species, respectively. The subclade containing 16 species (referred to here as the *D. semistriatus* clade) has *D. semistriatus* as sister to the rest of the clade. At the next node above, *D. tuberculatus* is sister to the remaining 14 species. One more node above this one contains a trichotomy of *D. caesitius*, a clade of eight species (the *D. earlei* clade), and another of five species. The *D. earlei* clade contains a subclade of five species (the *D. fortunatus* clade) The *D. earlei* clade includes taxa with narrow elongate radula with a broad, laterally directed, inner lateral tooth. The other clade of five species (the *D. albus* clade) contains taxa that all have a denticulate inner lateral tooth with all the other laterals smooth. Within this clade, *D. otome* and *D. striatus* are sister taxa. This is of particular note, asBaba (1992) had repeatedly mistaken specimens of *D. otome* to be *D. striatus*.

The other large clade of 15 species (the *D. marginlatus* clade) has two basal sister clades, one containing *D. marginlatus* and the sister species, *D. rubidus* and *D. pulcherrimus*. Anatomical comparison of these taxa showed that *D. rubidus* and *D. pulcherrimus* are synonyms (present study). In the sister clade of these three taxa, *D. pustulosus* is sister to a clade containing 11 taxa. Of these 11 taxa, there is a clade containing the sister species *D. cymatilis* and *D. semilunus* and their collective sister taxon, *D. phyllodes*. These three taxa all have a broad radula with a relatively narrow rachidian tooth that has a thin elongate central cusp that extends well beyond the basal denticles. The clade of nine taxa (*D. albopunctulatus* clade) contains *D. albopunctulatus* as sister to the remaining species. The remaining eight species include a polytomy with one clade within the polytomy containing another polytomy of four species (the *D. ornatus* clade).

#### 

##### Phylogenetic discussion

The present study confirms preliminary results by Kolb & Wägele (1998) and Willan (1997) with regard to the paraphyly of the Arminina. What the present study does appear to demonstrate is that the Heterodorididae, *Dirona picta*, *Hero formosa*, and *Pinufius rebus* are more closely related to the Proctonotidae and Madrellidae than to other Arminina. It is readily apparent that Arminidae is a very distinct lineage from the other Arminina. What is also intriguing is the implied proximity of relationship amongst the other Arminina that make up the sister group of the Doridina. This relationship certainly requires additional corroboration. Traditionally (Odhner,1939), the presence of an oral veil has been included as a feature that unites the Arminina. From the present study, it is evident that the presence of an oral veil is a plesiomorphic feature and that its retention cannot therefore be used as a derived feature supporting inclusion of these taxa in a clade. The presence of a caruncle in some Arminidae and Proctonotidae has also been suggested as a possible similarity. As stated previously, they are structurally different in the two groups, being arranged longitudinally in Proctonotidae and transversely in Arminidae. The present study shows that they have evolved independently and are clearly not homologous structures.

Kolb & Wägele (1998) provided the first phylogeny of the Arminidae. In their study, *Armina* was monophyletic, but *Dermatobranchus* was paraphyletic. In our study, which was based on using several outgroups rather than a single hypothetical ancestor, *Histiomena* is basal within the Arminidae, *Dermatobranchus* is monophyletic, and *Armina* remains unresolved as to whether it was monophyletic or paraphyletic.

On this basis, *Histiomena* is retained as a distinct taxon. *Armina* is unresolved in the present analysis, probably because of the limited taxon-sampling of *Armina* included in this study, or possibly also as a result of the fact that characters providing resolution within *Dermatobranchus* are insufficient to provide resolution within *Armina*. A more comprehensive analysis of all taxa should be undertaken before the systematics of this taxon is altered. *Armina* is in need of much systematic revision, particularly in regard to the status of most of the Indo-Pacific species. Most species are poorly understood morphologically and are known only from incomplete original descriptions. Important recent revisions of Atlantic taxa (Kolb, 1998; Kolb & Wägele,1998; Ardila & Diaz, 2002; Ardila & Valdés, 2004) have vastly improved our knowledge of Atlantic species. The work of Jensen (1997) is the only modern treatment of any Indo-Pacific species of *Armina*, but only five taxa were included in the study.

From the present analysis, *Dermatobranchus* represents a clade that is strongly supported by our second analysis. *Dermatobranchus arminus* is the most basal taxon and has radular characteristics similar to many *Armina* species and also retains hypobranchial lamellae, but has lost the branchial lamellae. The two largest subclades of *Dermatobranchus* are both supported by a single synapomorphy based on the shape of the rachidian or inner lateral teeth. Characteristics of the buccal armature in general are largely responsible for the support of the clades within *Dermatobranchus*, with a couple of exceptions. *Dermatobranchus otome* and *D. striatus* are sister species based on the shape of the penis and *D. rubidus* and *D. pulcherrimus*, which are here considered synonyms, have elongated lateral corners of the foot.

It appears that much of the diversification within *Dermatobranchus* is related to their specialization on different types of octocoral prey as evidenced by the number of synapomorphies related to the buccal armature. The buccal armature, including the shape of the jaws and form and location of the denticles along the masticatory border, and the shape of the radular teeth are likely to have driven diversification within *Dermatobranchus*. Data as to diversity of prey are lacking for most species of *Dermatobranchus*. However, within the *semistriatus* clade, *D. caesitius* has been observed feeding on xeniid soft corals, *D. albus* has been found on gorgonians and *D. albineus* has been found on *Eleutherobia*. Within the *D. ornatus* clade, *D. ornatus* has been found on gorgonians, whereas *D. caeruleomaculatus* and *D. dendronephthyphagus* have been observed feeding on *Dendronephthya*. *Dermatobranchus gonatophorus* has also been found feeding on *Eleutherobia.* Far more data on prey species and trophic variability are necessary to better understand evolutionary patterns of prey diversification within *Dermatobranchus*. It would be premature to try to map trophic evolution on the present phylogeny, given that the prey of most species remains unknown and there is scant evidence suggesting how generalized or specific prey preferences might be in *Dermatobranchus*. Evidence from the present study suggests that patterns of trophic diversification and evolution of buccal armature are related and the combination of these two processes has been responsible for the evolution of so many taxa within the Indo-Pacific. Similar patterns of the evolutionary diversification have been found in other taxa such as *Phyllodesmium*, where evolution of symbiosis and accommodation of zooxanthellae has been observed as a major adaptive force in the diversification of the clade (Rudman, 1981a, b, 1991; Moore & Gosliner, 2009).

The vast majority of species of *Dermatobranchus* are distributed in the Indo-Pacific tropics and the remaining species are restricted to adjacent temperate waters of Japan and South Africa. Interestingly, of the two temperate South African endemics, *D. arminus* is the most basal taxon, whereas *D. albineus* is a member of one of the most derived clades. The Japanese temperate endemics include members of the both the *D. albopunctulatus* and the *D. semistriatus* derived clades. A few taxa, where sufficient data are available, appear to be relatively widespread within the Indo-Pacific tropics. *Dermatobranchus albus*, *D. ornatus*, *D. gonatophorus*, *D. fortunatus*, and *D. rodmani* are known from the western Indian Ocean to the western Pacific. The largest number of species (16) has been found only from the western Pacific, but seven of these are known only from a single locality. It appears that the greatest diversity of *Dermatobranchus* species is restricted to the western Pacific, which is also the area of greatest octocoral diversity (G. Williams, pers. comm.). Two additional species are known only from a single locality, one species in the western Indian Ocean and another from the Gulf of Oman.

Sister group relationships, in cases where sufficient phylogenetic resolution is found, exhibit vicariant patterns. *Dermatobranchus otome* is known only from temperate Japan whereas its sister species, *D. striatus* is known from tropical Japan south to Papua New Guinea. Two other sister species pairs exhibit a similar pattern. *Dermatobranchus cymatilus* is known only from tropical Japan whereas its sister species, *D. semilunus*, is known from other areas of the western Pacific (the Philippines, Papua New Guinea, and eastern Malaysia). The other species pair, consisting of *D. gonatophorus* and *D. fasciatus*, includes species that are likely to be sympatric. In this case, *D. gonatophorus* is widely distributed, whereas *D. fasciatus* is known only from the Philippines and Papua New Guinea. With greater phylogenetic resolution and comprehensive surveys from other Indo-Pacific localities, more generalized patterns of distribution and vicariance are likely to emerge.

Several species are similar in external appearance, such as *D. fasciatus*, *D. semilunus*, *D. rodmani*, and *D. tuberculatus*. All of these species have an off-white body colour with a dark transverse band of colour across the dorsum. However, their internal anatomy, especially the structure of the radula, is radically different. Additionally, these taxa appear in very different clades of *Dermatobranchus*. These taxa, although not closely related to each other, are difficult to identify from living animals and are here considered to represent cryptic species. The presence of sympatric species with such external similarity is strongly suggestive that overall diversity of opisthobranchs may be masked by this similarity. It is likely that diversity of Indo-Pacific opisthobranchs may be even greater than previously estimated (Gosliner *et al.*, 2008).

The present study provides new insights into the systematics, morphological evolution, biology, and biogeography of arminid nudibranchs. The focus on *Dermatobranchus* has allowed us to essentially double the known diversity of this clade of nudibranchs whose members are specialist predators on octocoral coelenterates.
